# Discovery of Iboga-Derived Ligands for the Sigma‑2 Receptor

**DOI:** 10.1021/acsbiomedchemau.5c00011

**Published:** 2025-05-12

**Authors:** Alexander J. Hughes, Julie A. Talbert, Steven D. Townsend

**Affiliations:** Department of Chemistry, 5718 Vanderbilt University, Nashville, Tennessee 37235, United States

**Keywords:** molecular modeling, substance use disorder, ibogamine, iboga alkaloids, Sigma-2 receptor

## Abstract

Substance use disorder (SUD) is a mental condition that affects a person’s brain and behavior, leading to a lack of control with alcohol, drug, and medication use. The lack of efficacious and novel treatments for SUD is a growing concern. As such, we have synthesized a series of iboga alkaloid derivatives and evaluated their receptor binding profiles against a panel of CNS-based proteins, which were performed at the National Institute of Mental Health Psychoactive Drug Screening Program. These studies revealed two compounds that exhibit high affinity for the sigma-2 receptor and introduce the iboga alkaloid framework as a new scaffold for the development of sigma-2 ligands.

## Graphical abstract

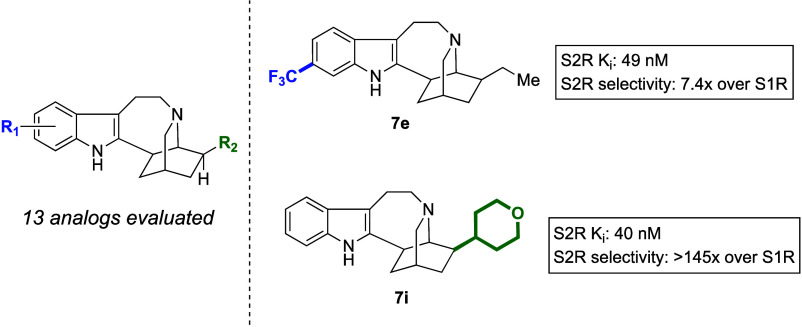

## Introduction

Substance use disorder (SUD), colloquially known as drug addiction, is a mental health condition where a person experiences a pattern of substance use that negatively affects their well-being. The substances most commonly over- or misused by human beings (including alcohol, amphetamines, caffeine, cocaine, nicotine, and opiates) increase the amount of dopamine released by neurons in the brain’s reward center.^1,2^ While the resultant feelings of pleasure or reward play a significant role in sustained use of the drug, it is only one variable in the cycle of addiction. Learning is also a major contributing factor in SUD. The intense reward sensation that accompanies intoxication includes a complementary learning response that associates drug use with pleasure.^3,4^ This relationship leads to increased drug administration to try to recapture the pleasure of the reward.

The search for novel and effective medicine to combat SUD is currently a society-level grand challenge. Unfortunately, there are limited treatments available for SUD that are not associated with weak efficacy and adverse side effects. Given the need for efficacious treatments for SUD, the search for effective therapeutics that are ligands for novel targets has been a major focus of the community. It is in this context that we took an interest in the sigma receptors (SRs) as they have been implicated in the neuropathology of addiction.^5^

Formerly described as a subtype of the opioid receptor and as a member of the phencyclidine/*N*-methyl-d-aspartate (PCP/NMDA) receptor family,^6^ the SRs are believed to modulate diverse physiological roles relevant to neurological disorders. There are two types of SRs: S1R and S2R, which have discrete pharmacological profiles.^7^ Human S1R has been cloned and sequenced with multiple crystal structures having been reported.^8,9^ Commercially available therapeutics, such as donepezil (**I**), fluvoxamine (**II**), and opipramol (**III**), possess high affinity for S1R (Figure 1A). While the therapeutic contribution of S1R binding for these drugs is not clear, clinical data supports efficacy, in part, to S1R activation.

**Figure 1:**
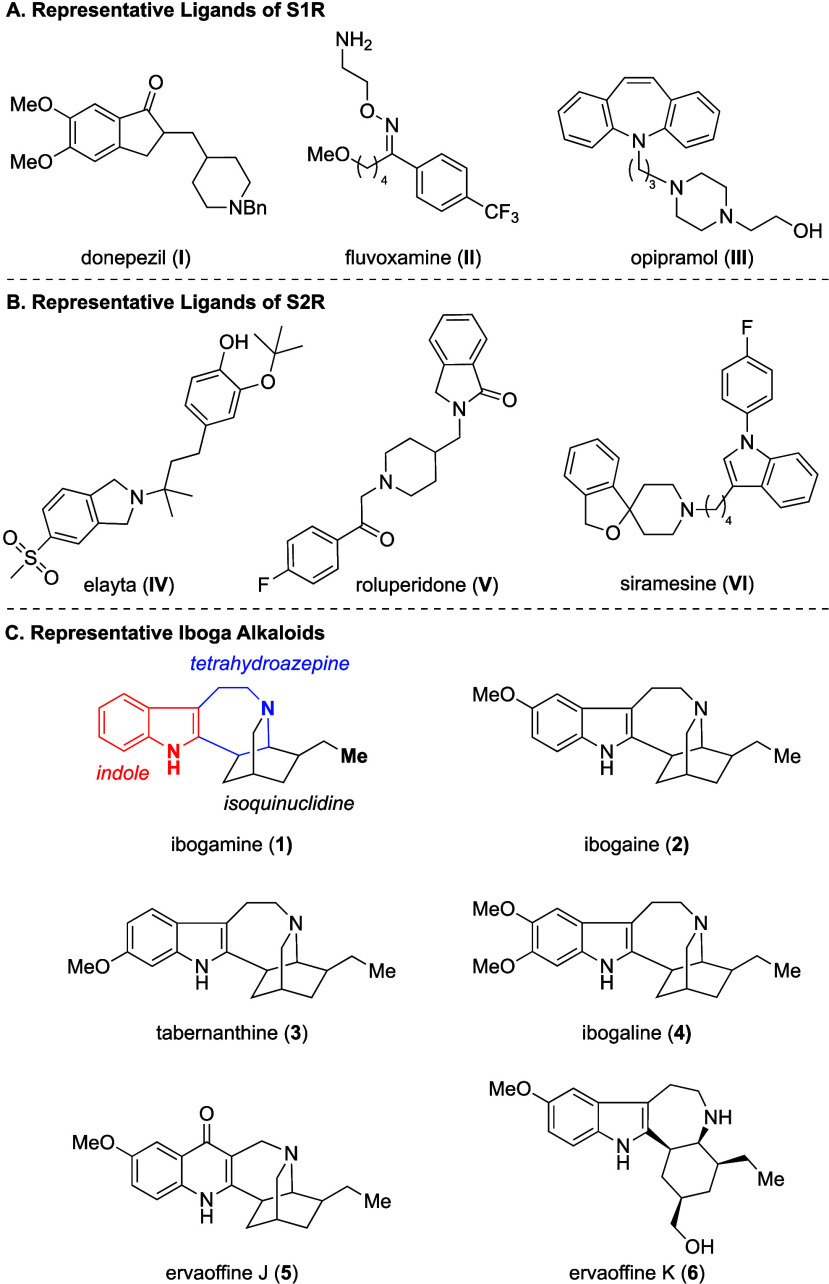
(A) Representative S1R ligands, (B) Representative S2R ligands, and (C) Select iboga alkaloids.

Similarly, S2R ligands have been shown to exhibit diverse clinically relevant properties in dopaminergic transmission, microglia activation, and neuroprotection.^10,11^ Specifically, small molecules that bind S2R have had beneficial effects in Alzheimer’s disease,^11–14^ neuropathic pain,^15–18^ traumatic brain injury,^19^ Huntington’s disease,^20^ and Parkinson’s disease,^21^ among others. As a promising therapeutic target, S2R modulators, Elayta (**IV**)^22,23^ and Roluperidone (**V**),^24^ are currently in clinical trial for treating Alzheimer’s disease and schizophrenia, respectively (Figure 1B). These discoveries have increased interest in S2R as a novel target for drug discovery. Moreover, the crystal structure of the bovine S2R has been solved, enabling *in silico* identification of probes to study this receptor using structure-based screens.^25^

In the context of SUD, certain S2R ligands appear to modulate the toxic effects of cocaine. Several studies have exhibited that ligands including UMB24, (±)-SM 21, and Siramesine (**VI**) can attenuate some of the behavioral effects of cocaine (Figure 1B).^26,27^ SN79, active at both S1R and S2R, has been found to reduce cocaine-induced effects and block methamphetamine-induced microglial activation.^28,29^ Although most studies have focused on cocaine and methamphetamine, growing evidence supports the role of the SR system in the misuse of other substances, such as ethanol. Accordingly, ligands of the SRs are being investigated as therapeutics for the SUD.

Iboga alkaloids are known to have activity at SRs with ibogamine (**1**), ibogaine (**2**), and tabernanthine (**3**) demonstrating varying affinity at S2R (K_i_ = 137 nM, 201 nM, and 194 nM, respectively).^30^ These compounds feature a signature molecular architecture incorporating indole, azepine, and isoquinuclidine within its core. Flagship members of the family, **1** and **2**, have long presented themselves as a challenge to synthetic chemists (Figure 1C). Indeed, we recently completed the total synthesis of ibogamine (**1**), in addition to tabernanthine (**3**), ibogaline (**4**), ervaoffine J (**5**), and ervaoffine K (**6**) (Figure 1C).^31–33^ In order to further explore the antiaddictive properties of this class of natural products, we embarked on an analog synthesis campaign. In the context of analog synthesis, previous efforts in the community have focused on simplifying the structure of the iboga alkaloids to access simplified analogs that often lacked the tetrahydroazepine and isoquinuclidine subunits.^34^ With an efficient synthesis of ibogamine, we sought to synthesize iboga analogs containing all three structural features, with functionalization at both C6 and C20.

Growing evidence implies that the SRs may be involved in the pathology of SUD. Described in this report are the syntheses of a series of ibogamine alkaloid derivatives that possess an affinity for the S2R. A broad screening of the candidates across a panel of 44 neurotransmitter receptors and transporters was conducted by the Psychoactive Drug Screening Program (PDSP) at the University of North Carolina (Chapel Hill) to confirm the CNS selectivity of the ligands. Lastly, we performed *in silico* molecular docking of the S2R ligands to identify residues critical to ligand recognition.

## Results and Discussion

Our analog synthesis campaign is focused on exploring the SAR of two areas of interest in the natural product scaffold (Scheme 1). Substitution of the 6 position of tryptamine containing natural products have been shown to reduce hallucinogenic potential.^35^ Accordingly, our first goal was to synthesize ibogamine derivatives functionalized at C6 of the indole, which are currently undescribed in the literature. Second, we sought to vary the alkyl functional group at C20 (ethyl is native) as manipulation of this functionality has been shown to reduce ibogaine’s toxicity.^36^ To generate a pool of analogs, our guiding synthetic analysis started with a late-stage C–N bond-forming reaction. Intermediate **8** can be synthesized by an intramolecular Friedel–Crafts alkylation using the conditions developed in our second-generation synthesis. Finally, this alkylation precursor **9** is envisioned to come from a Mitsunobu coupling of nosyl-protected tryptamine **10** and racemic enone **11**.

**Scheme 1:**
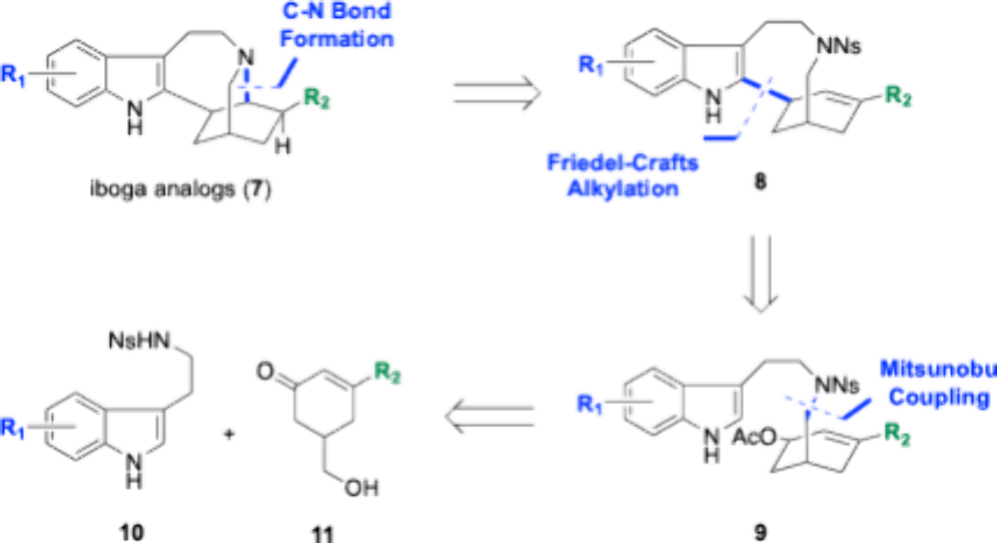
Retrosynthetic Analysis of Iboga Alkaloid Analogs

The first goal of this synthesis campaign was to modify the indole of ibogamine, which required the synthesis of substituted tryptamines. While there are many ways to make tryptamines, the reduction of 3-(2-nitroalkyl) indoles is likely the most used.^37^ Shown in Scheme 2A, indoles (**12a**–**e**) were alkylated at C3 with nitroalkene donor **13**.

**Scheme 2:**
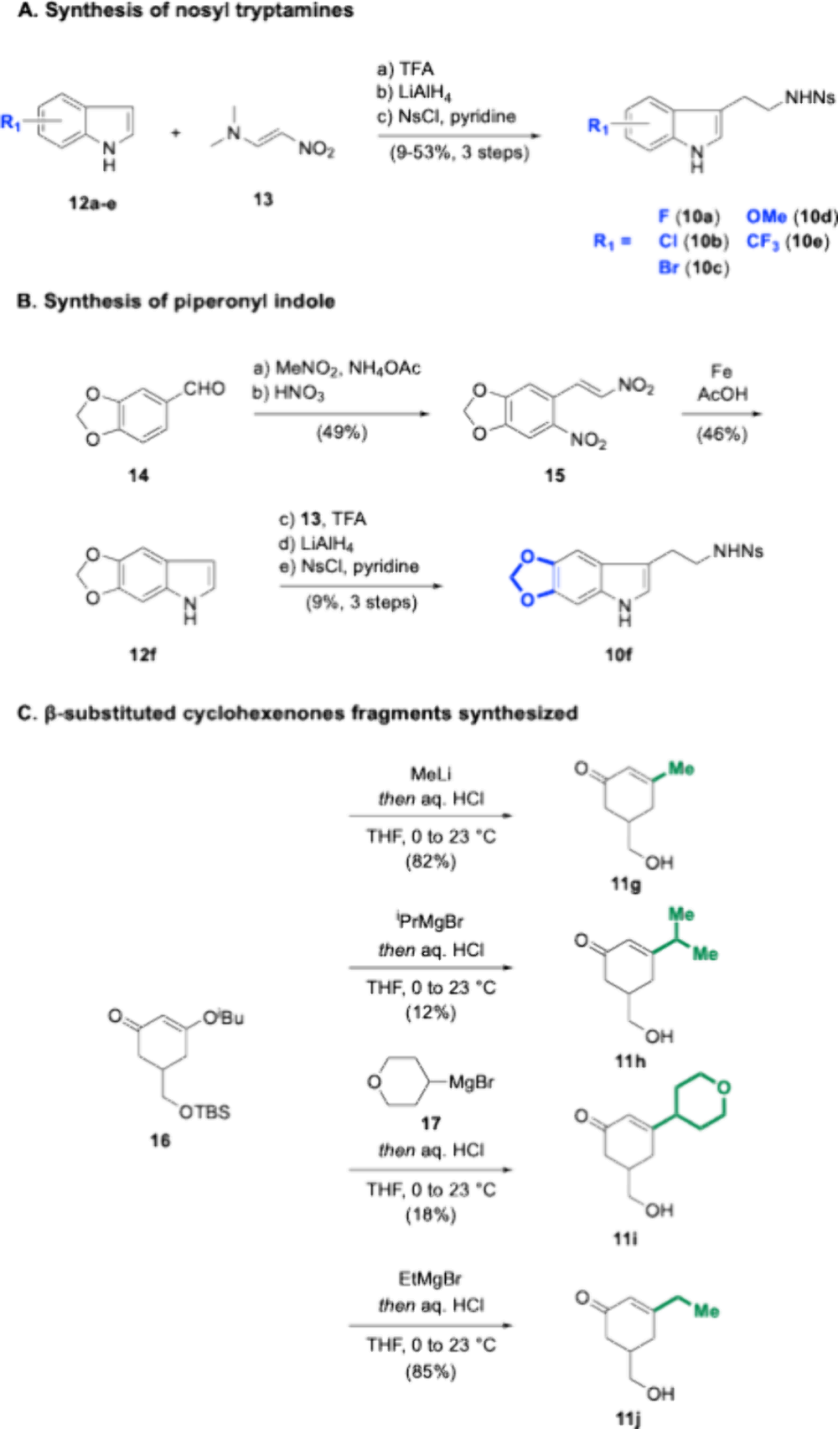
Ibogamine Analog Fragment Syntheses

The alkylated nitroalkenes were exhaustively reduced with excess LiAlH_4_ and nosyl protected to afford nosyl-protected tryptamines (**10a**–**e**).

One additional indole-modified ibogamine analog containing a piperonyl group was of interest. To synthesize piperonyl-substituted tryptamine **10f**, commercially available piperonal **14** was used as a starting material (Scheme 2B). Piperonal **14** was homologated via a Henry reaction and nitrated to give nitroarene **15**. Iron-catalyzed Batcho-Leimgruber indole synthesis gave access to piperonyl indole **12f** in gram quantities. Taken together, indoles **12a**–**f** were homologated to nosyl-protected tryptamines **10a**–**f** in 9–53% yields over three steps.

Substituting the ethyl group of ibogamine required access to various β-substituted cyclohexenones (Scheme 2C). The use of methyllithium as the nucleophile in the Stork-Danheiser transposition of enone **16** gave **11g** in a good yield. Both isopropyl magnesium bromide and tetrahydropyran-containing Grignard **17** gave the respective enones **11h** and **11i** in a lower yield. We hypothesize this is due to the preference of bulky, secondary Grignard reagents to act as bases, precluding 1,2-addition.

With a pool of nosyl-protected tryptamines and β-substituted cyclohexenones in hand, the next challenge was joining them via a Fukuyama-Mitsunobu coupling (Scheme 3). The conversions for these couplings were high, but purification for certain analogs proved challenging. The Fukuyama-Mitsunobu couplings were used to deliver the nine enones **18a**–**i**. Once the fragment couplings were accomplished, the next step was forming the key C–C bond from C2 of the indole to the isoquinuclidine. Closing the nine-membered macrocycle was accomplished in three steps with purification occurring at the end of the sequence. Luche reduction of enones **18a**–**i** was followed by acetylation to give a pool of allylic acetates. These macrocyclization precursors were subjected to magnesium­(II) perchlorate and CSA to generate macrocyclic alkenes **8a**–**i**.

**Scheme 3:**
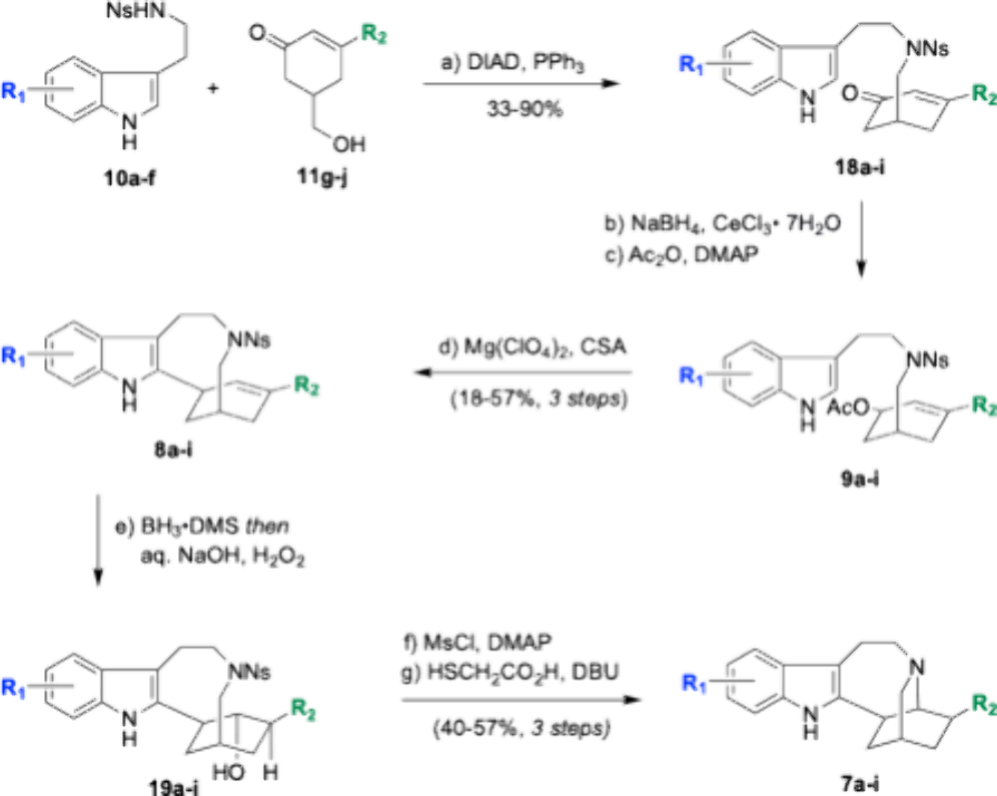
Ibogamine Analog Synthesis

To complete the iboga alkaloid skeleton, the last maneuver was forming the key C–N bond. This transformation was accomplished in a three-step sequence with only a single purification. The macrocyclic alkenes are first subjected to hydroboration-oxidation conditions to affect regio- and diastereoselective hydration. The resultant secondary alcohols (**19a**–**i**) were mesylated. Liberation of the amine is accompanied by concomitant ring closure. This sequence afforded the ibogamine analogs in a consistent 40 to 57% yield. This three-step method was successful in delivering nine ibogamine analogs (**7a**–**i**) including natural product (±)-tabernanthine (**3**). Approximately 10 to 50 mg of each compound were synthesized with complete diastereomeric control, and every analog was made in a single pass. Although linear, the syntheses were efficient and the route robust.

Our final objective was to selectively manipulate the oxidation state of the ibogamine indole as well as the basicity of its tertiary amine (Scheme 4). The Sames group reported diverse pharmacological activities of mitragynine analogs, which were accessed by selective oxidation of the natural product’s indole moiety.^38^ With gram-scale access to ibogamine, we successfully oxidized the indole to 4-quinolone (**20**) using a Witkop-Winterfeldt oxidation.^39^ Treatment of ibogamine with iodine and sodium carbonate achieved a selective C–H oxidation to deliver lactam **21**.^40^ To access 3-oxo-indolenine (**22**), DMDO was used as an oxidant. We found protonation of the tertiary amine to be critical to prevent N-oxidation. Finally, the indole N–H was alkylated with prenyl bromide using LiHMDS as a base to afford **23**.

**Scheme 4:**
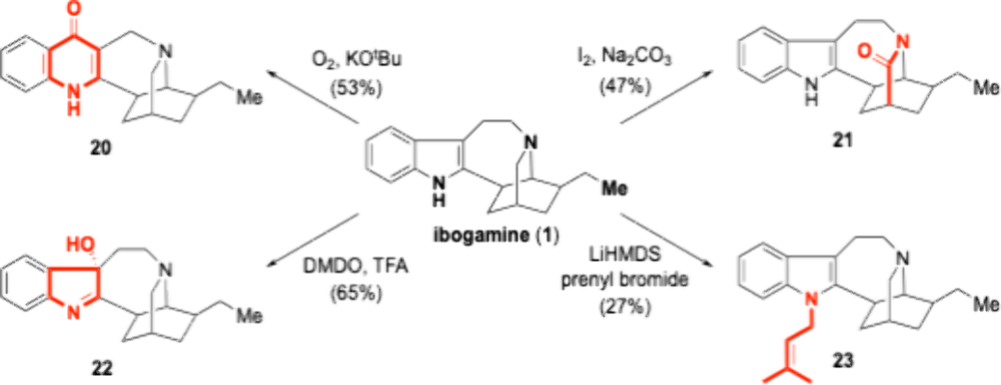
Synthesis of Analogs Directly from Ibogamine

Thirteen analogs, in addition to ibogamine itself (Figure 2), were submitted to the PDSP for biological evaluation. Primary radioligand displacement assays were run on 44 neurotransmitter receptors and transporters to give a snapshot of the receptor affinity for each ligand. Secondary assays were used to determine the inhibitory constant (K_i_) for each target where the primary assay found that the ibogamine analog displaced >50% of the radioligand used.

**Figure 2:**
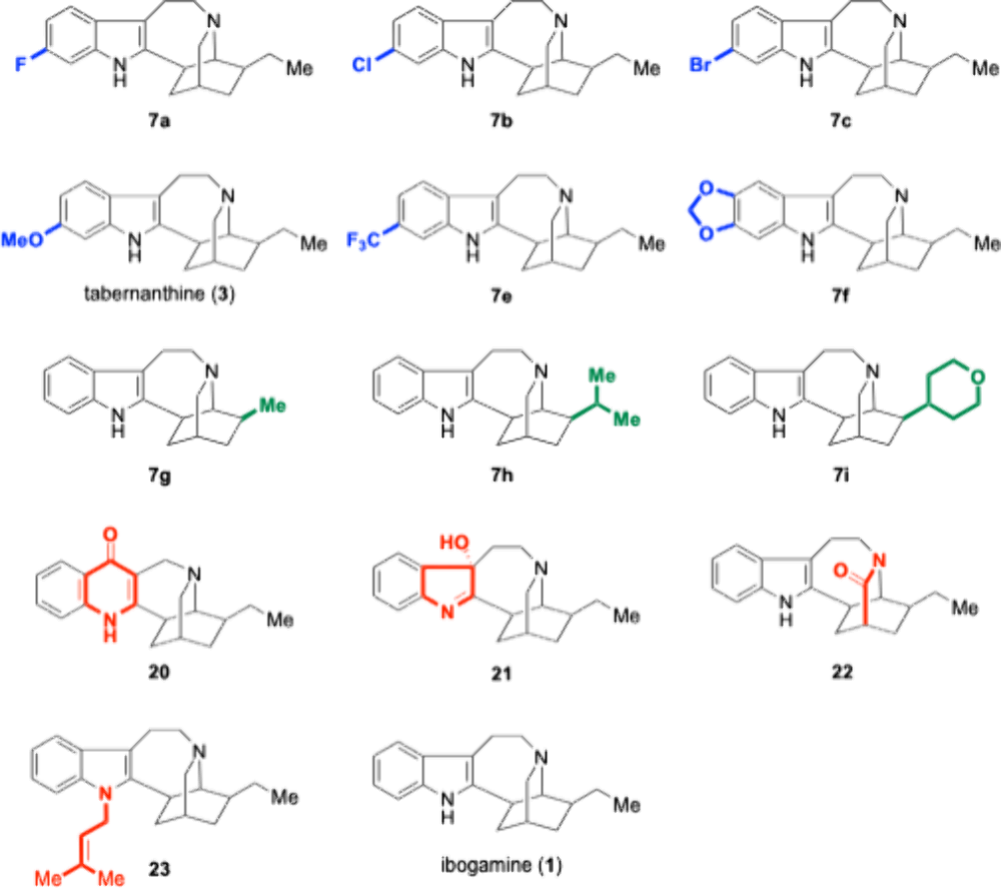
Iboga analogs evaluated.

The high-throughput screening data revealed that many of the analogs possessed high affinity for the S2R (Figure 3A, Table 1). Compound **7e** had an affinity of 49 nM for S2R and a selectivity of 7.4× over S1R. Analog **7i** was similarly potent at 40 nM but had greater S2R selectivity of 145× over S1R. Compound **7e** and **7i** possess activity outside of the SRs. Analog **7e** is most notably a nonselective ligand for α_2_ adrenergic receptors. It is most potent at the α_2C_ receptor (110 nM, Figure 3B). Compound **7i** does not possess notable α_2_ adrenergic activity but does have activity at the muscarinic acetylcholine receptors M_1_-M_5_ (Figure 3C). Detailed binding data of each analog synthesized can be found in the (Table S1). To better understand how these compounds bind to the S2R, we studied the *in silico* docking of the ligands to elucidate key binding interactions.

**Table 1:** Inhibitory constants and selectivity for S1R & S2R

compound #	S1R K_i_ ^a^	S2R K_i_ ^a^	S2R selectivity
ibogamine (1)	4106	319	12.9
7a	1652	98	16.8
7b	631	76	12.9
7c	920	91	10.1
tabernanthine (3)	5816	692	8.4
7e	361	49	7.4
7f	2879	803	3.6
7g	984	655	1.5
7h	>5800	192	>30.2
7i	>5800	40	>145
23	>5800	749	>7.7

a S1R and S2R K_i_ (nM).

**Figure 3:**
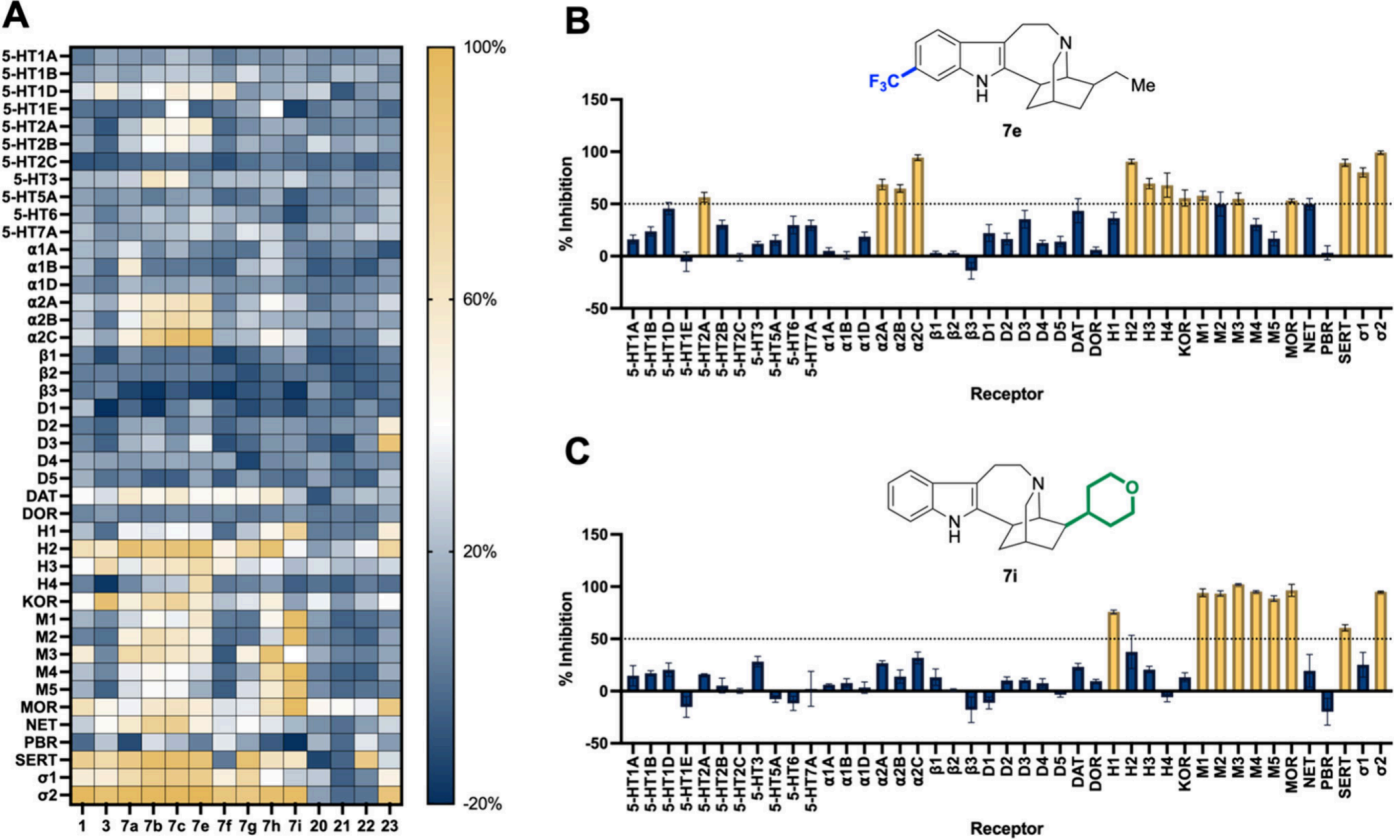
PDSP data reveal varying afiinity of our 14 compounds for 43 receptors. (A) Heatmap visualization of the percent radioligand displacement from each analog across the receptor panel. Colors displayed indicate low percent displacement (dark blue) to high percent displacement (yellow). (B) Receptometric profile of **7e**. (C) Recepterome profile of **7i**. For both B and C, receptors identified as hits (more than 50% displacement of standard radioligands) are colored yellow.

As the crystal structure for the human S2R has not been elucidated, we opted to use a homology model from the Swiss Model database (Entry ID: Q5BJF2 (SGMR2_HUMAN)).^41^ Compounds **7a**, **7b**, **7c**, **7e** (which contain C6 indole substituents), and **7i** (which has a tetrahydropyran-modified isoquinuclidine ring) were measured experimentally to have a K_i_ at the S2R < 100 nM. These five analogs and ibogamine (319 nM at S2R) were docked using Autodock Vina (Figure 4AB).^42,43^ All tested compounds were predicted to dock in the binding site of S2R (Figure 4AB). Notably, these five analogs were predicted to have higher docking scores than the parent natural product ibogamine at the S2R receptor. This agrees with the measured binding constants.

**Figure 4:**
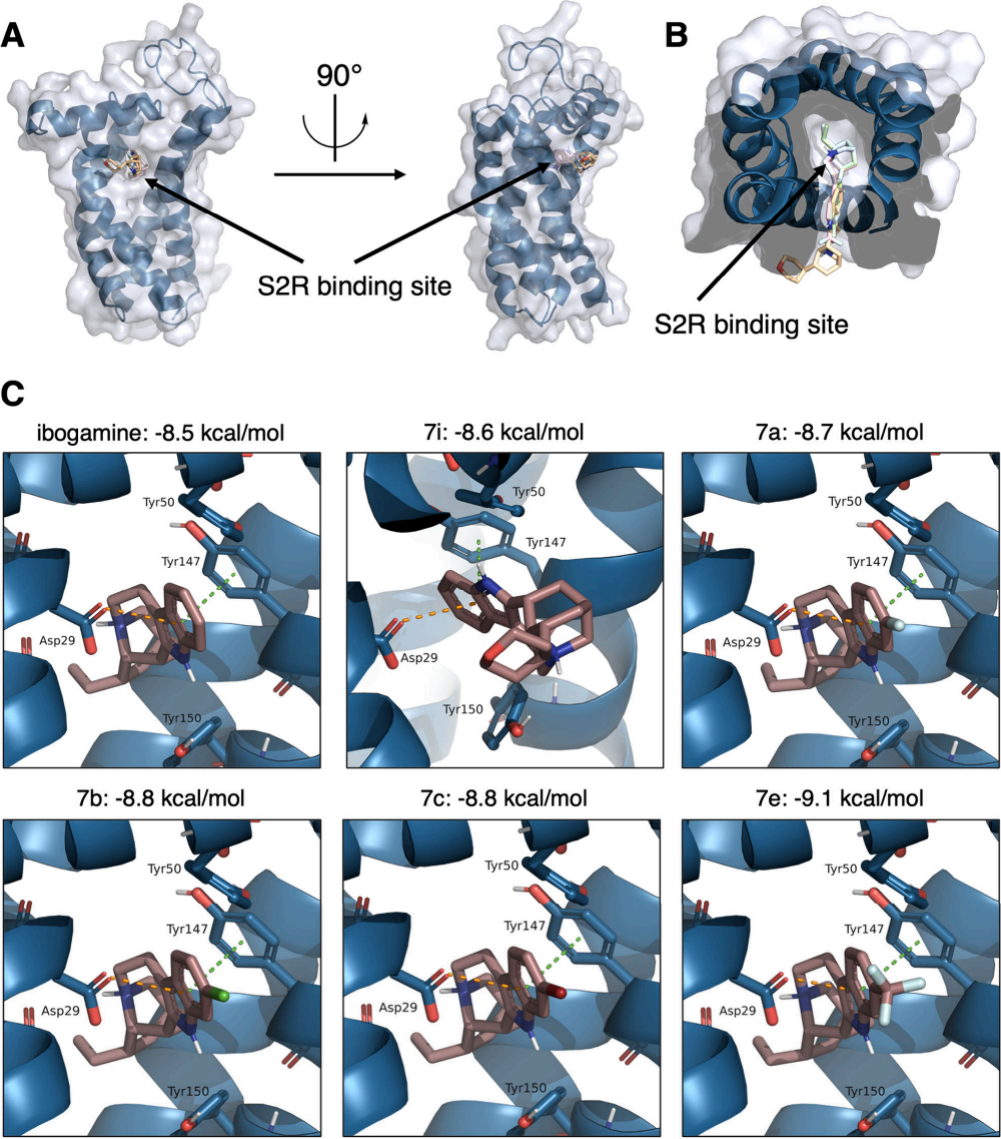
*In silico* modeling of the S2R receptor with select analogs. (A) Front and side views of the predicted S2R receptor with all six ligands docked. (B) Top view of predicted S2R receptor with all ligands docked. (C) Docking scores and poses for select analogs in the binding site of the S2R receptor. Green dotted lines indicate π-stacking interactions, and orange dotted lines indicate anion-π interactions. C = navy (S2R), mauve (ligand), O = red, N = blue, H = white.

First, we examined the positioning of all tested analogs together. Compounds **1**, **7a**, **7b**, **7c**, and **7e** are predicted to dock in almost identical positions (Figure 4AB). The indole portion of **7i** is also predicted to dock in the same location as that of the other compounds; however, its orientation is flipped. Gratifyingly, the indole moiety of each compound is predicted to interact with Asp29 in an anion-π interaction and with Tyr147 via π-stacking (Figure 4C). Interactions with both Asp29 and Tyr147 are commonly noted in the literature for S2R binding.^15,25,44–46^

Next, we investigated the substituent differences between **7a**, **7b**, **7c**, and **7e** at the C6 position of the indole (6-Fl, -Cl, -Br, and −CF_3_ substitution, respectively) (Figure 4C). To our delight, the compound with the highest experimental affinity (49 nM) of these four, **7e**, had the highest docking score (−9.1 kcal/mol). This can be attributed to the three-dimensional character and increased hydrophobicity of the −CF_3_ substituent. Indeed, Martin’s group found that their CF_3_-bearing ligands were predicted to dock with the −CF_3_ substituent facing the outside of the binding pocket, similar to our computational findings.^45^

Finally, we examined the predicted docking differences between ibogamine and **7i**, as these compounds solely differ at the C20 isoquinuclidine substituent (ethyl vs tetrahydropyran, respectively). As mentioned above, the predicted orientation of these ligands is different. Although the indoles are predicted to dock in the same location, the C20 substituents are facing opposite ways with ibogamine’s ethyl group facing the inside of the pocket and the tetrahydropyran of **7i** curling around the outside of S2R (Figure 4B). We believe that the reversal of the indole positioning for **7i** is due to the bulky nature of the tetrahydropyran. This forces the tetrahydropyran group to be docked outside the binding pocket. Although the indole faces opposite all other analogs, **7i** still has a slightly higher docking score (−8.6 kcal/mol) over ibogamine’s (−8.5 kcal/mol).

The modeling study herein concurs with the measured binding affinities of the reported ibogamine analogs at the S2R and sheds light on the critical receptor–ligand interactions. Interactions with Asp29 and Tyr147 as well as the hydrophobic nature of all analogs appear critical for ligands to have affinity for S2R.^15,25,44–46^ A complete list of specific ligand binding interactions and affinities can be found in .

In summary, we have synthesized 13 analogs of the natural product ibogamine. The biological activity of these compounds was evaluated by high-throughput screening, and their binding affinity to over 40 different CNS receptors was determined via radioligand displacement. Secondary assays were used to determine the inhibitory constants of the hits found in the radioligand binding assays. The most notable compounds were **7e** and **7i**, with compound **7i** having a K_i_ of 40 nM at the S2R and being more than 100-fold selective over S1R. Current work includes minimizing the muscarinic activity of compound **7i** to achieve a potent and selective inhibitor for the S2R. Additional ADME, DMPK, and angonist/antagonist testing of **7e** and **7i** will highlight any additional therapeutic parameters of interest for these compounds. Results in this regard will be reported in due course.

## Experimental Section

### General Procedure

All nonaqueous reactions were performed in flame-dried or oven-dried round-bottomed flasks under an atmosphere of nitrogen or argon, unless otherwise noted. Stainless steel syringes or cannulas were used to transfer air- and moisture-sensitive liquids. Reaction temperatures were controlled using a thermocouple thermometer and analog hot plate stirrer. Reactions were conducted at room temperature (rt, approximately 23 °C) unless otherwise noted. The anhydrous solvents used in the reactions were obtained from an MBraun MB-SPS 800 anhydrous Solvent System. Solvents for chromatography were of analytical grade and distilled under reduced pressure prior to use. Commercially available reagents were obtained from Aldrich, Fisher, TCI, TRC, and Carbosynth. Flash column chromatography was conducted as described^47^ using silica gel 230–400 mesh or Silica RediSep Rf flash columns on a CombiFlash Rf automated flash chromatography system. Thin layer chromatography (TLC) was performed using glass-backed 60-F254 silica gel plates obtained from Silicycle. Visualization of TLC plates was performed by UV (215 and 254 nm). ^1^H NMR and LCMS was used to determine that compounds were ≥95% pure.

### Instrumentation

^1^H NMR, ^13^C NMR, and 2D NMR spectra were recorded in the Vanderbilt Small Molecule NMR Facility on Bruker 400 and 600 MHz instruments. Structural assignments were made with additional information from gCOSY, gHSQC, and gHMBC experiments. Chemical shifts are reported in parts per million (ppm) of the δ scale. Spectra were recorded in CDCl_3_ using the solvent residual peak chemical shift as the internal standard (CDCl_3_: S7 7.26 ppm ^1^H, 77.0 ppm ^13^C) or in DMSO using the solvent as the internal standard in ^1^H NMR (DMSO: 2.50 ppm ^1^H) unless otherwise stated. ^1^H NMR spectral data are presented as follows: Chemical shifts (δ ppm), multiplicity (s = singlet, d = doublet, dd = doublet of doublets, dq = doublet of quadruplet, ddd = doublet of doublet of doublet, t = triplet, q = quartet, p = pentet, br = broad, m = multiplet), coupling constants (Hz), integration. High-resolution mass spectra (HRMS) were obtained from the Department of Chemistry, Vanderbilt University, using an LTQ-Orbitrap XL mass spectrometer.

## Supplementary Material







## Notes

Compounds, SMILEs, *K*_i_ values (XLSX)
Illustrated crystallographic information (ZIP)
PDSP comprehensive K_i_ data, recepterome profiles for analogs, docking methods, NIMH PDSP protocol book URL, compound characterization and preparation, spectra of synthetic products and intermediates, LCMS traces of lead compounds, and LCMS methods (PDF)

## †

Pfizer Chemical Research and Development, Pfizer Worldwide Research and Development, Groton, Connecticut 06340, United States
