# Supplementary material for: Discovery of Iboga-Derived Ligands for the Sigma‑2 Receptor
Source: ACS Bio Med Chem Au. 2025 May 12;5(3):379–86. doi: 10.1021/acsbiomedchemau.5c00011 (PMC12183592; doi:10.1021/acsbiomedchemau.5c00011)
Supplement: Supplementary file 3 [file bg5c00011_si_003.pdf]

Discovery of Iboga-Derived Ligands for the Sigma-2 Receptor

Alexander J. Hughes, Julie A. Talbert, and Steven D. Townsend*

Department of Chemistry, Vanderbilt University, 7330 Stevenson Center, Nashville, Tennessee 37235, United States

Email: steven.d.townsend@vanderbilt.edu

Supporting Information

Table of Contents

I. Title and authors S1

II. Additional Data

Table S1. PDSP Comprehensive K_i data S2

Figure S1. Recepterome profile for ibogamine (**1**), tabernanthine (**3**), & **20** S3

Figure S2. Recepterome profile for **21**, **22**, & **23** S4

Figure S3. Recepterome profile for **7a**, **7b**, and **7c** S5

Figure S4. Recepterome profile for **7f**, **7g**, and **7h** S6

Molecular docking methods and NIMH PDSP Protocol Book URL S7

Table S2. Summation of docking interactions for S2R S8

III. List of NMR spectra for new compounds S9-S10

IV. Compound characterization and preparation S11-S45

V. ^{1}H, ^{13}C, and 2D NMR spectra of synthetic products and intermediates S46-S145

VI. LCMS traces for compounds **7e** and **7i** S146-S147

VII. LCMS method S148

VIII. Supporting information references S149

Table S1. PDSP Comprehensive K_i data

	K_i (nM)													
	**ibogamine (1)**	**7a**	**7b**	**7c**	**tabernanthine (3)**	**7e**	**7f**	**7g**	**7h**	**7i**	**20**	**21**	**22**	**23**
**5-HT1D**	n.d.	n.d.	n.d.	7455.9	7657.73	n.d.	> 10,000	n.d.	n.d.	n.d.	n.d.	n.d.	n.d.	n.d.
**5-HT2A**	n.d.	n.d.	2997.78	n.d.	n.d.	1796.8	n.d.	n.d.	n.d.	n.d.	n.d.	n.d.	n.d.	n.d.
**5-HT3**	n.d.	n.d.	1014.38	1400.55	n.d.	n.d.	n.d.	n.d.	n.d.	n.d.	n.d.	n.d.	n.d.	n.d.
**Alpha1B**	n.d.	2090.26	n.d.	n.d.	n.d.	n.d.	n.d.	n.d.	n.d.	n.d.	n.d.	n.d.	n.d.	n.d.
**Alpha2A**	n.d.	4095.43	1720.28	1625.17	n.d.	1423.97	n.d.	n.d.	n.d.	n.d.	n.d.	n.d.	n.d.	n.d.
**Alpha2B**	n.d.	n.d.	2364.29	1624.05	n.d.	1650.06	n.d.	n.d.	n.d.	n.d.	n.d.	n.d.	n.d.	n.d.
**Alpha2C**	n.d.	623.45	1355.5	71.01	n.d.	110.36	n.d.	n.d.	n.d.	n.d.	n.d.	n.d.	n.d.	n.d.
**D2**	n.d.	n.d.	n.d.	n.d.	n.d.	n.d.	n.d.	n.d.	n.d.	n.d.	n.d.	n.d.	n.d.	2419.91
**D3**	n.d.	n.d.	n.d.	n.d.	n.d.	n.d.	n.d.	n.d.	n.d.	n.d.	n.d.	n.d.	n.d.	249.46
**DAT**	n.d.	1315.53	n.d.	806.68	n.d.	n.d.	n.d.	n.d.	2023.95	n.d.	n.d.	n.d.	n.d.	n.d.
**H1**	n.d.	n.d.	n.d.	n.d.	n.d.	n.d.	n.d.	n.d.	n.d.	881.66	n.d.	n.d.	n.d.	1712.77
**H3**	n.d.	n.d.	2880.05	3323.53	2303.03	2288.76	5413.77	n.d.	n.d.	n.d.	n.d.	n.d.	n.d.	n.d.
**H4**	n.d.	n.d.	n.d.	n.d.	n.d.	3033.89	n.d.	n.d.	n.d.	n.d.	n.d.	n.d.	n.d.	n.d.
**KOR**	n.d.	4772	1401.52	1044	319.23	1496.92	n.d.	n.d.	994.95	n.d.	n.d.	n.d.	n.d.	n.d.
**M1**	n.d.	n.d.	n.d.	n.d.	n.d.	4571.93	n.d.	n.d.	n.d.	138.01	n.d.	n.d.	n.d.	n.d.
**M2**	n.d.	2685.34	2936.97	8026.02	n.d.	n.d.	n.d.	n.d.	n.d.	149.73	n.d.	n.d.	n.d.	n.d.
**M3**	2462.07	733.84	810.96	1521.25	n.d.	1948.5	n.d.	n.d.	414.29	73.65	n.d.	n.d.	n.d.	n.d.
**M4**	n.d.	n.d.	n.d.	n.d.	n.d.	n.d.	n.d.	n.d.	415.81	37.89	n.d.	n.d.	n.d.	n.d.
**M5**	n.d.	n.d.	n.d.	n.d.	n.d.	n.d.	n.d.	n.d.	1098.75	224.34	n.d.	n.d.	n.d.	n.d.
**MOR**	2463.2	n.d.	4450.41	4210.17	n.d.	3810.66	n.d.	n.d.	3128.24	1806.76	n.d.	n.d.	n.d.	6444.66
**NET**	n.d.	5701.64	1794.73	1745.02	n.d.	n.d.	n.d.	n.d.	n.d.	n.d.	n.d.	n.d.	n.d.	n.d.
**SERT**	272.46	153.11	176.89	161.58	1301.37	205.97	n.d.	370.77	708.6	1710.8	n.d.	1384.52	n.d.	n.d.
**Sigma 1**	4105.82	1653.48	630.81	920.45	5815.67	360.99	2878.72	983.56	n.d.	n.d.	n.d.	n.d.	n.d.	n.d.
**Sigma 2**	803.16	98.08	75.65	91.35	692.63	49.18	803.16	655.54	191.56	39.9	n.d.	n.d.	n.d.	749.03

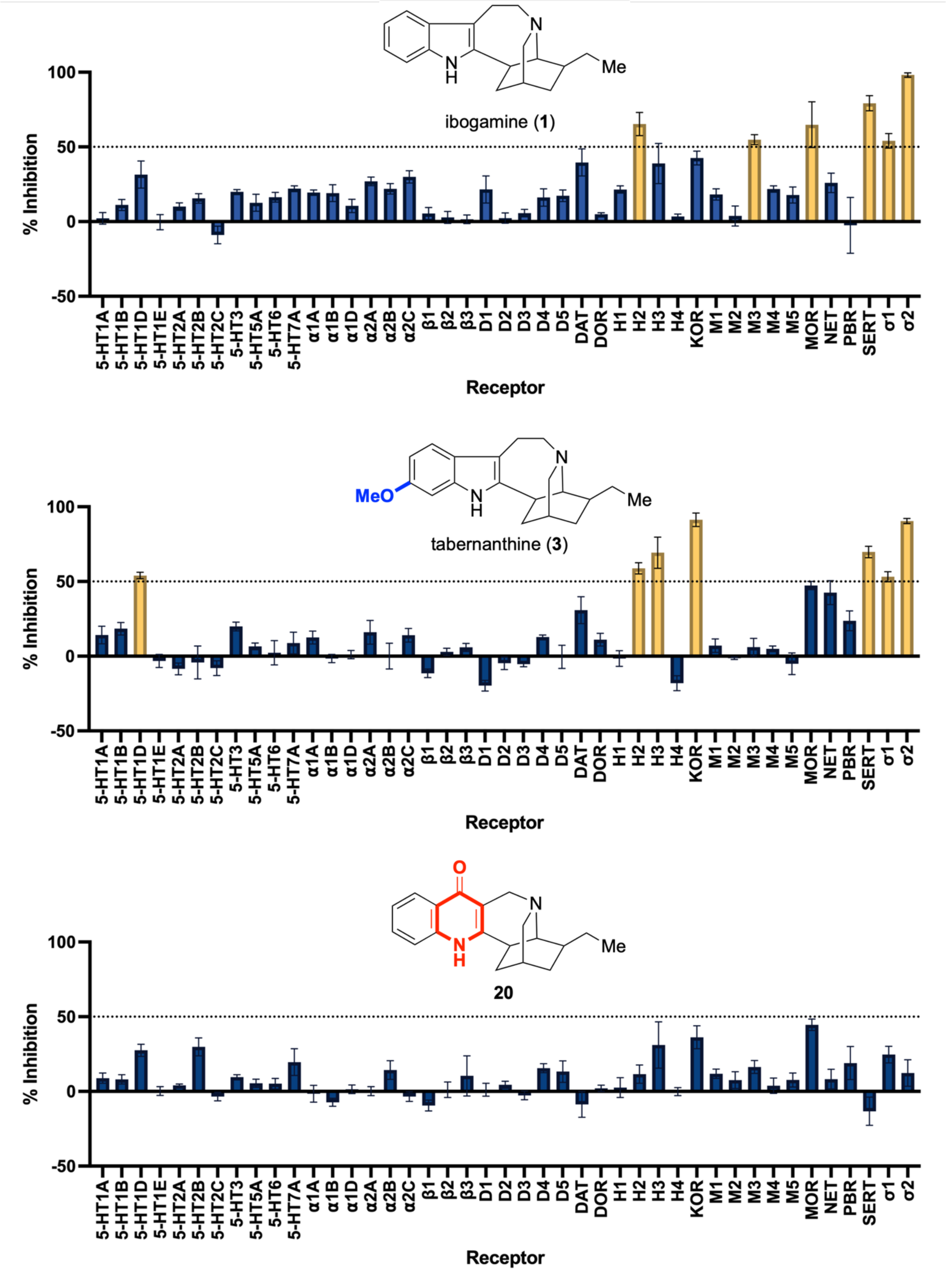

Figure S1. Recepterome profile for ibogamine (**1**), tabernanthine (**3**), and **20**.

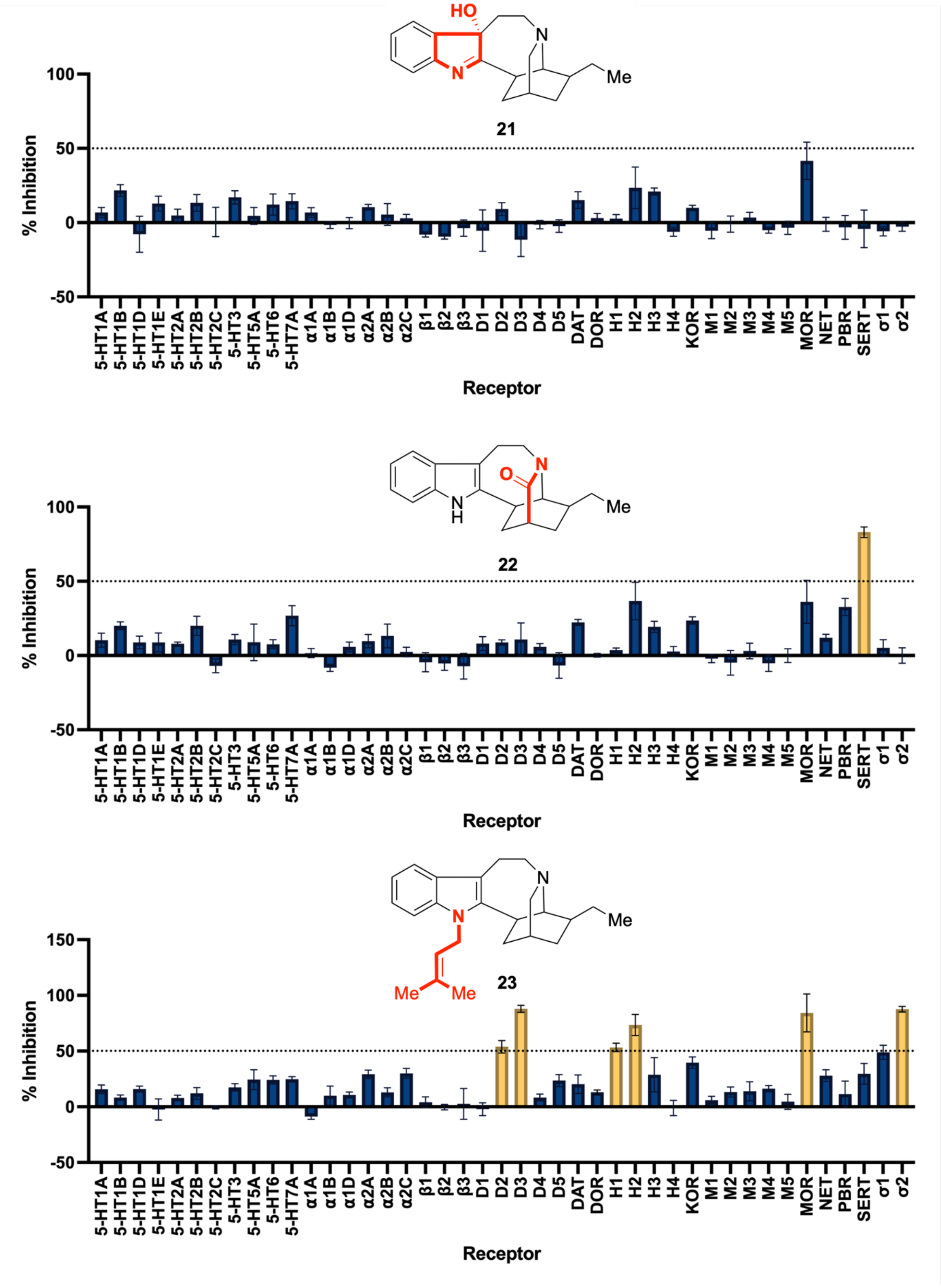

Figure S2. Recepterome profile for **21**, **22**, and **23**.

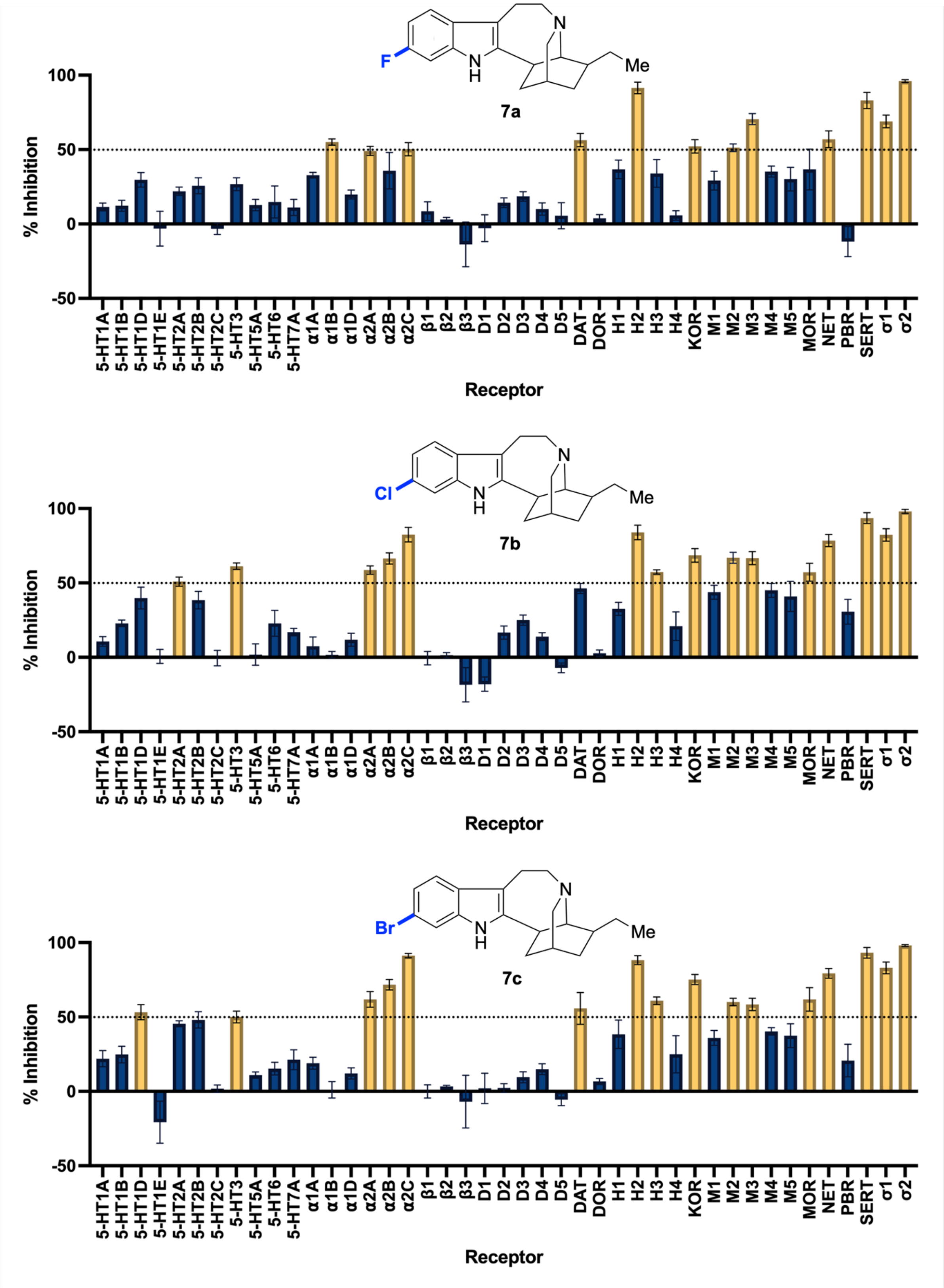

Figure S3. Recepterome profile for **7a**, **7b**, and **7c**.

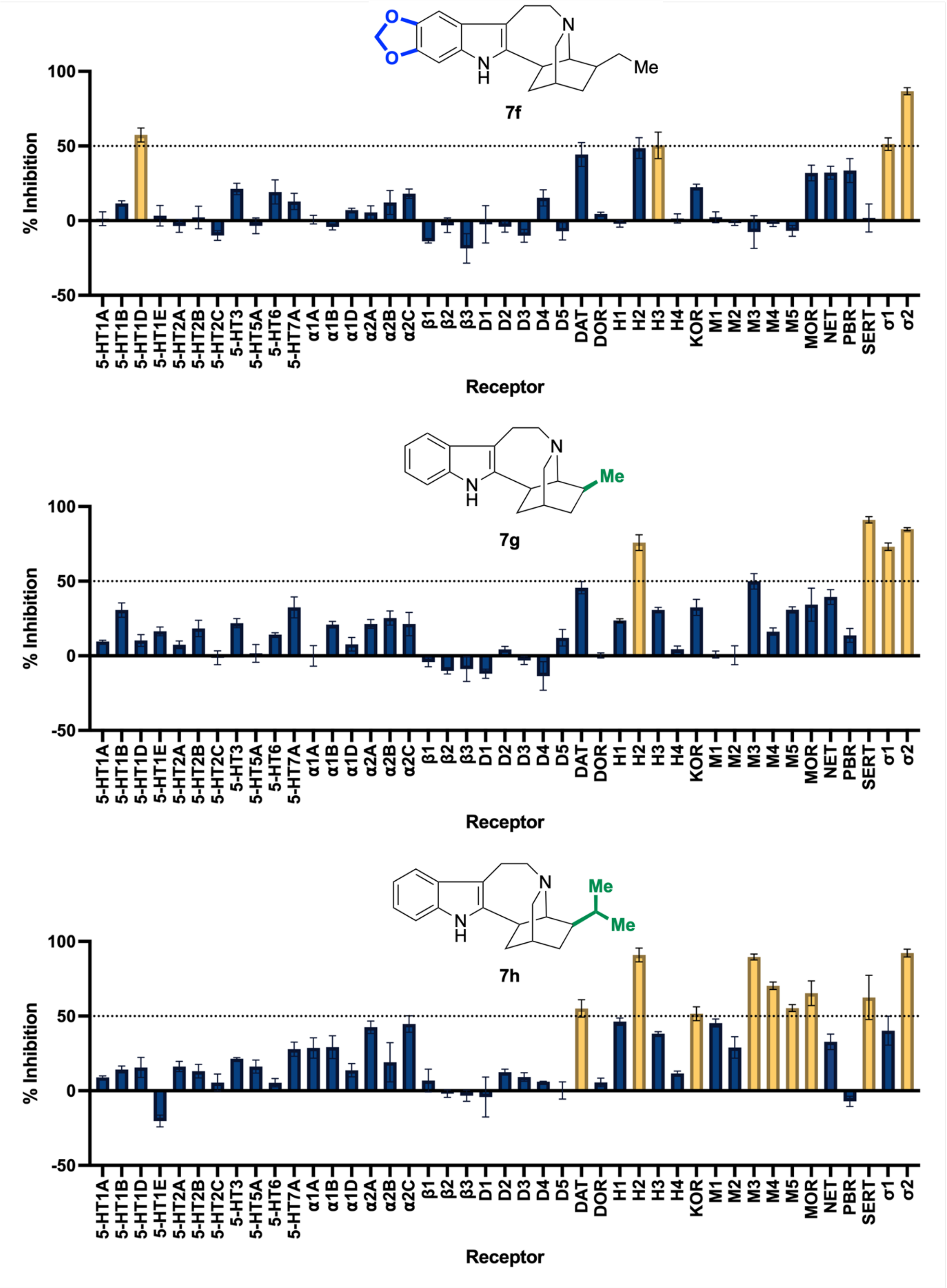

Figure S4. Recepterome profile for **7f**, **7g**, and **7h**.

Structural Prediction and Molecular Docking. The structure of the S2R homology modeled structure was acquired from Swiss Model (Entry ID: Q5BJF2 (SGMR2_HUMAN)).[1] Chain B, ligands, and water were deleted in AutoDock Tools and polar hydrogens and Gasteiger charges were added before exporting the protein for docking. Structures for all analogs with correct protonation and stereochemistry were made in ChemDraw (version 22.2.0), then converted to 3D structures and optimized using the MMFF94 force field in Avogadro (version 1.2.0).[2] Each enantiomer for each analog was optimized 3 individual times in Avogadro giving 6 structures. The structure with the lowest energy calculated by Avogadro was taken into the docking. Docking was performed using AutoDock Vina (version 1.1.2) with a grid box of 30 Å x 30 Å x 30 Å around the S2R binding site.[3,4] All visualization was performed using PyMOL (version 2.5.7).

NIMH PDSP Protocol Book:
https://pdspdb.unc.edu/pdspweb/content/PDSP%20Protocols%20II%202013-03-28.pdf

Table S2. Summation of docking interactions for S2R as calculated by AutoDock Vina.

**Compound**	**K_i (nM)**	**Docking Score (kcal/mol)**	**Interactions**	
			**Anion-π**	**π-π stacking**
Ibogamine	319	-8.5	Asp29	Tyr147
7i	40	-8.6	Asp29	Tyr147
7a	98	-8.7	Asp29	Tyr147
7b	76	-8.8	Asp29	Tyr147
7c	91	-8.8	Asp29	Tyr147
7e	49	-9.1	Asp29	Tyr147

List of NMR spectra for new compounds

^{1}H, ^{13}C of compound **24** S46-S47

^{1}H, ^{13}C of compound **15** S48-S49

^{1}H, ^{13}C of compound **12f** S50-S51

^{1}H, ^{13}C of compound **10a** S52-S53

^{1}H, ^{13}C of compound **10b** S54-S55

^{1}H, ^{13}C of compound **10c** S56-S57

^{1}H, ^{13}C of compound **10d** S58-S59

^{1}H, ^{13}C of compound **10e** S60-S61

^{1}H, ^{13}C of compound **10f** S62-S63

^{1}H, ^{13}C of compound **11g** S64-S65

^{1}H, ^{13}C of compound **11h** S66-S67

^{1}H, ^{13}C of compound **18a** S68-S69

^{1}H, ^{13}C of compound **18b** S70-S71

^{1}H, ^{13}C of compound **18c** S72-S73

^{1}H, ^{13}C of compound **18d** S74-S75

^{1}H, ^{13}C of compound **18e** S76-S77

^{1}H, ^{13}C of compound **18f** S78-S79

^{1}H, ^{13}C of compound **18g** S80-S81

^{1}H, ^{13}C of compound **18h** S82-S83

^{1}H, ^{13}C of compound **18i** S84-S85

^{1}H, ^{13}C of compound **8a** S86-S87

^{1}H, ^{13}C of compound **8b** S88-S89

^{1}H, ^{13}C of compound **8c** S90-S91

^{1}H, ^{13}C of compound **8d** S92-S93

^{1}H, ^{13}C of compound **8e** S94-S95

^{1}H, ^{13}C of compound **8f** S96-S97

^{1}H, ^{13}C of compound **8g** S98-S99

^{1}H, ^{13}C of compound **8h** S100-S101

^{1}H, ^{13}C of compound **8i** S102-S103

^{1}H, ^{13}C, and ^{19}F of compound **7a** S104-S106

^{1}H, ^{13}C of compound **7b** S107-S108

^{1}H, ^{13}C of compound **7c** S109-S110

^{1}H, ^{13}C of compound tabernanthine **3** S111-S112

^{1}H, ^{13}C, and ^{19}F of compound **7e** S113-S115

^{1}H, ^{13}C of compound **7f** S116-S117

^{1}H, ^{13}C of compound **7g** S118-S119

^{1}H, ^{13}C of compound **7h** S120-S121

^{1}H, ^{13}C and 2D spectra of compound **7i** S122-S125

^{1}H, ^{13}C and 2D spectra of compound **20** S126-S130

^{1}H, ^{13}C and 2D spectra of compound **21** S131-S135

^{1}H, ^{13}C and 2D spectra of compound **22** S136-S140

^{1}H, ^{13}C and 2D spectra of compound **23** S141-S145

(*E*)-5-(2-nitrovinyl)benzo[*d*][1,3]dioxole (24):

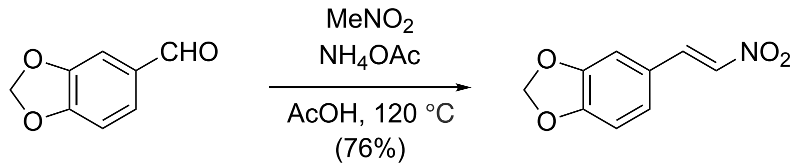

14 **24**

To a 500 mL round bottom flask was added piperonal **14** (5.0 g, 1.00 equiv., 33 mmol), acetic acid (100 mL), nitromethane (6.1 g, 5.4 mL, 3.00 equiv., 100 mmol), and ammonium acetate (6.9 g, 2.70 equiv., 90 mmol). The flask was placed in a 120 °C preheated oil bath where it stirred for 4h. The reaction was cooled to 23 °C and poured into ice cold water (400 mL). The slurry was filtered and washed with cold water (4 x 100 mL). The solids were dried under high vacuum to afford nitroalkene **24** (4.9 g, 76% yield, 25 mmol) as a yellow solid. ^{1}H NMR (600 MHz, DMSO) δ 8.13 (d, *J* = 13.5 Hz, 1H), 8.06 (d, *J* = 13.5 Hz, 1H), 7.53 (d, *J* = 1.7 Hz, 1H), 7.39 (dd, *J* = 8.0, 1.8 Hz, 1H), 7.03 (d, *J* = 8.0 Hz, 1H), 6.13 (s, 2H). ^{13}C NMR (151 MHz, DMSO) δ 150.9, 148.2, 139.6, 136.2, 127.8, 124.4, 108.8, 107.5, 102.1.

(*E*)-5-nitro-6-(2-nitrovinyl)benzo[*d*][1,3]dioxole (15):

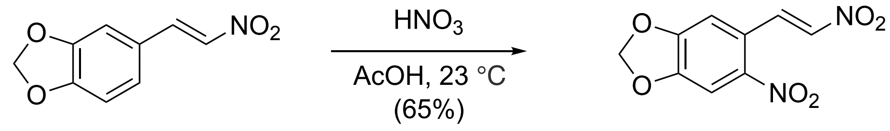

24 **15**

To a 250 mL round bottom flask was added nitroalkene **24** (4.90 g, 1.00 equiv., 25.4 mmol) and acetic acid (30 mL). The reaction was cooled to 0 °C and nitric acid (24.0 g, 17.0 mL, 15.0 equiv., 381 mmol) was added slowly. The reaction warmed to 23 °C where it stirred for 3h. The reaction was poured into ice water (300 mL) and filtered washing with cold water (3 x 50 mL). The crude solid was recrystallized from ethanol to afford nitroarene **15** (3.90 g, 65% yield, 16.4 mmol) as a yellow solid. ^{1}H NMR (600 MHz, DMSO) δ 8.34 (d, *J* = 13.4 Hz, 1H), 8.11 (d, *J* = 13.4 Hz, 1H), 7.78 (s, 1H), 7.57 (s, 1H), 6.32 (s, 2H). ^{13}C NMR (151 MHz, DMSO) δ 151.9, 150.1, 144.1, 139.9, 135.1, 121.5, 107.9, 105.9, 104.2.

5*H*-[1,3]dioxolo[4,5-*f*]indole (12f):

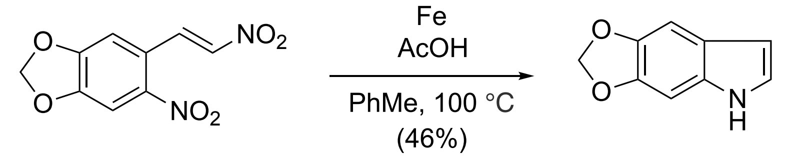

To a 500 mL round bottom flask was added nitroarene **15** (3.38 g, 1.00 equiv., 14.2 mmol), iron (11.9 g, 15.0 equiv., 213 mmol), and silica (15 g). To the reaction flask was added a degassed mixture of toluene (140 mL) and acetic acid (80 mL). The reaction was heated to 110 °C for 30 minutes before cooling and diluting with water (300 mL). Solid sodium bicarbonate was added until all of the acetic acid was consumed. The biphasic mixture was passed through celite washing with EtOAc. The organic was separated, washed with brine, dried over sodium sulfate, and concentrated *in vacuo*. The crude material was purified via flash column chromatography (9:1 to 1:3 Hexane/DCM) to afford indole **12f** (1.06 g, 46% yield, 6.58 mmol) as a white solid. R_f 0.8 (1:1 Hexane/EtOAc). ^1H NMR (600 MHz, $CDCl_3$) δ 8.01 (s, 1H), 7.08 (t, J = 2.8 Hz, 1H), 7.01 (s, 1H), 6.85 (s, 1H), 6.46 – 6.41 (m, 1H), 5.93 (s, 2H). ^{13}C NMR (151 MHz, $CDCl_3$) δ 145.1, 143.2, 130.8, 122.9, 121.8, 103.0, 100.7, 99.3, 92.0.

General procedure A for syntheses of nosyl tryptamines:

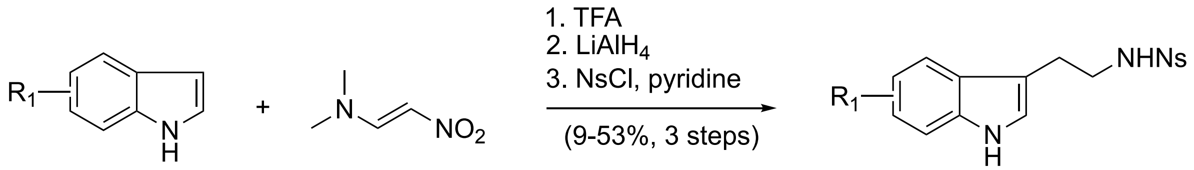

12a-f **13** **10a-f**

To a 25 mL round bottom flask was added enamine **13** (0.750 g, 6.46 mmol, 1.0 equiv.), indole **12a-f** (6.46 mmol, 1.0 equiv.), and DCM (5.00 mL). To the stirring solution was added TFA (3.68 g, 2.49 mL, 32.3 mmol, 5.00 equiv.) The reaction stirred for 45 minutes before slowly pouring into a rapidly stirring saturated sodium bicarbonate solution (100 mL). The aqueous layer was extracted with EtOAc (3 x 100 mL). The organics were combined and washed with sodium bicarbonate (1 x 100 mL), brine (1 x 100 mL), dried over sodium sulfate and concentrated. The crude material was triturated in cold DCM (8 mL) and filtered. The solids were washed with cold 1:1 DCM/hexane (3 x 10 mL) and dried under high vacuum. The crude material was used with no further purification.

To a 100 mL round bottom flask was added crude nitro alkene (1.0 equiv.) and THF to make a 0.1 M solution. Solid lithium aluminum hydride (6.0 equiv.) was added in two portions. The resulting suspension was heated to 60 °C and stirred for 5 hours. The reaction was cooled to 0 °C and quenched via dropwise addition with water (1.0 mL), 2 M NaOH (1.0 mL), and water (3.0 mL). Magnesium sulfate was added and stirred for 20 minutes. The reaction mixture was then filtered over celite washing with THF (3 x 50 mL). The solution was concentrated and the crude material used with no further purification.

To a 50 mL round bottom flask was added the crude tryptamine (1.00 equiv.) and pyridine to make a 0.4 M solution. The reaction mixture was cooled to 0 °C and 4-nitrobenzenesulfonyl chloride (1.00 equiv.) was added in two portions. The reaction stirred at 0 °C for two hours before diluting with EtOAc (100 mL) and washing with 2 N HCl (4 x 50 mL), sodium bicarbonate (50 mL), and brine (50 mL). The organics were dried over sodium sulfate and adsorbed onto celite. Flash chromatography of the crude material eluting with a gradient of EtOAc/Hexane (1:10 to 1:1) on a 80g Combiflash ISCO column gave nosyl tryptamines **12a-f**.

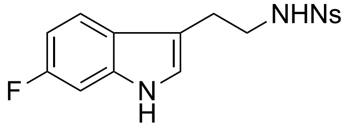

10a

The title compound was prepared following general procedure A and isolated as an orange foam (1.02 g, 2.81 mmol, 43% yield). ^{1}H NMR (600 MHz, $CDCl_3$) δ 8.17 (d, *J* = 8.8 Hz, 2H), 8.06 (s, 1H), 7.83 (d, *J* = 8.7 Hz, 2H), 7.29 – 7.26 (m, 1H), 6.99 (dd, *J* = 9.5, 2.2 Hz, 1H), 6.97 – 6.95 (m, 1H), 6.81 (td, *J* = 8.9, 1.9 Hz, 1H), 4.65 (t, *J* = 5.9 Hz, 1H), 3.34 (d, *J* = 6.3 Hz, 2H), 2.93 (t, *J* = 6.5 Hz, 2H). ^{13}C NMR (151 MHz, $CDCl_3$) δ 161.0, 159.5, 149.9, 145.6, 136.5 (d, $J_{C,F}$ = 12.1 Hz), 128.1, 124.2, 123.4, 123.0 (d, $J_{C,F}$ = 3.0 Hz), 119.2 (d, $J_{C,F}$ = 9.0 Hz), 111.5, 108.7 (d, $J_{C,F}$ = 24.2 Hz), 97.9 (d, $J_{C,F}$ = 25.7 Hz), 43.2, 25.7. HRMS (ESI) calcd. for $C_{16}H_{14}FN_3O_4S$ $(M)^{\bullet}$ 363.0689, found 363.0684 m/z.

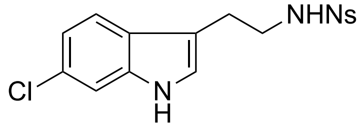

10b

The title compound was prepared following general procedure A and isolated as an orange foam (1.09 g, 2.87 mmol, 44% yield). ^{1}H NMR (600 MHz, DMSO) δ 10.92 (s, 1H), 8.32 ($CHCl_3$), 8.24 (d, *J* = 8.8 Hz, 2H), 8.09 (t, *J* = 5.6 Hz, 1H), 7.88 (d, *J* = 8.9 Hz, 2H), 7.37 (d, *J* = 8.4 Hz, 1H), 7.26 (d, *J* = 1.9 Hz, 1H), 7.13 (d, *J* = 2.3 Hz, 1H), 6.93 (dd, *J* = 8.4, 1.9 Hz, 1H), 3.12 (td, *J* = 7.2, 5.6

Hz, 2H), 2.78 (t, *J* = 7.1 Hz, 2H). ^{13}C NMR (151 MHz, DMSO) δ 149.1, 146.1, 136.4, 127.7, 127.6, 125.7, 125.7, 124.4, 124.1, 119.4, 118.5, 111.1, 110.9, 79.2 ($CHCl_3$), 43.2, 25.1. HRMS (ESI) calcd. for $C_{16}H_{14}ClN_3O_4S$ $(M)^{\bullet}$ 379.0394, found 379.0393 m/z.

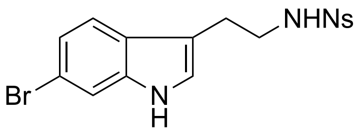

10c

The title compound was prepared following general procedure A and isolated as an orange solid (1.45 g, 3.42 mmol, 53% yield). ^{1}H NMR (600 MHz, DMSO) δ 10.93 (s, 1H), 8.32 ($CHCl_3$), 8.24 (d, *J* = 8.8 Hz, 2H), 8.09 (t, *J* = 5.6 Hz, 1H), 7.88 (d, *J* = 8.8 Hz, 2H), 7.41 (d, *J* = 1.8 Hz, 1H), 7.32 (d, *J* = 8.4 Hz, 1H), 7.12 (d, *J* = 2.3 Hz, 1H), 7.04 (dd, *J* = 8.4, 1.8 Hz, 1H), 3.11 (td, *J* = 7.1, 5.5 Hz, 2H), 2.77 (t, *J* = 7.1 Hz, 2H). ^{13}C NMR (151 MHz, DMSO) δ 149.1, 146.1, 136.9, 127.6, 125.9, 124.3, 124.1, 121.1, 119.8, 113.9, 113.7, 111.1, 79.2 ($CHCl_3$), 43.2, 25.0. HRMS (ESI) calcd. for $C_{16}H_{14}BrN_3O_4S$ $(M)^{\bullet}$ 422.9888, found 422.9885 m/z.

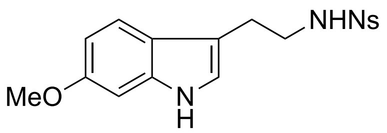

10d

The title compound was prepared following general procedure A and isolated as an orange foam (294 mg, 0.783 mmol, 12% yield). ^{1}H NMR (600 MHz, DMSO) δ 10.55 (s, 1H), 8.32 ($CHCl_3$), 8.27 – 8.22 (m, 2H), 8.07 (t, *J* = 5.6 Hz, 1H), 7.91 – 7.86 (m, 2H), 7.21 (d, *J* = 8.6 Hz, 1H), 6.93 (d, *J* = 2.3 Hz, 1H), 6.73 (d, *J* = 2.2 Hz, 1H), 6.57 (dd, *J* = 8.6, 2.3 Hz, 1H), 3.72 (s, 3H), 3.10 (td, *J* = 7.3, 5.6 Hz, 2H), 2.74 (t, *J* = 7.3 Hz, 2H). ^{13}C NMR (151 MHz, DMSO) δ 155.4, 149.1, 146.1, 136.8, 127.7, 127.6, 124.2, 121.7, 121.2, 118.5, 110.6, 108.5, 94.3, 79.2 ($CHCl_3$), 55.0, 43.3, 25.3. HRMS (ESI) calcd. for $C_{17}H_{17}N_3O_5S$ $(M + H)^{+}$ 376.0967, found 376.0961 m/z.

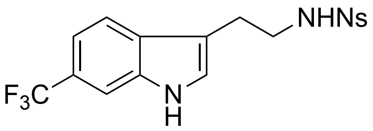

10e

The title compound was prepared following general procedure A and isolated as an orange foam (280 mg, 0.677 mmol, 10% yield). ^{1}H NMR (600 MHz, DMSO) δ 11.24 (s, 1H), 8.32 ($CHCl_3$), 8.24 – 8.19 (m, 2H), 8.11 (t, J = 5.6 Hz, 1H), 7.89 – 7.84 (m, 2H), 7.60 – 7.55 (m, 2H), 7.35 (d, J = 2.4 Hz, 1H), 7.20 (dd, J = 8.5, 1.6 Hz, 1H), 3.15 (td, J = 7.1, 5.5 Hz, 2H), 2.83z (t, J = 7.0 Hz, 2H). ^{13}C NMR (151 MHz, DMSO) δ 149.1, 146.1, 134.8, 129.3, 127.6, 126.9, 125.4 (q, $J_{C,F}$ = 271.8 Hz), 124.1, 121.3 (q, $J_{C,F}$ = 30.2 Hz), 118.9, 114.5 (q, $J_{C,F}$ = 3.0 Hz), 114.5, 111.4, 108.6 (q, $J_{C,F}$ = 6.0 Hz), 79.2 ($CHCl_3$), 43.3, 25.0. HRMS (ESI) calcd. for $C_{17}H_{14}F_3N_3O_4S$ (M - H)$^-$ 412.0579, found 412.0579 m/z.

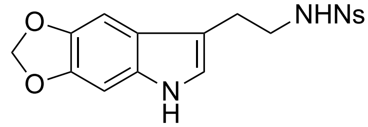

10f

The title compound was prepared following general procedure A and isolated as an orange foam (236 mg, 0.606 mmol, 9% yield). ^{1}H NMR (600 MHz, DMSO) δ 10.55 (s, 1H), 8.32 ($CHCl_3$), 8.29 – 8.24 (m, 2H), 8.04 (t, J = 5.6 Hz, 1H), 7.93 – 7.87 (m, 2H), 6.90 (d, J = 2.3 Hz, 1H), 6.79 (s, 1H), 6.75 (s, 1H), 5.88 (s, 2H), 5.76 (DCM), 3.07 (td, J = 7.2, 5.5 Hz, 2H), 2.70 (t, J = 7.2 Hz, 2H). ^{13}C NMR (151 MHz, DMSO) δ 149.1, 146.1, 143.8, 141.6, 130.8, 127.7, 127.7, 124.2, 121.7, 120.7, 111.0, 100.0, 96.7, 92.1, 79.2 ($CHCl_3$), 54.8 (DCM), 43.3, 25.3. HRMS (ESI) calcd. for $C_{17}H_{15}N_3O_6S$ (M + H)$^+$ 390.0760, found 390.0757 m/z.

5-(hydroxymethyl)-3-methylcyclohex-2-en-1-one (11g):

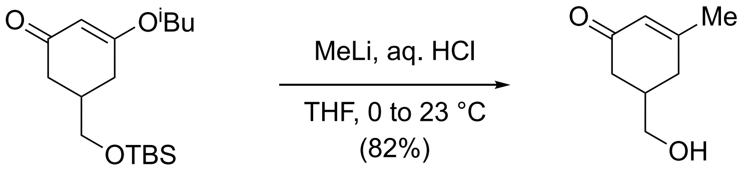

16 **11g**

To a 250 mL round bottom flask was added silyl ether **16** (3.00 g, 9.60 mmol, 1.00 equiv) and THF (40 mL). The reaction mixture was cooled to 0 °C and methyllithium (12.0 mL, 1.60 M, 19.2 mmol, 2.00 equiv.) was added dropwise. The reaction mixture was warmed to 23 °C where it stirred for one hour. The reaction mixture was re-cooled to 0 °C before quenching with 6 N HCl (6.4 mL, 4.00 equiv.). The reaction mixture stirred for four hours at 23 °C before neutralizing with saturated aqueous sodium bicarbonate (60 mL). The aqueous layer was extracted with 3:1 $CHCl_3$/IPA (3 x 80 mL), dried over magnesium sulfate and concentrated *in vacuo.* Flash chromatography of the crude material eluting with a gradient of EtOAc/Hexane (1:1 to 1:0) on a 40g Combiflash ISCO column gave enone **3.22** (1.10g, 9.60 mmol, 82% yield) as a clear oil. Characterization data matched literature preparations.[5]

5-(hydroxymethyl)-3-isopropylcyclohex-2-en-1-one (11h):

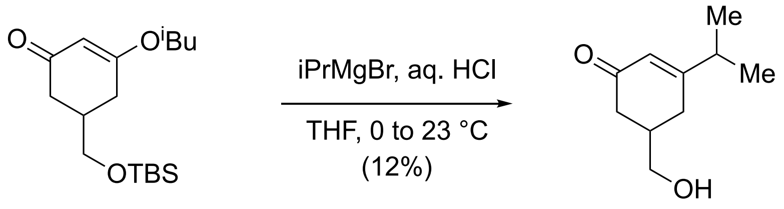

16 **11h**

To a 250 mL round bottom flask was added a solution of isopropylmagnesium bromide in 2-MeTHF (5.52 mL, 2.90 M, 16.0 mmol, 2.50 equiv.) and THF (40 mL). The reaction mixture was cooled to 0 °C silyl ether **16** (3.00 g, 9.60 mmol, 1.00 equiv) in THF (5 mL) was added dropwise. The reaction mixture was warmed to 23 °C where it stirred for three hours. The reaction mixture was re-cooled to 0 °C before quenching with 6 N HCl (10.7 mL, 10.0 equiv.). The reaction mixture stirred for four hours at 23 °C before neutralizing with saturated aqueous sodium bicarbonate (100 mL). The aqueous layer was extracted with EtOAc (3 x 100 mL), dried over sodium sulfate and concentrated *in vacuo.* Flash chromatography of the crude material eluting with a gradient of EtOAc/Hexane (2:3 to 1:0) on a 40g Combiflash ISCO column gave enone **11h** (130 mg, 0.773 mmol, 12% yield) as a clear oil. R_f 0.48 (EtOAc). ^{1}H NMR (600 MHz, $CDCl_3$) δ 5.88 (s, 1H), 3.63 (ddd, *J* = 28.1, 10.6, 5.4 Hz, 2H), 2.49 – 2.39 (m, 3H), 2.31 – 2.15 (m, 3H), 1.84 (s, 1H), 1.10 (dd, *J* = 6.9, 2.4 Hz, 6H). ^{13}C NMR (151 MHz, $CDCl_3$) δ 200.1, 171.1, 123.6, 123.6, 66.2, 40.3, 37.8, 35.9, 30.8, 20.9, 20.5. HRMS (ESI) calcd. for $C_{10}H_{16}O_2$ (M + H)$^+$ 169.1228, found 169.1224 m/z.

5-(hydroxymethyl)-3-(tetrahydro-2*H*-pyran-4-yl)cyclohex-2-en-1-one (11i):

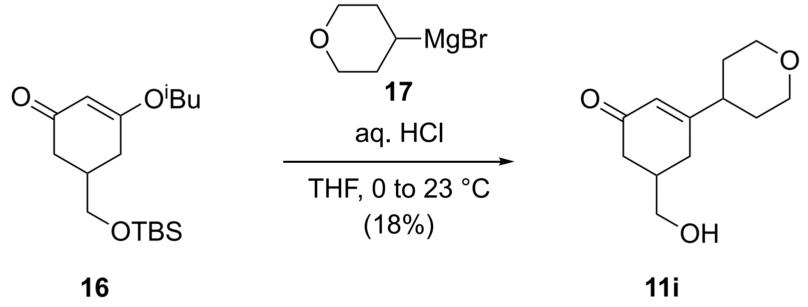

To a 250 mL round bottom flask was added a solution of **17** in THF (25.6 mL, 0.50 molar, 2.50 equiv., 12.8 mmol) THF (15 mL). The reaction mixture was cooled to 0 °C before silyl ether **16** (1.60 g, 5.12 mmol, 1.00 equiv) in THF (3 mL) was added dropwise. The reaction mixture was warmed to 23 °C where it stirred for three hours. The reaction mixture was re-cooled to 0 °C before quenching with 6 N HCl (4.27 mL, 5.0 equiv.). The reaction mixture stirred for four hours at 23 °C before neutralizing with saturated aqueous sodium bicarbonate (50 mL). The aqueous layer was extracted with EtOAc (3 x 50 mL), dried over sodium sulfate and concentrated *in vacuo.* Flash chromatography of the crude material eluting with EtOAc on a 40g Combiflash ISCO column gave enone **11i** (130 mg, 0.773 mmol, 12% yield) as a clear oil. R_f 0.40 (EtOAc). ^{1}H NMR (600 MHz, $CDCl_3$) δ 5.88 (s, 1H), 4.09 – 4.01 (m, 2H), 3.63 (ddd, *J* = 38.9, 10.5, 5.0 Hz, 2H), 3.44 (tt, *J* = 11.6, 2.3 Hz, 2H), 2.45 (ddd, *J* = 23.8, 16.6, 3.2 Hz, 2H), 2.38 – 2.16 (m, 4H), 1.76 (s, 1H), 1.72 – 1.57 (m, 4H). ^{13}C NMR (151 MHz, $CDCl_3$) δ 199.7, 167.3, 124.4, 67.9, 67.9, 66.1, 43.2, 40.3, 37.7, 31.0, 30.9, 30.5. HRMS (ESI) calcd. for $C_{12}H_{18}O_3$ $(M + H)^+$ 211.1334, found 211.1327 m/z.

General Procedure B for Fukuyama-Mitsunobu Coupling:

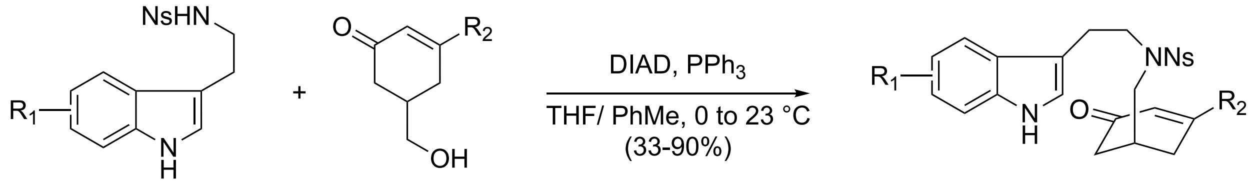

10a-f **11g-j** **18a-i**

To a 100 mL round bottom flask was added nosyl tryptamine **10a-f** (1.00 equiv.), enone **11g-f** (1.50 equiv.), triphenylphosphine (1.60 equiv.), and 1:1 THF/PhMe to make a 0.1 M solution. The reaction mixture was cooled to 0 °C and diisopropyl azodicarboxylate (1.60 equiv.) was added dropwise. The reaction mixture was warmed to 23 °C where it stirred for 16h. The reaction mixture was concentrated *in vacuo* and purified via flash column chromatography to give coupling adducts **18a-i**.

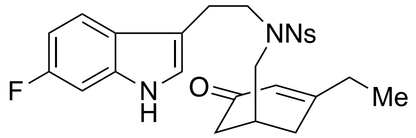

18a

The title compound was prepared following general procedure B. Purified via flash column chromatography eluting with Hexanes/EtOAc (17:3 to 7:13) gave **18a** as an orange foam (277 mg, 0.544 mmol, 58% yield). R_f 0.40 (1:1 Hexane/EtOAc). ^{1}H NMR (600 MHz, $CDCl_3$) δ 8.23 (d, J = 8.2 Hz, 2H), 8.13 (s, 1H), 7.87 (d, J = 8.3 Hz, 2H), 7.38 (dd, J = 8.7, 5.1 Hz, 1H), 6.98 (dd, J = 9.5, 2.2 Hz, 1H), 6.94 (d, J = 2.2 Hz, 1H), 6.89 – 6.84 (m, 1H), 5.89 (s, 1H), 3.45 (ddt, J = 14.7, 13.0, 7.7 Hz, 2H), 3.34 (dd, J = 13.9, 8.4 Hz, 1H), 3.10 – 3.00 (m, 2H), 2.96 (dt, J = 14.8, 7.6 Hz, 1H), 2.45 (dd, J = 16.2, 4.0 Hz, 1H), 2.37 – 2.26 (m, 2H), 2.26 – 2.12 (m, 3H), 2.06 (dd, J = 16.2, 10.9 Hz, 1H), 1.08 (td, J = 7.4, 0.9 Hz, 3H). ^{13}C NMR (151 MHz, $CDCl_3$) δ 198.3, 166.5, 161.0, 159.4, 150.0, 145.1, 136.3 (d, $J_{C,F}$ = 12.1 Hz), 128.2, 124.5, 124.3, 123.6, 122.6 (d, $J_{C,F}$ = 4.5 Hz), 119.2 (d, $J_{C,F}$ = 10.6 Hz), 112.0, 108.6 (d, $J_{C,F}$ = 24.2 Hz), 97.9 (d, $J_{C,F}$ =

25.7 Hz), 53.2, 49.7, 41.2, 33.9, 33.5, 30.9, 25.3, 11.3. HRMS (ESI) calcd. for $C_{25}H_{26}FN_3O_5S$ (M + H)$^+$ 500.1655, found 500.1653 m/z.

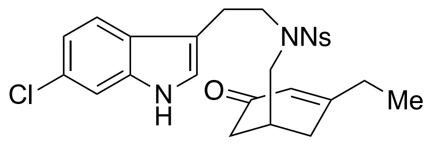

18b

The title compound was prepared following general procedure B. Purified via flash column chromatography eluting with Hexanes/EtOAc (17:3 to 7:13) gave **18b** as an orange foam (331 Mg, 0.641 mmol, 62% yield). R_f 0.42 in (1:1 Hexane/EtOAc). ^{1}H NMR (600 MHz, $CDCl_3$) δ 8.22 (d, *J* = 8.8 Hz, 2H), 8.18 (s, 1H), 7.86 (d, *J* = 8.8 Hz, 2H), 7.37 (d, *J* = 8.4 Hz, 1H), 7.28 (d, *J* = 1.8 Hz, 1H), 7.06 (dd, *J* = 8.4, 1.8 Hz, 1H), 6.96 (d, *J* = 2.3 Hz, 1H), 5.89 (d, *J* = 2.0 Hz, 1H), 3.43 (tdd, *J* = 14.7, 11.3, 7.5 Hz, 2H), 3.33 (dd, *J* = 13.9, 8.4 Hz, 1H), 3.10 – 3.00 (m, 2H), 2.96 (ddd, *J* = 14.7, 8.1, 6.5 Hz, 1H), 2.45 (dd, *J* = 16.7, 3.9 Hz, 1H), 2.37 – 2.26 (m, 2H), 2.26 – 2.12 (m, 3H), 2.11 – 2.01 (m, 1H), 1.08 (t, *J* = 7.4 Hz, 3H). ^{13}C NMR (151 MHz, $CDCl_3$) δ 198.3, 166.6, 149.9, 145.0, 136.7, 128.5, 128.2, 125.6, 124.5, 124.3, 123.1, 120.6, 119.3, 112.1, 111.5, 53.2, 49.7, 41.2, 33.9, 33.5, 30.9, 25.2, 11.3. HRMS (ESI) calcd. for $C_{25}H_{26}ClN_3O_5S$ (M + H)$^+$ 516.1360, found 516.1359 m/z.

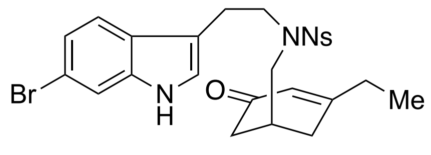

18c

The title compound was prepared following general procedure B. Purified via flash column chromatography eluting with Hexanes/EtOAc (9:1 to 2:3) gave **18c** as an orange foam (220mg, 0.393 mmol, 42% yield). R_f 0.44 in 1:1 (Hexane/EtOAc). ^{1}H NMR (600 MHz, $CDCl_3$) δ 8.23 (d, *J* = 8.8 Hz, 2H), 8.10 (s, 1H), 7.86 (d, *J* = 8.8 Hz, 2H), 7.45 (d, *J* = 1.7 Hz, 1H), 7.34 (d, *J* = 8.4 Hz, 1H), 7.20 (dd, *J* = 8.4, 1.7 Hz, 1H), 6.96 (d, *J* = 2.3 Hz, 1H), 5.89 (s, 1H), 3.49 – 3.37 (m, 2H), 3.33 (dd, *J* = 13.9, 8.4 Hz, 1H), 3.10 – 3.01 (m, 2H), 3.01 – 2.93

(m, 1H), 2.44 (dd, J = 15.7, 3.6 Hz, 1H), 2.35 – 2.11 (m, 5H), 2.06 (dd, J = 16.2, 10.9 Hz, 1H), 1.08 (t, J = 7.4 Hz, 3H). ^{13}C NMR (151 MHz, $CDCl_3$) δ 198.2, 166.5, 150.0, 145.0, 137.1, 128.2, 125.9, 124.5, 124.3, 123.2, 123.0, 119.7, 116.1, 114.5, 112.2, 53.3, 49.8, 41.2, 33.9, 33.5, 30.9, 25.2, 11.3. HRMS (ESI) calcd. for $C_{25}H_{26}BrN_3O_5S$ $(M + H)^+$ 560.0855, found 560.0853 m/z.

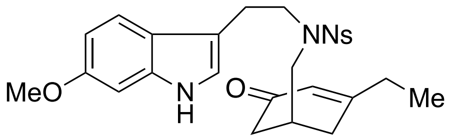

18d

The title compound was prepared following general procedure B. Purified via flash column chromatography eluting with Hexanes/EtOAc (17:3 to 7:13) gave **18d** as an orange foam (294mg, 0.783 mmol, 90% yield). R_f 0.38 in 1:1 (Hexane/EtOAc). ^{1}H NMR (600 MHz, $CDCl_3$) δ 8.13 (d, J = 8.8 Hz, 2H), 7.94 (s, 1H), 7.78 (d, J = 8.8 Hz, 2H), 7.28 (d, J = 8.4 Hz, 1H), 6.80 (d, J = 2.3 Hz, 1H), 6.77 – 6.72 (m, 2H), 5.90 (s, 1H), 3.82 (s, 3H), 3.48 (t, J = 7.3 Hz, 2H), 3.40 (dd, J = 13.9, 8.6 Hz, 1H), 3.11 (dd, J = 13.9, 5.6 Hz, 1H), 3.01 – 2.87 (m, 2H), 2.47 (dd, J = 16.2, 4.0 Hz, 1H), 2.37 (d, J = 14.7 Hz, 2H), 2.31 – 2.16 (m, 3H), 2.08 (dd, J = 16.1, 10.8 Hz, 1H), 1.09 (t, J = 7.4 Hz, 3H). ^{13}C NMR (151 MHz, $CDCl_3$) δ 198.3, 166.6, 156.9, 149.7, 145.2, 137.1, 128.0, 124.5, 124.0, 121.2, 121.0, 119.0, 111.8, 110.0, 94.7, 55.7, 52.5, 49.1, 41.2, 33.6, 33.5, 30.9, 24.9, 11.3. HRMS (ESI) calcd. for $C_{26}H_{29}N_3O_6S$ $(M + H)^+$ 512.1855, found 512.1849 m/z.

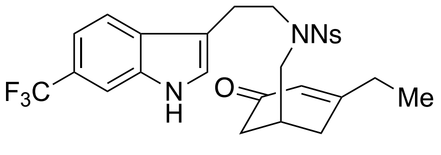

18e

The title compound was prepared following general procedure B. Purified via flash column chromatography eluting with Hexanes/EtOAc (17:3 to 7:13) gave **18e** as an orange foam (161 mg, 0.293 mmol, 51% yield). R_f 0.42 (1:1 Hexane/EtOAc). ^{1}H NMR (600 MHz, $CDCl_3$) δ 8.32 (s, 1H), 8.26 (d, J = 7.2 Hz, 2H), 7.90 (d, J = 7.2 Hz, 2H), 7.63 – 7.56 (m,

2H), 7.35 (d, J = 8.3 Hz, 1H), 7.17 (s, 1H), 5.87 (s, 1H), 3.51 – 3.38 (m, 2H), 3.30 (dd, J = 13.9, 8.3 Hz, 1H), 3.12 (dt, J = 14.7, 7.4 Hz, 1H), 3.04 (dt, J = 13.6, 6.4 Hz, 2H), 2.43 (dd, J = 16.3, 4.0 Hz, 1H), 2.33 – 2.08 (m, 5H), 2.08 – 1.98 (m, 1H), 1.06 (t, J = 7.4 Hz, 3H). ^{13}C NMR (151 MHz, $CDCl_3$) δ 198.2, 166.4, 150.1, 144.9, 135.2, 129.3, 128.3, 124.9 (q, $J_{C,F}$ = 95.1z Hz), 124.6 (q, $J_{C,F}$ = 33.2 Hz), 124.4, 118.9, 116.6 (q, $J_{C,F}$ = 3.0 Hz), 112.4, 109.2 (q, $J_{C,F}$ = 4.5 Hz), 53.7, 50.1, 41.2, 34.0, 33.5, 30.9, 25.5, 11.2. HRMS (ESI) calcd. for $C_{26}H_{26}F_3N_3O_5S$ $(M + H)^+$ 550.1623, found 550.1618 m/z.

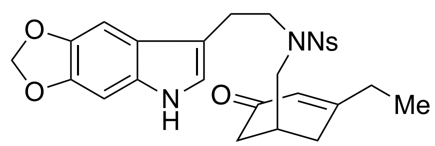

18f

The title compound was prepared following general procedure B. Purified via flash column chromatography eluting with Hexanes/EtOAc (17:3 to 7:13) gave **18f** as an orange foam (224mg, 0.426 mmol, 70% yield). R_f 0.40 (1:1 Hexane/EtOAc). ^{1}H NMR (600 MHz, $CDCl_3$) δ 8.22 (dd, J = 8.6, 1.3 Hz, 2H), 7.88 (s, 1H), 7.85 (d, J = 8.7 Hz, 2H), 6.81 (s, 1H), 6.77 (s, 1H), 6.74 (s, 1H), 5.93 (d, J = 5.2 Hz, 2H), 5.90 (s, 1H), 3.48 – 3.34 (m, 3H), 3.07 (dd, J = 14.0, 5.6 Hz, 1H), 2.94 (dt, J = 14.3, 7.1 Hz, 1H), 2.87 (dt, J = 14.7, 7.6 Hz, 1H), 2.45 (dd, J = 16.1, 4.0 Hz, 1H), 2.36 (td, J = 17.7, 5.9 Hz, 2H), 2.29 – 2.15 (m, 3H), 2.07 (dd, J = 16.2, 10.9 Hz, 1H), 1.10 (t, J = 7.4 Hz, 3H). ^{13}C NMR (151 MHz, $CDCl_3$) δ 198.3, 166.5, 149.9, 145.4, 145.2, 143.2, 131.2, 128.2, 124.6, 124.2, 121.0, 120.9, 112.1, 100.9, 97.0, 92.4, 52.9, 49.3, 41.2, 33.8, 33.5, 31.0, 25.2, 11.3. HRMS (ESI) calcd. for $C_{26}H_{27}N_3O_7S$ $(M + H)^+$ 526.1648, found 526.1639 m/z.

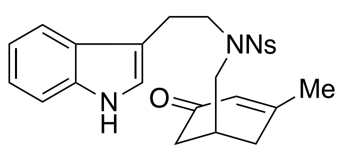

18g

The title compound was prepared following general procedure B. Purified via flash column chromatography eluting with Hexanes/EtOAc (9:1 to 2:3) gave **18g** as an orange foam (1.20 g, 2.57 mmol, 89% yield). R_f 0.36 (1:1 Hexane/EtOAc). ^{1}H NMR (600 MHz, $CDCl_3$) δ 8.14 (d, *J* = 8.5 Hz, 2H), 8.12 (s, 1H), 7.82 – 7.77 (m, 2H), 7.44 (d, *J* = 7.9 Hz, 1H), 7.28 (s, 1H), 7.16 (t, *J* = 7.6 Hz, 1H), 7.08 (t, *J* = 7.5 Hz, 1H), 6.93 (d, *J* = 2.4 Hz, 1H), 5.90 (s, 1H), 3.49 (dh, *J* = 14.7, 7.8 Hz, 2H), 3.41 (dd, *J* = 14.0, 9.0 Hz, 1H), 3.08 (dd, *J* = 13.9, 5.6 Hz, 1H), 2.99 (dtd, *J* = 30.0, 14.6, 7.4 Hz, 2H), 2.45 (dd, *J* = 15.9, 4.1 Hz, 1H), 2.43 – 2.33 (m, 2H), 2.21 (dd, *J* = 18.2, 9.0 Hz, 1H), 2.07 (dd, *J* = 16.0, 10.7 Hz, 1H), 1.96 (s, 3H), 1.26 (d, 1H). ^{13}C NMR (151 MHz, $CDCl_3$) δ 198.0, 161.5, 149.8, 145.2, 136.3, 128.0, 126.9, 126.6, 124.1, 122.5, 122.3, 119.8, 118.4, 111.7, 111.5, 52.5, 49.2, 40.8, 34.5, 33.5, 24.9, 24.6. HRMS (ESI) calcd. for $C_{24}H_{25}N_3O_5S$ $(M + H)^+$ 468.1593, found 468.1583 m/z.

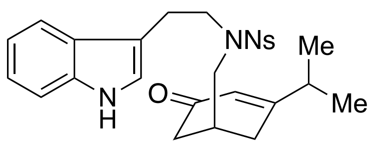

18h

The title compound was prepared following general procedure B. Purified via flash column chromatography eluting with Hexanes/EtOAc (19:1 to 1:1) gave **18h** as an orange foam (198 mg, 0.536 mmol, 75% yield). R_f 0.36 (1:1 Hexane/EtOAc). ^{1}H NMR (600 MHz, $CDCl_3$) δ 8.18 (dd, *J* = 8.8, 1.1 Hz, 2H), 7.99 (s, 1H), 7.86 – 7.81 (m, 2H), 7.45 (d, *J* = 7.9 Hz, 1H), 7.29 (d, *J* = 8.1 Hz, 1H), 7.17 (dd, *J* = 8.2, 6.9 Hz, 1H), 7.09 (dd, *J* = 8.1, 6.9 Hz, 1H), 6.96 (d, *J* = 2.3 Hz, 1H), 5.90 (s, 1H), 3.49 (ddd, *J* = 9.6, 6.9, 2.4 Hz, 2H), 3.37 (dd, *J* = 13.9, 8.6 Hz, 1H), 3.12 (dd, *J* = 13.9, 6.2 Hz, 1H), 3.06 (dt, *J* = 14.4, 7.2 Hz, 1H), 2.97 (dt, *J* = 14.7, 7.5 Hz, 1H), 2.46 (dd, *J* = 16.2, 4.1 Hz, 1H), 2.44 – 2.36 (m, 2H), 2.31 (dp, *J* = 15.1, 5.7, 5.1 Hz, 1H), 2.20 – 2.12 (m, 1H), 2.07 (dd, *J* = 16.2, 11.5 Hz, 1H), 1.09 (dd, *J* = 6.8, 1.7 Hz, 6H). ^{13}C NMR (151 MHz, $CDCl_3$) δ

198.6, 170.4, 149.8, 145.2, 136.3, 128.1, 126.9, 124.2, 123.7, 122.6, 122.3, 119.9, 118.4, 111.9, 111.6, 53.0, 49.4, 41.4, 35.8, 34.0, 31.9, 25.2, 20.9, 20.6. HRMS (ESI) calcd. for $C_{26}H_{29}N_3O_5S$ $(M + H)^+$ 496.1906, found 496.1894 m/z.

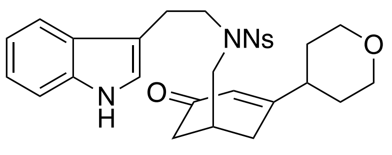

18i

The title compound was prepared following general procedure B. Purified via flash column chromatography eluting with Hexanes/EtOAc (17:3 to 7:13) gave **18i** as an orange foam (251 mg, 0.869 mmol, 54% yield). R_f 0.35 (1:1 Hexane/EtOAc). ^1H NMR (600 MHz, $CDCl_3$) δ 8.18 (dd, J = 8.8, 1.5 Hz, 2H), 8.08 (s, 1H), 7.83 (d, J = 7.3 Hz, 2H), 7.45 (d, J = 7.9 Hz, 1H), 7.29 (d, J = 8.1 Hz, 1H), 7.17 (t, J = 7.6 Hz, 1H), 7.09 (t, J = 7.5 Hz, 1H), 6.97 (s, 1H), 5.88 (s, 1H), 4.03 (dt, J = 11.2, 5.4 Hz, 2H), 3.54 – 3.46 (m, 2H), 3.46 – 3.35 (m, 3H), 3.11 – 3.02 (m, 2H), 2.97 (dt, J = 14.8, 7.6 Hz, 1H), 2.47 (dd, J = 16.2, 4.1 Hz, 1H), 2.39 (dd, J = 17.6, 4.4 Hz, 1H), 2.33 (tt, J = 9.2, 4.7 Hz, 1H), 2.29 – 2.22 (m, 1H), 2.22 – 2.13 (m, 1H), 2.08 (dd, J = 16.2, 11.1 Hz, 1H), 1.65 (s, 1H), 1.60 – 1.48 (m, 2H), 1.27 (dd, J = 8.5, 3.5 Hz, 1H). ^{13}C NMR (151 MHz, $CDCl_3$) δ 198.3, 167.0, 149.9, 145.0, 136.3, 128.1, 126.9, 124.3, 124.2, 122.6, 122.4, 119.9, 118.4, 111.8, 111.6, 67.9, 67.8, 52.9, 49.4, 42.9, 41.4, 33.9, 31.9, 30.8, 30.5, 25.1. HRMS (ESI) calcd. for $C_{28}H_{31}N_3O_6S$ $(M + H)^+$ 538.2012, found 538.1999 m/z.

General Procedure C for Luche Reduction, Acylation, & Friedel Crafts Alkylation:

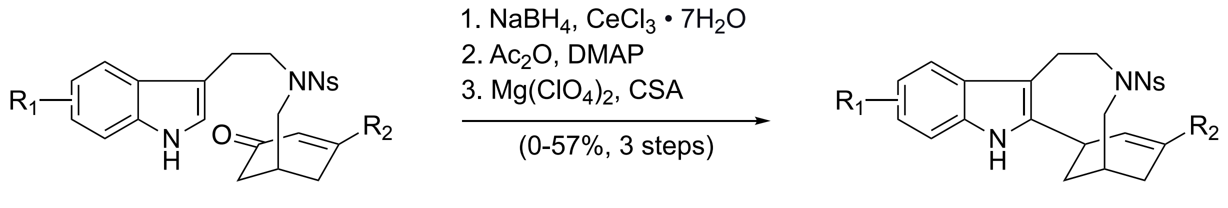

18a-i **8a-i**

To a 50 mL round bottom flask was added nosylamine **18a-i** (1.00 equiv.), 1:1 THF/MeOH to make a 0.1 M solution, and cerium (III) chloride heptahydrate (1.20 equiv). The reaction mixture was cooled to 0 °C and sodium borohydride (1.2 equiv.) was added in a single portion. The reaction mixture was stirred for two hours at 0 °C before quenching with water. The aqueous layer was extracted with EtOAc (3x) and the organics were combined, washed with water (2x), brine, dried over sodium sulfate and concentrated. The crude allylic alcohol was used with no further purification.

To a 25 mL round bottom flask was added crude allylic alcohol (1.0 equiv.), DMAP (2.0 equiv.), and DCM to make a 0.1 M solution. The reaction was cooled to 0 °C and acetic anhydride (1.40 equiv.) was added dropwise. The reaction stirred for one hour before quenching with water. The aqueous layer was extracted with DCM (3x), dried over sodium sulfate and concentrated. The crude allylic acetate was used with no further purification.

To a 500 mL round bottom flask was added magnesium (II) perchlorate (6.0 equiv.), CSA (3.0 equiv.), and DCM to make a 0.033 M solution. The reaction mixture stirred for one hour before the crude allylic acetate was added over the course of an hour as a DCM solution (10 mL/

mmol). Upon completion of the addition, the reaction mixture stirred for 15 more minutes before quenching with saturated aqueous sodium bicarbonate. The organic layer was washed with water (3x), and brine. The organics were dried over sodium sulfate, concentrated *in vacuo* and purified via flash column chromatography to afford macrocycles **8a-i**.

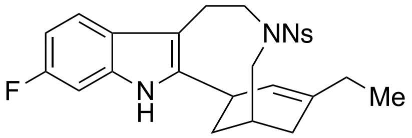

8a

The title compound was prepared following general procedure C. Purified via flash column chromatography eluting with Hexanes/DCM (13:7 to 1:0) gave **8a** as an orange foam (95 mg, 0.196 mmol, 37% yield). R_f 0.60 (DCM). ^{1}H NMR (600 MHz, $CDCl_3$) δ 8.12 (d, *J* = 8.4 Hz, 2H), 7.79 (s, 1H), 7.76 (d, *J* = 8.4 Hz, 2H), 7.18 (dd, *J* = 8.6, 5.1 Hz, 1H), 6.92 (dd, *J* = 9.6, 2.3 Hz, 1H), 6.82 – 6.74 (m, 1H), 5.63 (s, 1H), 3.63 – 3.52 (m, 2H), 3.29 (dd, *J* = 14.9, 6.9 Hz, 1H), 3.18 (ddd, *J* = 15.0, 11.3, 3.1 Hz, 1H), 2.94 (d, *J* = 15.6 Hz, 1H), 2.82 (dd, *J* = 14.9, 10.9 Hz, 1H), 2.76 – 2.69 (m, 1H), 2.69 – 2.62 (m, 1H), 2.49 (d, *J* = 14.6 Hz, 1H), 2.31 (dt, *J* = 17.2, 3.2 Hz, 1H), 2.06 (ddt, *J* = 15.5, 9.8, 5.2 Hz, 3H), 1.74 (d, *J* = 17.4 Hz, 1H), 1.04 (t, *J* = 7.4 Hz, 3H). ^{13}C NMR (151 MHz, $CDCl_3$) δ 160.6, 159.0, 149.9, 143.2, 140.3, 140.2 (d, $J_{C,F}$ = 3.0 Hz), 134.5 (d, $J_{C,F}$ = 12.1 Hz), 128.7, 125.3, 124.0, 119.9, 117.8 (d, $J_{C,F}$ = 9.1 Hz), 108.0 (d, $J_{C,F}$ = 22.7 Hz), 106.8, 97.1 (d, $J_{C,F}$ = 25.7 Hz), 50.3, 50.0, 32.5, 31.8, 31.5, 30.7, 28.7, 22.3, 12.4. HRMS (ESI) calcd. for $C_{25}H_{26}FN_3O_4S$ $(M + H)^+$ 484.1706, found 484.1698 m/z.

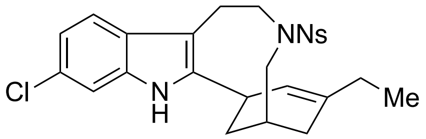

8b

The title compound was prepared following general procedure C. Purified via flash column chromatography eluting with Hexanes/DCM (13:7 to 1:0) gave **8b** as an orange foam (91 mg, 0.182 mmol, 33% yield). R_f 0.62 (DCM). ^{1}H NMR (600 MHz, $CDCl_3$) δ

8.11 (d, J = 8.8 Hz, 2H), 7.80 (s, 1H), 7.75 (d, J = 8.8 Hz, 2H), 7.21 (d, J = 1.8 Hz, 1H), 7.18 (d, J = 8.4 Hz, 1H), 6.98 (dd, J = 8.4, 1.8 Hz, 1H), 5.62 (s, 1H), 3.63 – 3.50 (m, 2H), 3.30 (dd, J = 14.9, 7.0 Hz, 1H), 3.17 (ddd, J = 15.1, 11.6, 3.2 Hz, 1H), 2.98 – 2.89 (m, 1H), 2.80 (dd, J = 14.8, 10.9 Hz, 1H), 2.71 (d, J = 12.3 Hz, 1H), 2.69 – 2.62 (m, 1H), 2.48 (d, J = 14.7 Hz, 1H), 2.31 (dt, J = 17.2, 3.1 Hz, 1H), 2.07 (ddd, J = 17.4, 8.6, 3.9 Hz, 3H), 1.74 (d, J = 17.4 Hz, 1H), 1.04 (t, J = 7.5 Hz, 3H). ^{13}C NMR (151 MHz, $CDCl_3$) δ 149.9, 143.1, 140.8, 140.5, 134.9, 128.7, 127.5, 127.4, 124.0, 120.1, 119.8, 118.0, 110.5, 107.0, 50.3, 50.0, 32.5, 31.8, 31.5, 30.7, 28.8, 22.2, 12.4. HRMS (ESI) calcd. for $C_{25}H_{26}ClN_3O_4S$ $(M + H)^+$ 500.1411, found 500.1409 m/z.

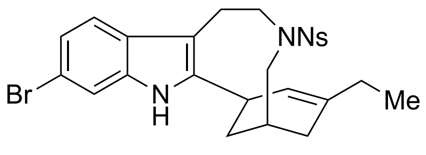

8c

The title compound was prepared following general procedure C. Purified via flash column chromatography eluting with Hexanes/DCM (13:7 to 1:0) gave **8c** as an orange foam (70 mg, 0.130 mmol, 39% yield). R_f 0.62 (DCM). ^{1}H NMR (600 MHz, $CDCl_3$) δ 8.12 (d, J = 8.7 Hz, 2H), 7.79 (s, 1H), 7.75 (d, J = 8.8 Hz, 2H), 7.37 (d, J = 1.6 Hz, 1H), 7.16 – 7.09 (m, 2H), 5.63 (s, 1H), 3.64 – 3.50 (m, 2H), 3.29 (dd, J = 14.9, 7.0 Hz, 1H), 3.17 (ddd, J = 15.0, 11.5, 3.1 Hz, 1H), 2.93 (d, J = 15.6 Hz, 1H), 2.79 (dd, J = 14.8, 10.9 Hz, 1H), 2.71 (d, J = 12.3 Hz, 1H), 2.69 – 2.64 (m, 1H), 2.49 (d, J = 14.7 Hz, 1H), 2.31 (dt, J = 17.2, 3.0 Hz, 1H), 2.13 – 1.99 (m, 3H), 1.74 (d, J = 17.4 Hz, 1H), 1.04 (t, J = 7.4 Hz, 3H). ^{13}C NMR (151 MHz, $CDCl_3$) δ 149.9, 143.1, 140.7, 140.5, 135.3, 128.7, 127.7, 124.0, 122.8, 119.8, 118.4, 115.0, 113.5, 107.1, 50.3, 50.0, 32.5, 31.8, 31.5, 30.7, 28.8, 22.2, 12.4. HRMS (ESI) calcd. for $C_{25}H_{26}BrN_3O_4S$ $(M + H)^+$ 544.0905, found 544.0896 m/z.

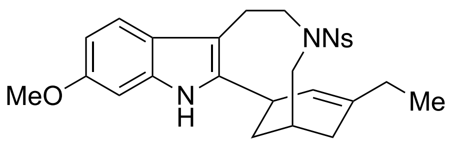

8d

The title compound was prepared following general procedure C. Purified via flash column chromatography eluting with Hexanes/DCM (13:7 to 1:0) gave **8d** as an orange foam (75 mg, 0.151 mmol, 22% yield). R_f 0.22 (DCM). ^{1}H NMR (600 MHz, $CDCl_3$) δ 8.02 (d, *J* = 8.4 Hz, 2H), 7.69 (d, *J* = 8.5 Hz, 2H), 7.62 (s, 1H), 7.10 (d, *J* = 8.6 Hz, 1H), 6.73 (d, *J* = 2.2 Hz, 1H), 6.65 (dd, *J* = 8.6, 2.2 Hz, 1H), 5.64 (s, 1H), 3.82 (s, 3H), 3.57 (d, *J* = 8.9 Hz, 1H), 3.55 – 3.46 (m, 1H), 3.39 (dd, *J* = 14.9, 6.9 Hz, 1H), 3.16 (ddd, *J* = 14.9, 11.4, 3.1 Hz, 1H), 2.92 (dt, *J* = 15.6, 3.8 Hz, 1H), 2.84 (dd, *J* = 14.8, 10.9 Hz, 1H), 2.77 (dt, *J* = 12.5, 3.8 Hz, 1H), 2.71 – 2.62 (m, 1H), 2.44 (d, *J* = 14.6 Hz, 1H), 2.34 – 2.26 (m, 1H), 2.13 – 1.98 (m, 3H), 1.74 (d, *J* = 17.3 Hz, 1H), 1.04 (t, *J* = 7.5 Hz, 3H). ^{13}C NMR (151 MHz, $CDCl_3$) δ 156.4, 149.7, 143.4, 140.1, 138.5, 135.3, 128.6, 123.8, 123.0, 120.2, 117.8, 109.0, 106.7, 94.3, 55.7, 50.3, 49.9, 32.5, 31.8, 31.5, 30.7, 22.4, 12.4. HRMS (ESI) calcd. for $C_{26}H_{29}N_3O_5S$ $(M + H)^+$ 496.1906, found 496.1899 m/z.

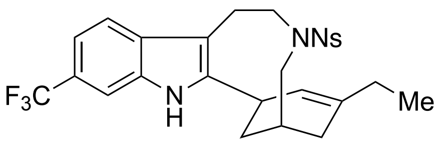

8e

The title compound was prepared following general procedure C. Purified via flash column chromatography eluting with Hexanes/DCM (3:7 to 1:0) gave **8e** as an orange foam (32 mg, 0.060 mmol, 20% yield). R_f 0.60 (DCM). ^{1}H NMR (600 MHz, $CDCl_3$) δ 8.17 (d, *J* = 8.4 Hz, 2H), 8.04 (s, 1H), 7.79 (d, *J* = 8.6 Hz, 2H), 7.54 (s, 1H), 7.41 (d, *J* = 8.3 Hz, 1H), 7.28 (d, *J* = 8.2 Hz, 1H), 5.64 (s, 1H), 3.72 – 3.59 (m, 2H), 3.28 – 3.18 (m, 2H), 3.01 (d, *J* = 15.7 Hz, 1H), 2.80 (dd, *J* = 14.8, 11.0 Hz, 1H), 2.74 – 2.62 (m, 2H), 2.55 (d, *J* = 14.7 Hz, 1H), 2.38 – 2.27 (m, 1H), 2.14 – 2.00 (m, 3H), 1.75 (d, *J* = 17.4 Hz, 1H), 1.04 (t, *J* = 7.5 Hz, 3H). ^{13}C NMR (151 MHz, $CDCl_3$) δ 150.0, 143.0, 143.0, 140.8, 133.5, 131.2, 128.7, 125.3 (q, $J_{C,F}$ = 135.9 Hz),

124.1, 123.8 (q, $J_{C,F}$ = 33.2 Hz), 119.6, 117.4, 116.3 (q, $J_{C,F}$ = 4.5 Hz), 108.0 (q, $J_{C,F}$ = 4.5 Hz), 107.4, 50.4, 50.2, 32.6, 31.7, 31.5, 30.7, 28.6, 22.3, 12.4. HRMS (ESI) calcd. for $C_{26}H_{26}F_3N_3O_4S$ $(M + H)^+$ 534.1674, found 534.1663 m/z.

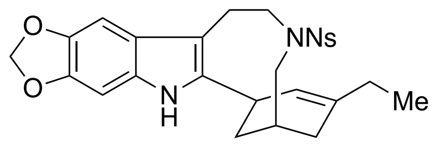

8f

The title compound was prepared following general procedure C. Purified via flash column chromatography eluting with DCM/EtOAc (1:0 to 19:1) gave **8f** as an orange foam (37 mg, 0.073 mmol, 18% yield). R_f 0.42 (DCM). ^1H NMR (600 MHz, $CDCl_3$) δ 8.07 (d, *J* = 8.4 Hz, 2H), 7.71 (d, *J* = 8.5 Hz, 2H), 7.61 (s, 1H), 6.71 (s, 1H), 6.60 (s, 1H), 5.89 (d, *J* = 5.5 Hz, 2H), 5.63 (s, 1H), 3.55 (d, *J* = 9.1 Hz, 1H), 3.48 – 3.32 (m, 2H), 3.12 (ddd, *J* = 15.2, 11.6, 3.2 Hz, 1H), 2.81 (dd, *J* = 14.9, 10.6 Hz, 2H), 2.73 (d, *J* = 12.4 Hz, 1H), 2.66 (s, 1H), 2.42 (d, *J* = 14.6 Hz, 1H), 2.34 – 2.25 (m, 1H), 2.12 – 1.99 (m, 3H), 1.74 (d, *J* = 17.3 Hz, 1H), 1.04 (t, *J* = 7.2 Hz, 3H). ^{13}C NMR (151 MHz, $CDCl_3$) δ 149.8, 144.5, 143.2, 142.8, 140.0, 138.8, 129.1, 128.8, 123.9, 122.5, 120.3, 107.0, 100.8, 96.1, 91.8, 50.0, 49.7, 32.7, 31.9, 31.5, 30.6, 22.3, 12.4. HRMS (ESI) calcd. for $C_{26}H_{27}N_3O_6S$ $(M + H)^+$ 510.1699, found 510.1696 m/z.

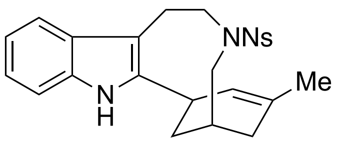

8g

The title compound was prepared following general procedure C. Purified via flash column chromatography eluting with DCM gave **8g** as an orange foam (482 mg, 1.07 mmol, 57% yield). R_f 0.40 (DCM). ^1H NMR (600 MHz, $CDCl_3$) δ 8.01 (d, *J* = 8.3 Hz, 2H), 7.75 (s, 1H), 7.68 (d, *J* = 8.3 Hz, 2H), 7.22 (dd, *J* = 16.3, 8.0 Hz, 2H), 7.07 (t, *J* = 7.5 Hz, 1H), 6.98 (t, *J* = 7.4 Hz, 1H), 5.68 (s, 1H), 3.66 – 3.59 (m, 1H), 3.59 – 3.52 (m, 1H), 3.37 (dd, *J* = 14.9, 6.7 Hz, 1H), 3.25 – 3.16 (m, 1H), 3.00 (dt, *J* = 15.6, 4.0 Hz, 1H), 2.86 (dd, *J* = 14.8, 11.1 Hz, 1H), 2.77 (dt, *J* = 12.7, 3.7 Hz,

1H), 2.67 (ddd, *J* = 8.8, 4.5, 2.3 Hz, 1H), 2.50 (d, *J* = 14.6 Hz, 1H), 2.32 (dt, *J* = 17.8, 4.6 Hz, 1H), 2.05 (ddd, *J* = 14.3, 8.9, 4.7 Hz, 1H), 1.78 (s, 3H), 1.70 (d, *J* = 17.7 Hz, 1H). ^{13}C NMR (151 MHz, $CDCl_3$) δ 149.7, 143.3, 139.6, 135.0, 134.6, 128.6, 128.5, 123.9, 121.8, 121.5, 119.5, 117.1, 110.5, 107.0, 50.5, 49.9, 33.4, 32.6, 30.9, 28.2, 24.9, 22.6. HRMS (ESI) calcd. for $C_{24}H_{25}N_3O_4S$ $(M + H)^+$ 452.1644, found 452.1633 m/z.

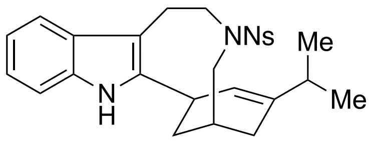

8h

The title compound was prepared following general procedure C. Purified via flash column chromatography eluting with Hexanes/DCM (3:7 to 1:0) gave **8h** as an orange foam (37 mg, 0.077 mmol, 19% yield). R_f 0.50 (DCM). ^{1}H NMR (600 MHz, $CDCl_3$) δ 7.84 (d, *J* = 8.4 Hz, 2H), 7.60 (s, 1H), 7.52 (d, *J* = 8.5 Hz, 2H), 7.09 (t, *J* = 7.0 Hz, 2H), 6.95 (d, *J* = 7.4 Hz, 1H), 6.85 (t, *J* = 7.5 Hz, 1H), 5.52 (s, 1H), 3.46 (dd, *J* = 8.7, 4.3 Hz, 1H), 3.38 – 3.25 (m, 2H), 3.06 (ddd, *J* = 15.4, 11.9, 3.4 Hz, 1H), 2.79 (dd, *J* = 15.4, 3.6 Hz, 1H), 2.74 – 2.63 (m, 2H), 2.59 – 2.50 (m, 1H), 2.26 (d, *J* = 14.6 Hz, 1H), 2.21 – 2.09 (m, 2H), 1.97 (ddd, *J* = 14.4, 9.6, 4.7 Hz, 1H), 1.68 (d, *J* = 17.1 Hz, 1H), 0.92 (d, *J* = 6.8 Hz, 6H). ^{13}C NMR (151 MHz, $CDCl_3$) δ 149.7, 144.0, 143.2, 140.1, 134.5, 128.7, 128.5, 123.8, 121.8, 119.5, 119.3, 117.1, 110.5, 106.7, 49.9, 49.8, 36.0, 32.6, 30.3, 29.5, 22.0, 21.4, 20.9. HRMS (ESI) calcd. for $C_{26}H_{29}N_3O_4S$ $(M + H)^+$ 480.1957, found 480.1948 m/z.

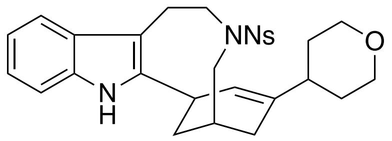

8i

The title compound was prepared following general procedure C. Purified via flash column chromatography eluting with DCM/EtOAc (1:0 to 19:1) gave 8i as an orange foam (75 mg, 0.144 mmol, 31% yield). **8i** contained an inseperable 10 mol% impurity which was purged in

downstream chemistry. R_f 0.20 (DCM). ^{1}H NMR (600 MHz, $CDCl_3$) δ 7.95 (d, *J* = 8.4 Hz, 2H), 7.74 (s, 1H), 7.63 (d, *J* = 8.4 Hz, 2H), 7.20 (t, *J* = 7.2 Hz, 2H), 7.06 (t, *J* = 7.4 Hz, 1H), 6.97 (t, *J* = 7.4 Hz, 1H), 5.66 (s, 1H), 4.05 – 3.97 (m, 2H), 3.62 – 3.58 (m, 1H), 3.49 – 3.36 (m, 4H), 3.14 (ddd, *J* = 15.4, 11.7, 3.3 Hz, 1H), 2.94 – 2.87 (m, 1H), 2.79 (dd, *J* = 14.7, 10.6 Hz, 2H), 2.72 – 2.63 (m, 1H), 2.39 (d, *J* = 14.7 Hz, 1H), 2.27 (dp, *J* = 17.2, 2.7 Hz, 1H), 2.18 – 2.05 (m, 2H), 1.82 (d, *J* = 17.1 Hz, 1H), 1.65 – 1.52 (m, 5H). ^{13}C NMR (151 MHz, $CDCl_3$) δ 149.7, 143.1, 141.6, 139.8, 134.5, 128.6, 128.5, 123.8, 121.9, 120.7, 119.6, 117.1, 110.5, 106.8, 68.3, 49.9, 49.8, 43.4, 32.6, 31.5, 31.1, 30.3, 29.5, 22.0.

General Procedure D for Hydroboration/Oxidation, Mesylation, & Cyclization:

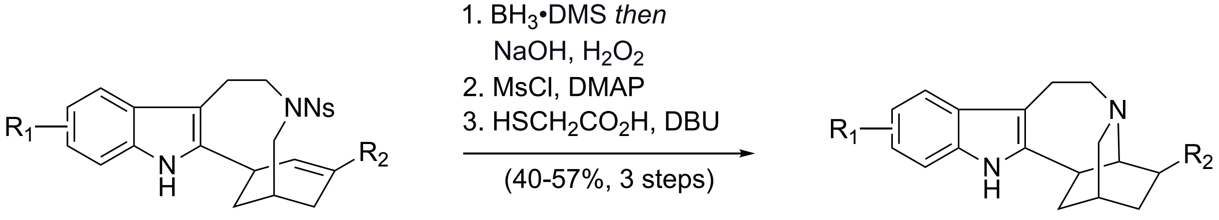

8a-i **7a-i**

To a 50 mL round bottom flask was added nosylamine **8a-i** (1.00 equiv.) and THF to make a 0.1 M solution. To the stirring reaction mixture was added borane dimethyl sulfide (5.00 equiv., 2 M in THF) dropwise. The reaction mixture stirred for two hours before cooling to 0 °C and quenching with water followed by addition of 3 M aqueous sodium hydroxide (30 equiv.), and 30% aqueous hydrogen peroxide (20 equiv.). The reaction mixture stirred for one hour diluting with 1 M sodium hydroxide and extracting with EtOAc (3x). The organics were combined, washed with brine, dried over sodium sulfate and concentrated. The crude secondary alcohol was used with no further purification.

To a 25 mL round bottom flask was added crude secondary alcohol, DMAP (1.0 equiv.) pyridine to make a 0.1 M solution. Methane sulfonyl chloride (4.00 equiv.) was added dropwise. The reaction mixture stirred for one hour at room temperature before diluting with EtOAc. The organic layer was washed with 2 N HCl (4x), sodium bicarbonate, brine, dried over sodium sulfate and concentrated. The crude secondary mesylate was used with no further purification.

To a 25 mL round bottom flask was added secondary mesylate, and 8:1 MeCN/DMF to make a 0.1 M solution. The reaction was cooled to 0 °C and DBU (4.00 equiv.) was added followed by thioglycolic acid (2.00 equiv.) dropwise. The reaction was warmed to room temperature where it

stirred for one hour before a second addition of DBU (4.00 equiv.) and thioglycolic acid (2.00 equiv.). The reaction stirred for one hour before pouring into a biphasic mixture of saturated bicarbonate and EtOAc. The aqueous layer was extracted with EtOAc once more and the organics combined, washed with sodium bicarbonate (2x), brine, dried over sodium sulfate and concentrated. Flash chromatography of the crude material eluting with a gradient of EtOAc/Hexane (1:9 to 4:1) on a 12g Combiflash ISCO column gave the ibogamine analogs **7a-i**.

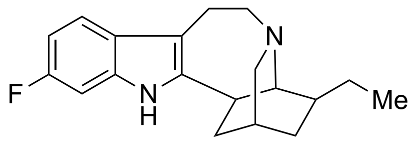

7a

The title compound was prepared following general procedure D and isolated as an clear oil (24 mg, 0.080 mmol, 48% yield). Tertiary amine **7a** had R_f 0.20 in 1:1 EtOAc/Hexane under UV visualization; ^{19}F NMR (471 MHz, $CDCl_3$) δ -121.1. ^{1}H NMR (600 MHz, $CDCl_3$) δ 7.60 (s, 1H), 7.35 (dd, J = 8.7, 5.3 Hz, 1H), 6.93 (dd, J = 9.8, 2.3 Hz, 1H), 6.88 – 6.79 (m, 1H), 3.42 – 3.30 (m, 2H), 3.19 – 3.10 (m, 1H), 3.06 (d, J = 9.3 Hz, 1H), 2.97 (dt, J = 9.3, 3.1 Hz, 1H), 2.95 – 2.89 (m, 1H), 2.85 (s, 1H), 2.67 – 2.58 (m, 1H), 2.09 – 2.00 (m, 1H), 1.85 (s, 1H), 1.80 (td, J = 9.9, 4.9 Hz, 1H), 1.64 (dd, J = 13.2, 3.5 Hz, 1H), 1.54 (dq, J = 13.5, 6.8 Hz, 2H), 1.46 (qd, J = 10.5, 9.9, 4.7 Hz, 1H), 1.24 – 1.18 (m, 1H), 0.90 (t, J = 7.0 Hz, 3H). ^{13}C NMR (151 MHz, $CDCl_3$) δ 160.4, 158.9, 142.2 (d, $J_{C,F}$ = 4.5 Hz), 134.6 (d, $J_{C,F}$ = 12.1 Hz), 126.5, 118.6 (d, $J_{C,F}$ = 10.6 Hz), 109.3, 107.6 (d, $J_{C,F}$ = 24.2 Hz), 96.7 (d, $J_{C,F}$ = 27.2 Hz), 57.7, 54.1, 50.0, 42.1, 41.6, 34.3, 32.3, 27.9, 26.6, 20.9, 12.0. HRMS (ESI) calcd. for $C_{19}H_{23}FN_2$ $(M + H)^+$ 299.1923, found 299.1909 m/z.

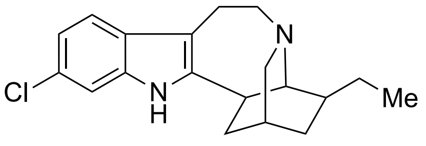

7b

The title compound was prepared following general procedure D and isolated as an clear oil (27 mg, 0.086 mmol, 51% yield). Tertiary amine **7b** had R_f 0.25 in 1:1 EtOAc/Hexane under UV visualization; ^{1}H NMR (600 MHz, $CDCl_3$) δ 7.61 (s, 1H), 7.35 (d, *J* = 8.4 Hz, 1H), 7.22 (d, *J* = 1.8 Hz, 1H), 7.04 (dd, *J* = 8.4, 1.9 Hz, 1H), 3.41 – 3.29 (m, 2H), 3.19 – 3.10 (m, 1H), 3.05 (d, *J* = 9.3 Hz, 1H), 3.01 – 2.95 (m, 1H), 2.91 (ddd, *J* = 11.6, 4.2, 1.9 Hz, 1H), 2.85 (s, 1H), 2.66 – 2.57 (m, 1H), 2.04 (ddt, *J* = 14.2, 11.9, 2.8 Hz, 1H), 1.88 – 1.83 (m, 1H), 1.83 – 1.76 (m, 1H), 1.64 (dq, *J* = 13.2, 3.6 Hz, 1H), 1.61 – 1.50 (m, 3H), 1.50 – 1.41 (m, 1H), 1.24 – 1.18 (m, 1H), 0.90 (t, *J* = 7.2 Hz, 3H). ^{13}C NMR (151 MHz, $CDCl_3$) δ 142.7, 135.1, 128.5, 126.9, 119.8, 118.9, 110.1, 109.6, 57.6, 54.2, 50.1, 42.1, 41.6, 34.3, 32.2, 27.9, 26.6, 20.7, 12.1. HRMS (ESI) calcd. for $C_{19}H_{23}ClN_2$ $(M + H)^+$ 315.1628, found 315.1615 m/z.

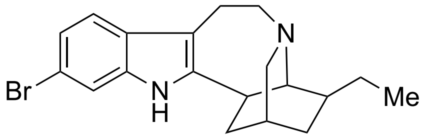

7c

The title compound was prepared following general procedure D and isolated as an clear oil (19 mg, 0.053 mmol, 50% yield). Tertiary amine **7c** had R_f 0.25 in 1:1 EtOAc/Hexane under UV visualization; ^{1}H NMR (600 MHz, $CDCl_3$) δ 7.62 (s, 1H), 7.38 (d, *J* = 1.7 Hz, 1H), 7.31 (d, *J* = 8.4 Hz, 1H), 7.17 (dd, *J* = 8.4, 1.7 Hz, 1H), 3.41 – 3.27 (m, 2H), 3.18 – 3.09 (m, 1H), 3.07 – 3.03 (m, 1H), 3.02 – 2.95 (m, 1H), 2.91 (ddd, *J* = 11.7, 4.2, 1.9 Hz, 1H), 2.85 (s, 1H), 2.67 – 2.58 (m, 1H), 2.04 (ddt, *J* = 14.1, 11.9, 2.8 Hz, 1H), 1.85 (s, 1H), 1.80 (ddd, *J* = 10.7, 7.1, 4.4 Hz, 1H), 1.63 (dq, *J* = 13.2, 3.5 Hz, 2H), 1.60 – 1.51 (m, *J* = 6.6, 5.7 Hz, 2H), 1.51 – 1.40 (m, 1H), 1.25 – 1.17 (m, 1H), 0.90 (t, *J* = 7.2 Hz, 3H). ^{13}C NMR (151 MHz, $CDCl_3$) δ 142.7, 135.6, 128.8, 122.4, 119.3, 114.5, 113.1, 109.6, 57.6, 54.2, 50.1, 42.0, 41.5, 34.2, 32.2,

27.9, 26.6, 20.7, 12.1. HRMS (ESI) calcd. for $C_{19}H_{23}BrN_2$ $(M + H)^+$ 359.1123, found 359.1112 m/z.

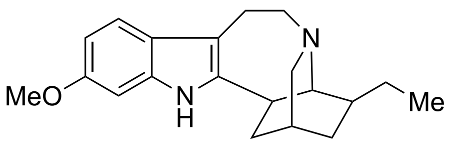

tabernanthine (**3**)

The title compound was prepared following general procedure D and isolated as an clear oil (12 mg, 0.068 mmol, 57% yield). Tabernanthine **3** had R_f 0.20 in 1:1 EtOAc/Hexane under UV visualization; ^1H NMR (600 MHz, $CDCl_3$) δ 7.49 (s, 1H), 7.33 (d, *J* = 8.5 Hz, 1H), 6.77 (s, 1H), 6.75 (d, *J* = 8.6 Hz, 1H), 3.83 (s, 3H), 3.40 – 3.28 (m, 2H), 3.20 – 3.10 (m, 1H), 3.06 (d, *J* = 9.4 Hz, 1H), 2.99 – 2.92 (m, 1H), 2.90 (dd, *J* = 9.8, 2.3 Hz, 1H), 2.85 (s, 1H), 2.65 – 2.60 (m, 1H), 2.02 (t, *J* = 12.3 Hz, 1H), 1.84 (s, 1H), 1.80 (ddd, *J* = 12.7, 9.5, 2.3 Hz, 1H), 1.73 – 1.60 (m, 2H), 1.54 (qd, *J* = 13.8, 13.2, 7.7 Hz, 2H), 1.46 (dq, *J* = 13.8, 7.4, 6.8 Hz, 1H), 1.23 – 1.17 (m, 1H), 0.90 (t, *J* = 7.1 Hz, 3H). ^{13}C NMR (151 MHz, $CDCl_3$) δ 156.0, 140.7, 135.5, 124.4, 118.6, 109.1, 108.6, 94.5, 57.9, 56.0, 54.2, 50.0, 42.1, 41.6, 34.4, 32.3, 27.9, 26.7, 20.9, 12.1. HRMS (ESI) calcd. for $C_{20}H_{26}N_2O$ $(M + H)^+$ 311.2123, found 311.2115 m/z.

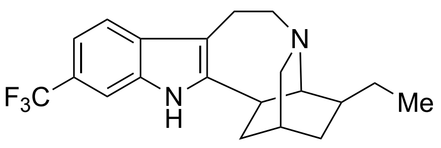

7e

The title compound was prepared following general procedure D and isolated as an clear oil (24 mg, 0.069 mmol, 56% yield). Tertiary amine **7e** had R_f 0.30 in 1:1 EtOAc/Hexane under UV visualization; ^{19}F NMR (471 MHz, $CDCl_3$) δ -60.3. ^1H NMR (600 MHz, $CDCl_3$) δ 7.84 (s, 1H), 7.55 – 7.50 (m, 2H), 7.32 (d, *J* = 8.3 Hz, 1H), 3.43 – 3.34 (m, 2H), 3.15 (td, *J* = 14.8, 3.8 Hz, 1H), 3.10 – 3.05 (m, 1H), 3.00 (dt, *J* = 9.4, 3.1 Hz, 1H), 2.96 (dd, *J* = 11.5, 3.8 Hz, 1H), 2.87 (s, 1H), 2.67 (dd, *J* = 15.4, 3.7 Hz, 1H), 2.12 – 2.04 (m, 1H), 1.87 (p, *J* = 3.0 Hz, 1H), 1.82 (ddt, *J* = 13.0, 10.0, 2.8 Hz, 1H), 1.66 (dq, *J* = 13.3, 3.5 Hz, 1H), 1.62 – 1.53

(m, 2H), 1.52 – 1.42 (m, 1H), 1.22 (ddd, J = 12.7, 5.5, 2.7 Hz, 1H), 0.91 (t, J = 7.0 Hz, 3H). ^{13}C NMR (151 MHz, $CDCl_3$) δ 145.0, 133.6, 132.2, 125.6 (q, $J_{C,F}$ = 4.5 Hz), 123.0 (q, $J_{C,F}$ = 31.7 Hz), 118.3, 116.0 (q, $J_{C,F}$ = 4.5 Hz), 110.1, 107.7 (q, $J_{C,F}$ = 6.0 Hz), 57.4, 54.2, 50.2, 42.1, 41.7, 34.2, 32.2, 28.0, 26.6, 20.7, 12.0. HRMS (ESI) calcd. for $C_{20}H_{23}F_3N_2$ $(M + H)^+$ 349.1891, found 349.1880 m/z.

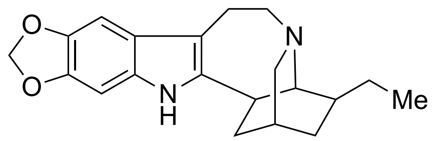

7f

The title compound was prepared following general procedure D and isolated as an clear oil (12 mg, 0.066 mmol, 56% yield). Tertiary amine **7f** had R_f 0.23 in 1:1 EtOAc/Hexane under UV visualization; ^{1}H NMR (600 MHz, $CDCl_3$) δ 7.47 (s, 1H), 6.86 (s, 1H), 6.74 (s, 1H), 5.91 – 5.86 (m, 2H), 3.40 – 3.26 (m, 2H), 3.17 – 3.09 (m, 1H), 3.05 (dd, J = 9.1, 2.2 Hz, 1H), 2.99 – 2.94 (m, 1H), 2.90 – 2.86 (m, 1H), 2.84 (s, 1H), 2.56 – 2.49 (m, 1H), 2.02 (ddd, J = 13.4, 11.3, 2.2 Hz, 1H), 1.84 (s, 1H), 1.82 – 1.75 (m, 1H), 1.67 – 1.60 (m, 2H), 1.58 – 1.51 (m, 2H), 1.48 – 1.42 (m, 1H), 1.23 – 1.16 (m, 1H), 0.89 (t, J = 6.3 Hz, 3H). ^{13}C NMR (151 MHz, $CDCl_3$) δ 144.1, 142.6, 140.8, 129.3, 123.7, 109.5, 100.5, 97.2, 91.6, 57.8, 54.3, 50.0, 42.1, 41.7, 34.5, 32.3, 27.9, 26.7, 21.0, 12.1. HRMS (ESI) calcd. for $C_{20}H_{24}N_2O_2$ $(M + H)^+$ 325.1916, found 325.1910 m/z.

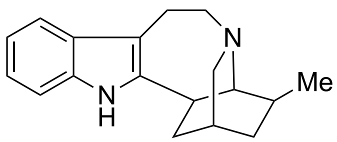

7g

The title compound was prepared following general procedure D and isolated as an clear oil (24 mg, 0.090 mmol, 41% yield). Tertiary amine **7g** had R_f 0.20 in 1:1 EtOAc/Hexane under UV visualization; ^{1}H NMR (600 MHz, $CDCl_3$) δ 7.63 (s, 1H), 7.48 (d, J = 7.0 Hz, 1H), 7.25 (d, J = 7.3 Hz, 1H), 7.10 (dtd, J = 17.9, 7.1, 1.3 Hz, 2H), 3.45 – 3.30 (m, 2H), 3.16 (ddd, J = 13.6, 12.2,

3.9 Hz, 1H), 3.10 – 3.06 (m, 1H), 3.04 – 2.93 (m, 2H), 2.75 – 2.67 (m, 2H), 2.03 (ddt, *J* = 14.0, 11.7, 2.6 Hz, 1H), 1.88 – 1.81 (m, 3H), 1.64 (dq, *J* = 13.2, 3.3 Hz, 1H), 1.24 – 1.16 (m, 1H), 1.12 (d, *J* = 5.9 Hz, 3H). ^{13}C NMR (151 MHz, $CDCl_3$) δ 141.7, 134.8, 129.8, 121.1, 119.3, 118.1, 110.2, 109.3, 60.2, 54.3, 50.0, 41.6, 34.4, 34.0, 33.6, 26.7, 20.8, 20.7. HRMS (ESI) calcd. for $C_{18}H_{22}N_2$ $(M + H)^+$ 267.1861, found 267.1854 m/z.

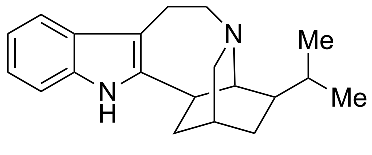

7h

The title compound was prepared following general procedure D and isolated as an clear oil (8 mg, 0.068 mmol, 40% yield). Tertiary amine **7h** had R_f 0.35 in 1:1 EtOAc/Hexane under UV visualization; ^{1}H NMR (600 MHz, $CDCl_3$) δ 7.62 (s, 1H), 7.47 (d, *J* = 7.6 Hz, 1H), 7.25 (d, *J* = 7.1 Hz, 1H), 7.15 – 7.06 (m, 2H), 3.41 – 3.32 (m, 2H), 3.16 (td, *J* = 14.9, 14.3, 3.8 Hz, 1H), 3.11 – 3.05 (m, 2H), 2.98 (dt, *J* = 9.4, 3.1 Hz, 1H), 2.94 – 2.87 (m, 1H), 2.69 (dd, *J* = 15.3, 3.8 Hz, 1H), 2.08 – 1.99 (m, 1H), 1.88 (s, 1H), 1.80 (ddt, *J* = 13.2, 10.4, 3.0 Hz, 1H), 1.73 – 1.61 (m, 2H), 1.36 (ddd, *J* = 12.3, 6.2, 3.0 Hz, 1H), 1.19 (td, *J* = 10.0, 6.2 Hz, 1H), 0.92 (dd, *J* = 25.5, 6.8 Hz, 6H). ^{13}C NMR (151 MHz, $CDCl_3$) δ 142.0, 134.8, 129.8, 121.1, 119.3, 118.1, 110.2, 109.3, 56.0, 54.2, 50.1, 48.0, 41.6, 34.1, 31.7, 31.4, 26.8, 21.4, 20.8, 20.6. HRMS (ESI) calcd. for $C_{20}H_{26}N_2$ $(M + H)^+$ 295.2174, found 295.2164 m/z.

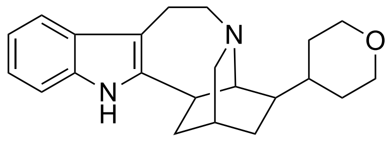

7i

The title compound was prepared following general procedure D and isolated as an clear oil (12 mg, 0.036 mmol, 56% yield). Tertiary amine **7i** had R_f 0.22 in 1:1 EtOAc/Hexane under UV visualization; ^{1}H NMR (600 MHz, $CDCl_3$) δ 7.69 (s, 1H), 7.47 (d, *J* = 7.6 Hz, 1H), 7.25 (d, *J* = 7.3 Hz, 1H), 7.15 – 7.04 (m, 2H), 4.01 (dd, *J* = 11.4, 4.2 Hz, 1H), 3.96 (dd, *J* = 11.5, 4.6 Hz,

1H), 3.46 – 3.33 (m, 4H), 3.17 – 3.07 (m, 2H), 3.05 (s, 1H), 2.97 (dt, *J* = 9.9, 3.2 Hz, 1H), 2.90 (ddd, *J* = 11.6, 4.1, 1.9 Hz, 1H), 2.72 – 2.64 (m, 1H), 2.05 (t, *J* = 12.7 Hz, 1H), 1.90 (s, 1H), 1.84 – 1.74 (m, 2H), 1.74 – 1.60 (m, 3H), 1.40 – 1.30 (m, 2H), 1.30 – 1.15 (m, 2H). ^{13}C NMR (151 MHz, $CDCl_3$) δ 141.7, 134.8, 129.8, 121.2, 119.3, 118.1, 110.3, 109.3, 68.6, 68.2, 54.7, 54.2, 50.0, 46.3, 41.4, 38.4, 34.1, 31.7, 31.1, 30.4, 26.5, 20.8. HRMS (ESI) calcd. for $C_{22}H_{28}N_2O$ (M + H)$^+$ 337.2280, found 337.2273 m/z.

7-ethyl-5,6,6a,7,8,9,10,12-octahydro-13*H*-6,9-methanobenzo[*b*]pyrido[1,2-*g*][1,6] naphthyridin-13-one (20):

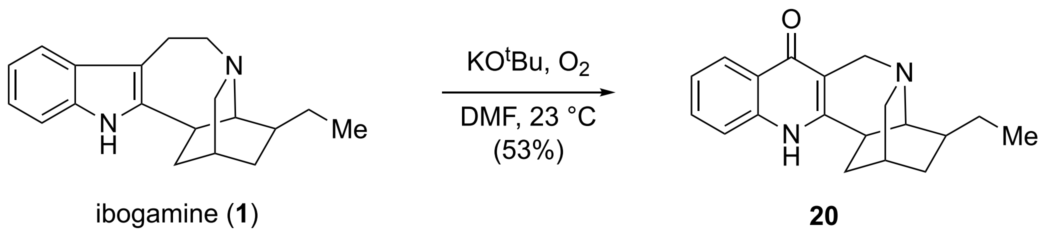

To a 25 mL round bottom flask was added (±) – ibogamine (0.050 g, 0.18 mmol, 1.00 equiv.), DMF (2.0 mL), and potassium tert-butoxide (60 mg, 0.53 mmol, 3.00 equiv.). The stirring solution was sparged with oxygen for two and a half hours. The reaction mixture was diluted with EtOAc and sodium bicarbonate. The aqueous layer was extracted with EtOAc (2 x 20 mL) and the organics combined, washed with water, brine, dried over sodium sulfate and concentrated *in vacuo.* Flash chromatography of the crude material eluting with a gradient of DCM/MeOH (1:0 to 4:1) on a 12g Combiflash ISCO column gave quinolone **20** (28 mg, 0.095 mmol, 53% yield) as a white amorphous solid. R_f 0.22 (9:1 DCM/MeOH). ^{1}H NMR (600 MHz, DMSO) δ 8.04 (dd, *J* = 8.1, 1.5 Hz, 1H), 7.59 (ddd, *J* = 8.4, 6.9, 1.6 Hz, 1H), 7.51 (d, *J* = 8.3 Hz, 1H), 7.24 (ddd, *J* = 8.0, 6.8, 1.1 Hz, 1H), 5.76 (DCM), 3.54 (s, 2H), 3.10 – 2.91 (m, 1H), 2.79 – 2.64 (m, 1H), 2.65 – 2.52 (m, 1H), 2.29 (s, 1H), 1.98 (t, *J* = 12.0 Hz, 1H), 1.82 – 1.71 (m, 1H),

1.62 (s, 2H), 1.50 (ddt, *J* = 20.6, 15.2, 6.4 Hz, 3H), 1.05 – 0.96 (m, 1H), 0.88 (t, *J* = 7.4 Hz, 3H). ^{13}C NMR (151 MHz, DMSO) δ 176.2, 152.2, 139.7, 131.1, 124.5, 123.7, 122.2, 117.7, 110.5, 54.8 (DCM), 54.1, 52.0, 51.5, 37.0, 32.7, 31.4, 31.0, 26.8, 24.5, 11.6. HRMS (ESI) calcd. for $C_{19}H_{22}N_2O$ $(M + H)^+$ 295.1810, found 295.1801 m/z.

7-ethyl-5,6,6a,7,8,9,12,13-octahydro-10*H*-6,9-methanopyrido[1',2':1,2]azepino[4,5-*b*]indol-10-one (21):

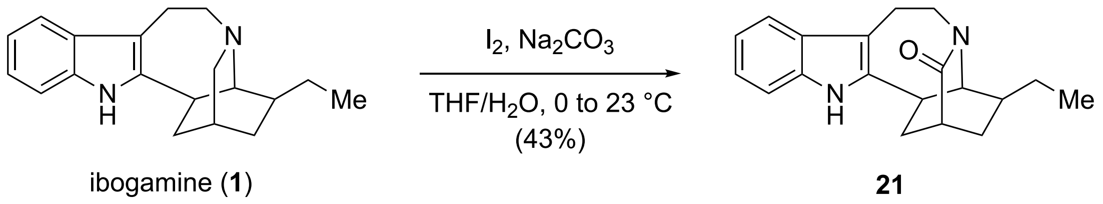

To a 25 mL round bottom flask was added (±) – ibogamine (0.058 g, 0.207 mmol, 1.00 equiv.), THF (2.0 mL), and aqueous sodium carbonate (1.03 mL, 2.0 M, 10.0 equiv.). The reaction mixture was cooled to 0 °C and iodine in THF (1.5 mL) was added slowly. After warming to 23 °C and stirring for 16 hours, the reaction mixture was quenched with sodium thiosulfate and diluted with sodium bicarbonate. The aqueous layer was extracted with EtOAc (3x) and the organics combined, washed with brine, dried over sodium sulfate and concentrated *in vacuo.* Flash chromatography of the crude material eluting with a gradient of Hexane/EtOAc (9:1 to 0:1) on a 12g Combiflash ISCO column gave amide **21** (26 mg, 0.095 mmol, 43% yield) as a white amorphous solid. R_f 0.15 (1:1 Hexane/EtOAc). ^{1}H NMR (600 MHz, DMSO) δ 10.78 (s, 1H), 8.32 (CHCl3), 7.39 (dd, *J* = 7.8, 1.1 Hz, 1H), 7.22 (dt, *J* = 8.0, 0.9 Hz, 1H), 6.99 (ddd, *J* = 8.0, 7.0, 1.2 Hz, 1H), 6.93 (ddd, *J* = 8.0, 7.0, 1.1 Hz, 1H), 4.35 – 4.21 (m, 1H), 4.03 (t, *J* = 1.8 Hz, 1H), 3.37 – 3.33 (m, 1H), 3.13 – 3.06 (m, 2H), 3.01 – 2.93 (m, 1H), 2.41 (h, *J* = 2.1 Hz, 1H), 2.18 (ddd, *J* = 13.4, 10.0, 1.9 Hz, 1H), 1.95 (ddd, *J* = 13.2, 10.2, 3.3 Hz, 1H), 1.80 – 1.72 (m, 1H), 1.66 – 1.59 (m, 1H), 1.46 (dp, *J* = 14.3, 7.3 Hz, 1H), 1.28 (dt, *J* = 13.3, 7.5 Hz, 1H), 1.19 (ddd, *J* = 13.2, 5.7, 2.8 Hz, 1H), 0.92 (t, *J* = 7.4 Hz, 3H). ^{13}C NMR (151 MHz, DMSO) δ 175.4, 139.0, 135.2, 128.2, 120.4, 118.2, 117.6, 110.4, 106.1, 79.2 (CHCl3), 57.2, 44.5, 38.2, 37.1, 33.0, 30.3, 27.4, 21.1, 11.3. HRMS (ESI) calcd. for $C_{19}H_{22}N_2O$ $(M + H)^+$ 295.1810, found 295.1805 m/z.

(13a*S*)-7-ethyl-6,6a,7,8,9,10,12,13-octahydro-13a*H*-6,9-methanopyrido[1',2':1,2] azepino[4,5-*b*]indol-13a-ol (22):

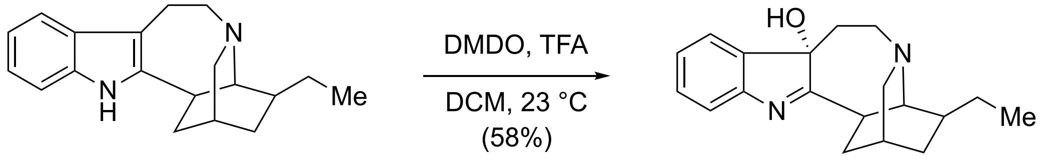

ibogamine (**1**) **22**

To a 25 mL round bottom flask was added (±) – ibogamine (0.059 g, 0.18 mmol, 1.00 equiv.) and DCM (4.0 mL). The reaction mixture was cooled to 0 °C and trifluoroacetic acid (36 mg, 24 μL, 0.32 mmol, 1.50 equiv.) was added followed by dimethyldioxirane (17 mg, 3.9 mL, 60 mM, 1.10 equiv.) dropwise. The reaction stirred for 30 minutes at 0 °C before quenching with sodium bicarbonate and extracting with EtOAc (3x). The organics were combined, washed with brine, dried over sodium sulfate and concentrated *in vacuo.* Flash chromatography of the crude material eluting with a gradient of Hexane/EtOAc (1:0 to 3:2) on a 12g Combiflash ISCO column gave indolenine **22** (28 mg, 0.095 mmol, 53% yield) as a clear oil. R_f 0.55 (1:1 Hexane/EtOAc). ^{1}H NMR (600 MHz, $CDCl_3$) δ 7.33 (d, *J* = 7.4 Hz, 1H), 7.20 (p, *J* = 7.2 Hz, 2H), 6.97 (d, *J* = 7.1 Hz, 1H), 4.93 (s, 1H), 3.60 – 3.54 (m, 1H), 3.48 (ddd, *J* = 15.1, 11.0, 4.3 Hz, 1H), 2.96 – 2.88 (m, 1H), 2.77 (dd, *J* = 8.0, 4.4 Hz, 1H), 2.63 (d, *J* = 9.0 Hz, 1H), 2.43 (d, *J* = 12.0 Hz, 1H), 1.98 (t, *J* = 12.8 Hz, 1H), 1.93 – 1.83 (m, 2H), 1.78 (dd, *J* = 13.8, 3.5 Hz, 1H), 1.73 (s, 1H), 1.66 (tt, *J* = 11.9, 2.6 Hz, 1H), 1.52 (ddq, *J* = 33.0, 13.7, 7.1 Hz, 2H), 1.33 – 1.26 (m, 1H), 1.11 (ddd, *J* = 13.0, 5.5, 2.2 Hz, 1H), 0.98 (t, *J* = 7.4 Hz, 3H). ^{13}C NMR (151 MHz, $CDCl_3$) δ 194.4, 151.7, 142.0, 129.3, 126.0, 121.8, 119.4, 86.7, 53.0, 49.9, 49.0, 43.3, 40.5, 34.7, 31.8, 31.6, 27.4, 27.2, 11.9. HRMS (ESI) calcd. for $C_{19}H_{24}N_2O$ $(M + H)^+$ 297.1967, found 297.1962 m/z.

7-ethyl-5-(3-methylbut-2-en-1-yl)-6,6a,7,8,9,10,12,13-octahydro-5*H*-6,9-methanopyrido [1',2':1,2]azepino[4,5-*b*]indole (23):

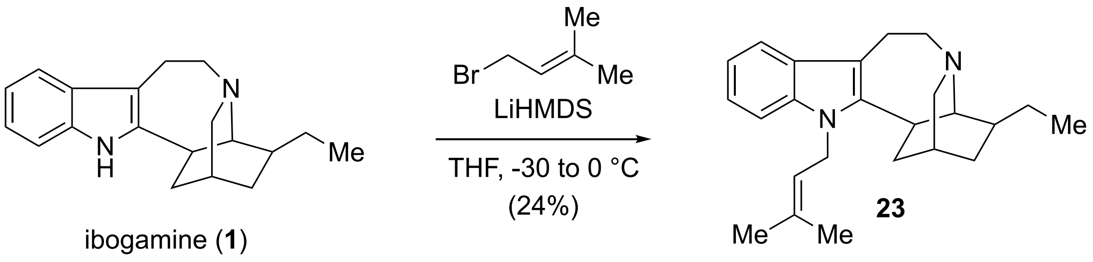

To a flame dried 25 mL round bottom flask was added (±) – ibogamine (0.050 g, 0.180 mmol, 1.00 equiv.) and THF (3.0 mL). Cooled to -20 °C and LiHMDS (0.18 mL, 1.5M, 0.26 mmol, 1.50 equiv.) was added dropwise. The reaction mixture stirred at -20 °C for one hour before prenyl bromide (32 mg, 25 µL, 0.21 mmol, 1.20 equiv.) was added dropwise. The reaction mixture was warmed to 23 °C where it stirred for 1h before quenching with sodium bicarbonate. The aqueous solution was extracted with EtOAc (3x) and the organics combined, washed with brine, dried over sodium sulfate and concentrated *in vacuo.* Flash chromatography of the crude material eluting with a gradient of Hexane/EtOAc (1:0 to 13:7) on a 12g Combiflash ISCO column gave prenylated indole **3.67** (15 mg, 0.043 mmol, 24% yield) as a clear oil. Indole **23** contained 7% of the terminal olefin isomer which came from the commercial prenyl bromide (90% pure). R_f 0.55 (1:1 Hexane/EtOAc). ^{1}H NMR (600 MHz, $CDCl_3$) δ 7.51 (d, *J* = 7.6 Hz, 1H), 7.29 (ddd, *J* = 7.7, 6.5, 2.1 Hz, 1H), 7.19 – 7.14 (m, 2H), 4.42 (dddd, *J* = 7.2, 5.8, 2.9, 1.5 Hz, 1H), 3.37 (ddd, *J* = 15.9, 13.0, 3.3 Hz, 1H), 3.20 (d, *J* = 2.9 Hz, 1H), 3.15 (dd, *J* = 14.8, 8.6 Hz, 1H), 3.07 – 3.01 (m, 2H), 2.72 (ddd, *J* = 9.0, 4.0, 2.7 Hz, 1H), 2.68 (dt, *J* = 9.1, 1.7 Hz, 1H), 2.62 – 2.54 (m, 1H), 2.34 (ddt, *J* = 13.3, 4.6, 2.3 Hz, 1H), 2.18 (ddt, *J* = 13.3, 11.2, 2.1 Hz, 1H), 1.84 – 1.77 (m, 2H), 1.77 – 1.68 (m, 2H), 1.54 – 1.49 (m, 6H), 1.48 (s, 3H), 1.18 – 1.11 (m, 1H), 0.94 (t, *J* = 7.2 Hz, 3H). ^{13}C NMR (151 MHz, $CDCl_3$) δ 194.5, 154.0, 145.4, 134.1, 127.7, 125.2,

121.0, 119.7, 118.3, 63.5, 55.2, 50.7, 49.5, 45.1, 40.5, 34.3, 32.3, 31.1, 30.9, 27.2, 27.1, 25.8, 18.2, 12.0. HRMS (ESI) calcd. for $C_{24}H_{32}N_2$ $(M + H)^+$ 349.2643, found 349.2638 m/z.

^{1}H NMR of (600 MHz, DMSO) of Compound 24

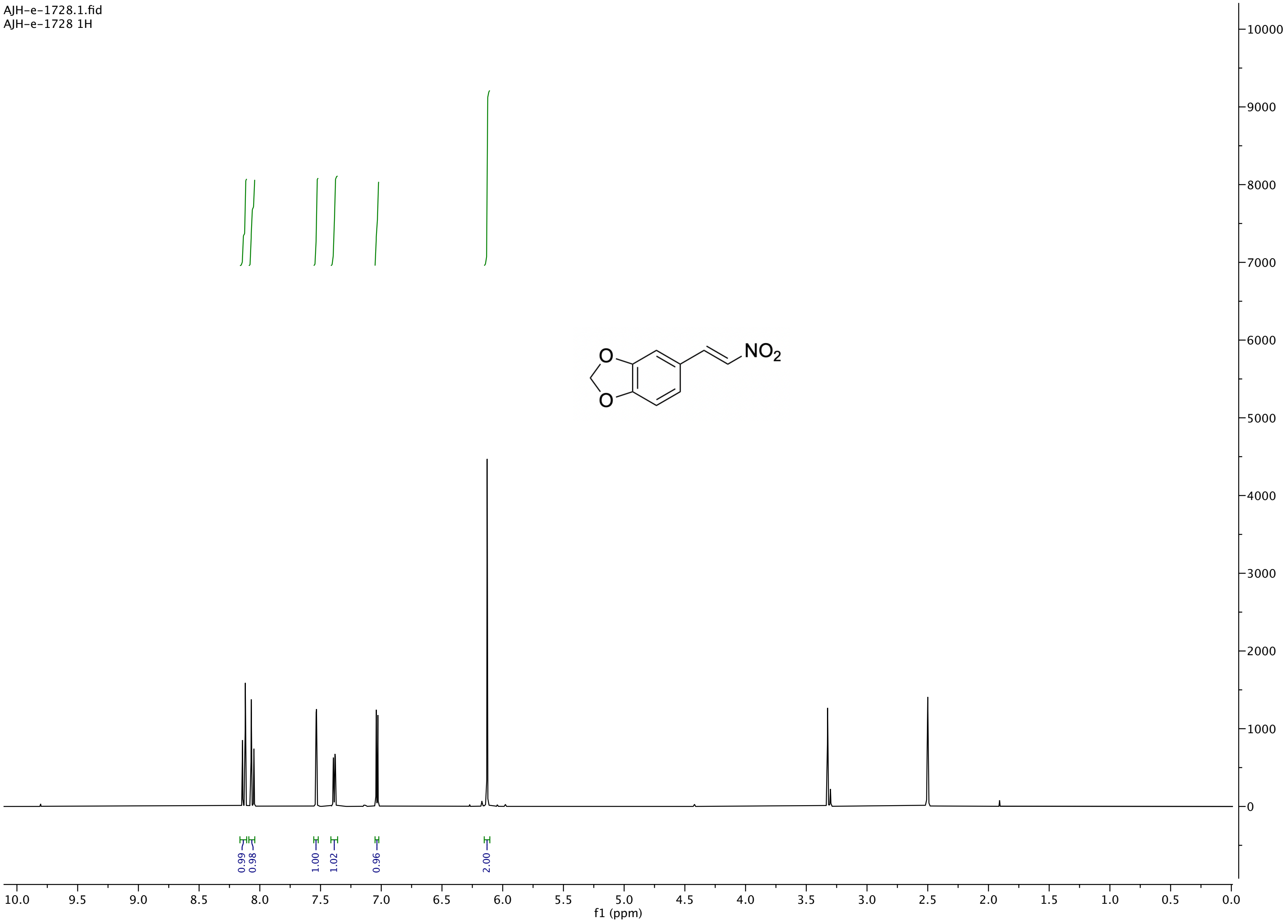

^{13}C NMR of (151 MHz, DMSO) of Compound 24

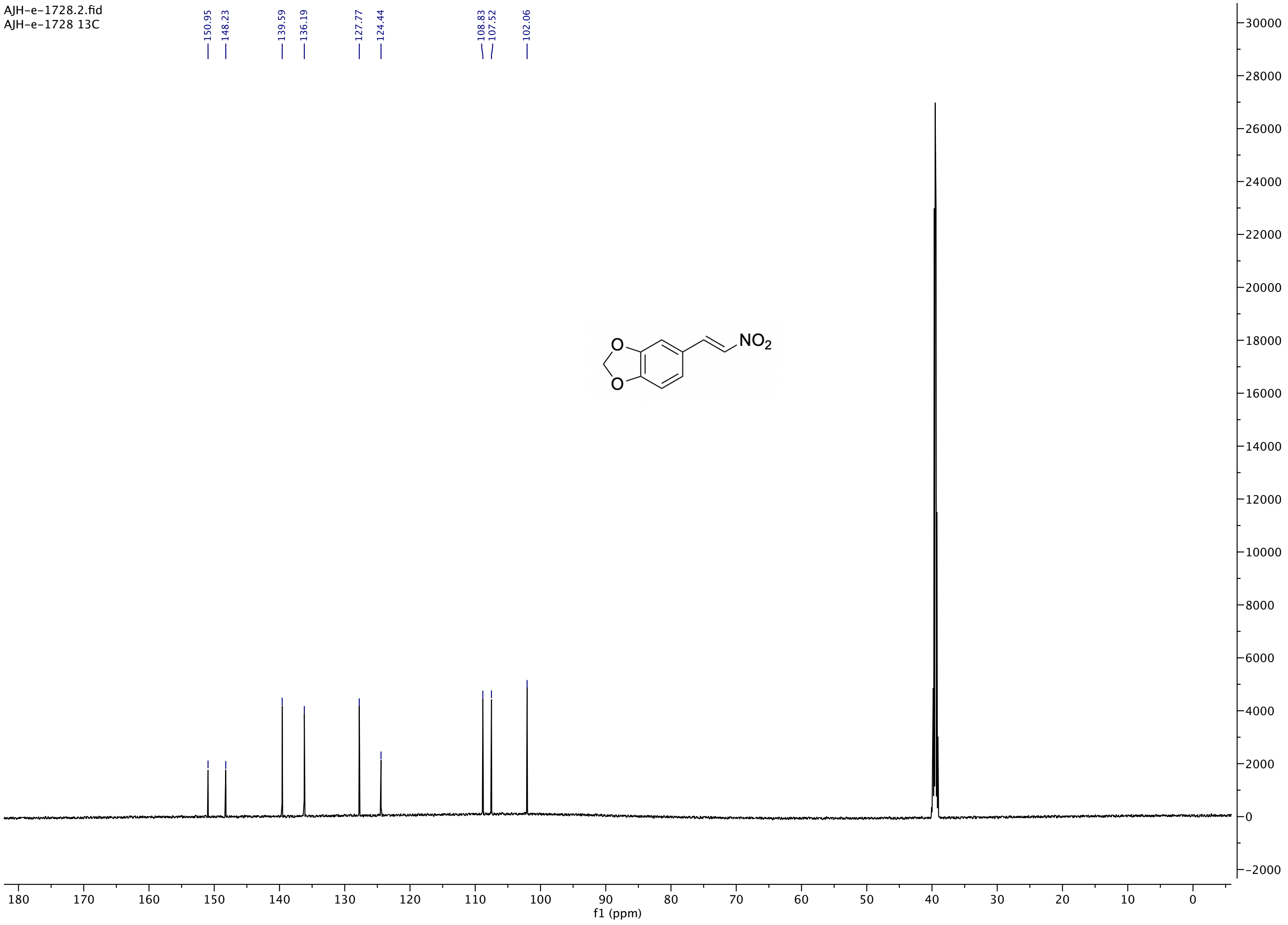

^{1}H NMR (600 MHz, DMSO) of Compound 15

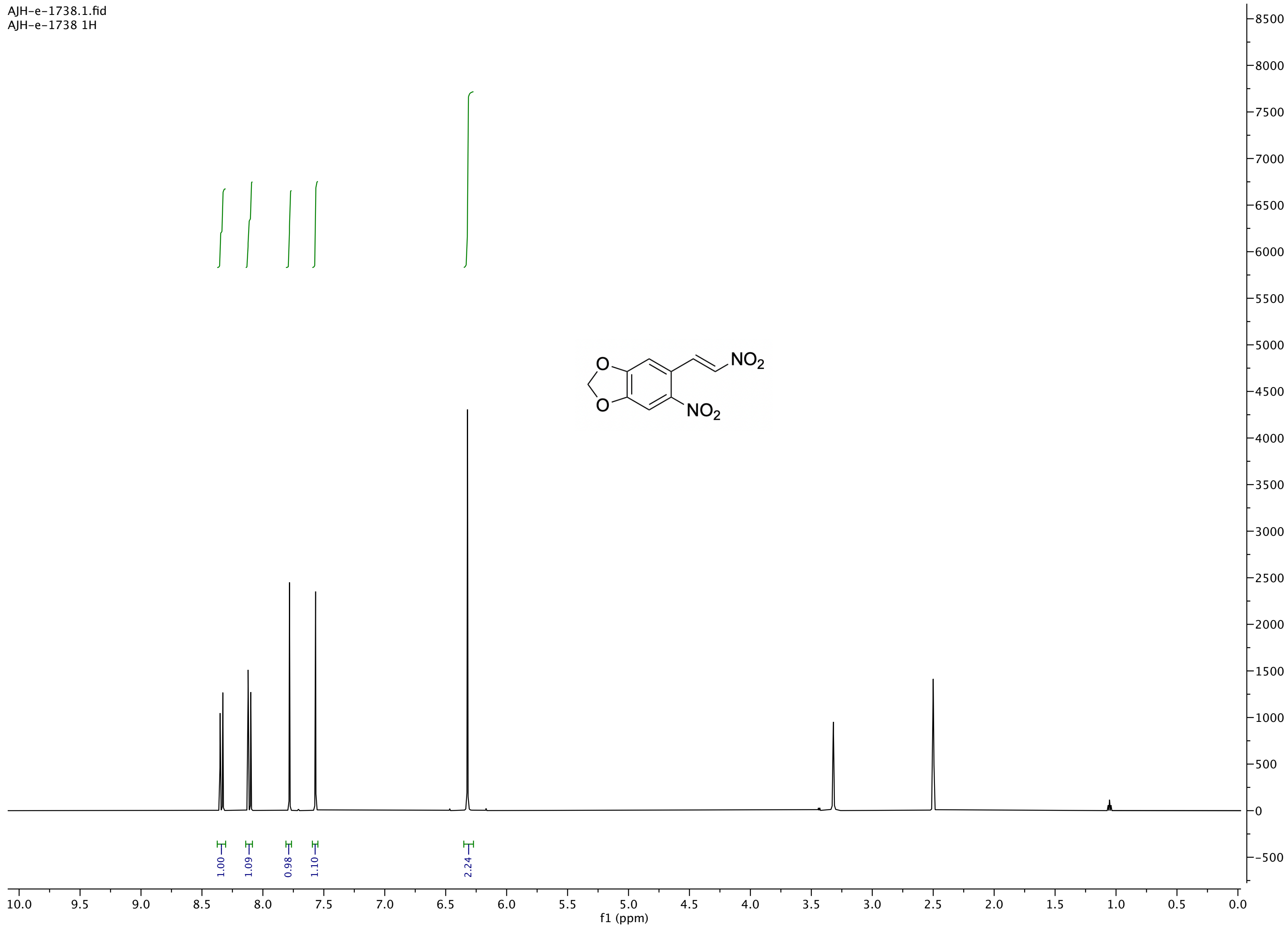

^{13}C NMR of (151 MHz, DMSO) of Compound 15

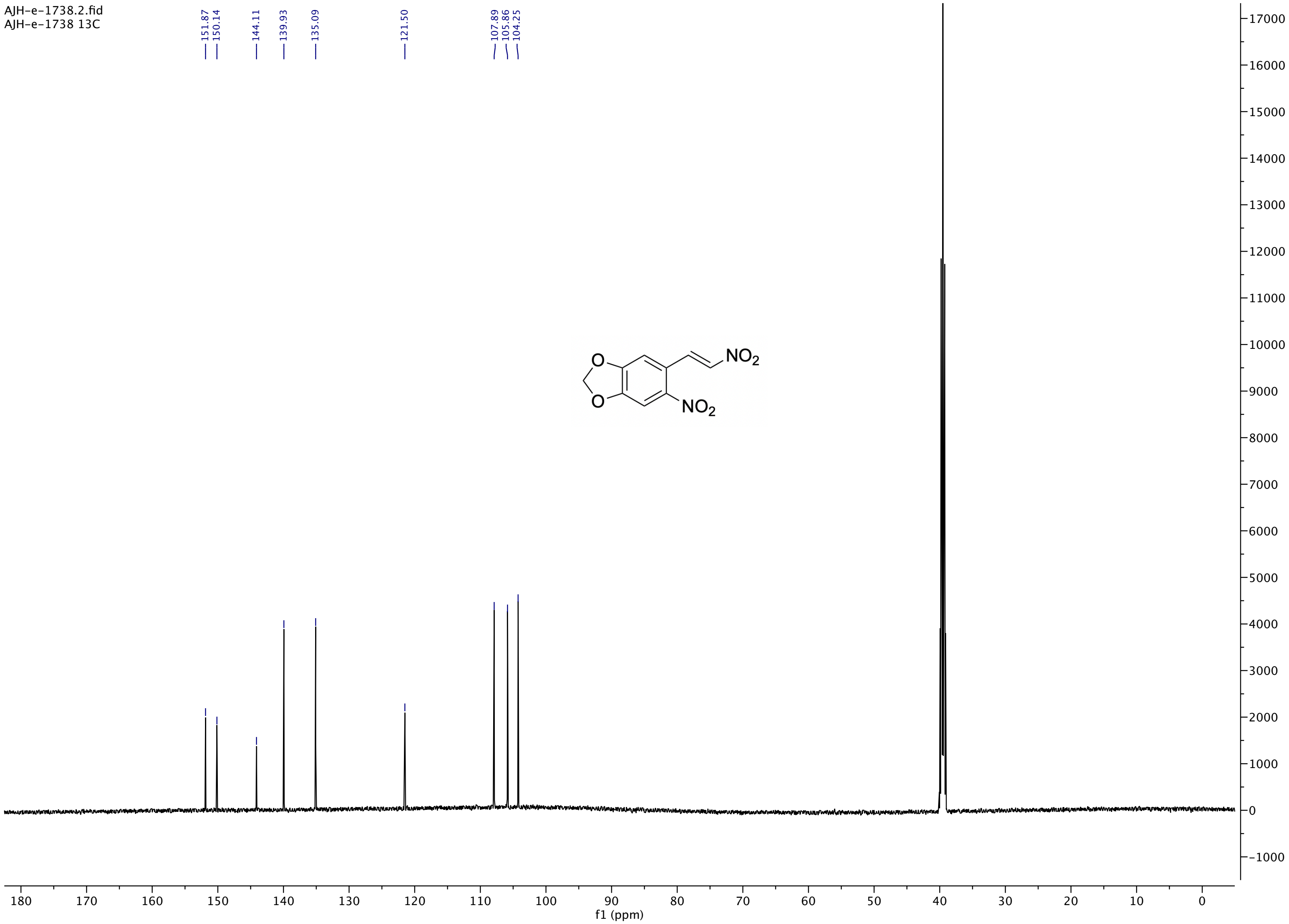

^{1}H NMR (600 MHz, $CDCl_3$) of Compound 12f

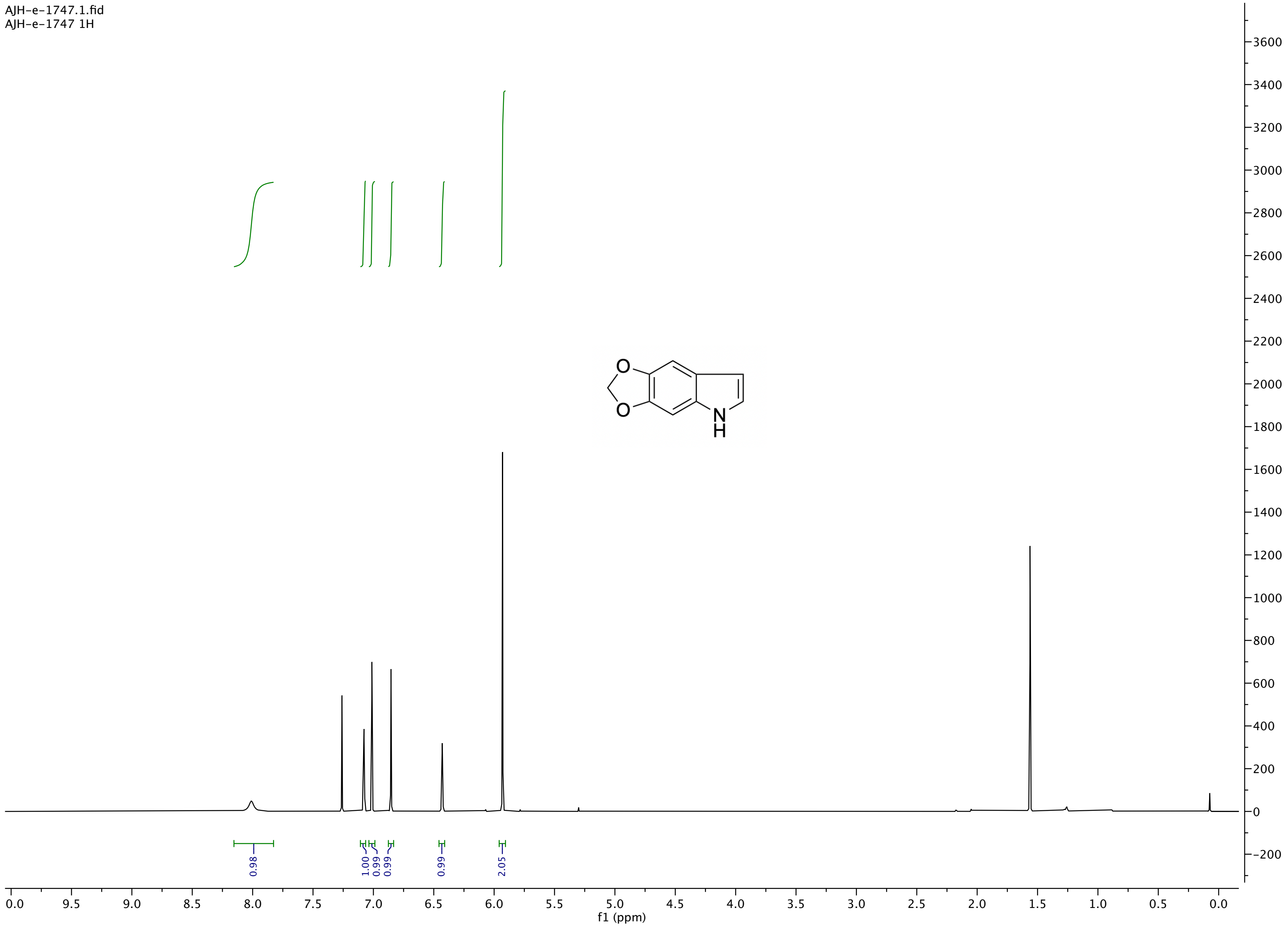

^{13}C NMR of (151 MHz, DMSO) of Compound 12f

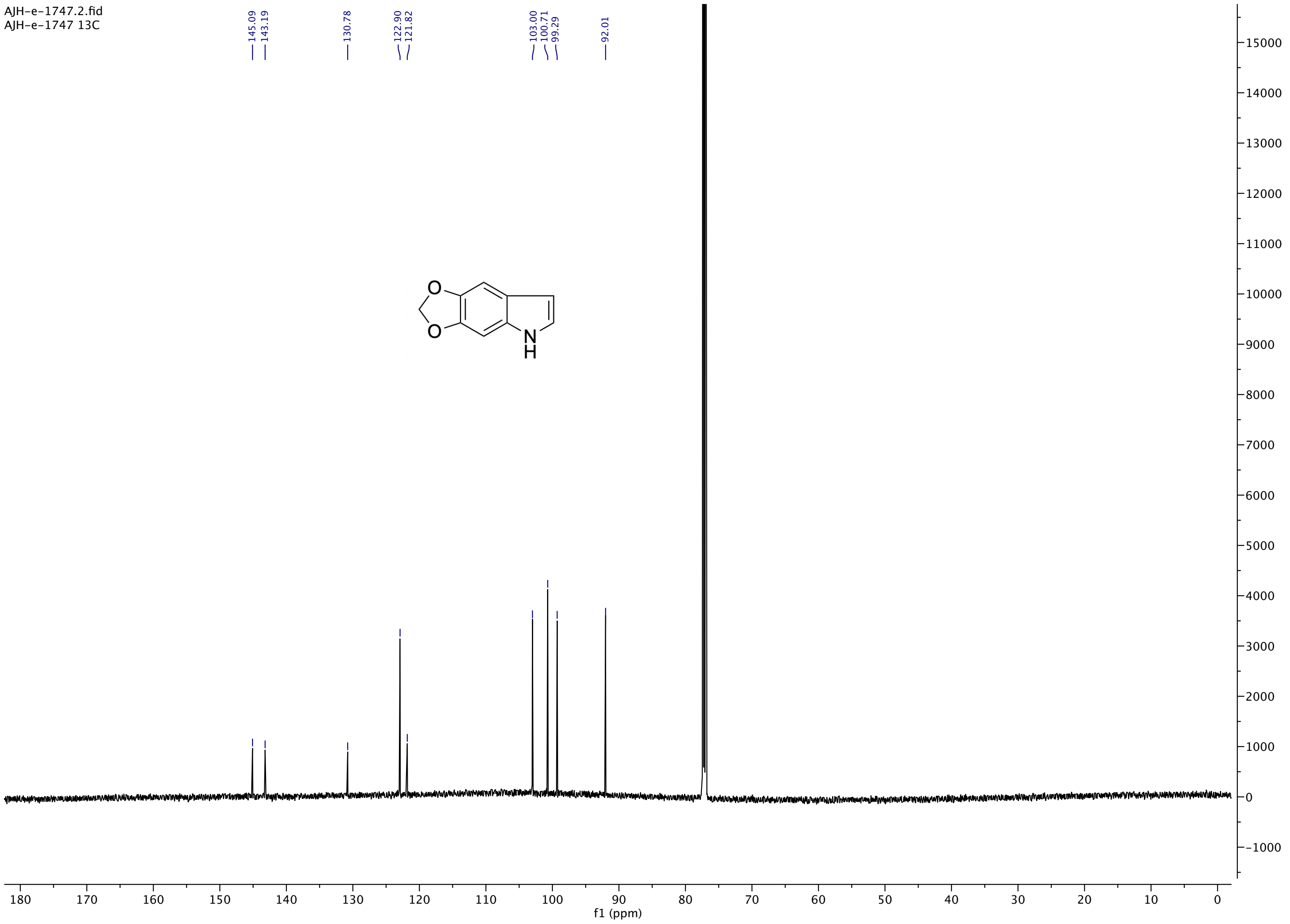

^{1}H NMR (600 MHz, $CDCl_3$) of Compound 10a

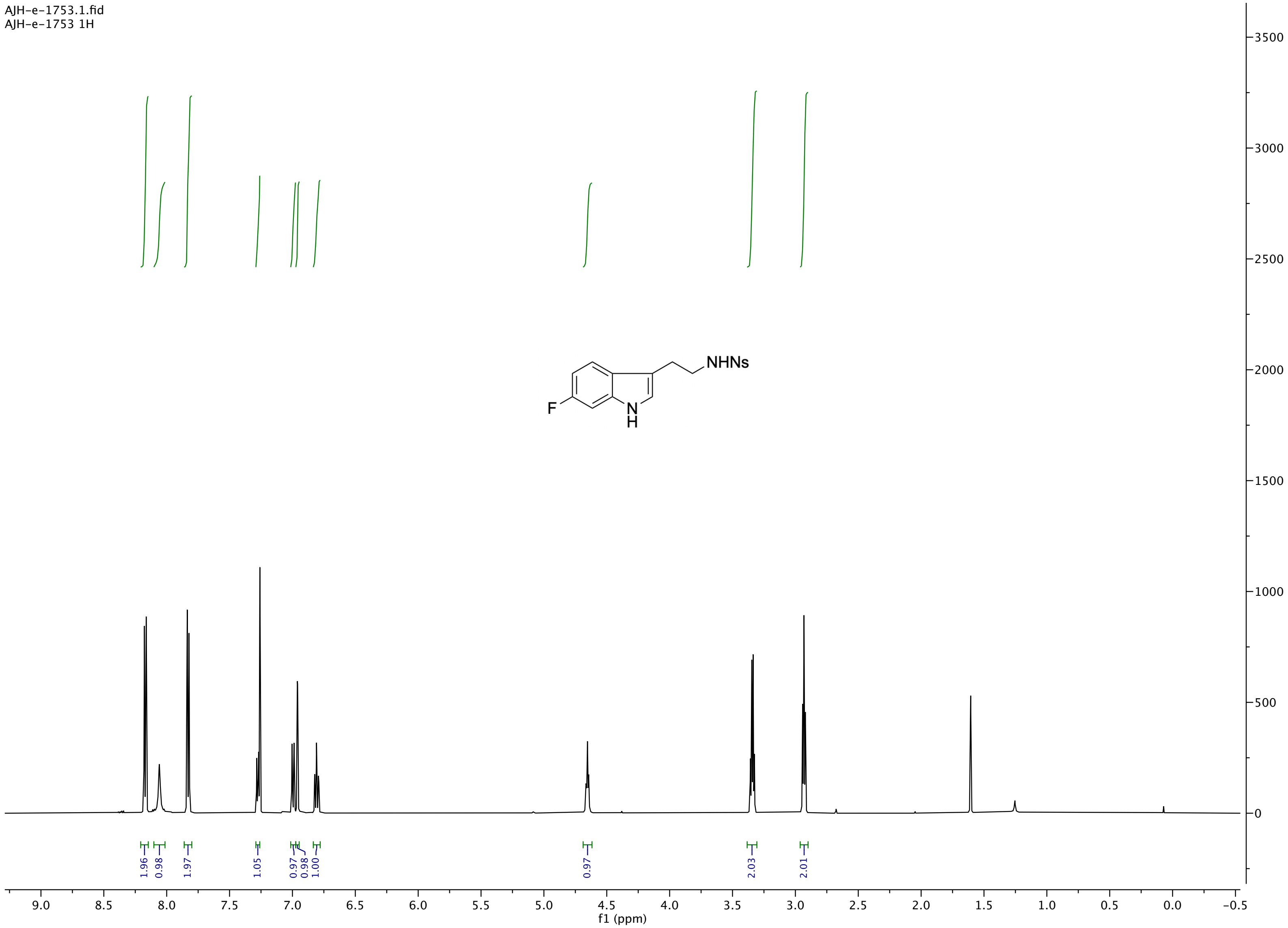

^{13}C NMR of (151 MHz, $CDCl_3$) of Compound 10a

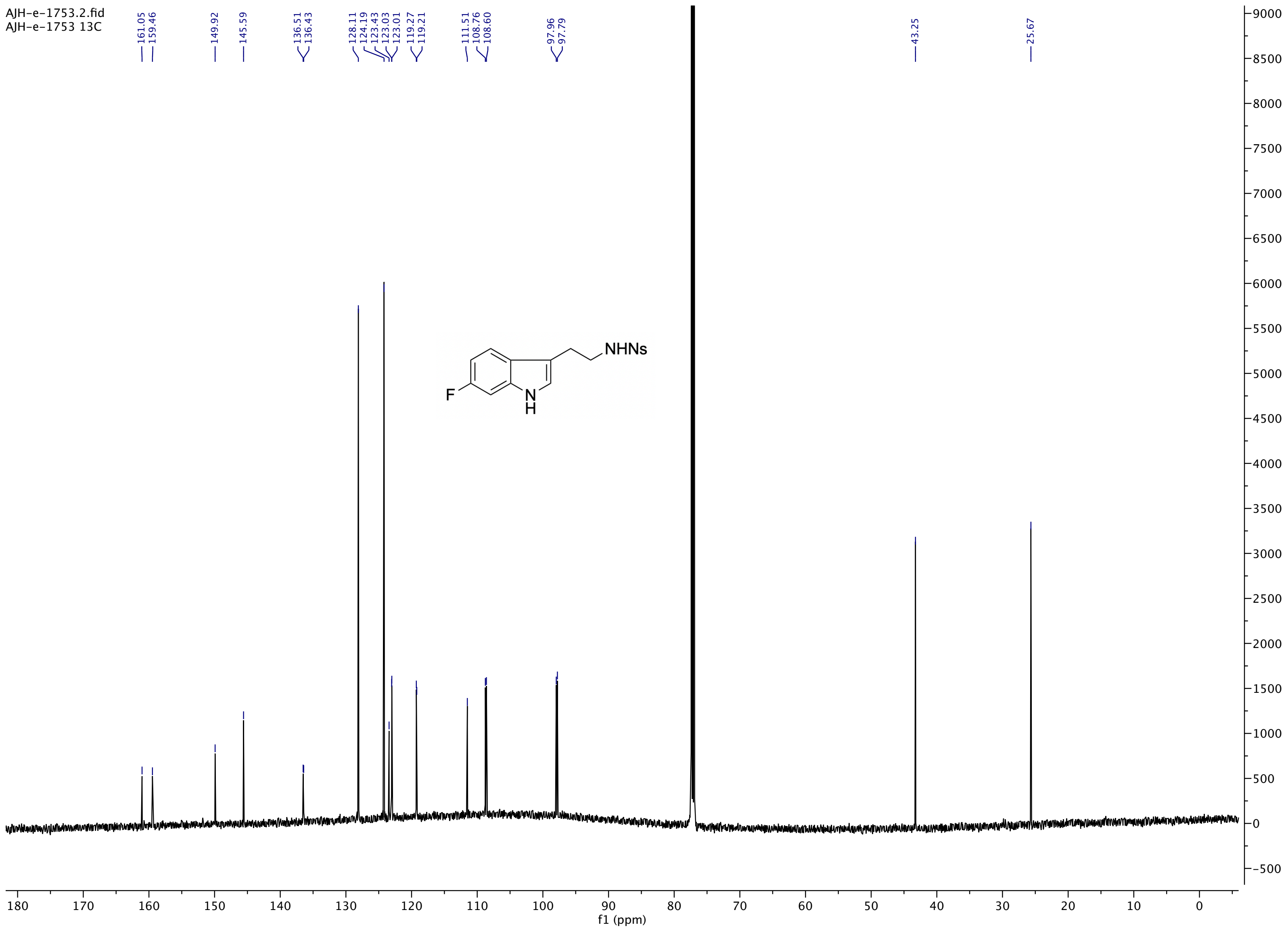

^{1}H NMR (600 MHz, DMSO) of Compound 10b

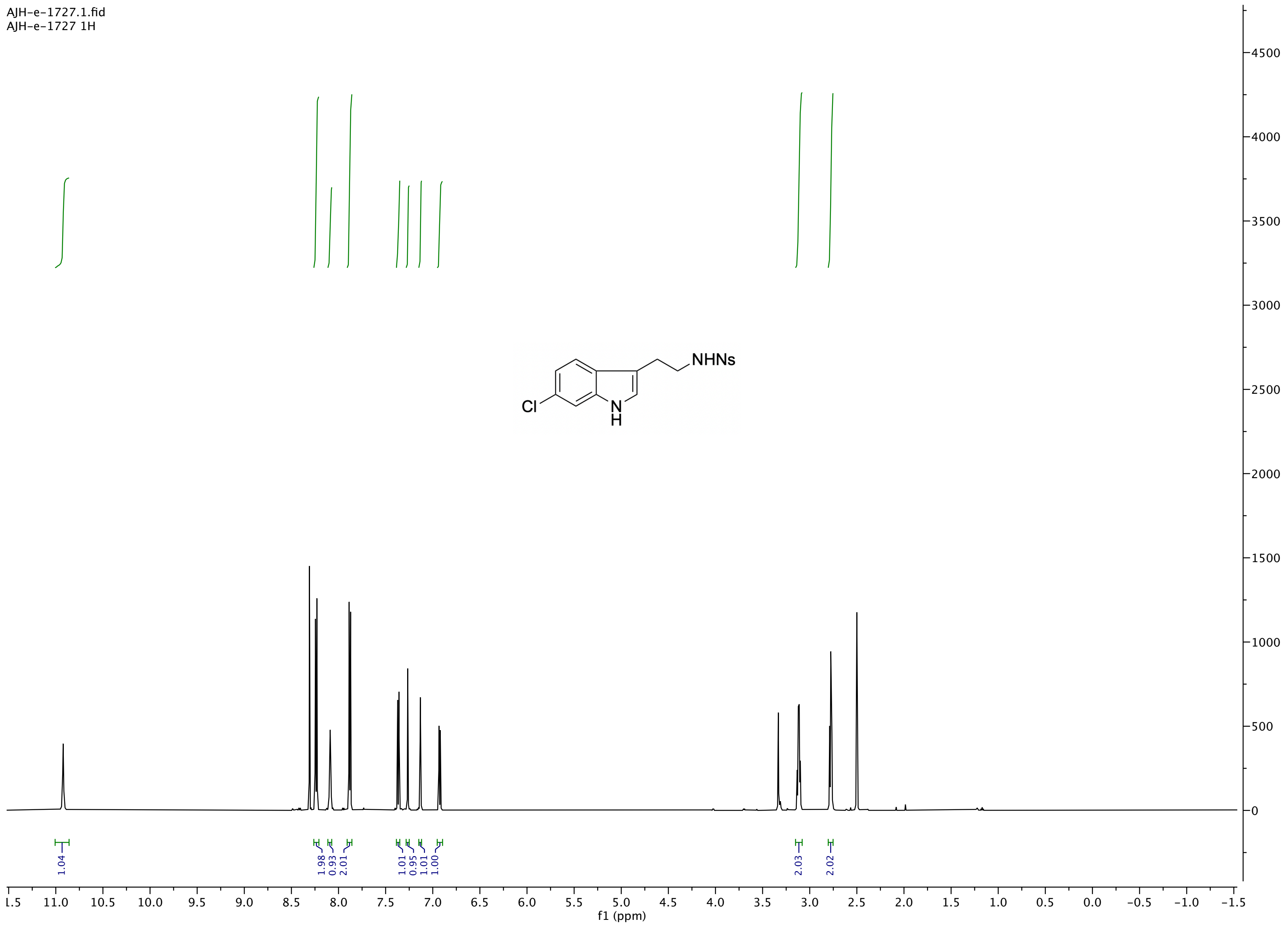

^{13}C NMR of (151 MHz, DMSO) of Compound 10b

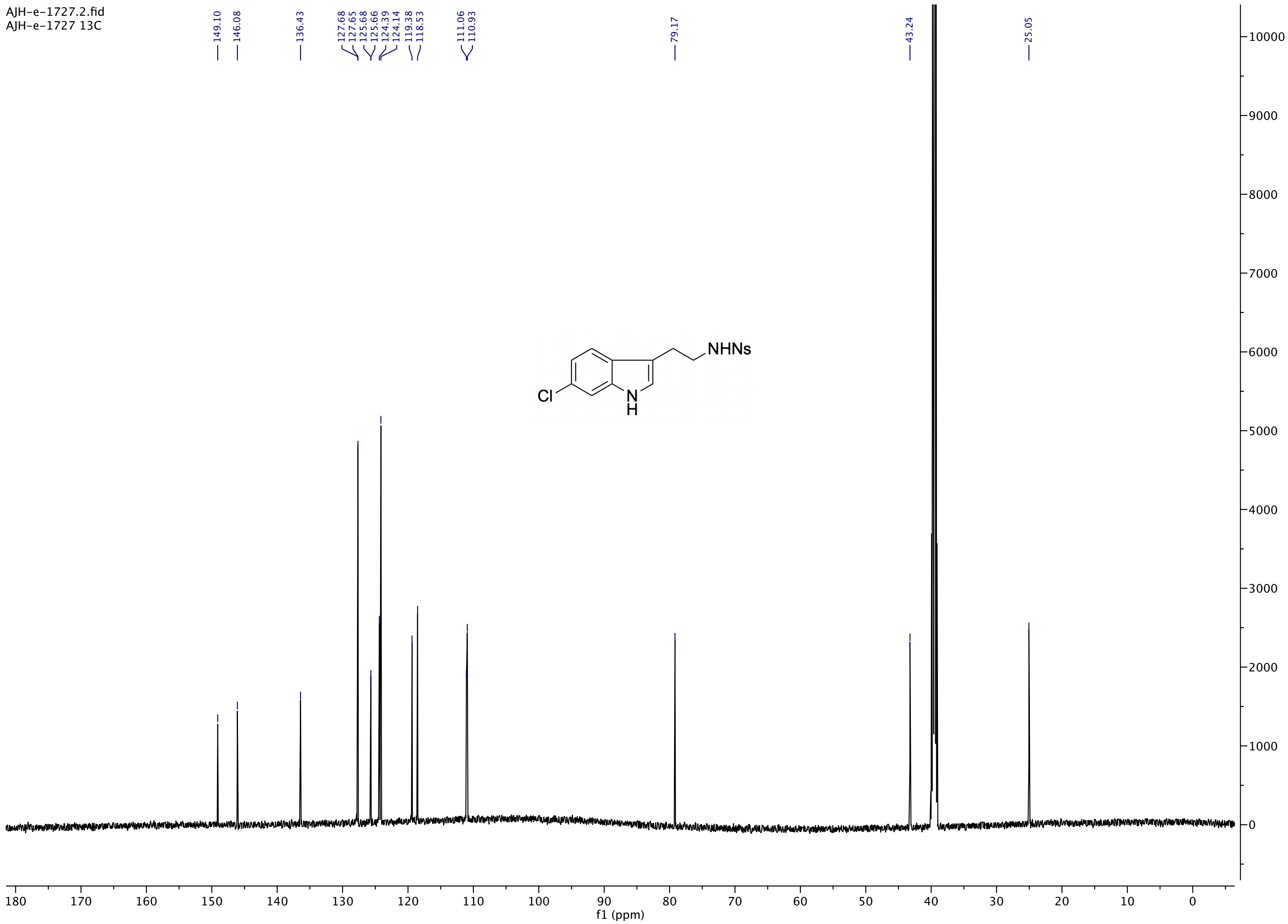

^{1}H NMR (600 MHz, DMSO) of Compound 10c

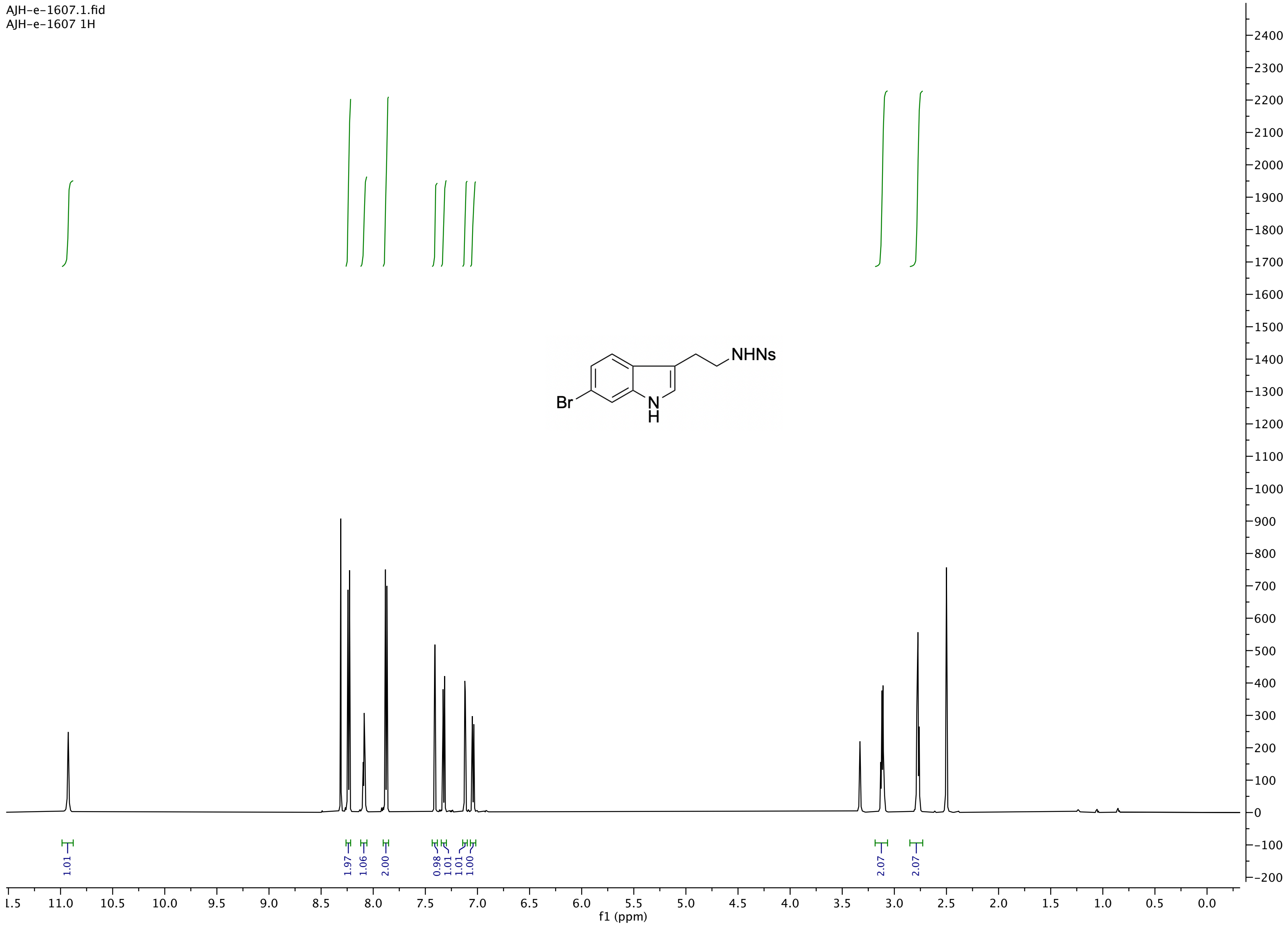

^{13}C NMR of (151 MHz, DMSO) of Compound 10c

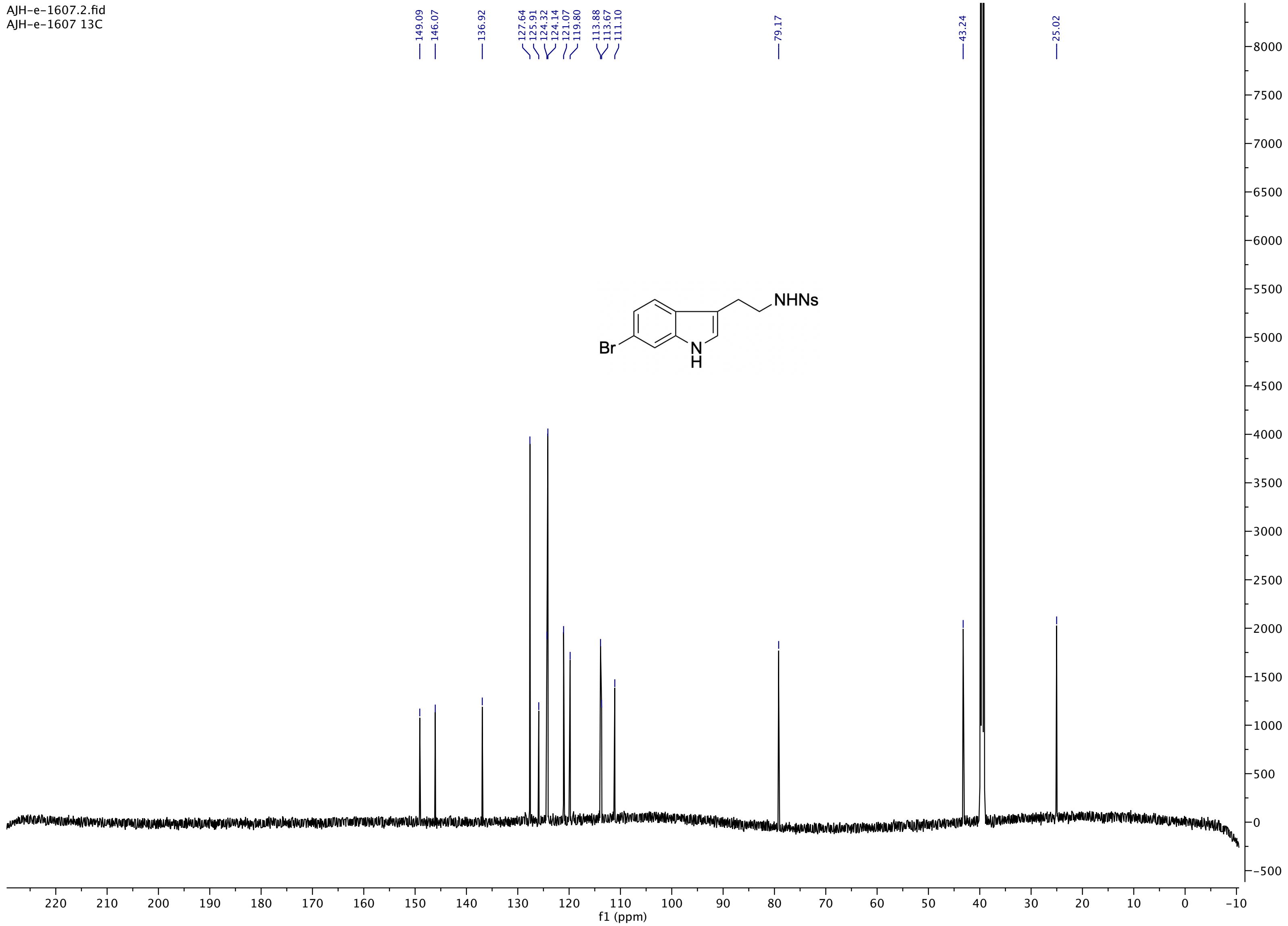

^{1}H NMR (600 MHz, DMSO) of Compound 10d

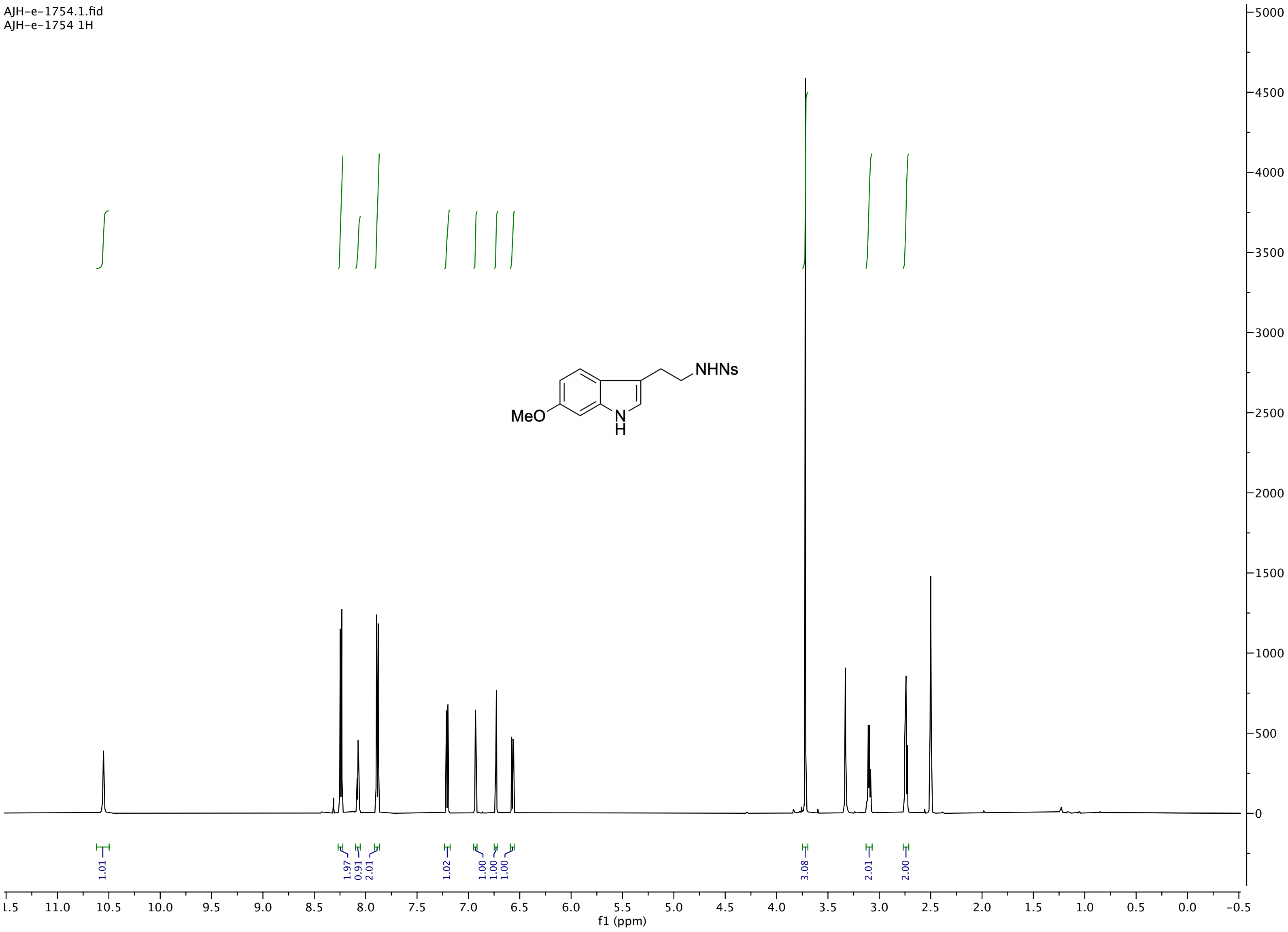

^{13}C NMR of (151 MHz, DMSO) of Compound 10d

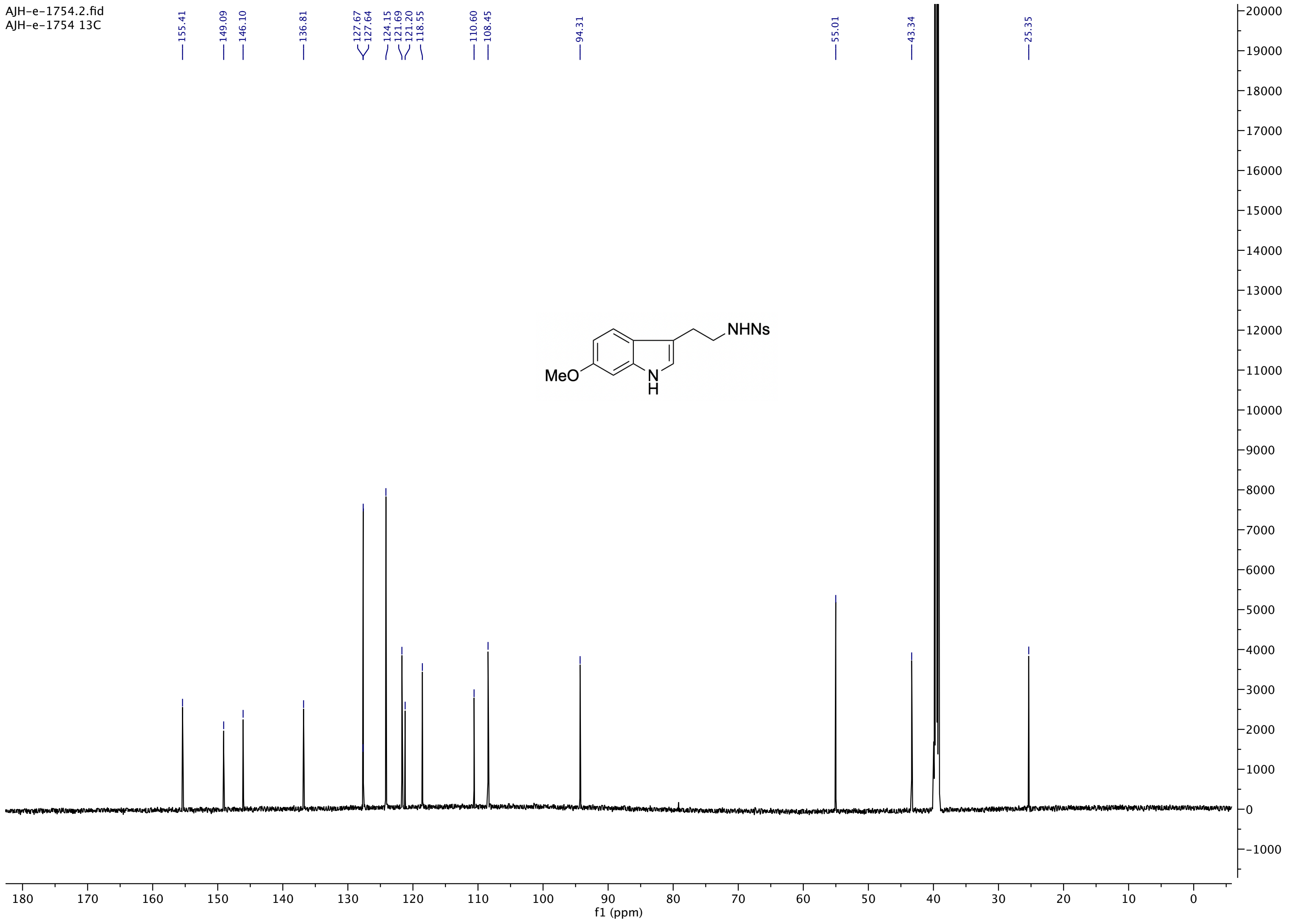

^{1}H NMR (600 MHz, DMSO) of Compound 10e

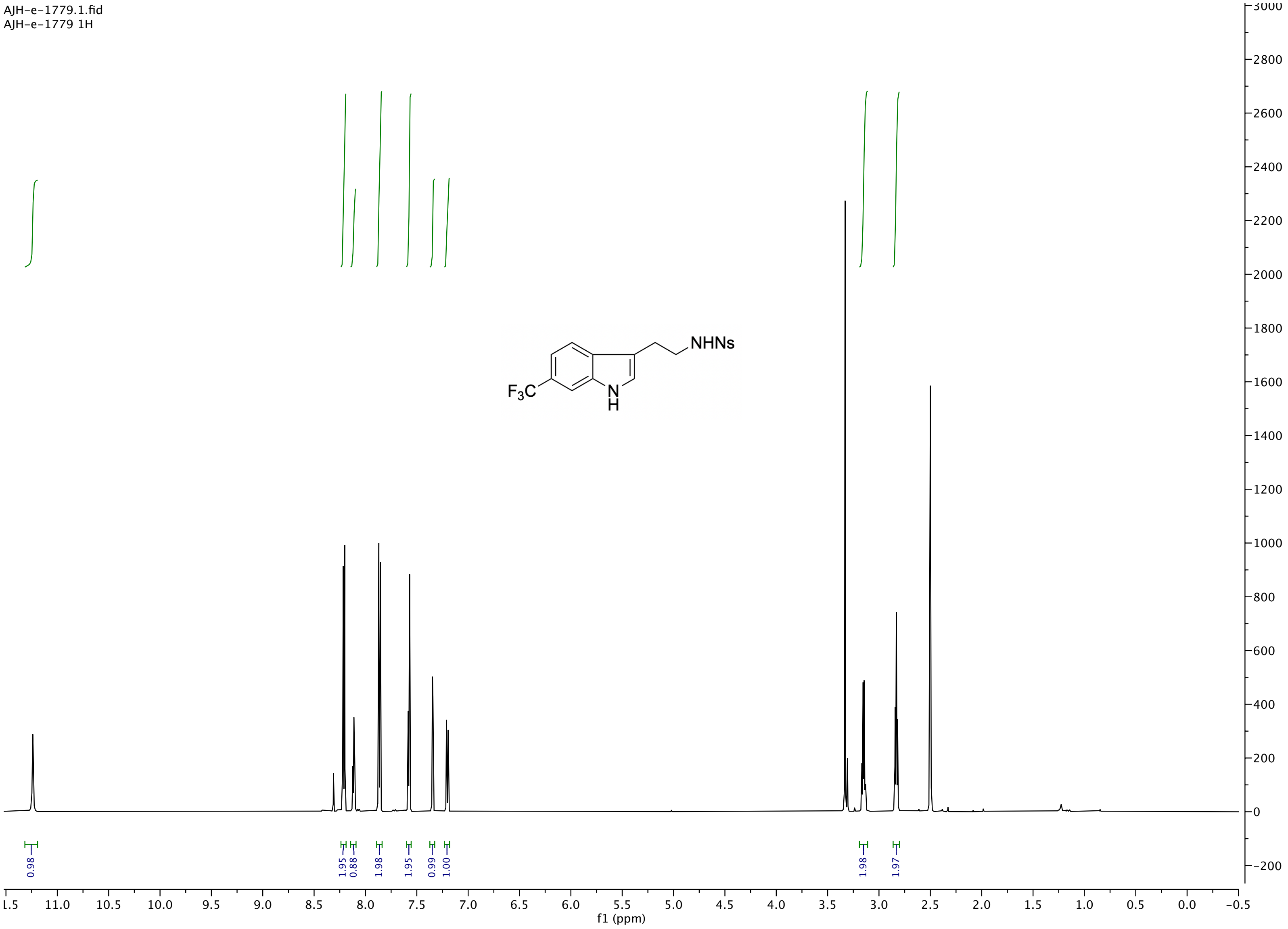

^{13}C NMR of (151 MHz, DMSO) of Compound 10e

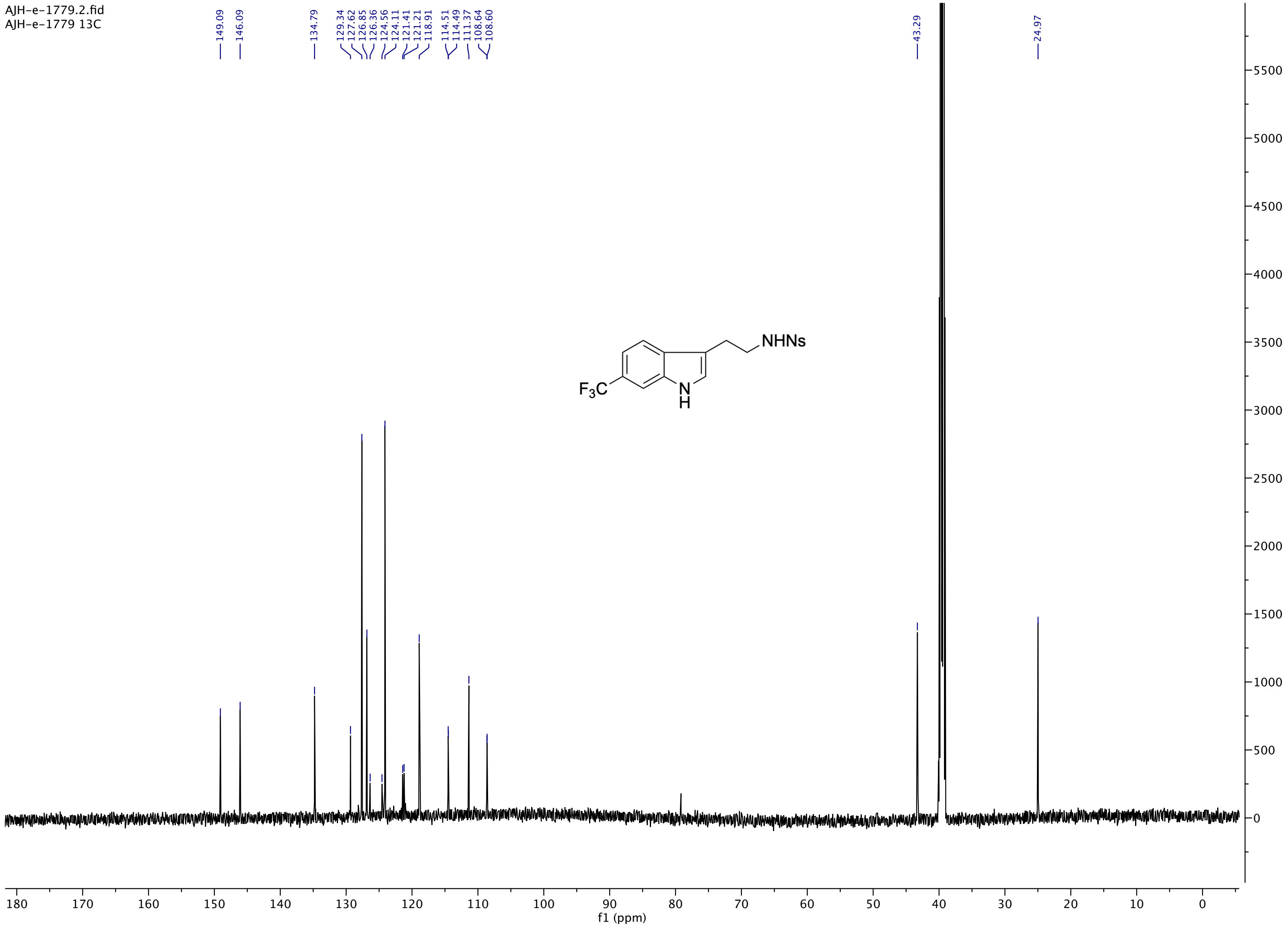

^{1}H NMR (600 MHz, DMSO) of Compound 10f

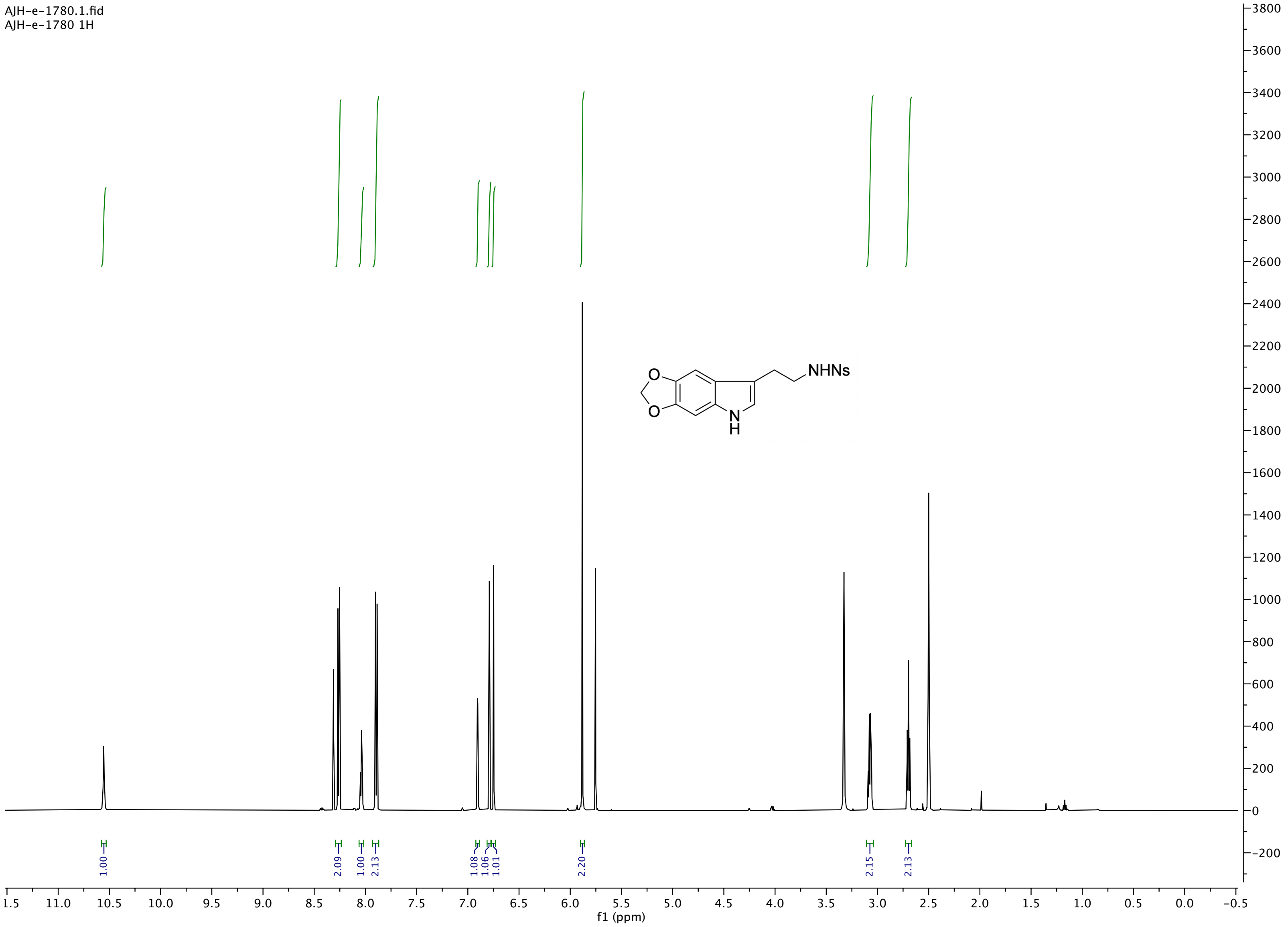

^{13}C NMR of (151 MHz, DMSO) of Compound 10f

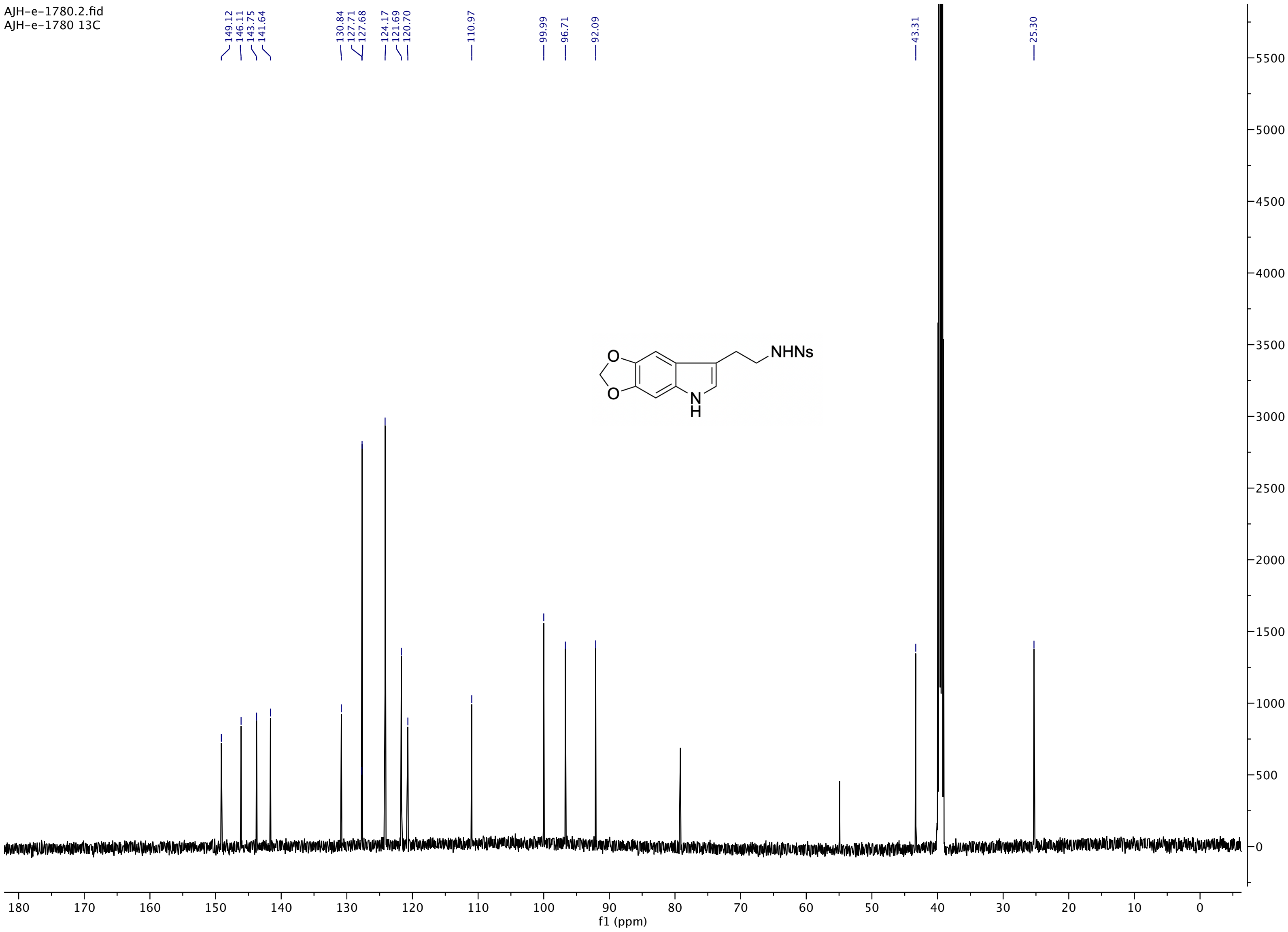

^{1}H NMR (600 MHz, $CDCl_3$) of Compound 11g

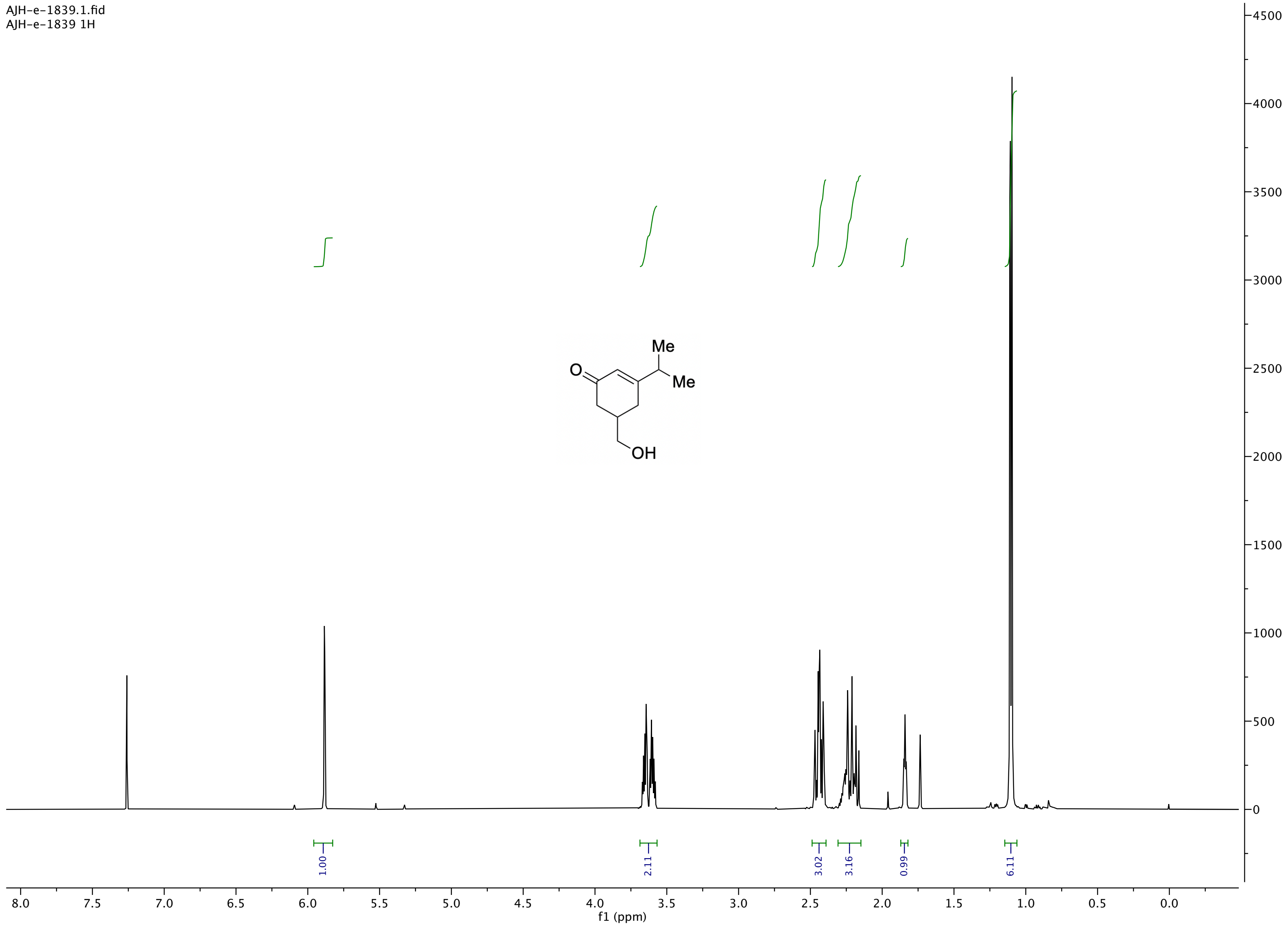

^{13}C NMR of (151 MHz, $CDCl_3$) of Compound 11h

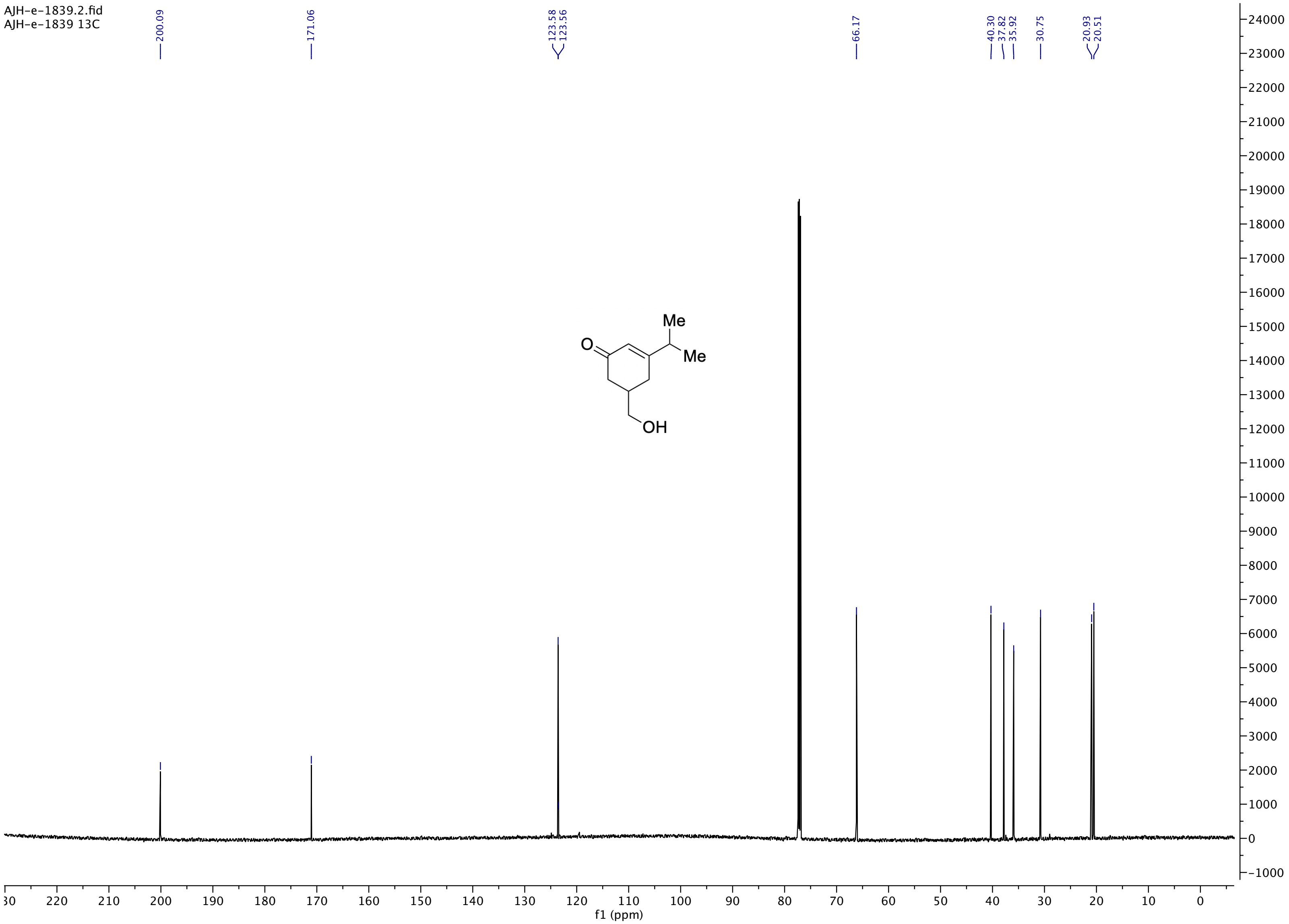

^{1}H NMR (600 MHz, $CDCl_3$) of Compound 11g

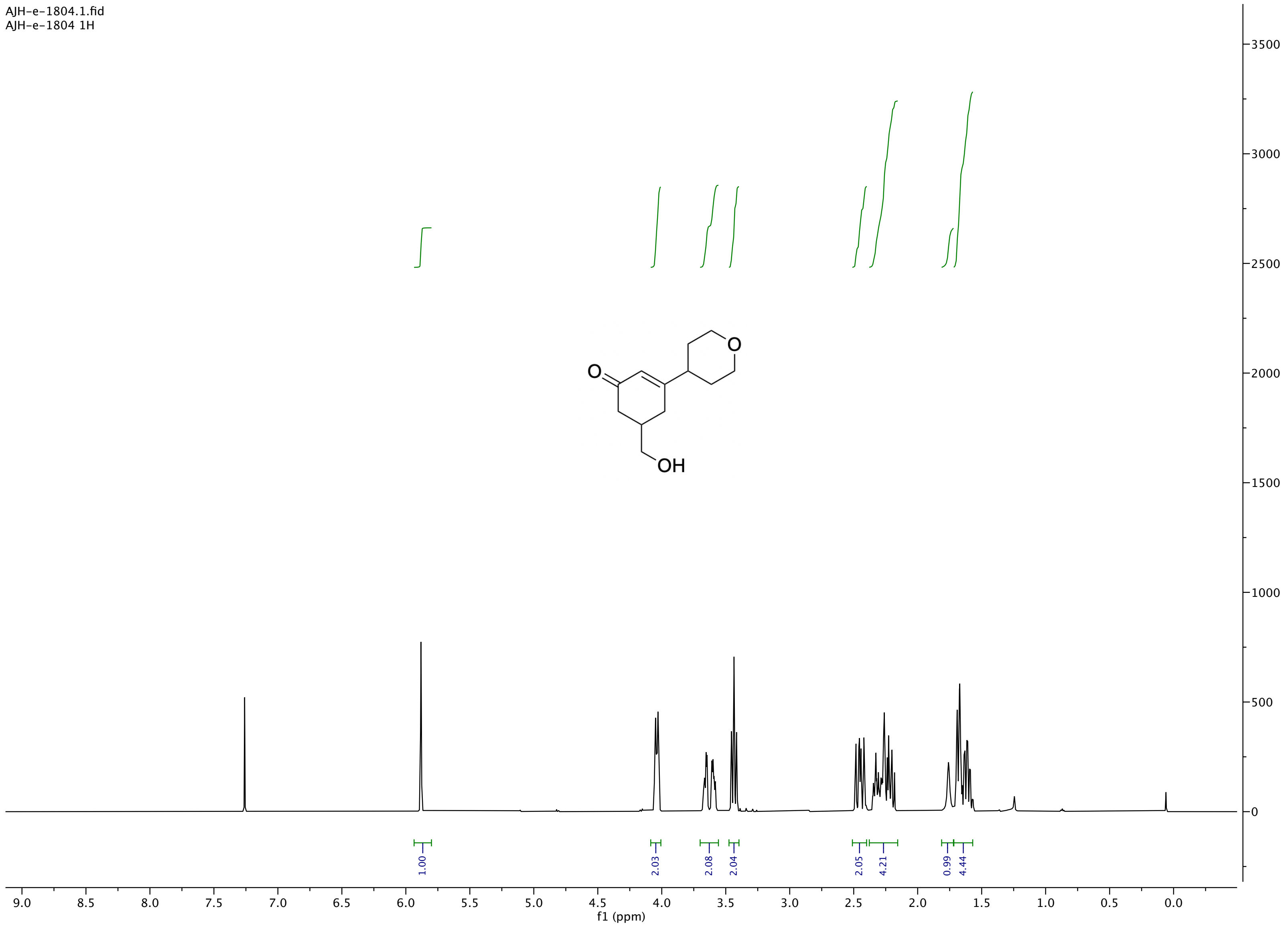

^{13}C NMR of (151 MHz, $CDCl_3$) of Compound 11g

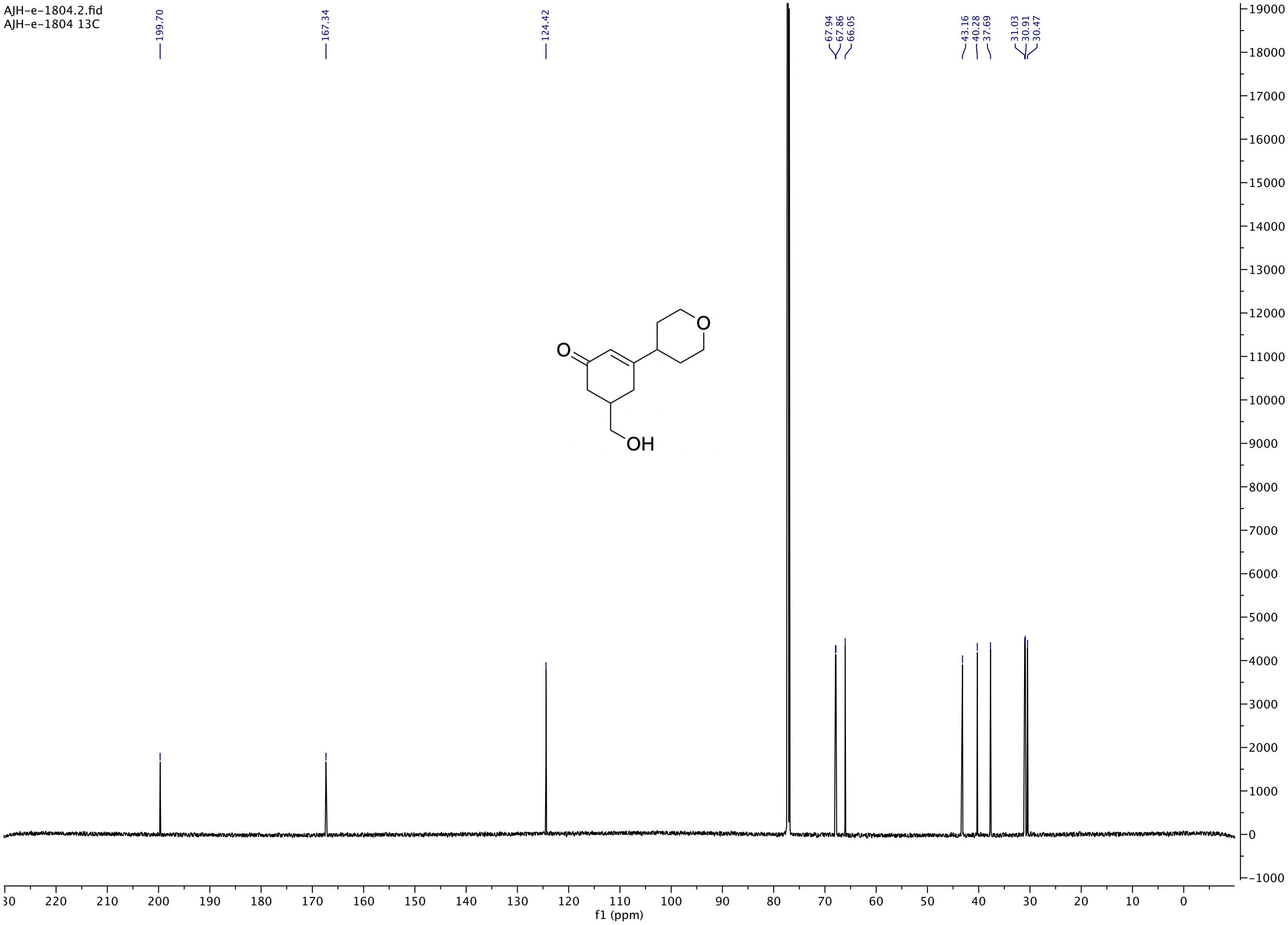

^{1}H NMR (600 MHz, CDCl₃) of Compound 18a

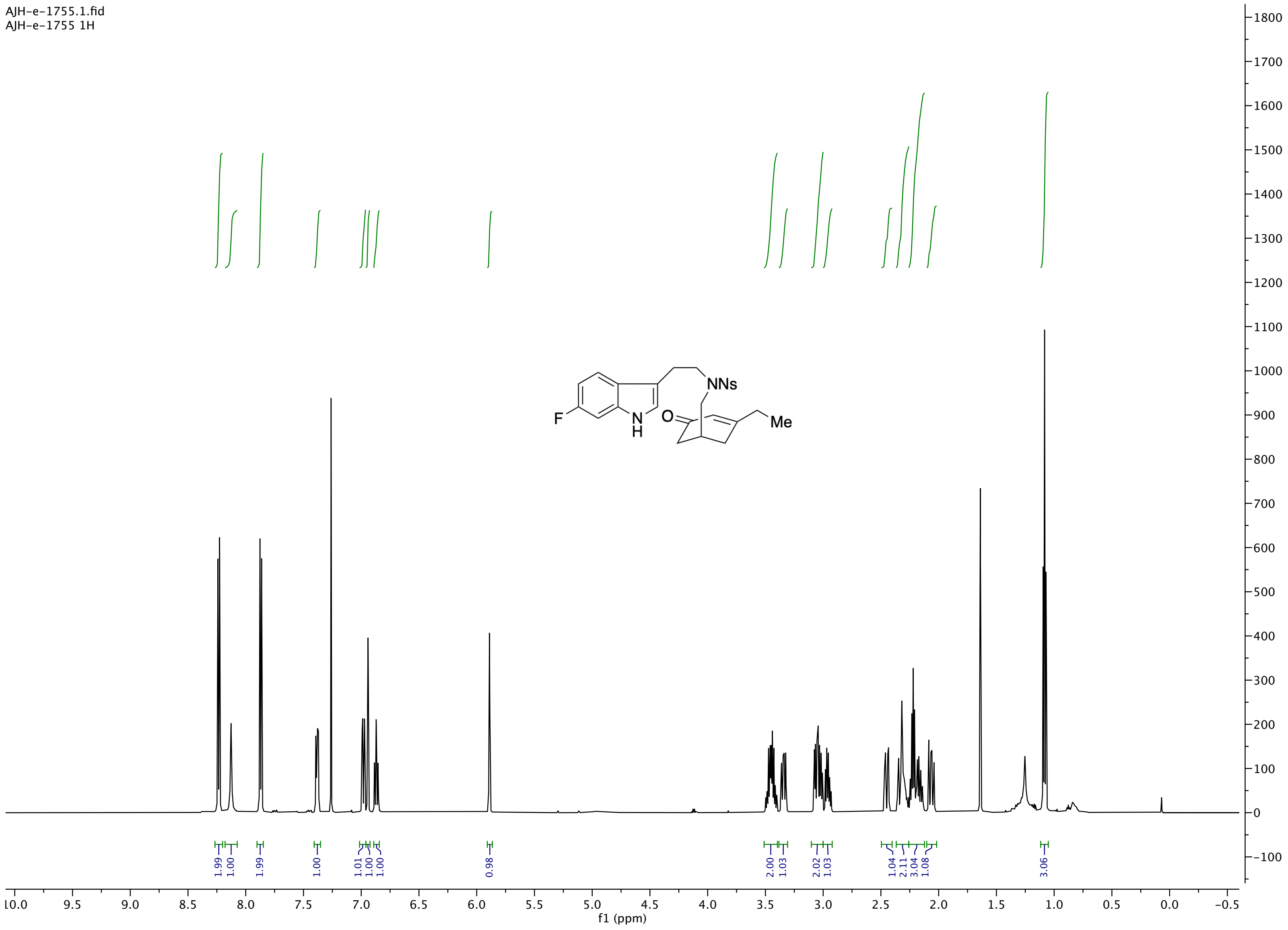

^{13}C NMR of (151 MHz, $CDCl_3$) of Compound 18a

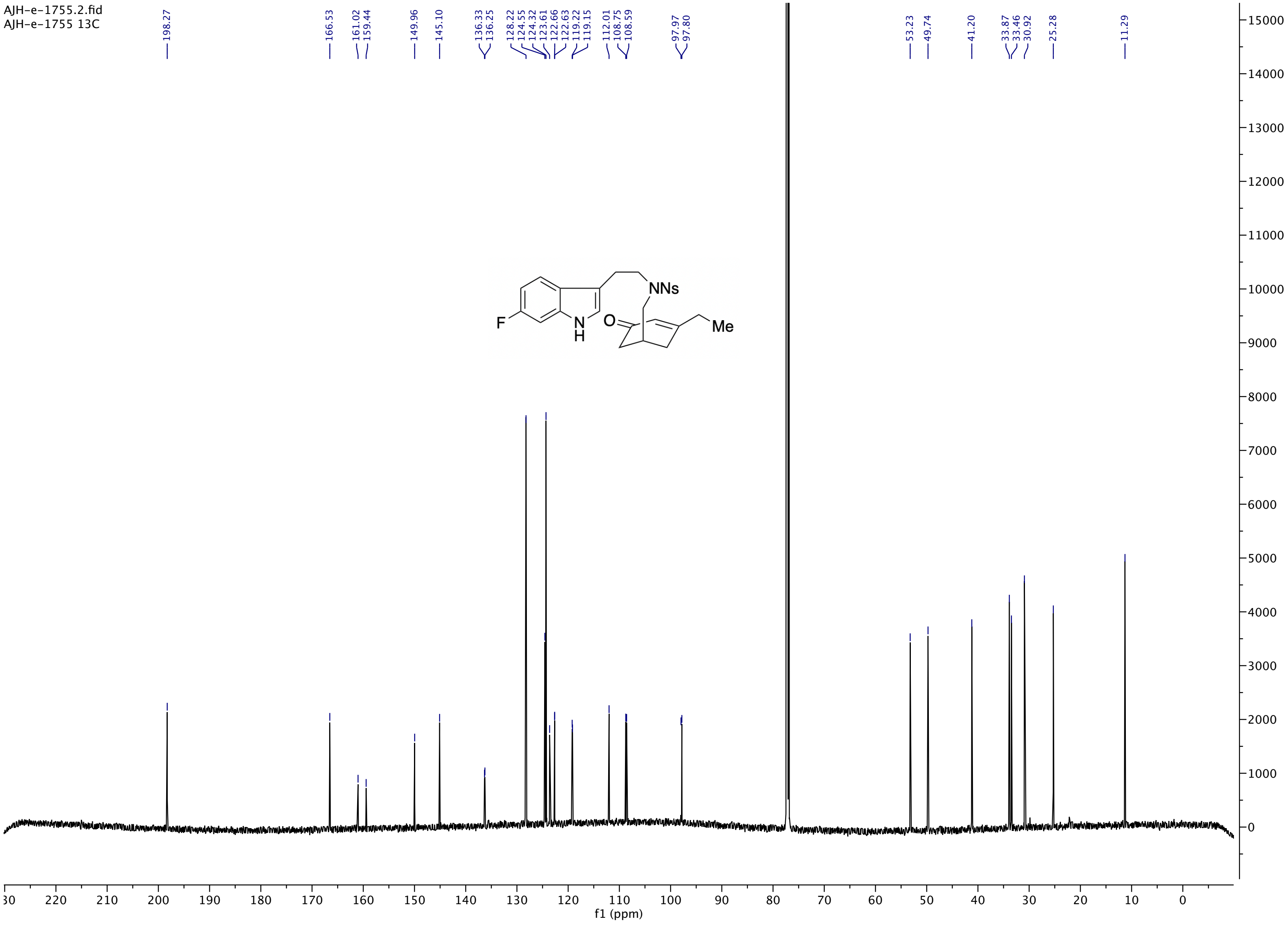

^{1}H NMR (600 MHz, $CDCl_3$) of Compound 18b

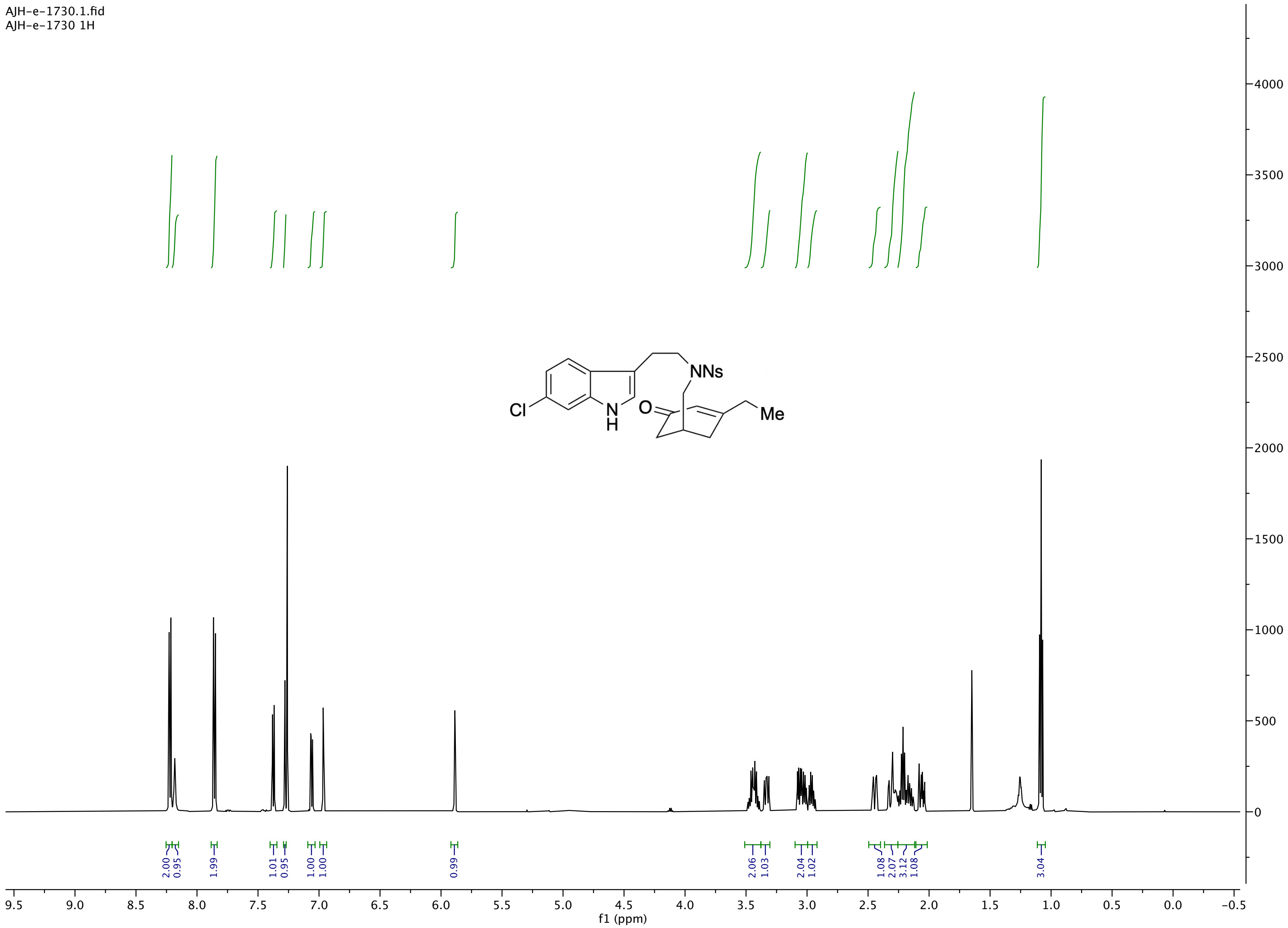

^{13}C NMR of (151 MHz, $CDCl_3$) of Compound 18b

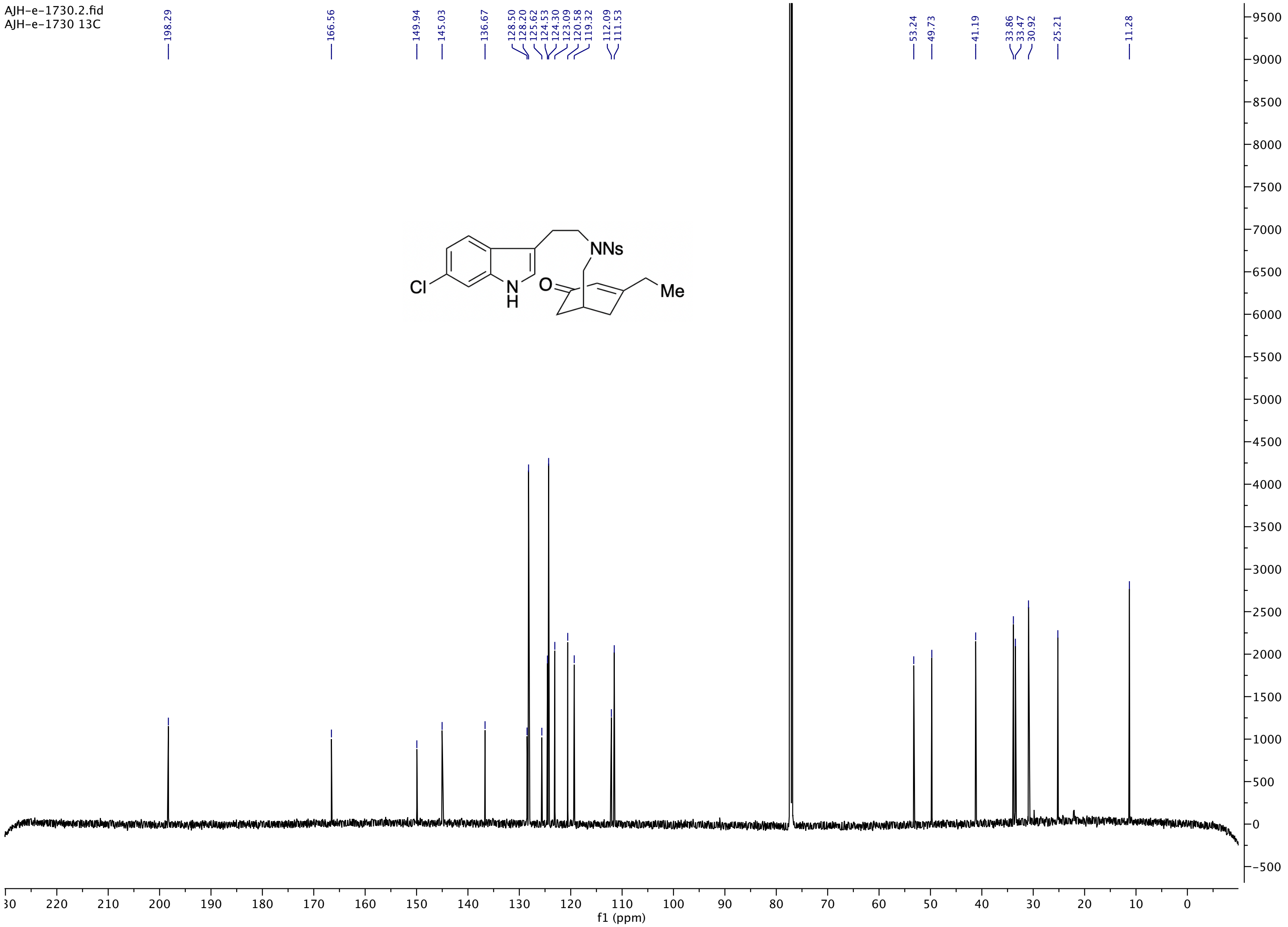

^{1}H NMR (600 MHz, $CDCl_3$) of Compound 18c

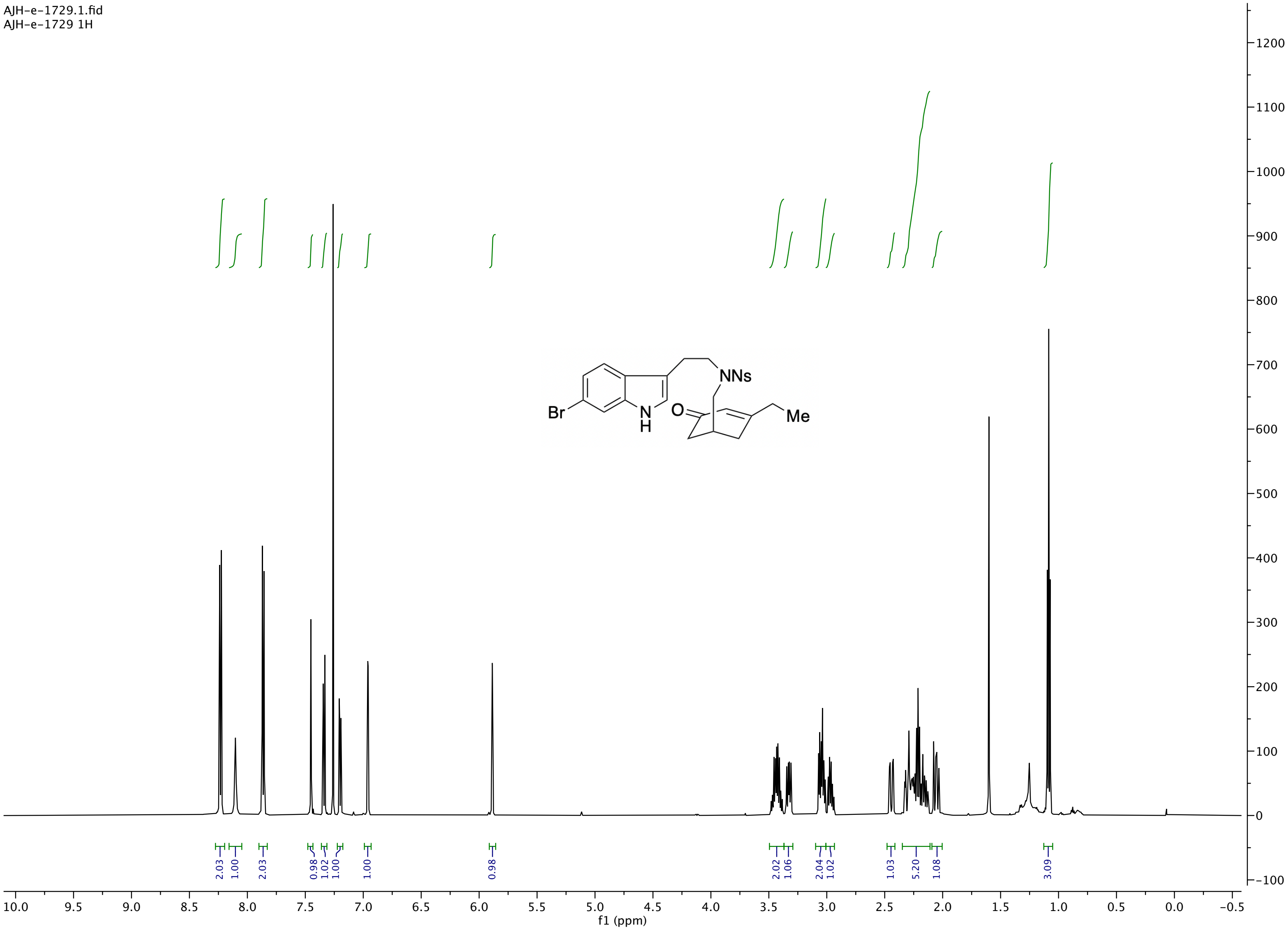

^{13}C NMR of (151 MHz, $CDCl_3$) of Compound 18c

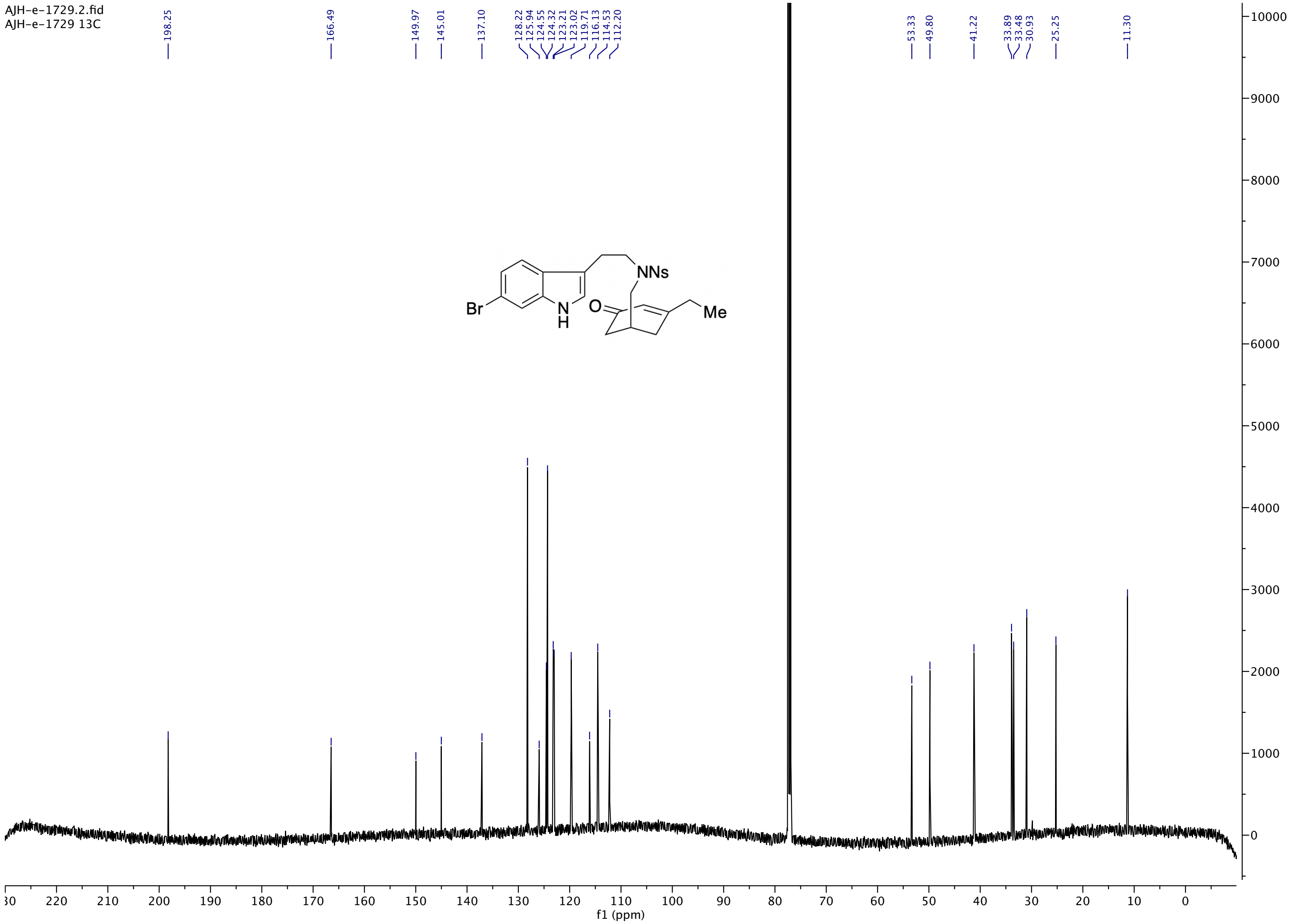

^{1}H NMR (600 MHz, $CDCl_3$) of Compound 18d

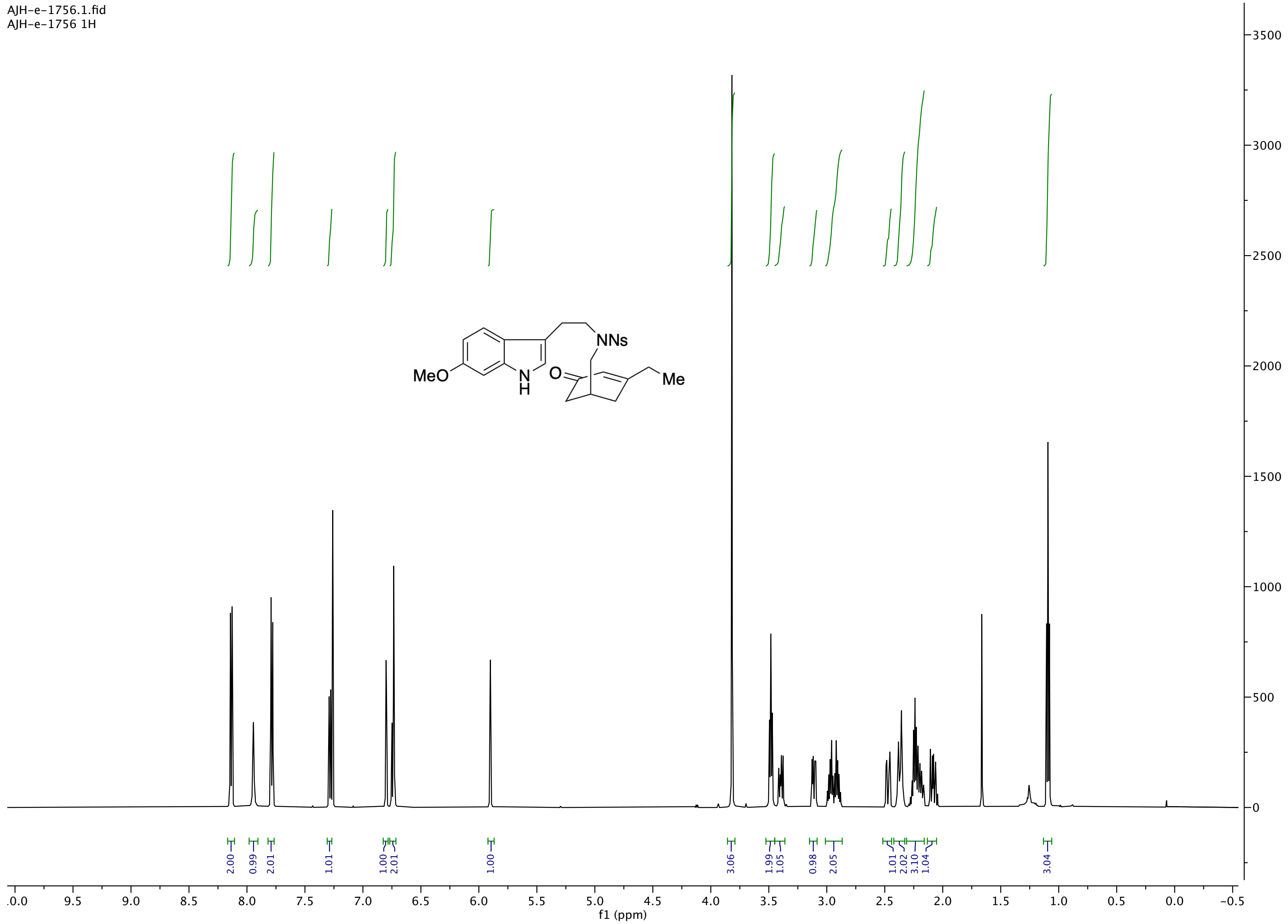

^{13}C NMR of (151 MHz, $CDCl_3$) of Compound 18d

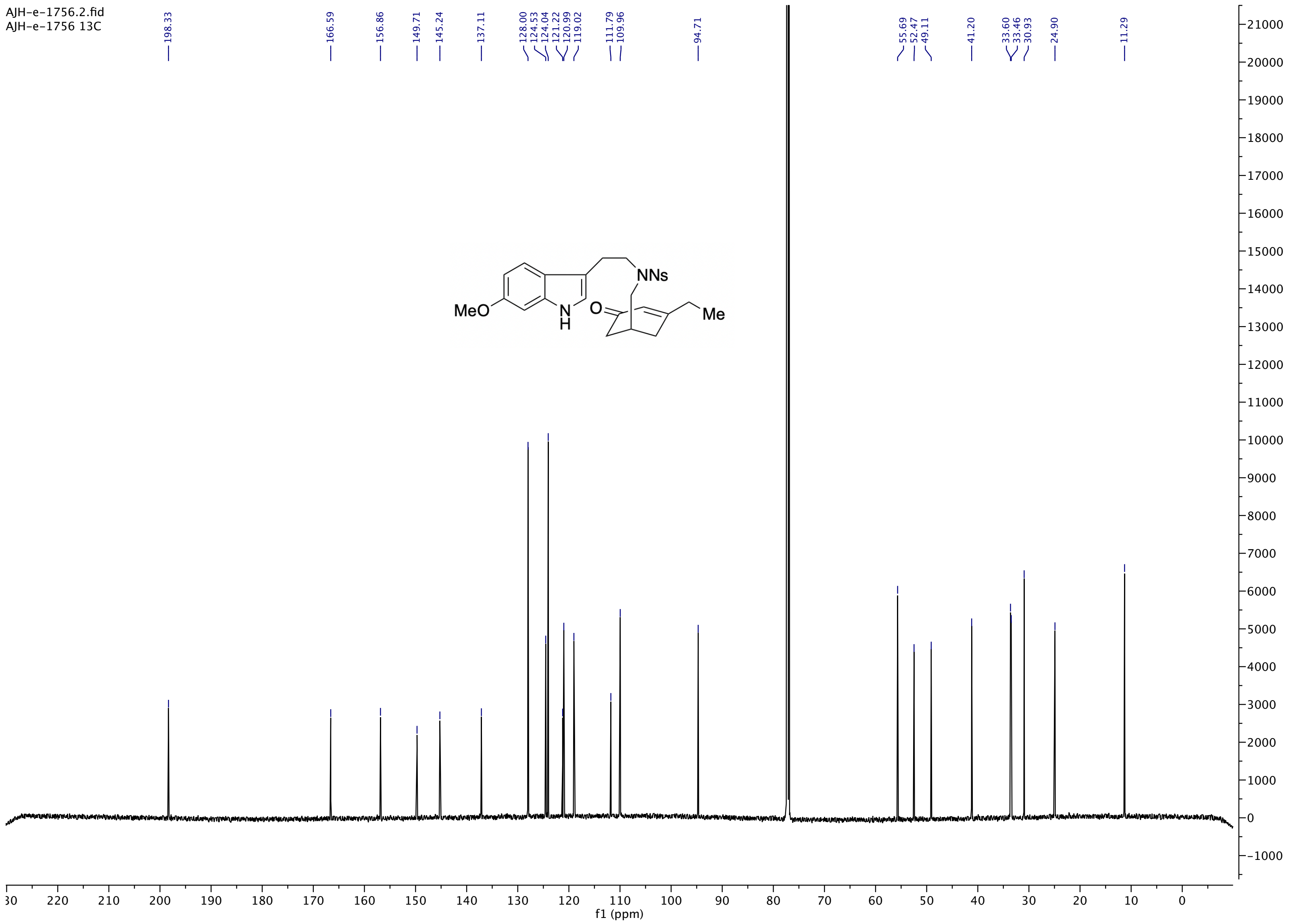

^{1}H NMR (600 MHz, CDCl3) of Compound 18e

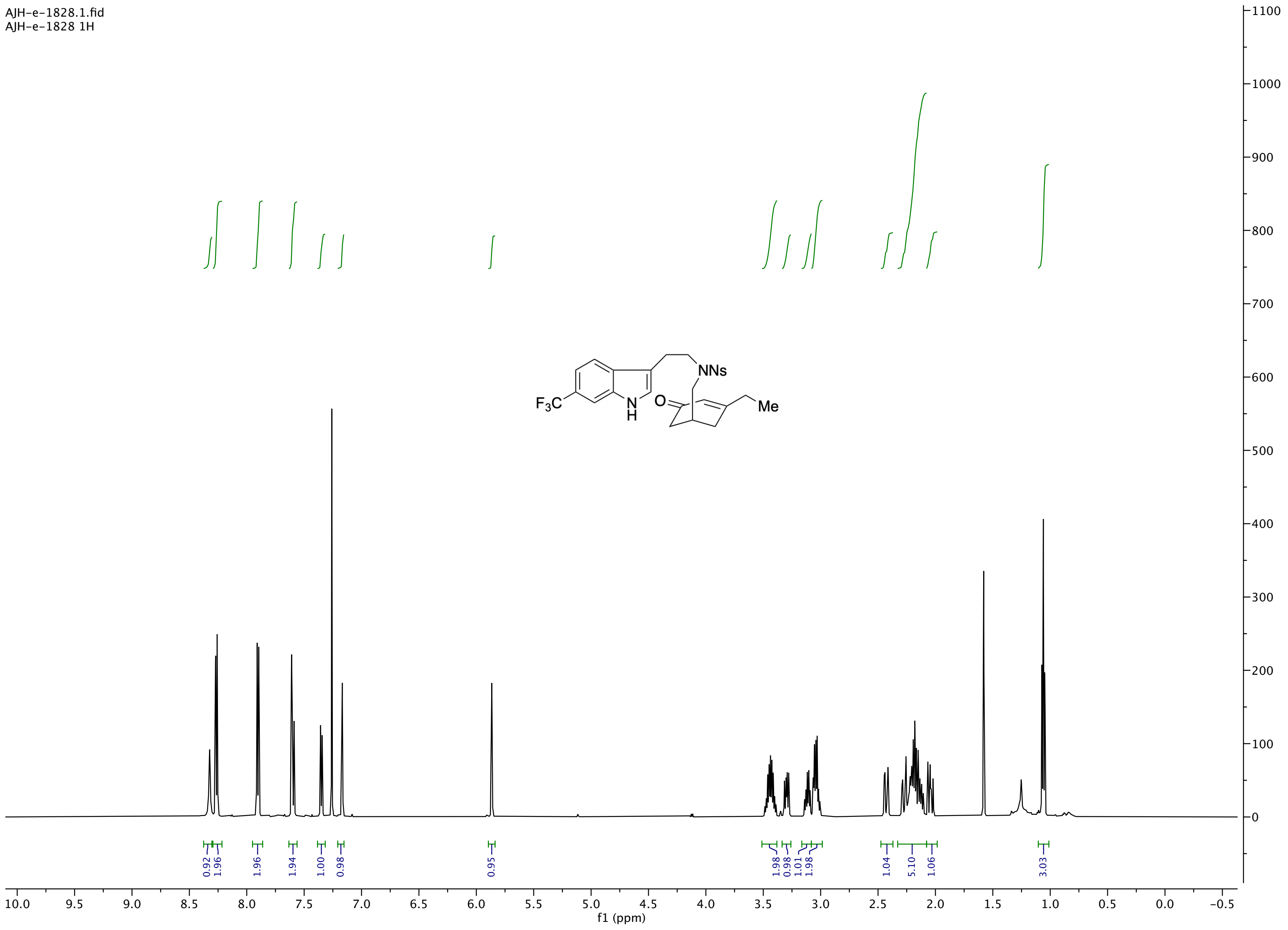

^{13}C NMR of (151 MHz, $CDCl_3$) of Compound 18e

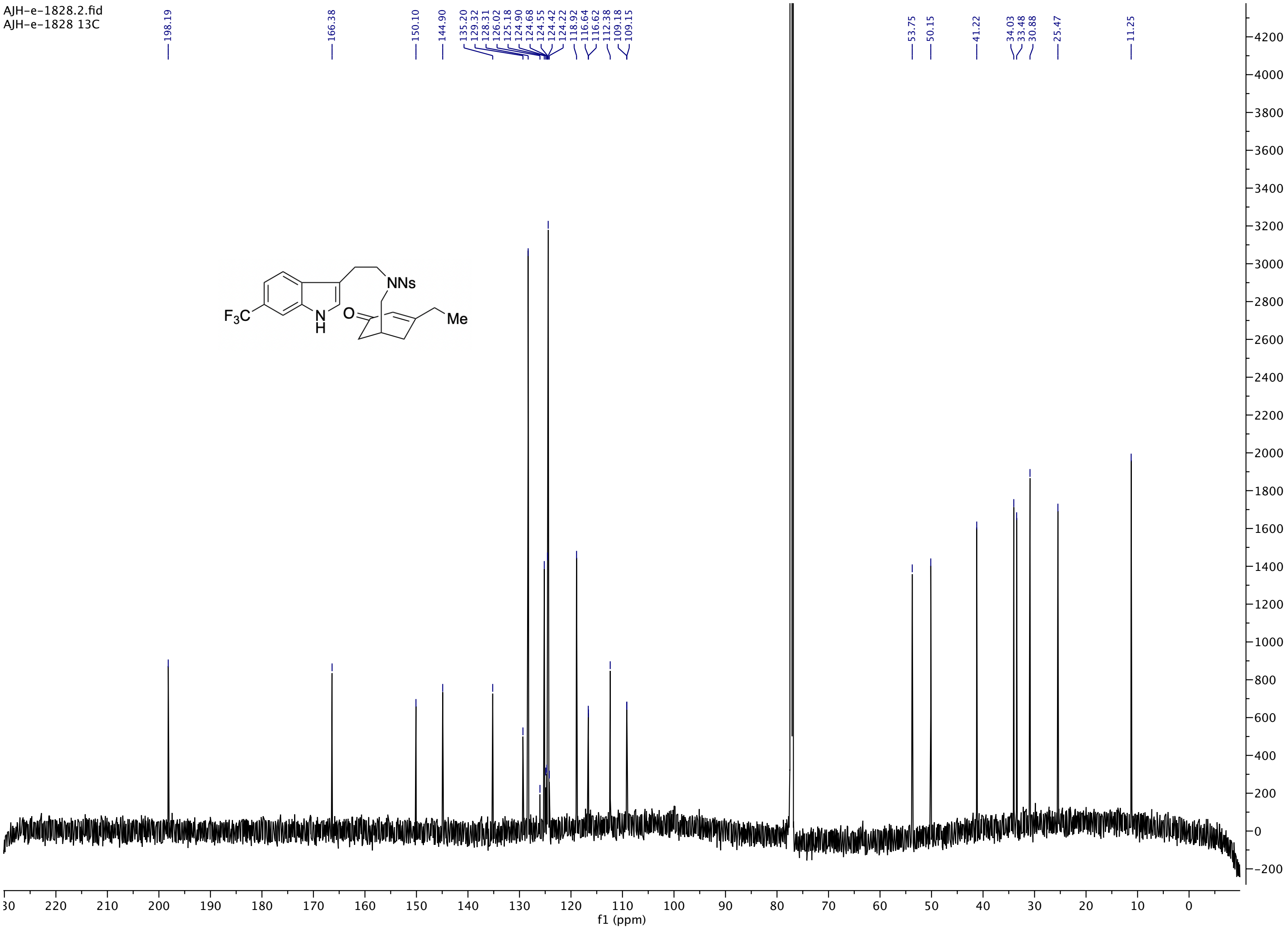

^{1}H NMR (600 MHz, $CDCl_3$) of Compound 18f

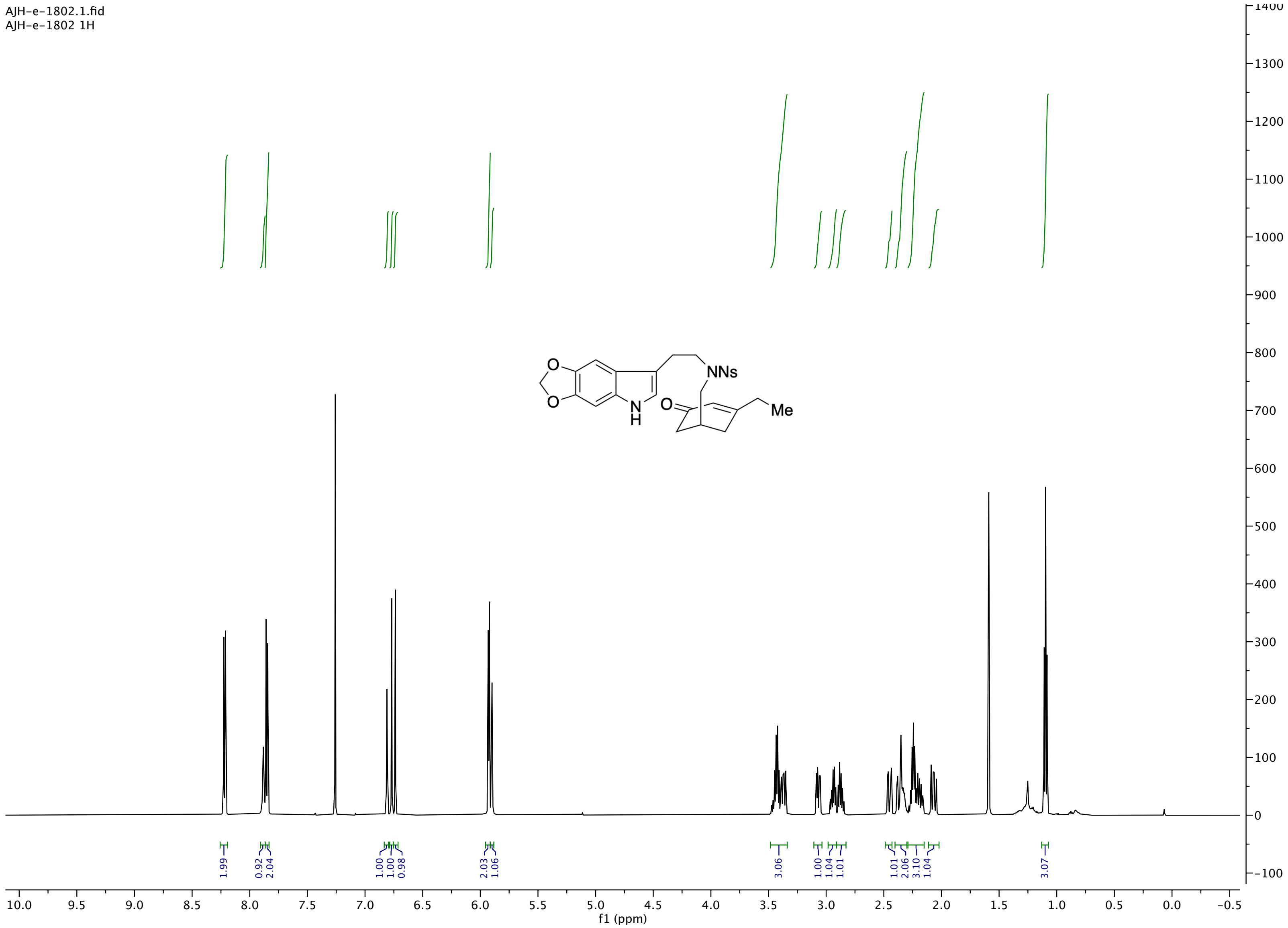

^{13}C NMR of (151 MHz, $CDCl_3$) of Compound 18f

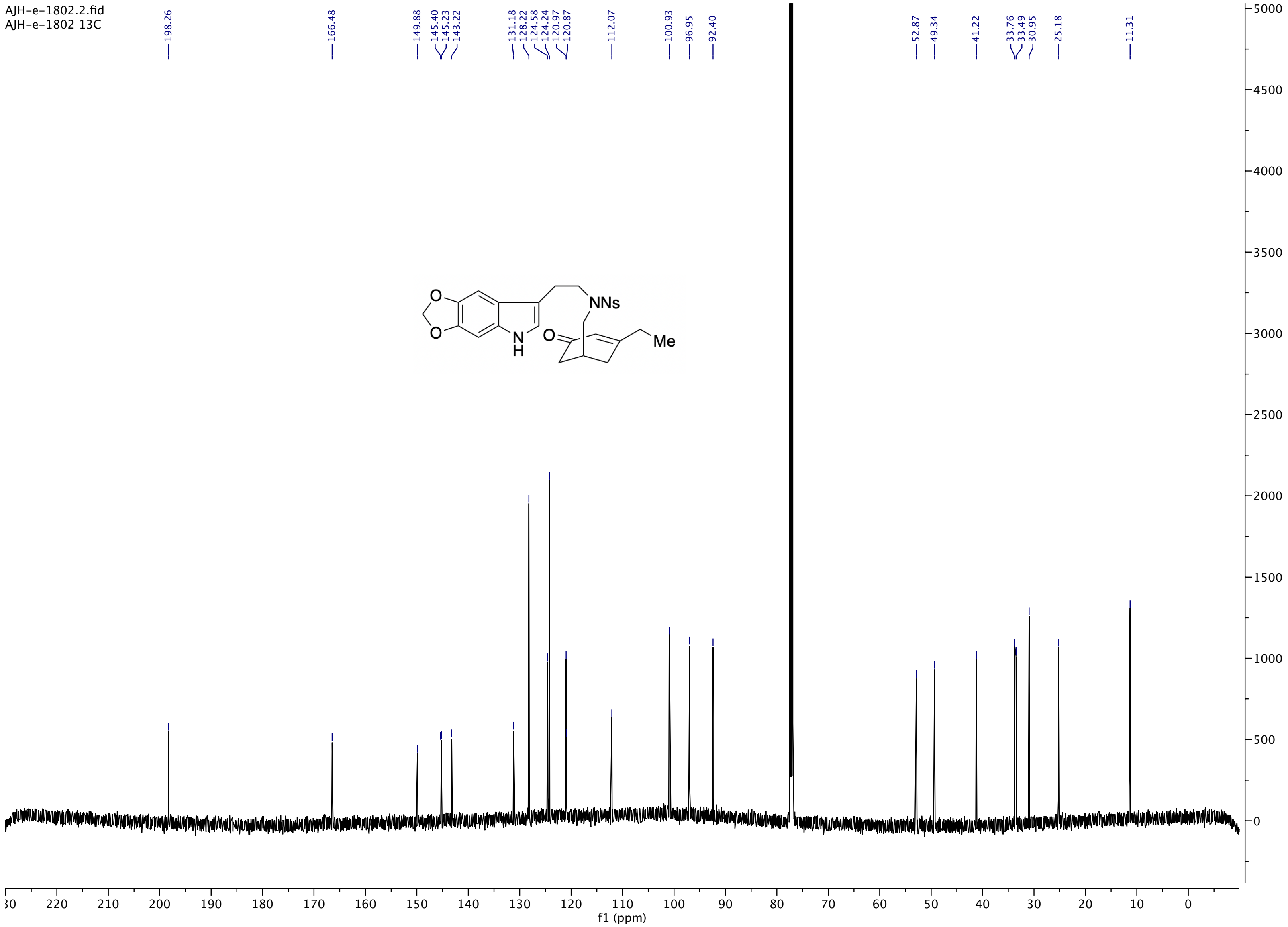

^{1}H NMR (600 MHz, $CDCl_3$) of Compound 18g

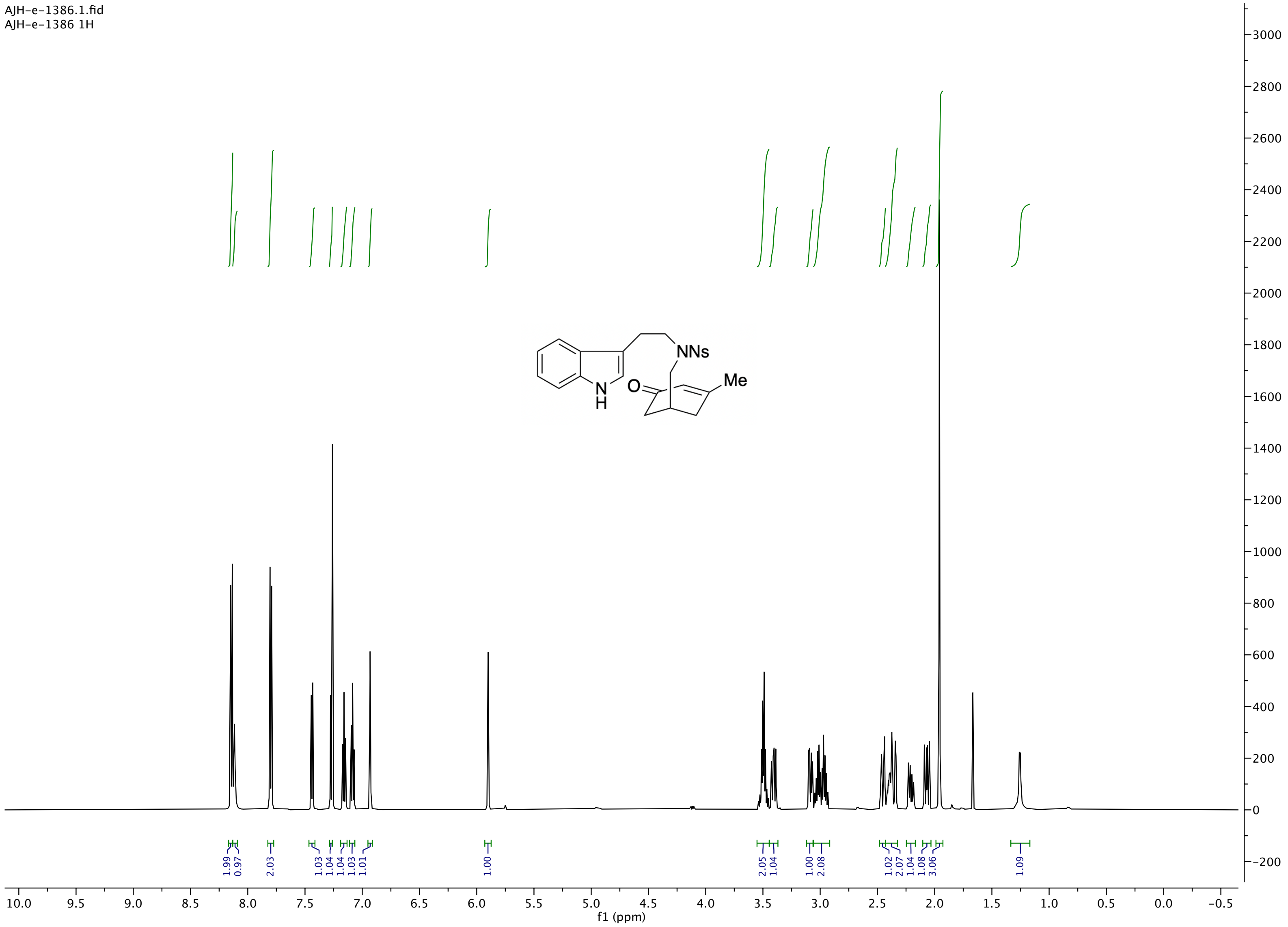

^{13}C NMR of (151 MHz, $CDCl_3$) of Compound 18g

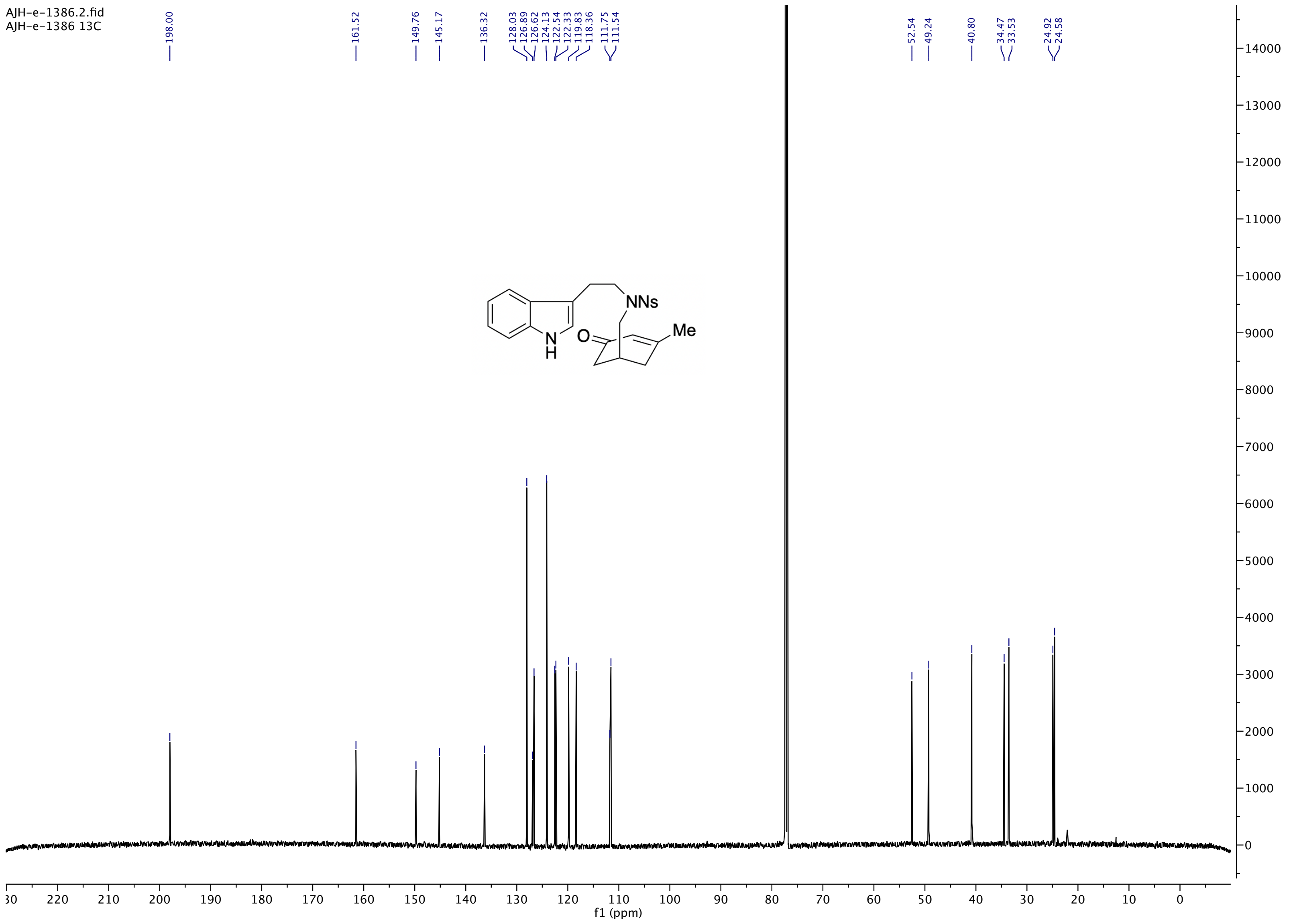

^{1}H NMR (600 MHz, $CDCl_3$) of Compound 18h

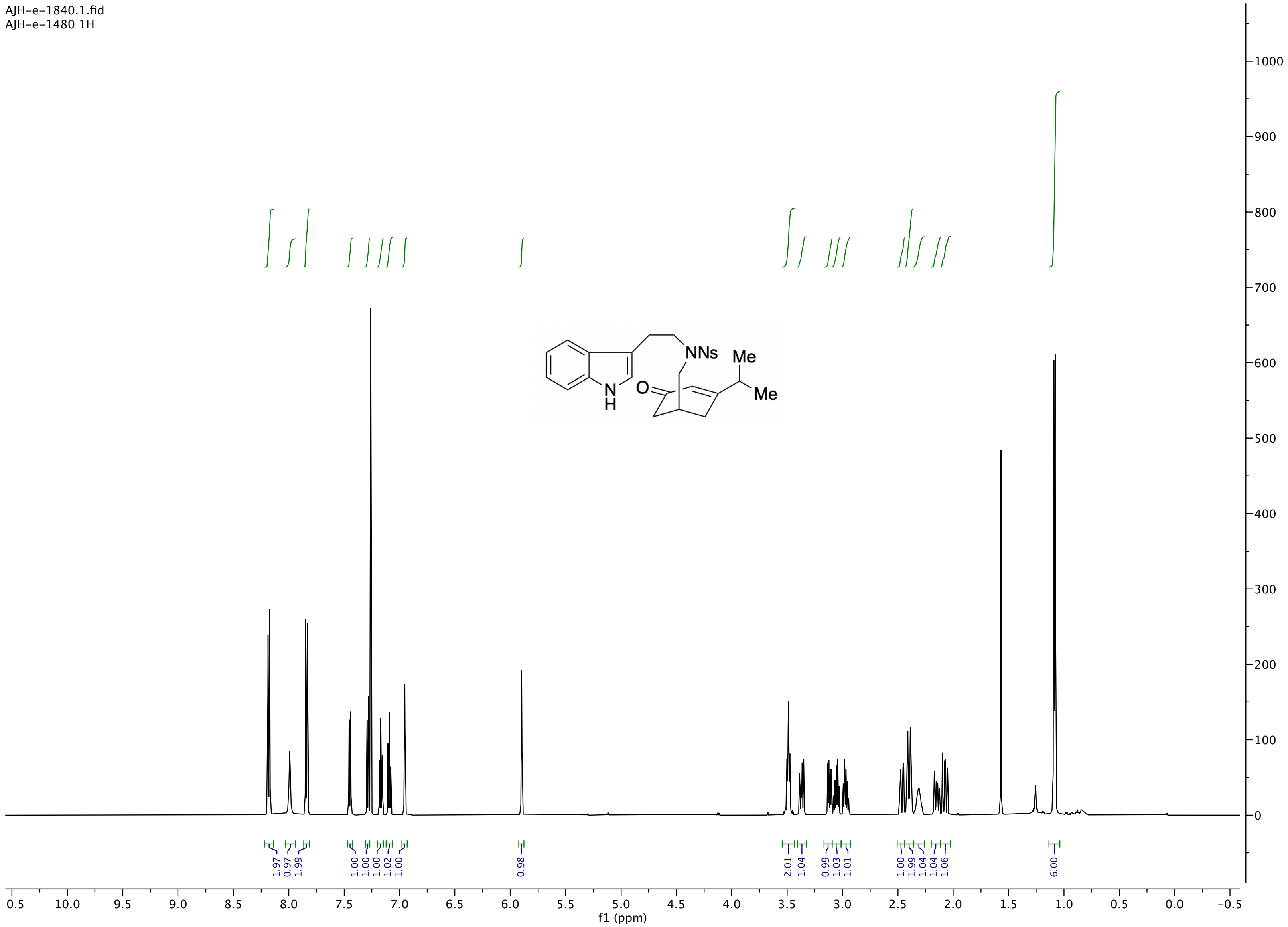

^{13}C NMR of (151 MHz, $CDCl_3$) of Compound 18h

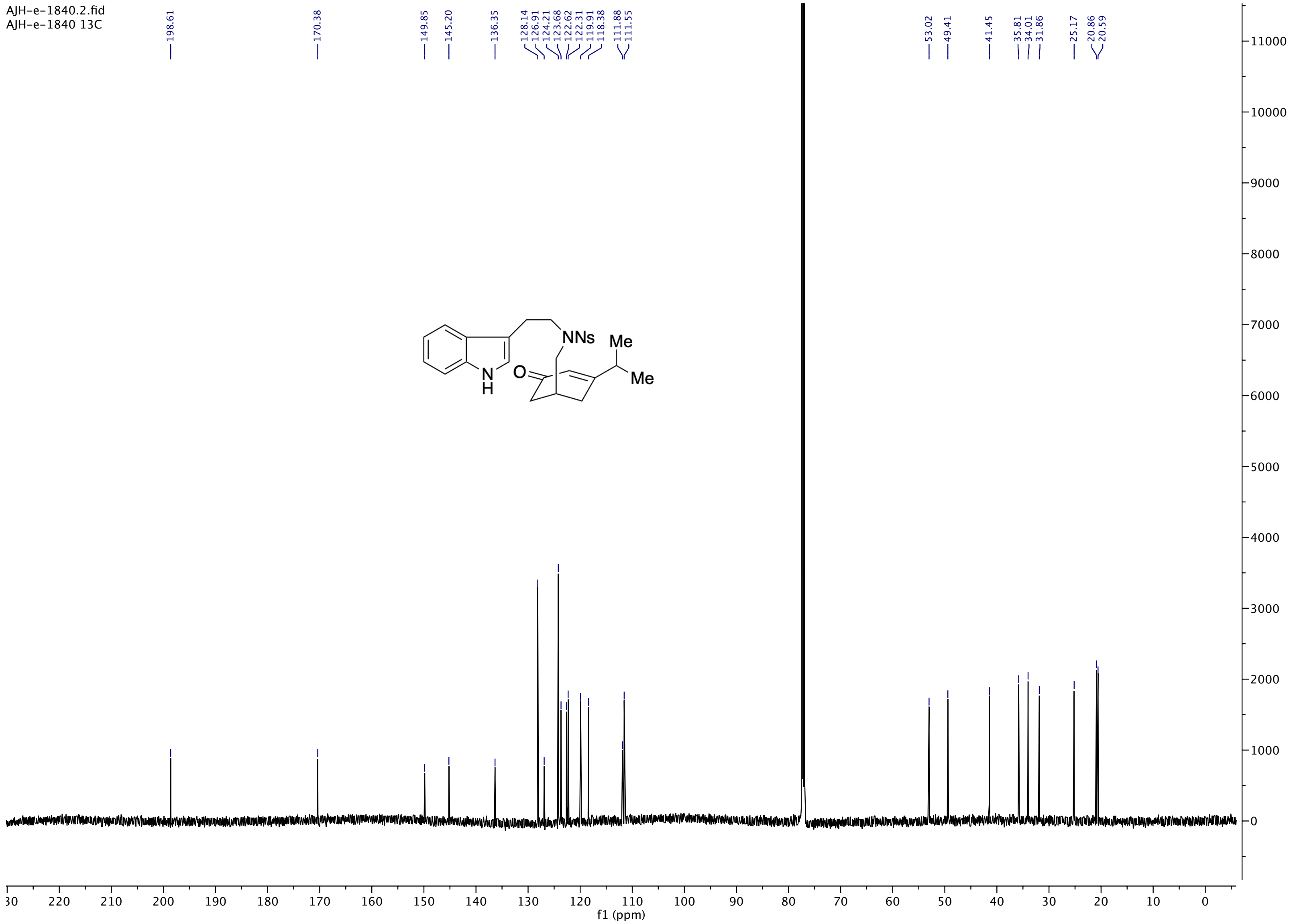

^{1}H NMR (600 MHz, $CDCl_3$) of Compound 18i

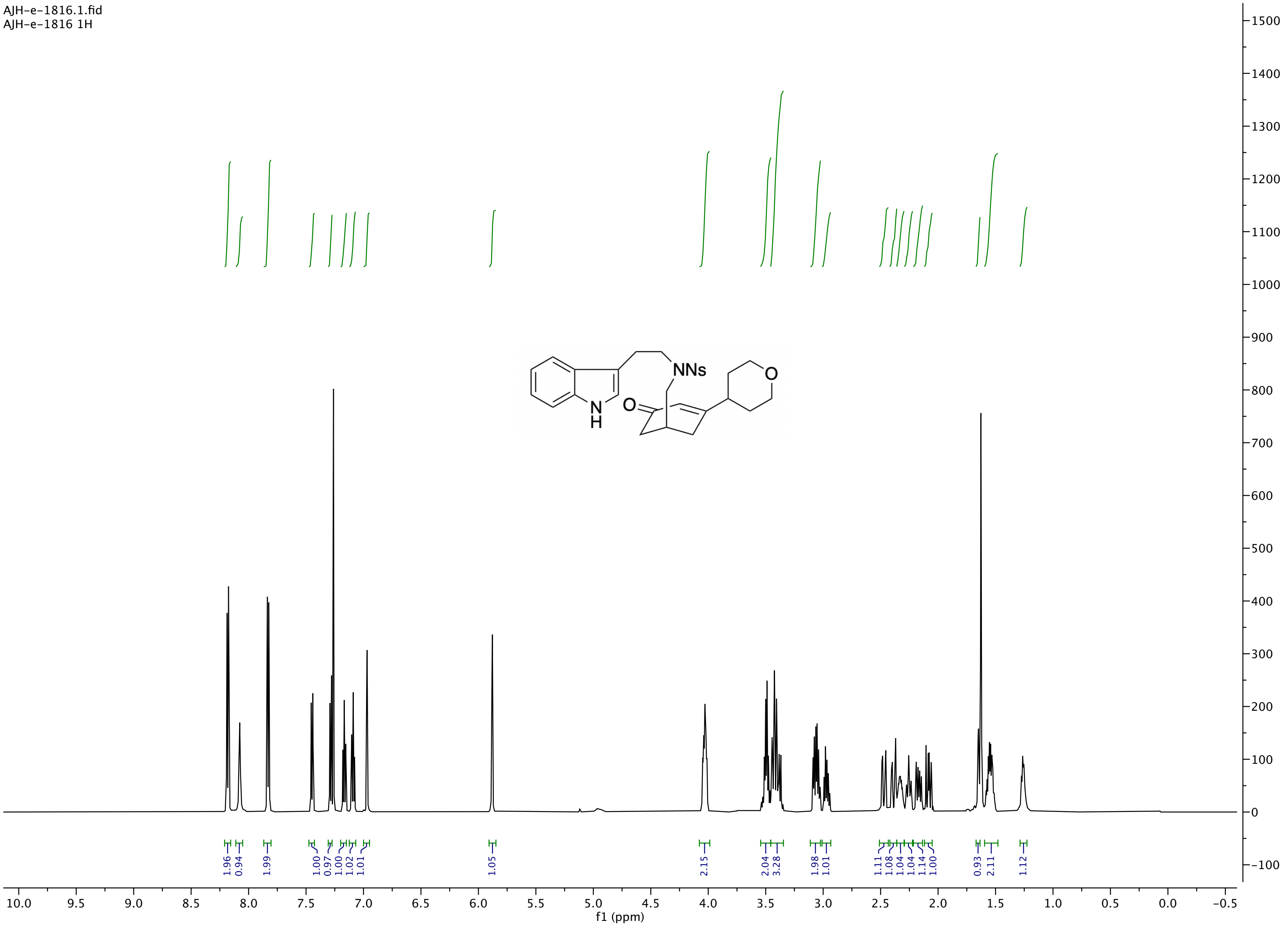

^{13}C NMR of (151 MHz, $CDCl_3$) of Compound 18i

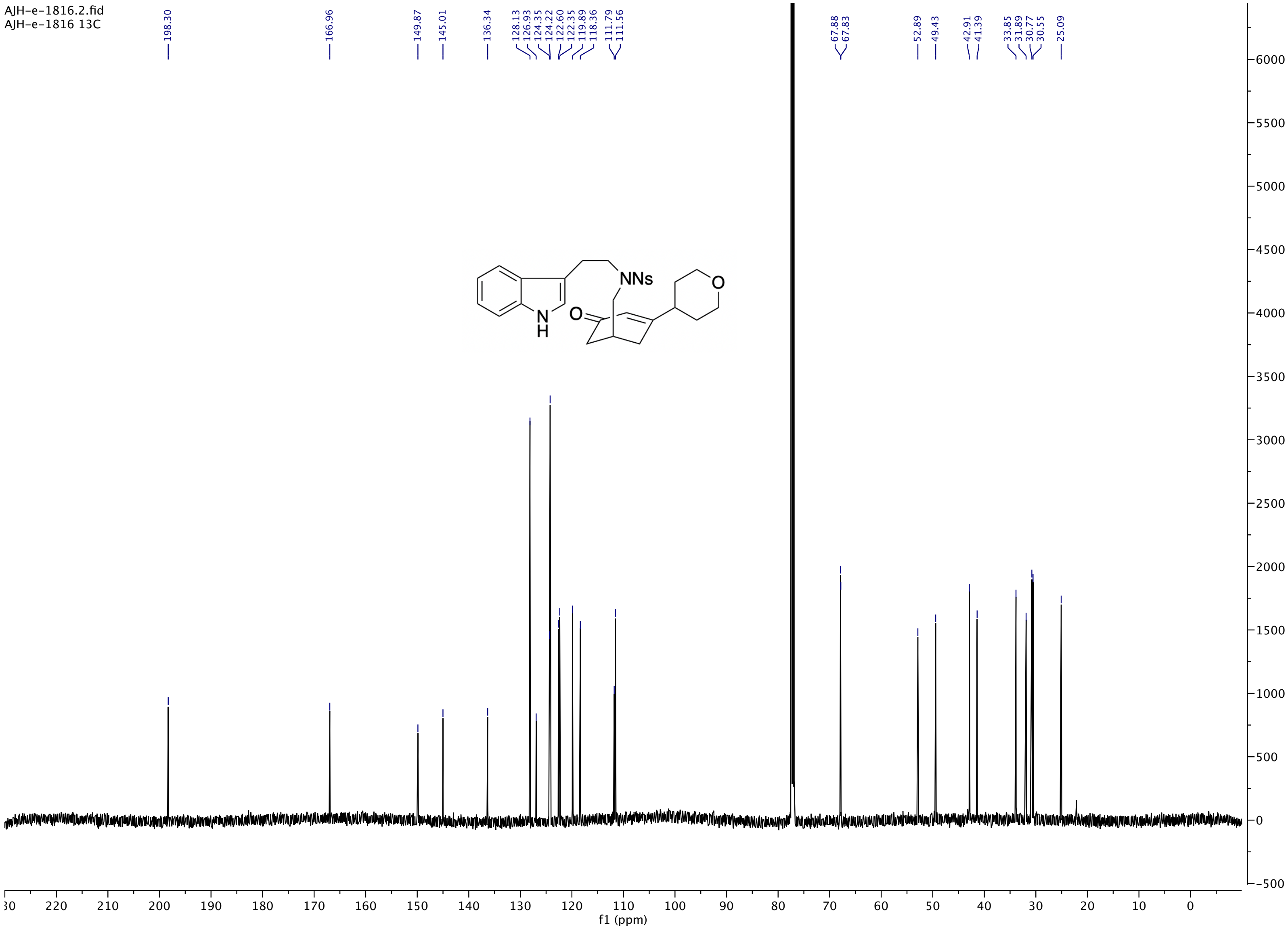

^{1}H NMR (600 MHz, $CDCl_3$) of Compound 8a

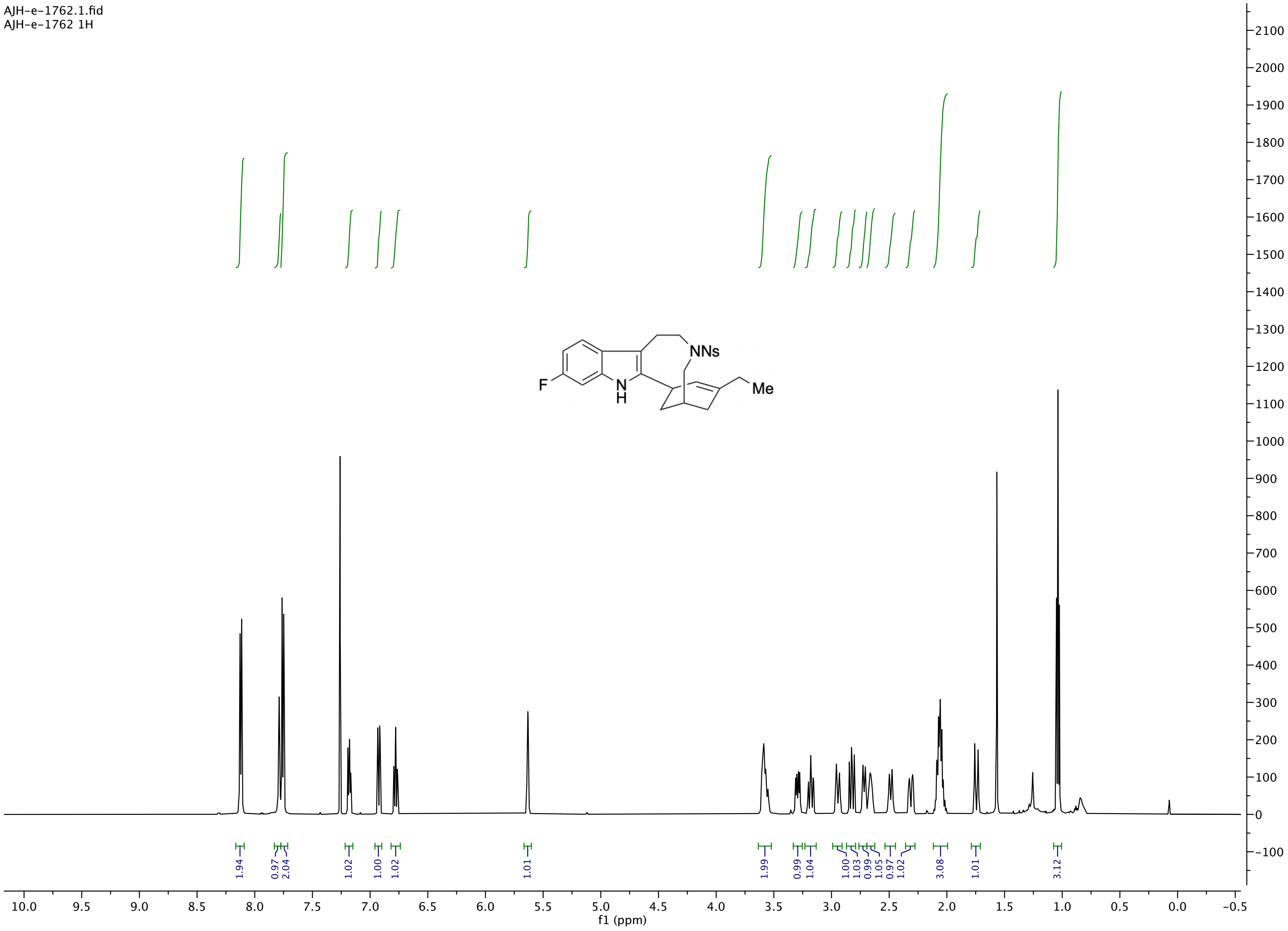

^{13}C NMR of (151 MHz, $CDCl_3$) of Compound 8a

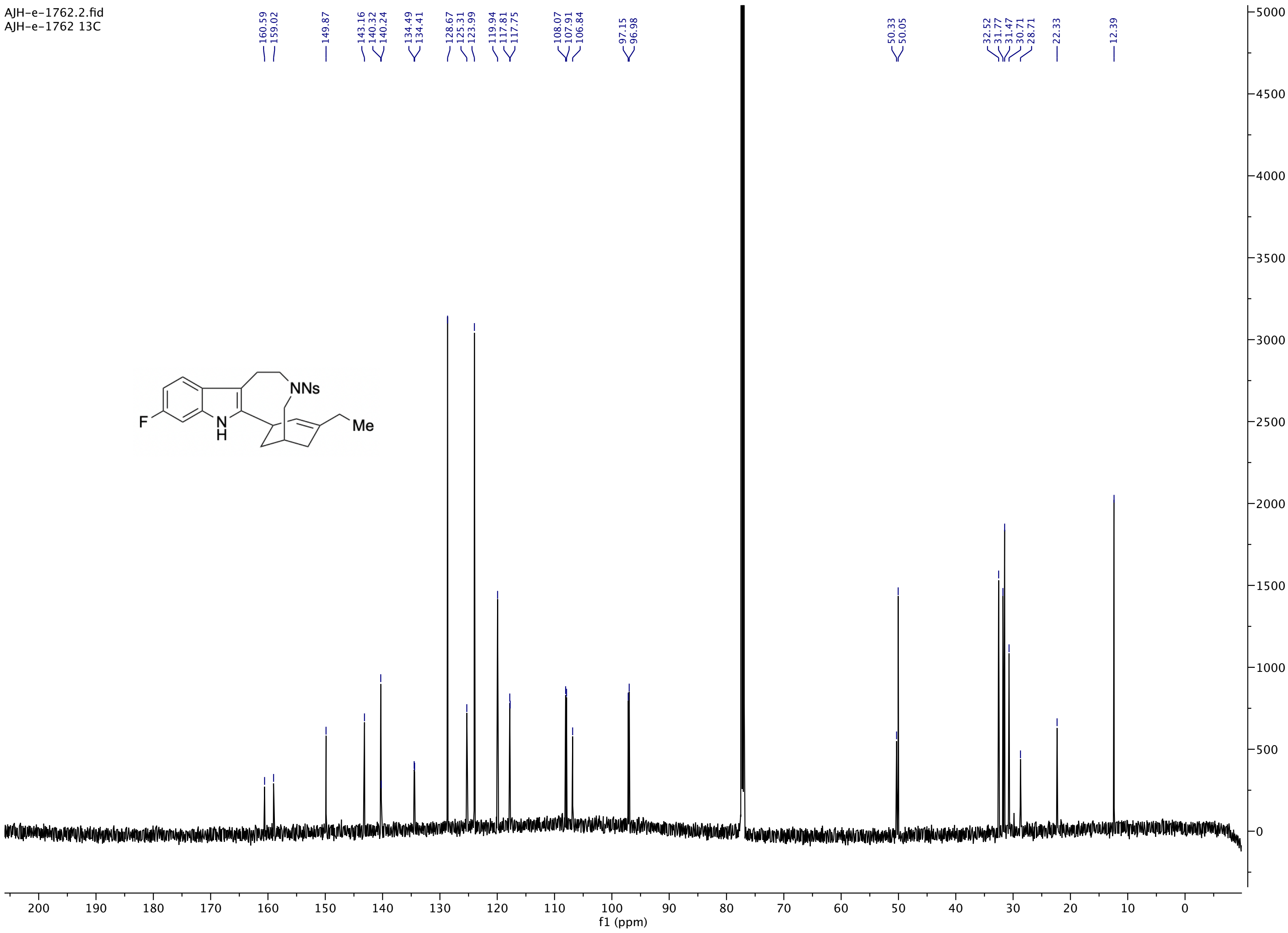

^{1}H NMR (600 MHz, $CDCl_3$) of Compound 8b

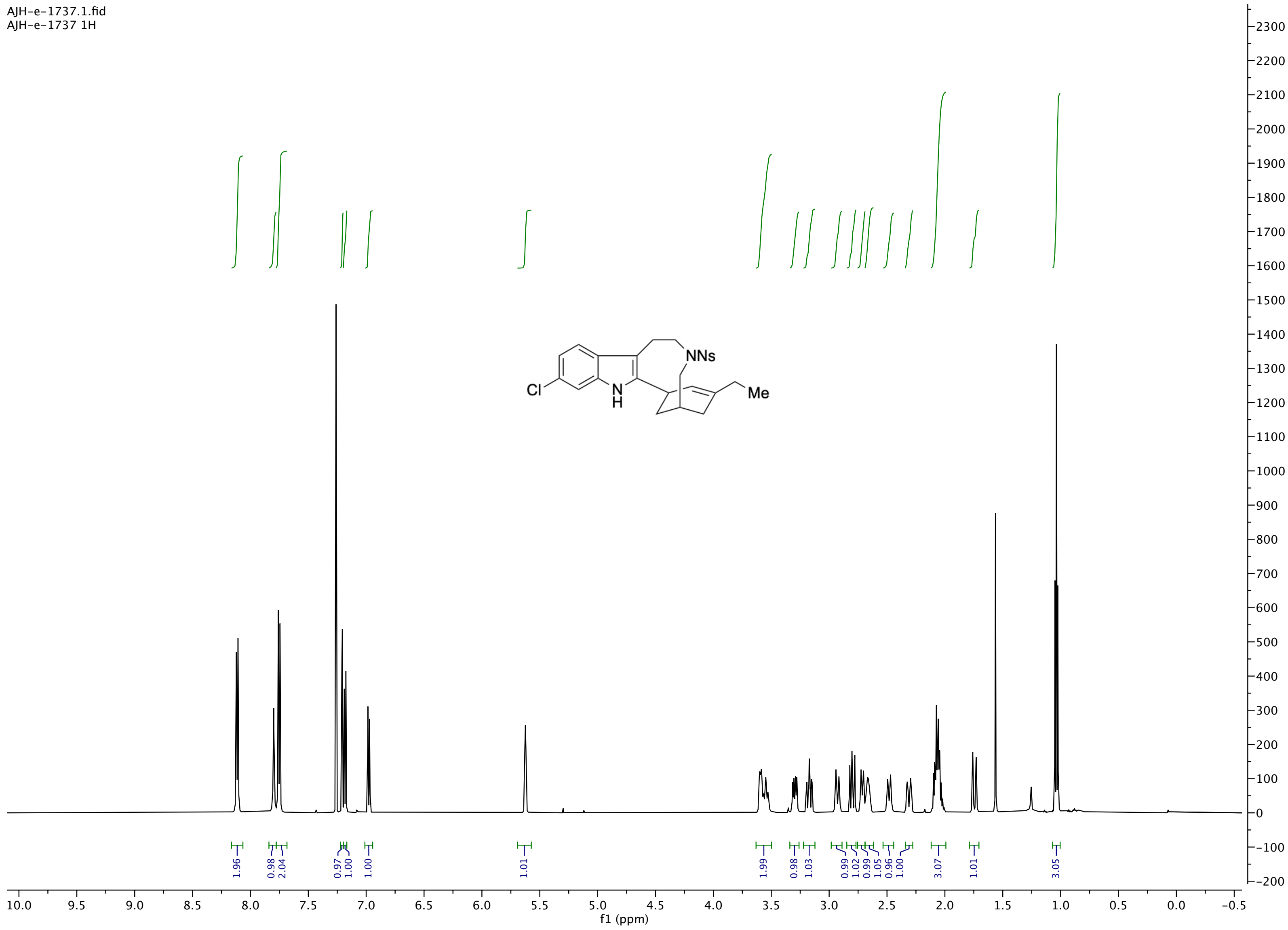

^{13}C NMR of (151 MHz, $CDCl_3$) of Compound 8b

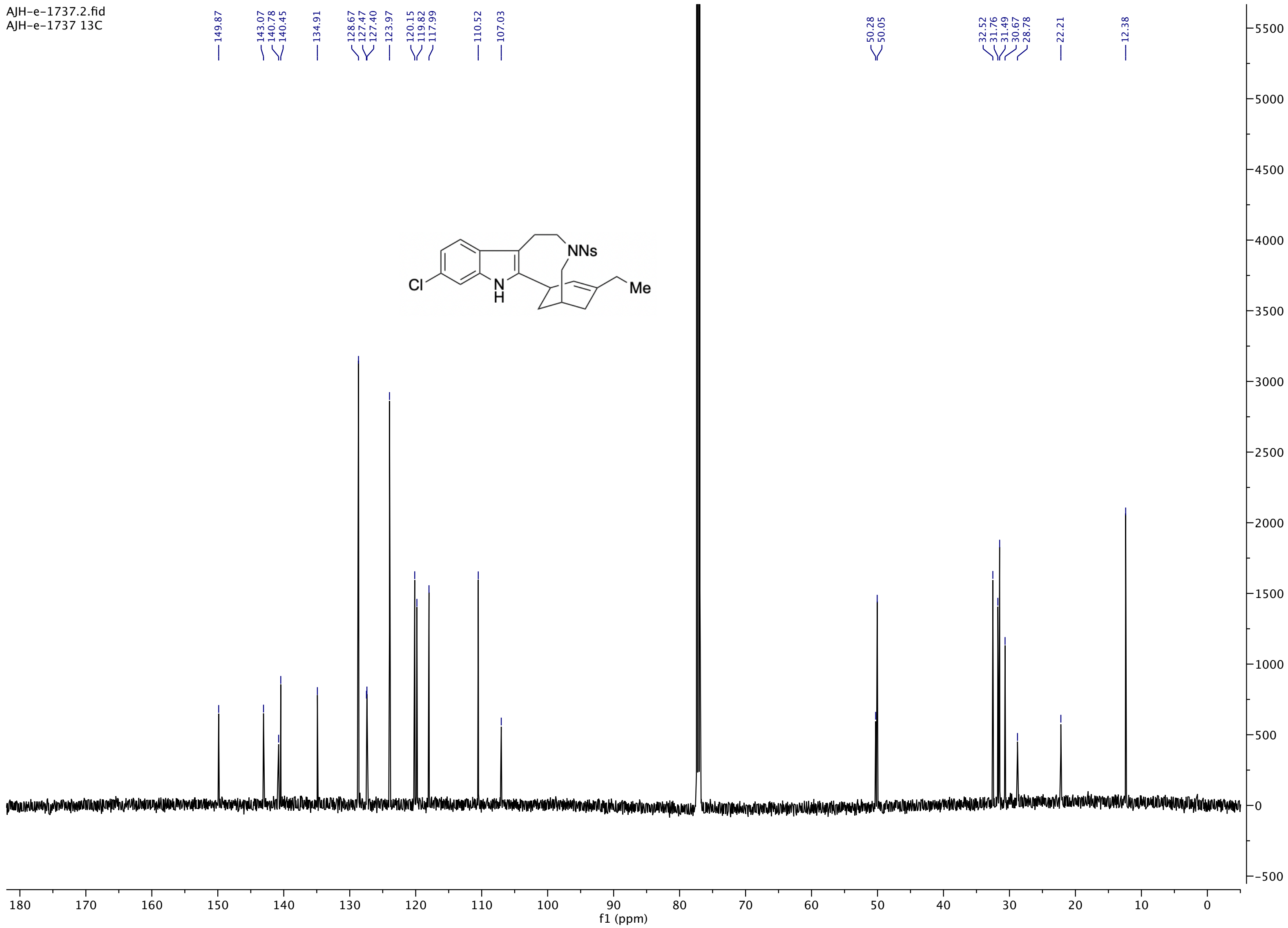

^{1}H NMR (600 MHz, $CDCl_3$) of Compound 8c

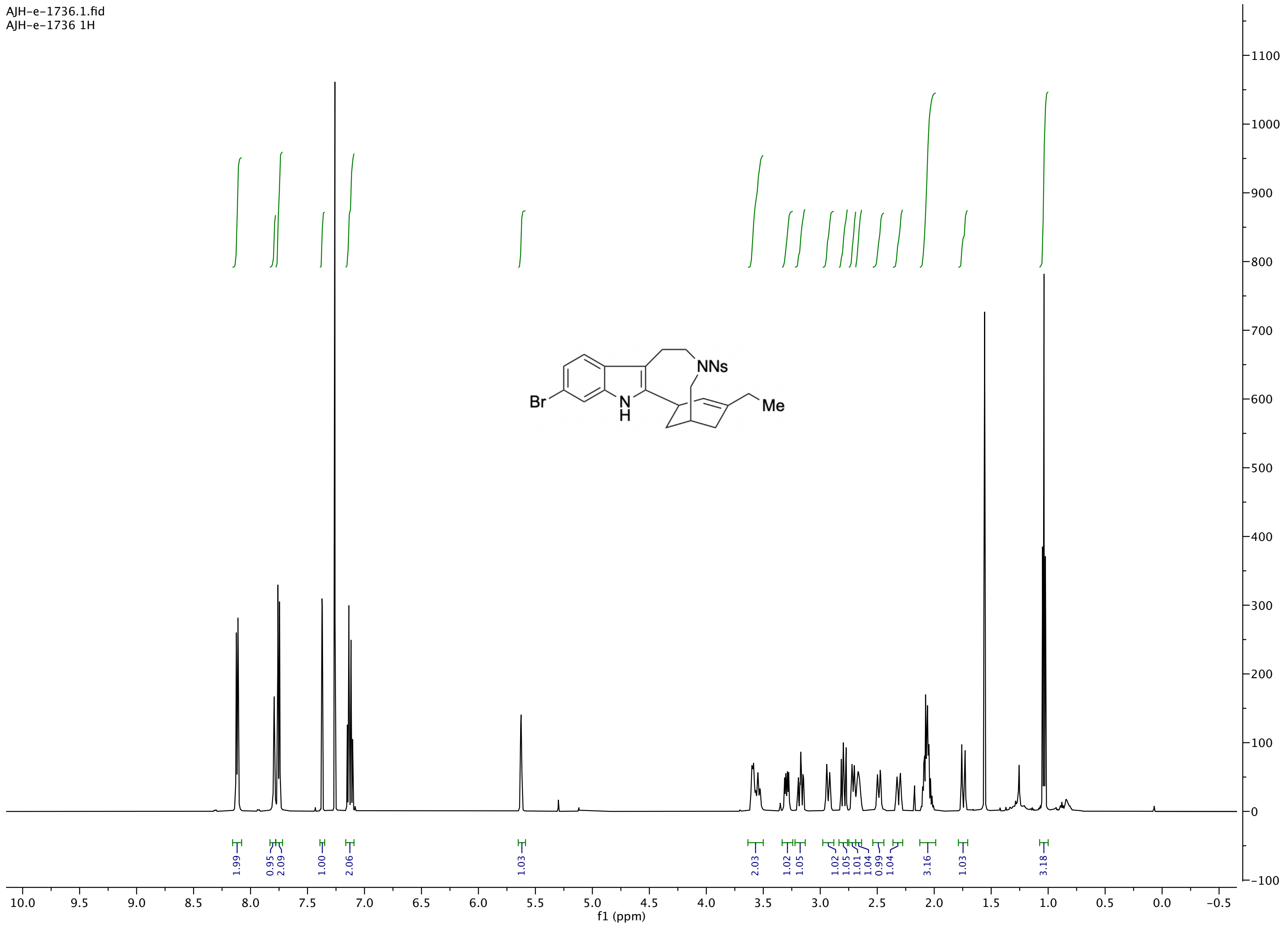

^{13}C NMR of (151 MHz, $CDCl_3$) of Compound 8c

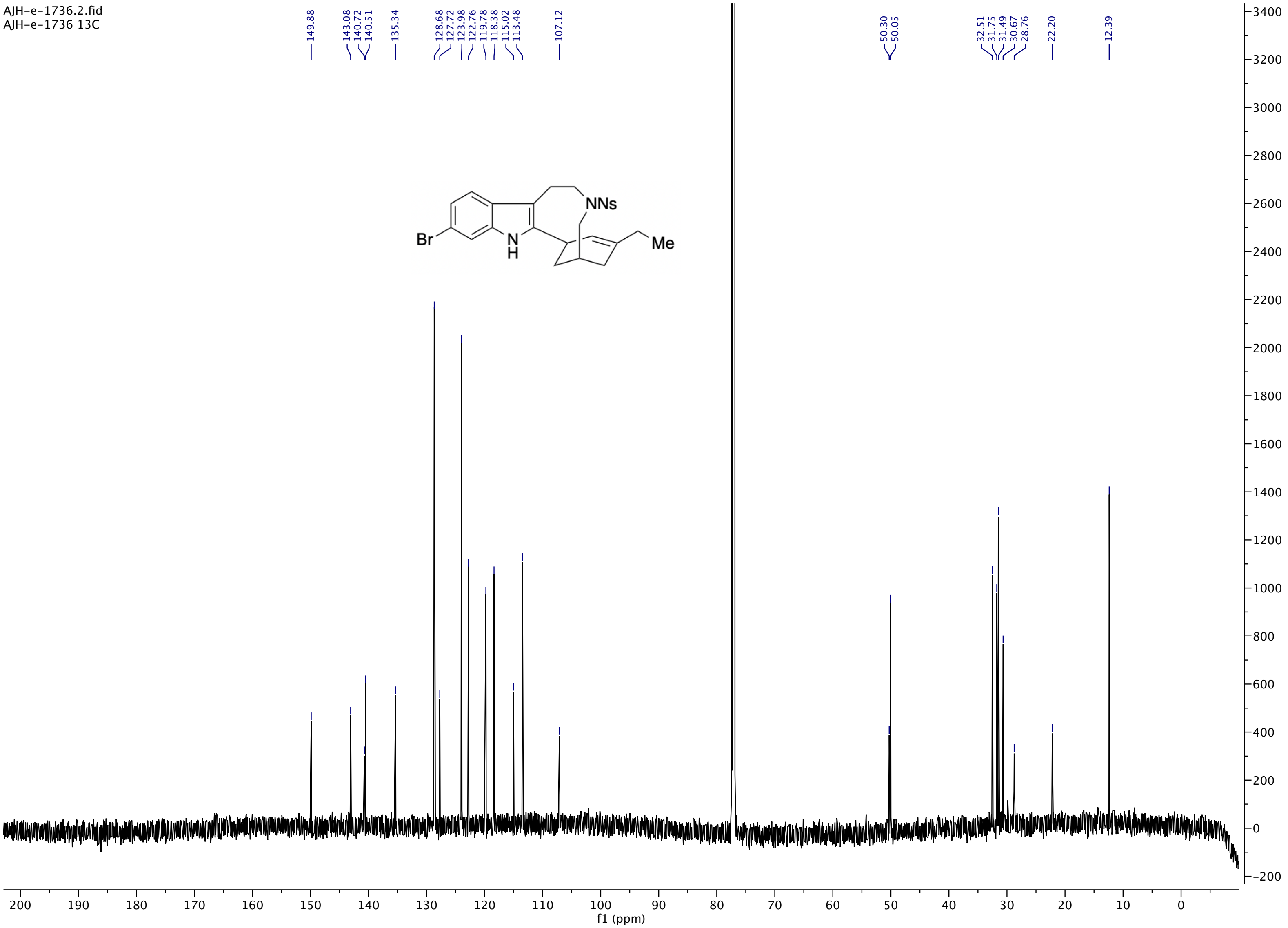

^{1}H NMR (600 MHz, $CDCl_3$) of Compound 8d

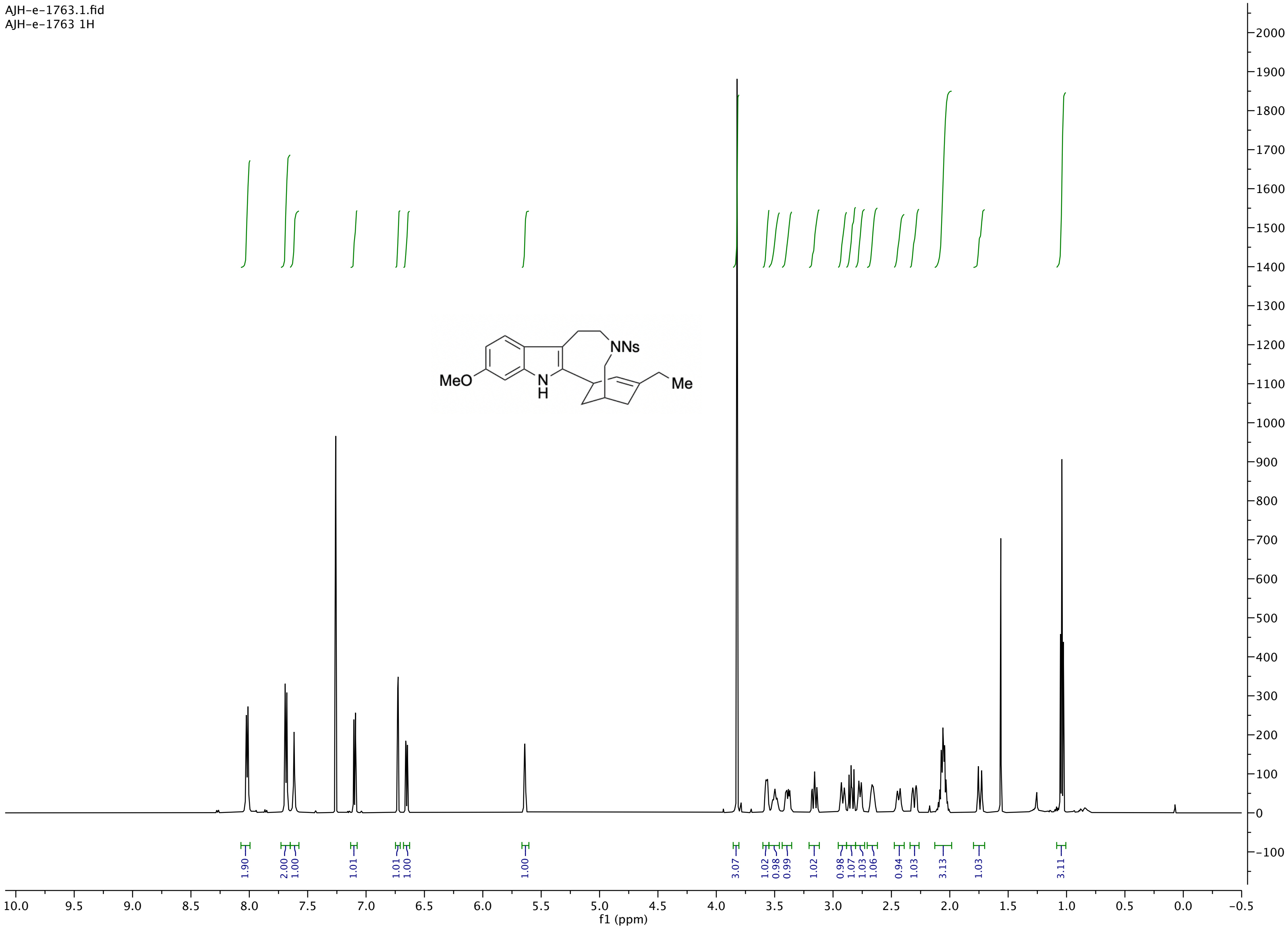

^{13}C NMR of (151 MHz, $CDCl_3$) of Compound 8d

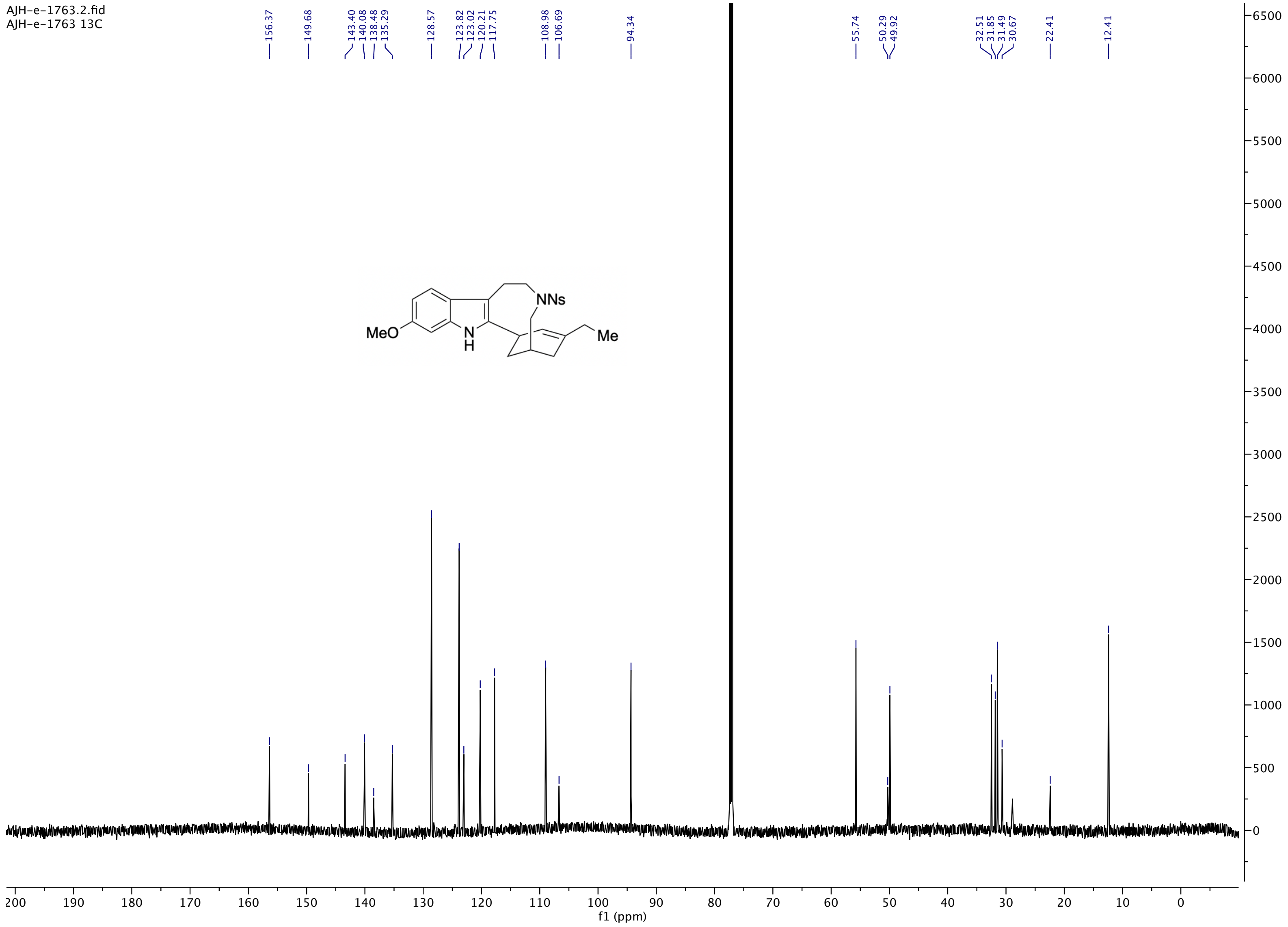

^{1}H NMR (600 MHz, $CDCl_3$) of Compound 8e

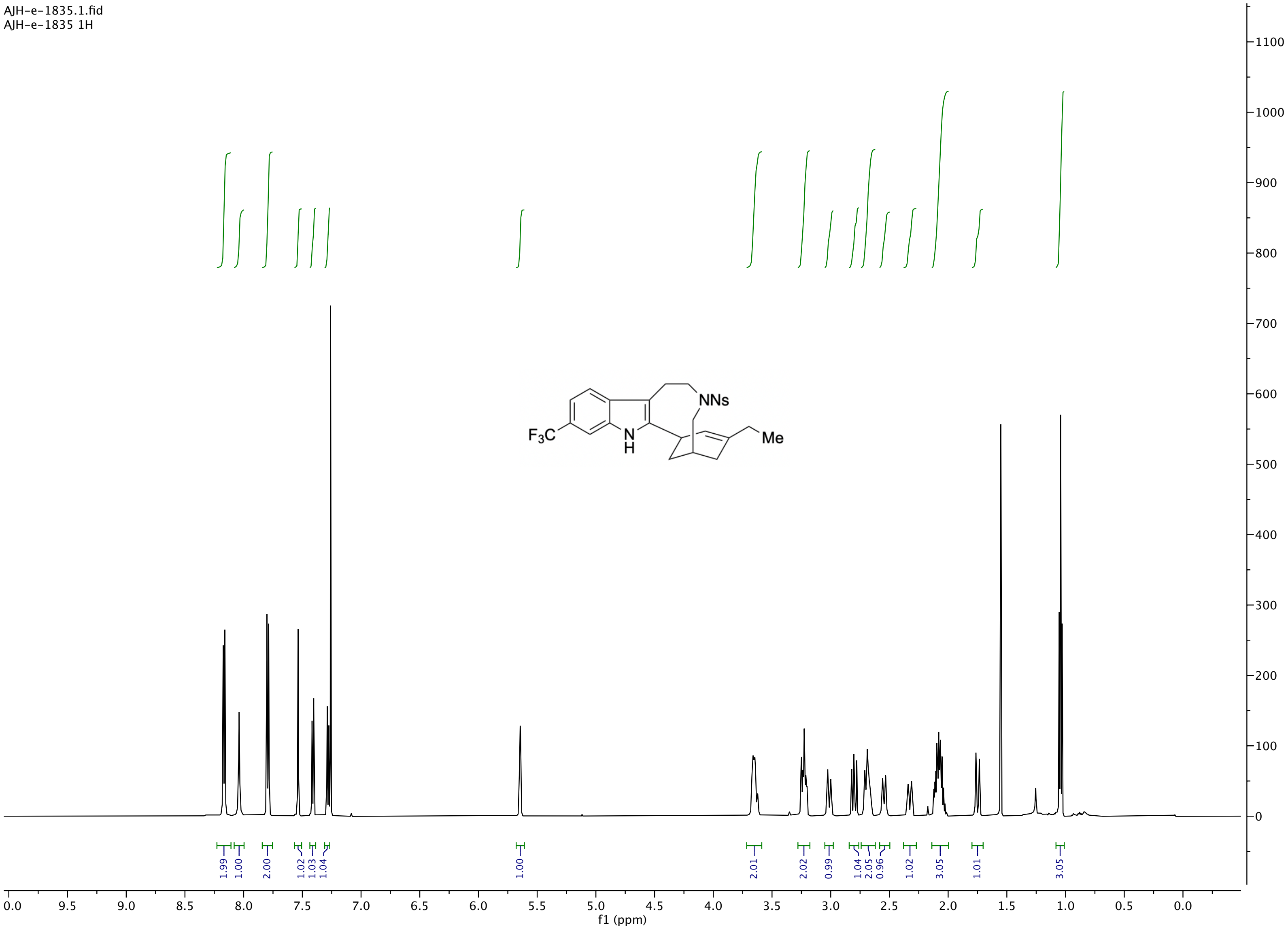

^{13}C NMR of (151 MHz, $CDCl_3$) of Compound 8e

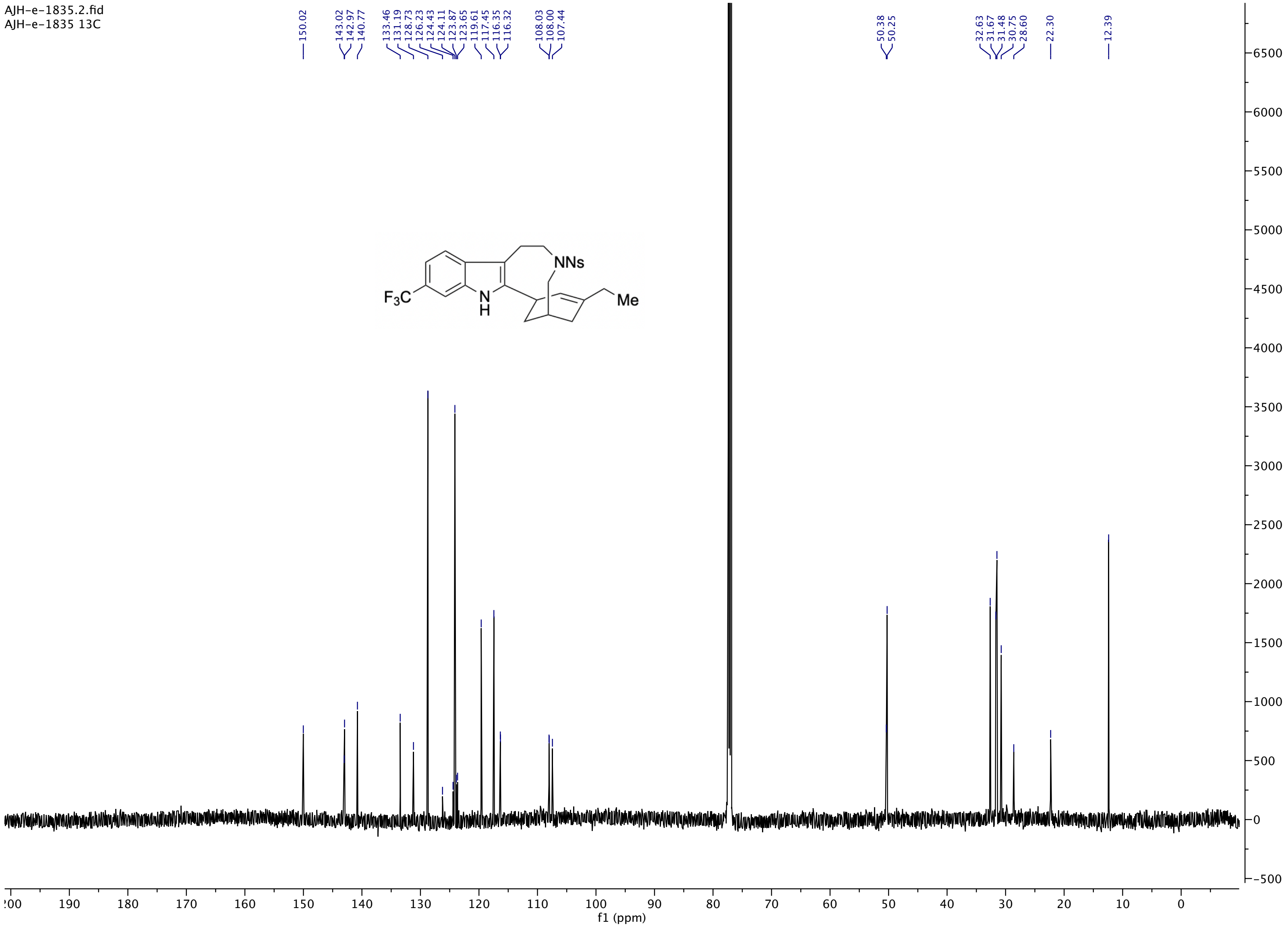

^{1}H NMR (600 MHz, CDCl$_3$) of Compound 8f

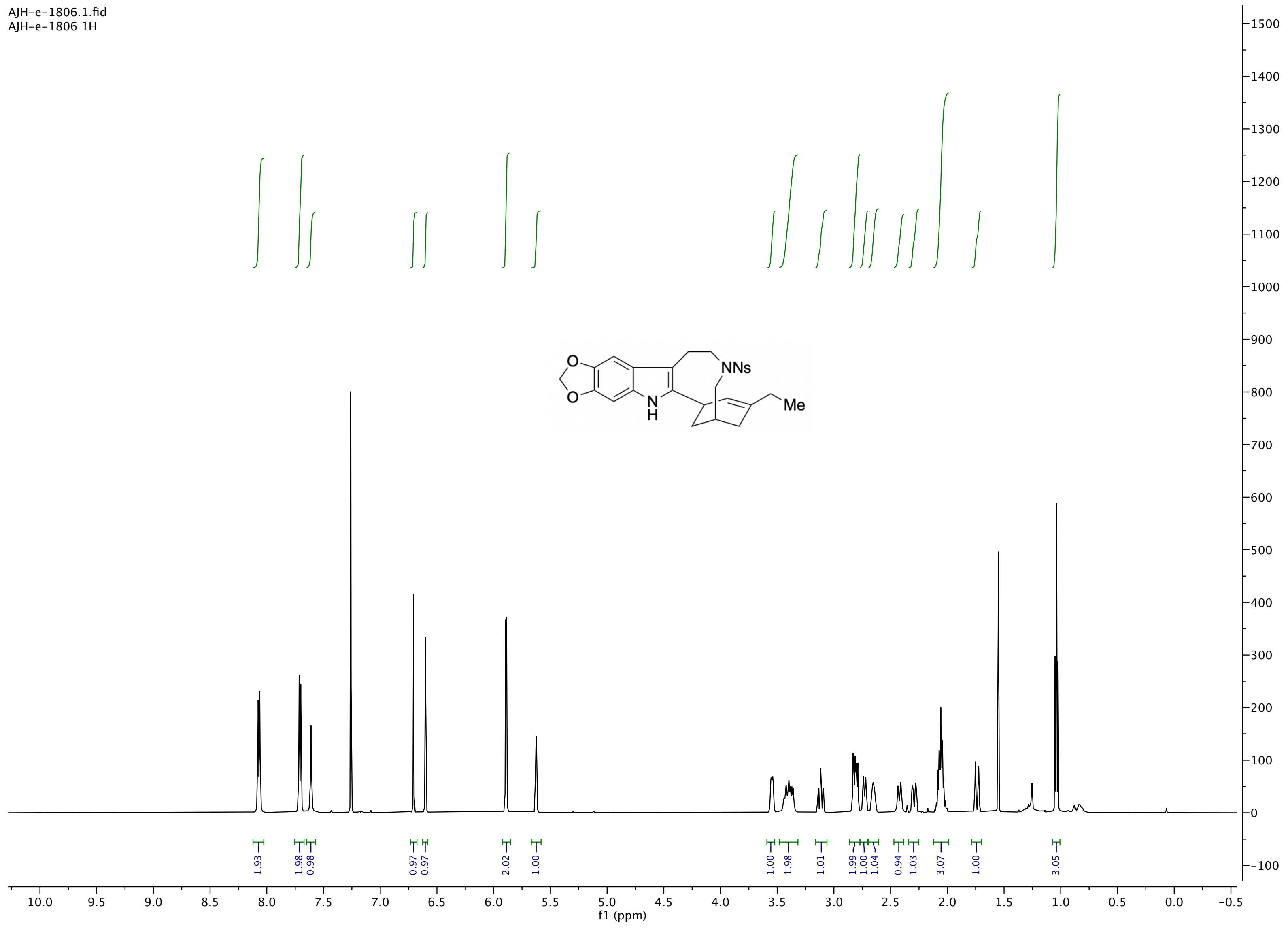

^{13}C NMR of (151 MHz, $CDCl_3$) of Compound 8f

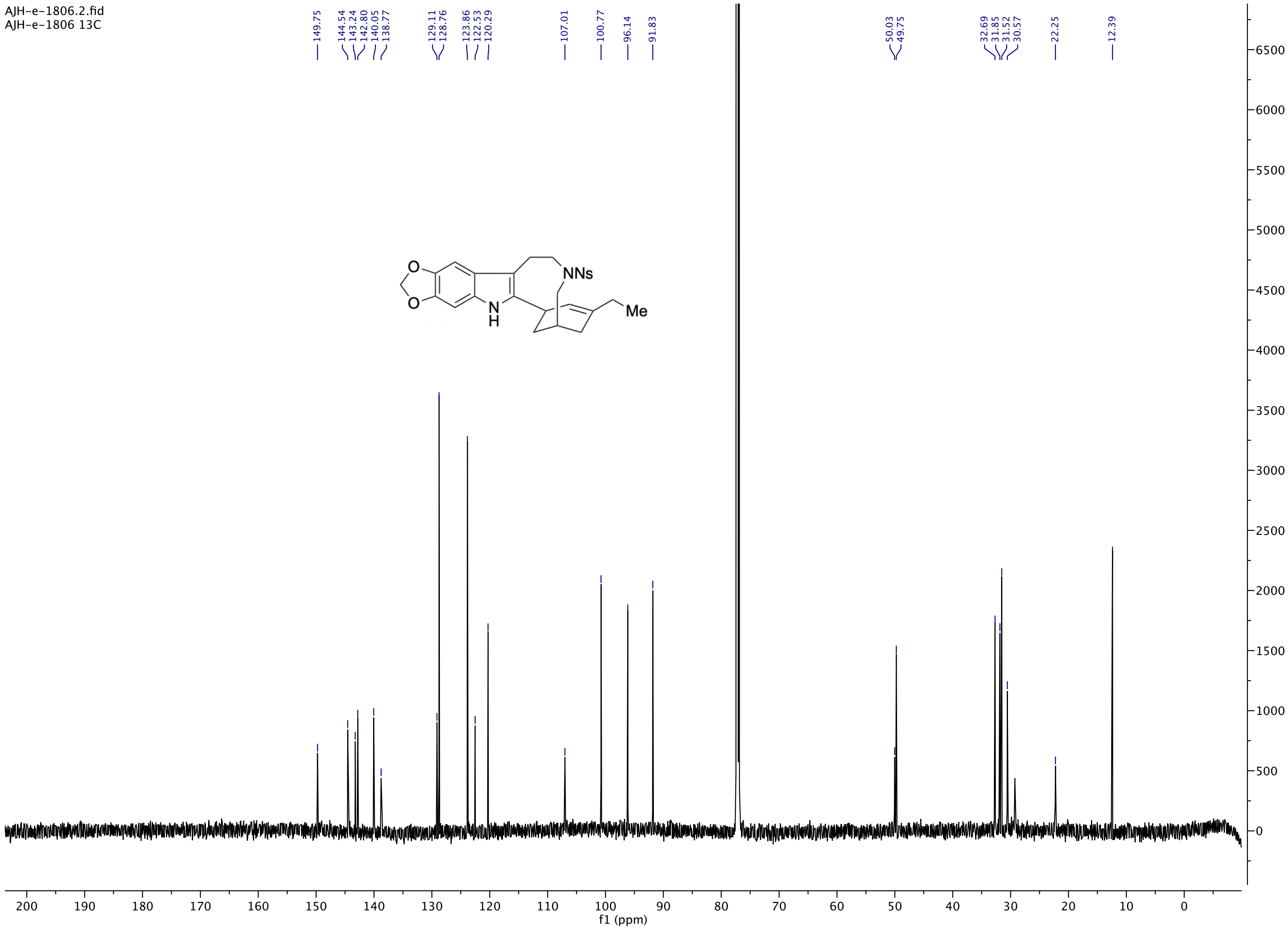

^{1}H NMR (600 MHz, $CDCl_3$) of Compound 8g

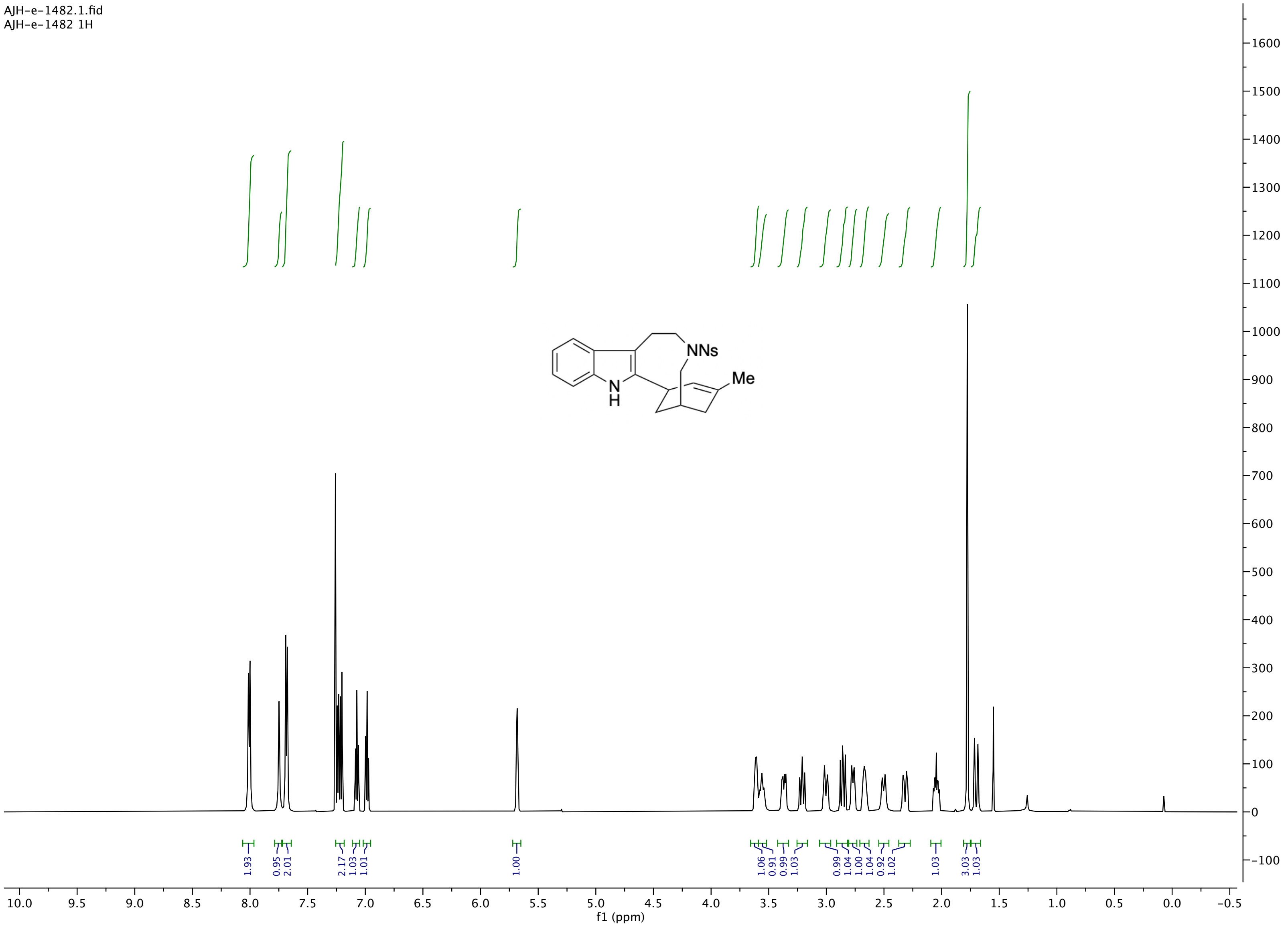

^{13}C NMR of (151 MHz, $CDCl_3$) of Compound 8g

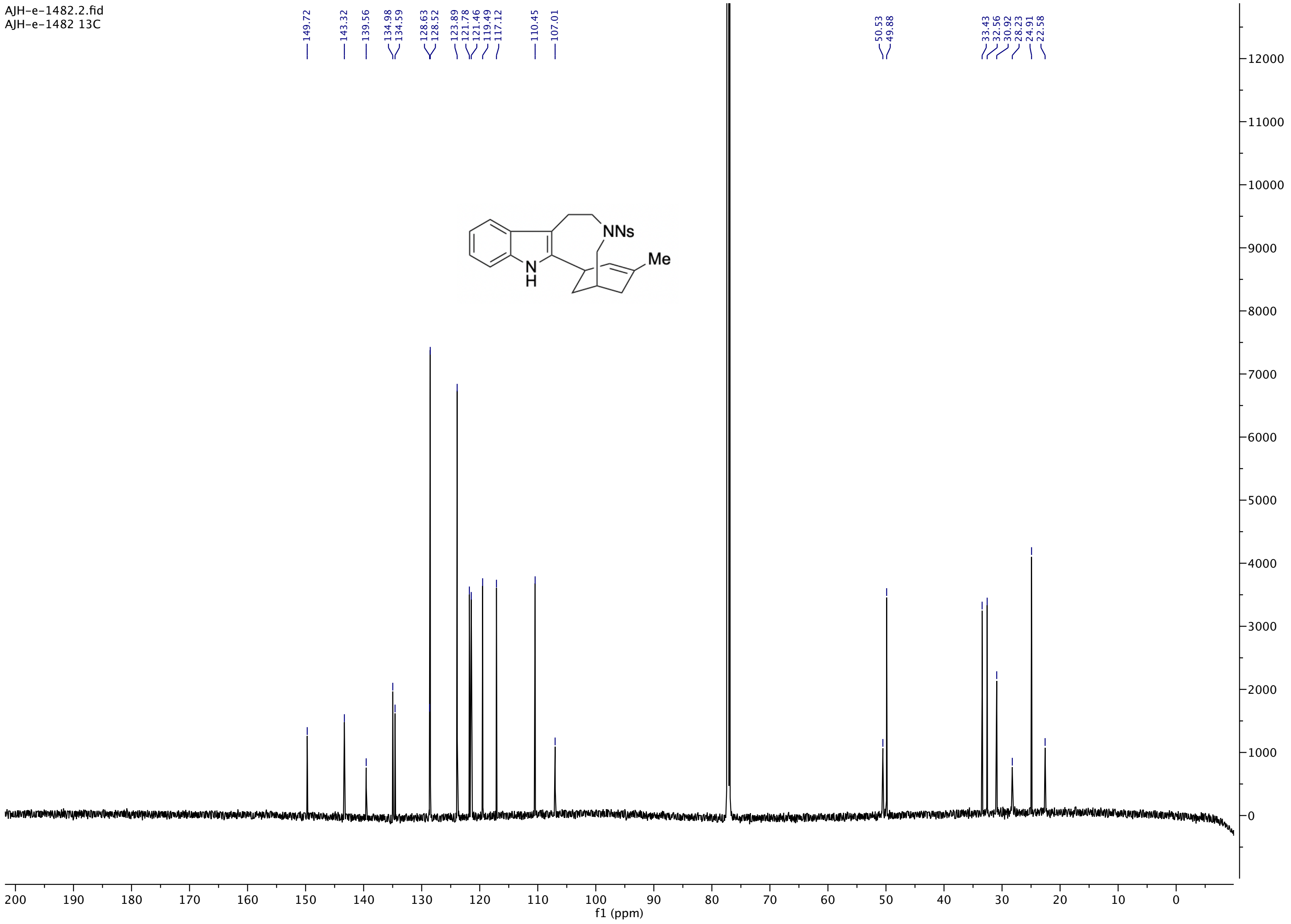

^{1}H NMR (600 MHz, CDCl3) of Compound 8h

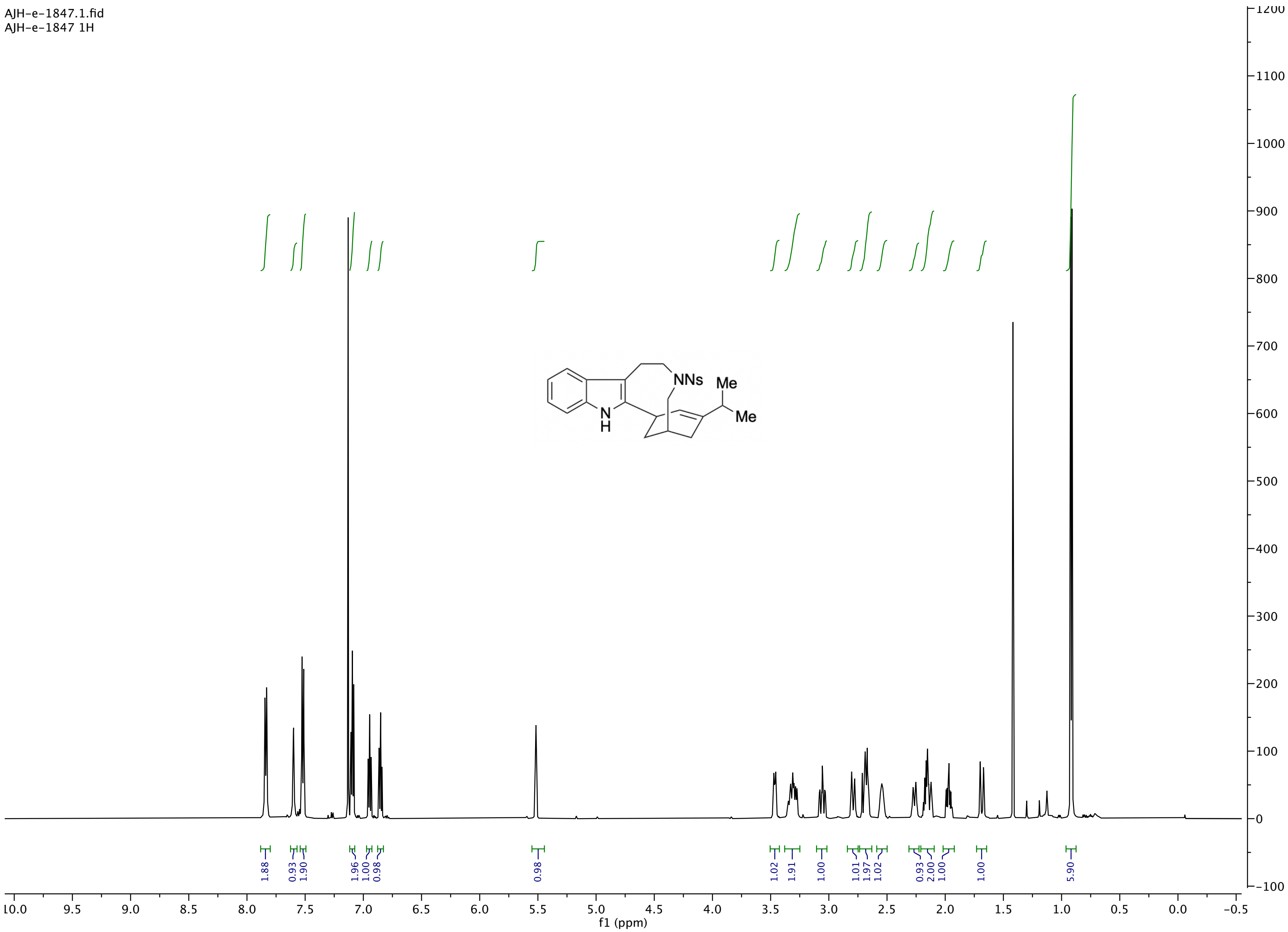

^{13}C NMR of (151 MHz, $CDCl_3$) of Compound 8h

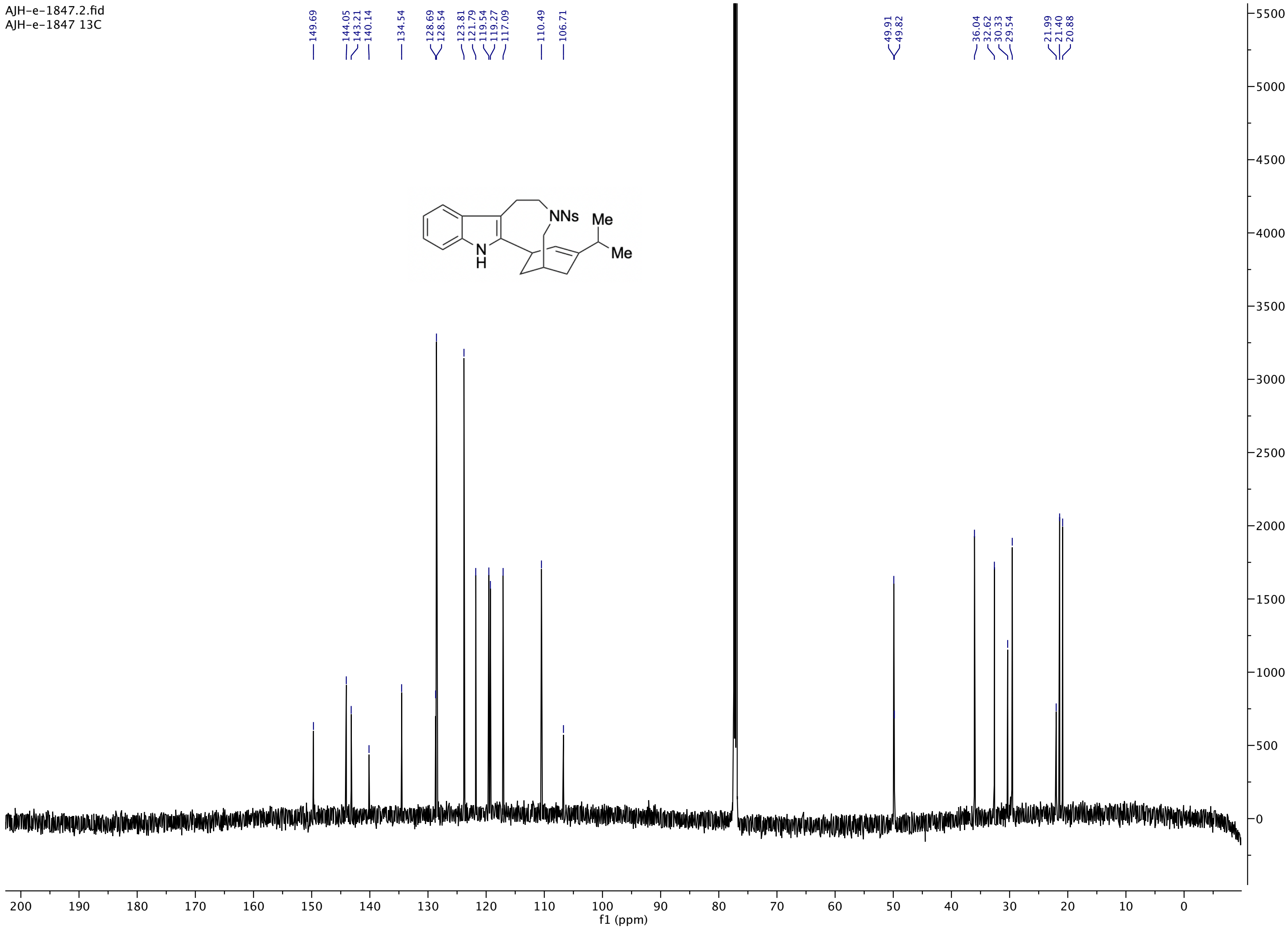

^{1}H NMR (600 MHz, $CDCl_3$) of Compound 8i

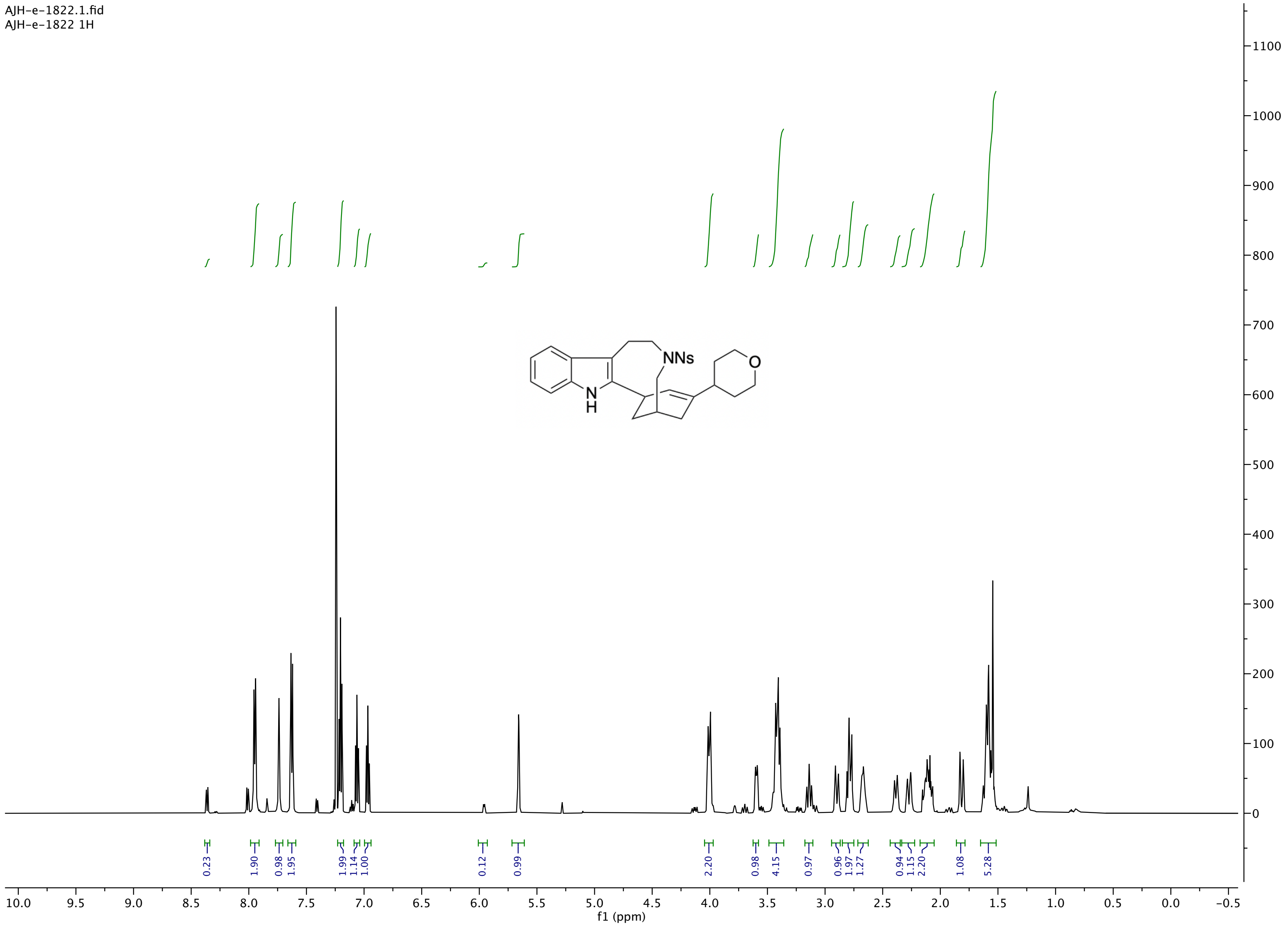

^{13}C NMR of (151 MHz, $CDCl_3$) of Compound 8i

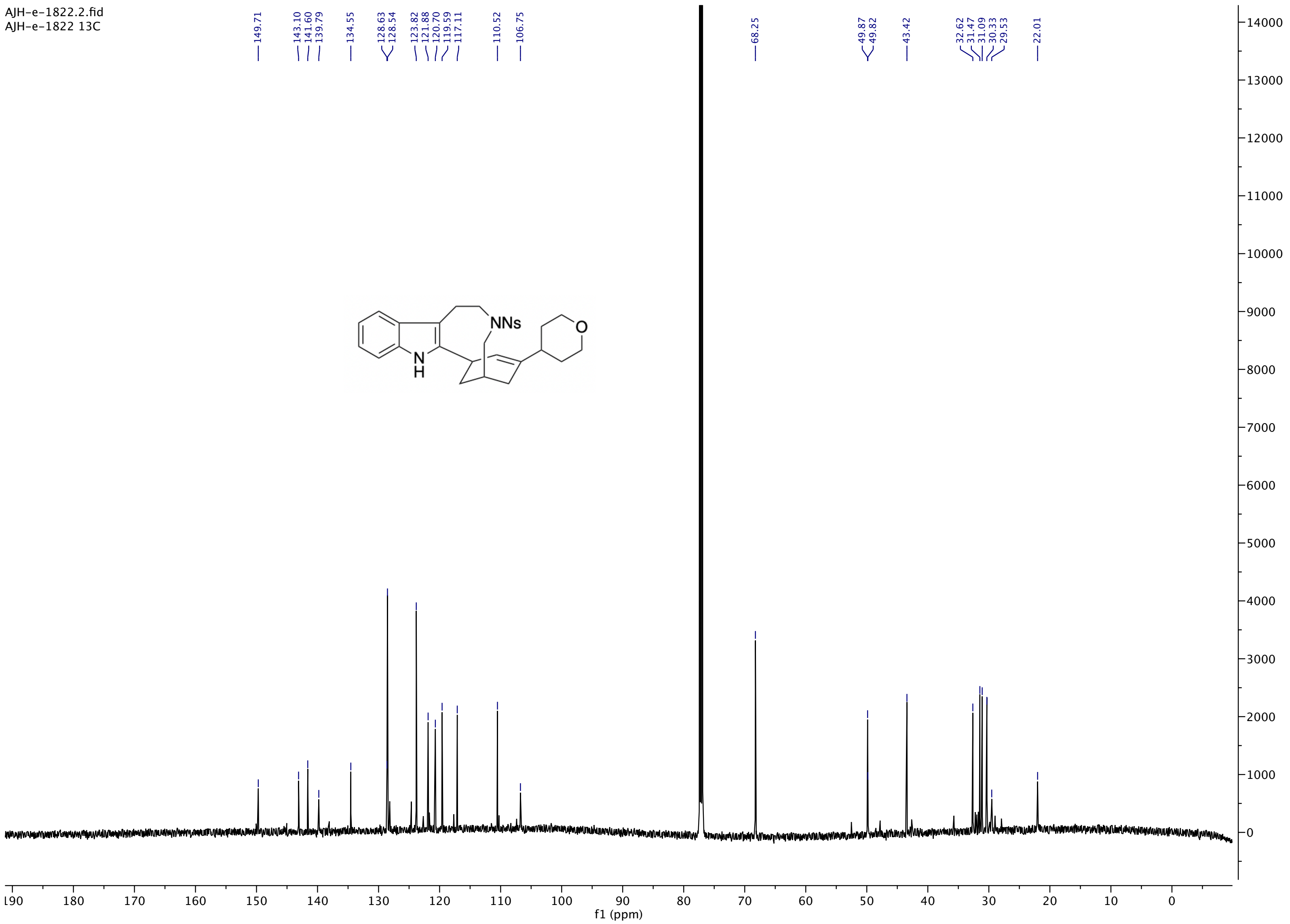

^{1}H NMR (600 MHz, $CDCl_3$) of Compound 7a

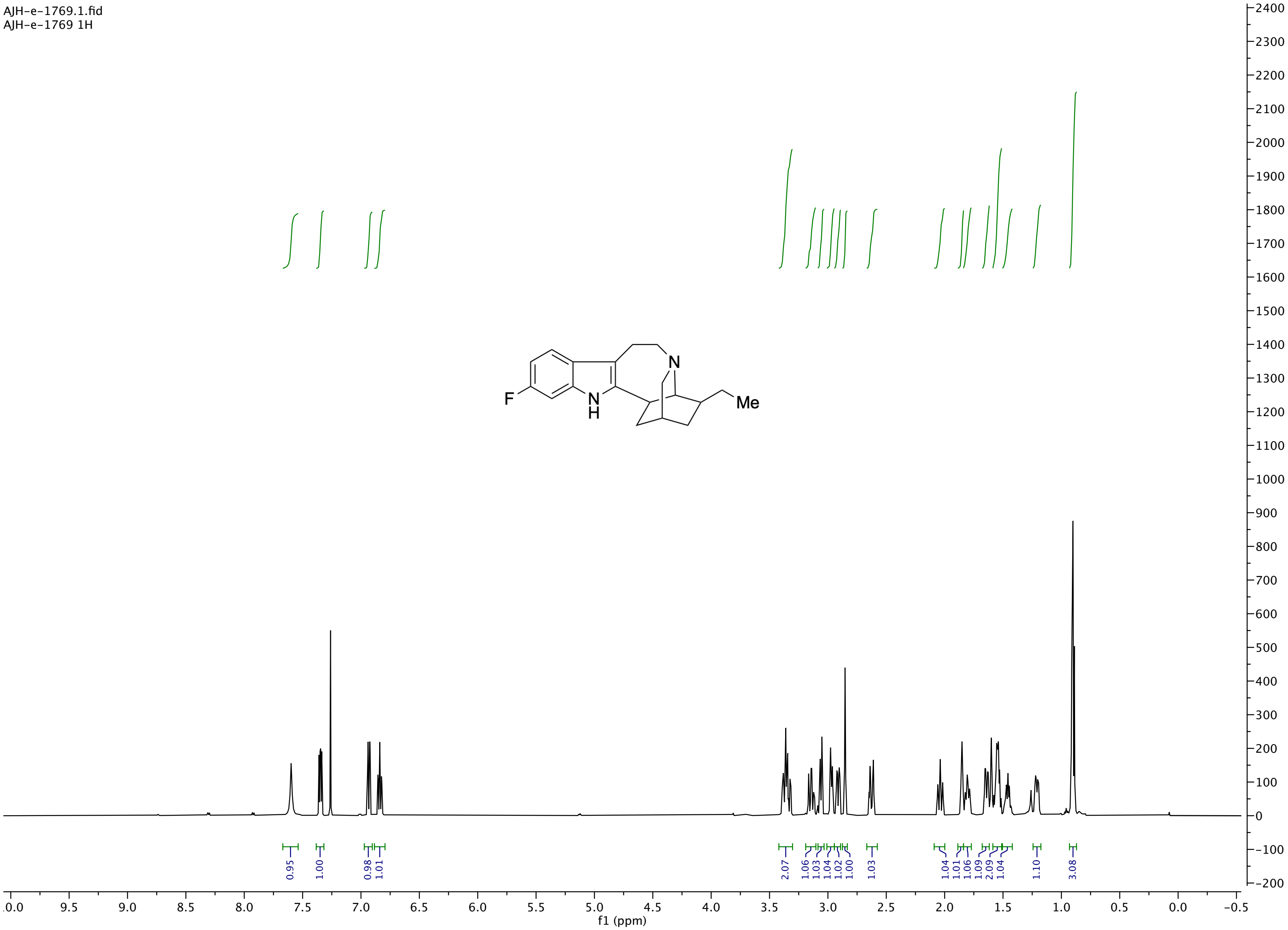

^{13}C NMR of (151 MHz, $CDCl_3$) of Compound 7a

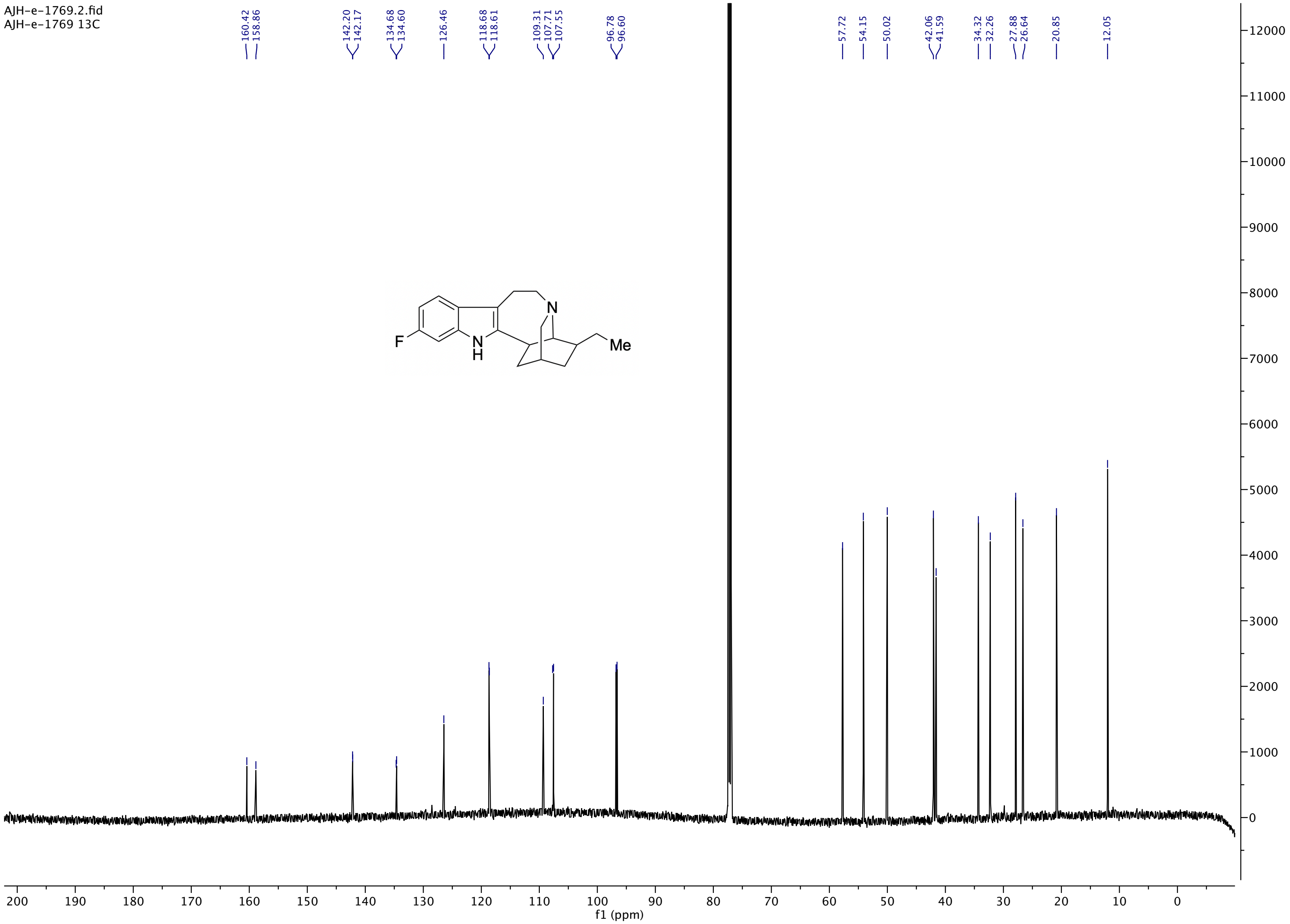

^{19}F NMR (471 MHz, $CDCl_3$) of 7a

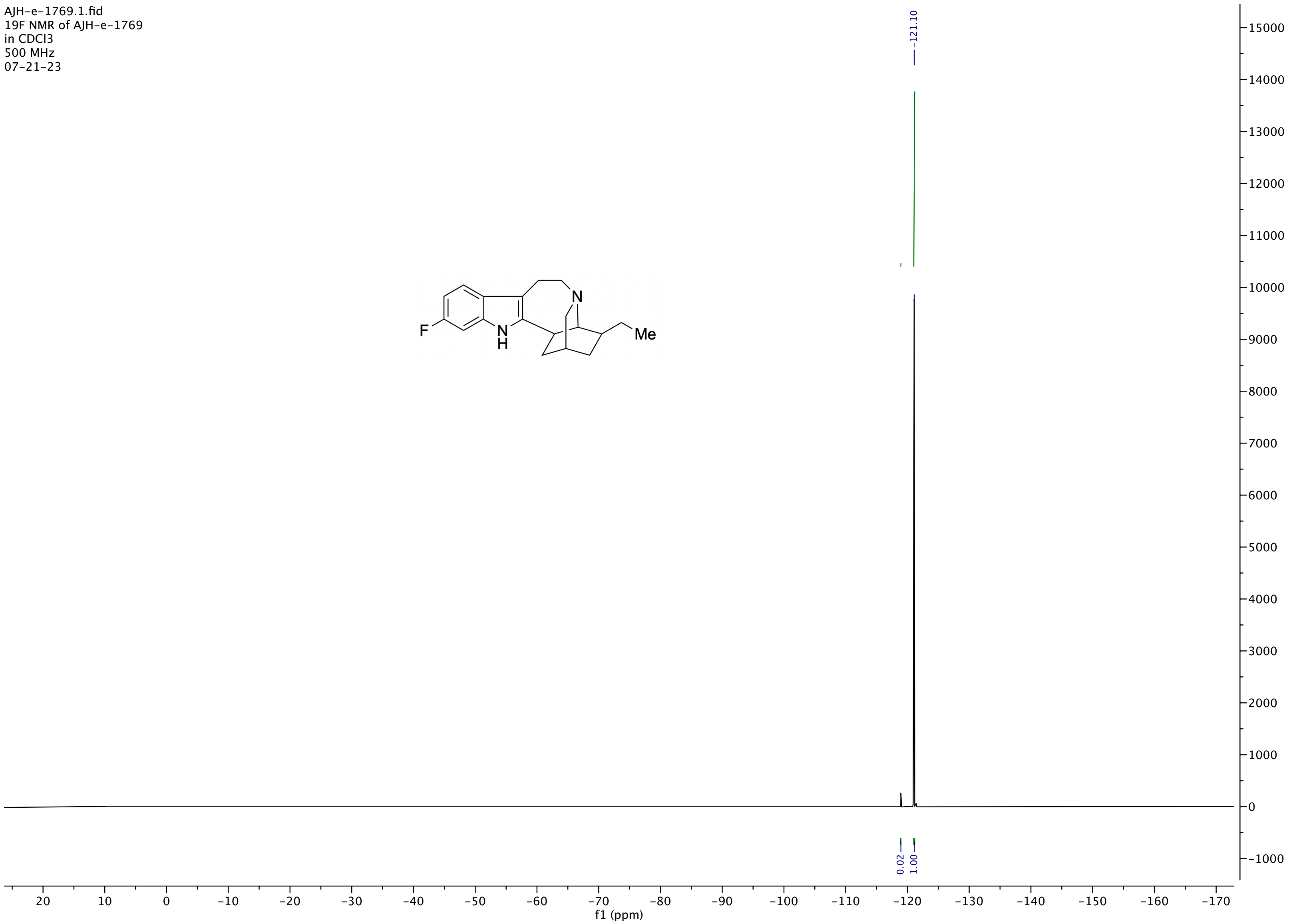

^{1}H NMR (600 MHz, CDCl$_3$) of Compound 7b

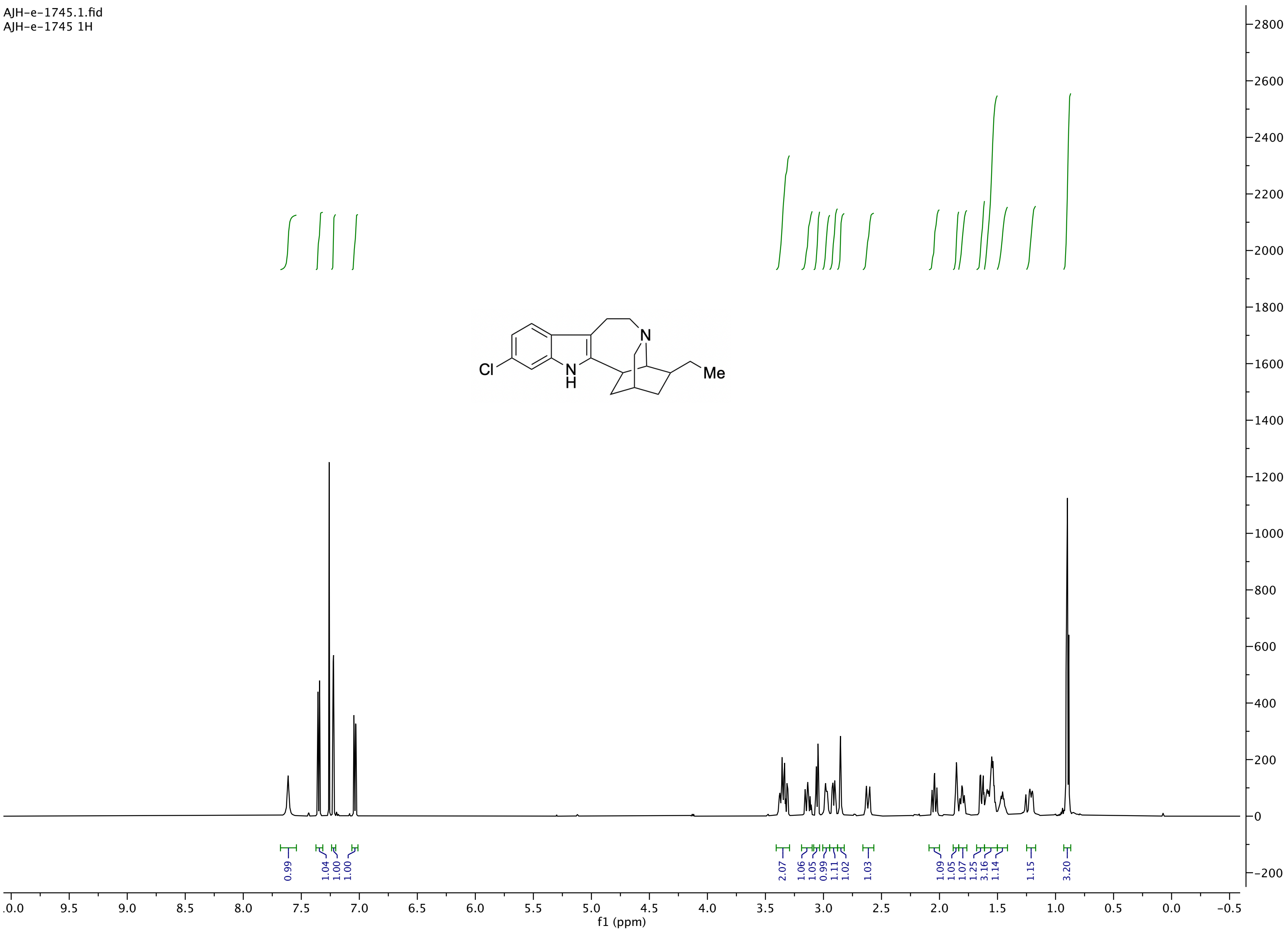

^{13}C NMR of (151 MHz, $CDCl_3$) of Compound 7b

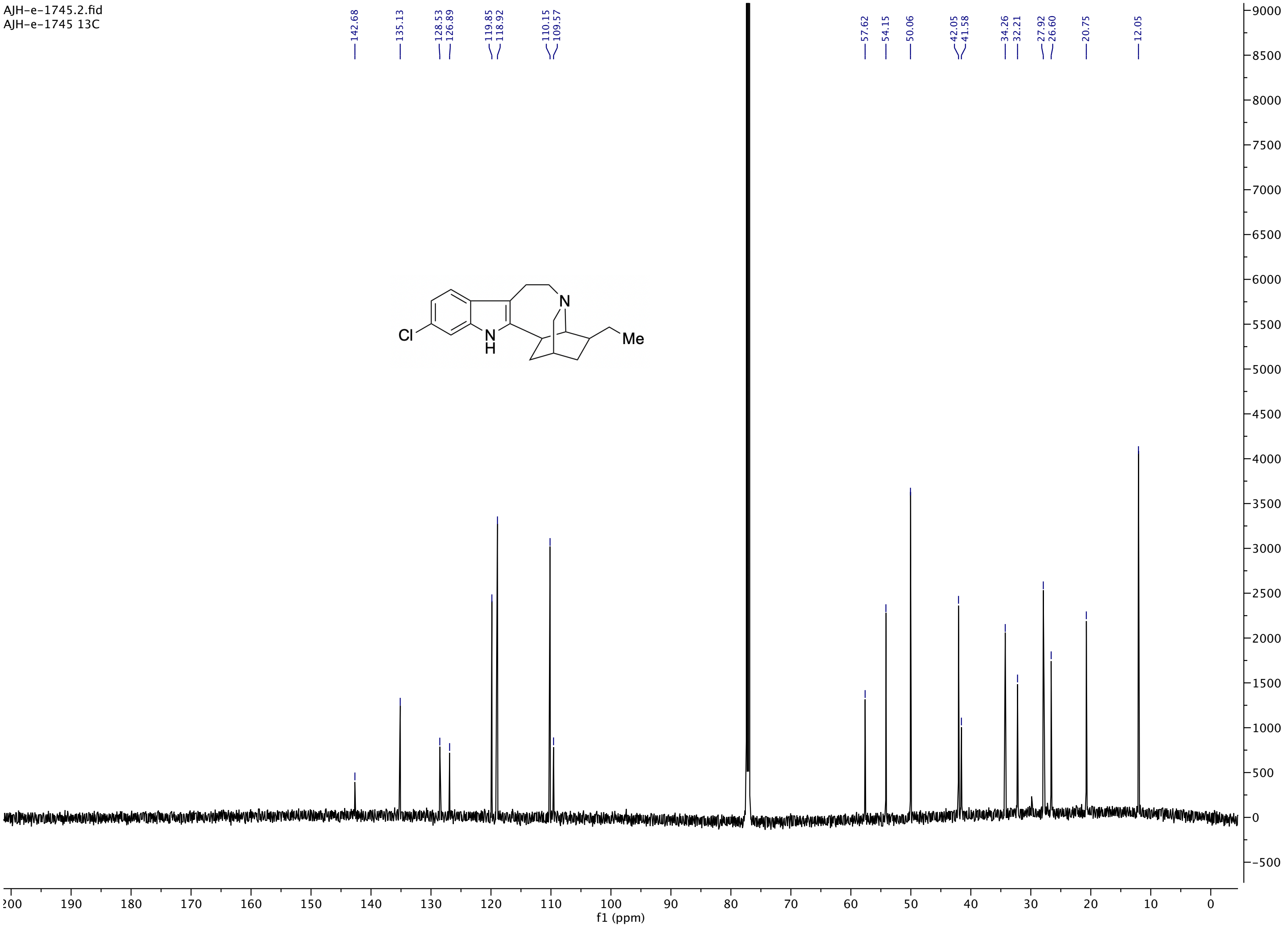

^{1}H NMR (600 MHz, $CDCl_3$) of Compound 7c

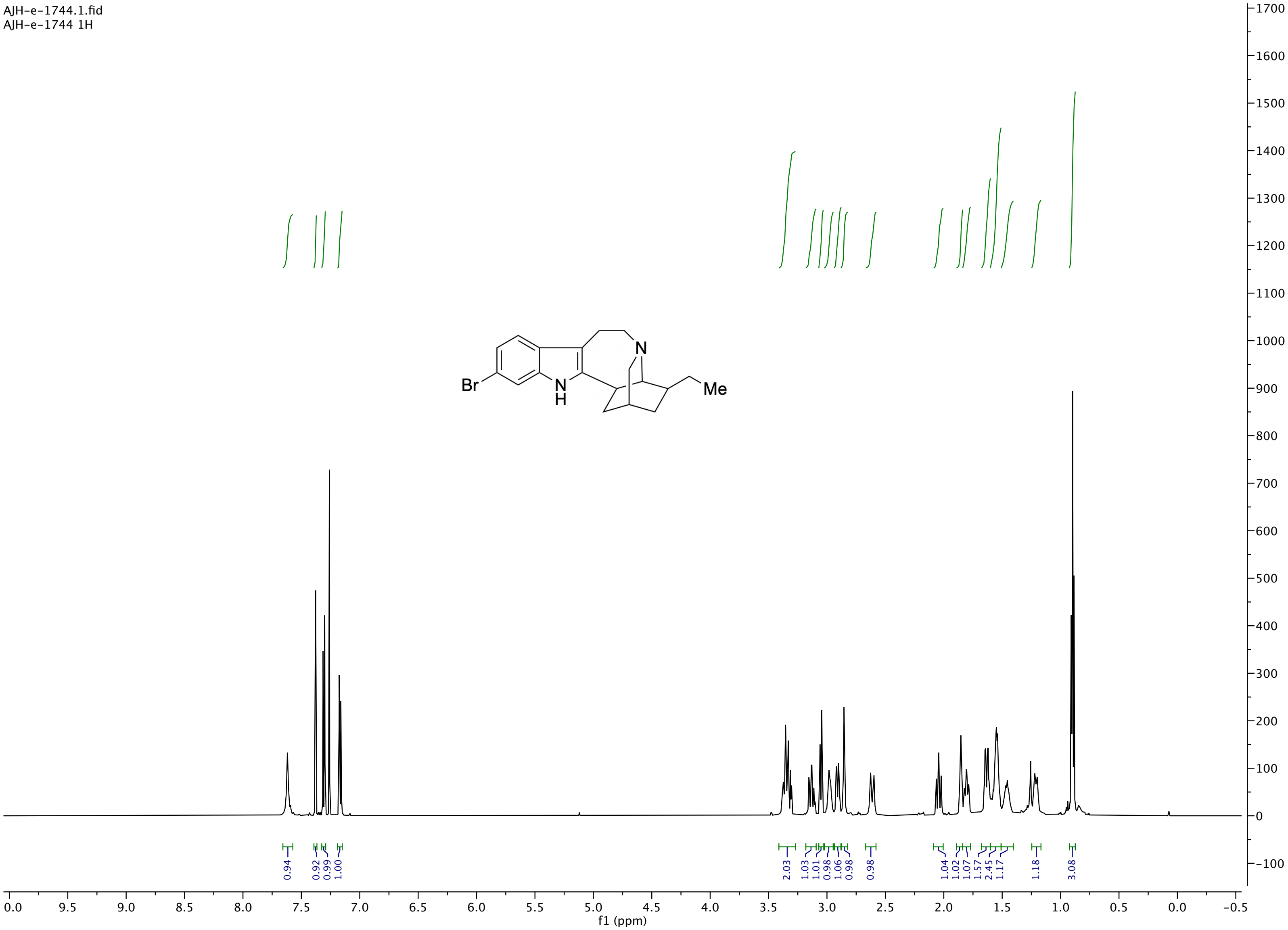

^{13}C NMR of (151 MHz, $CDCl_3$) of Compound 7c

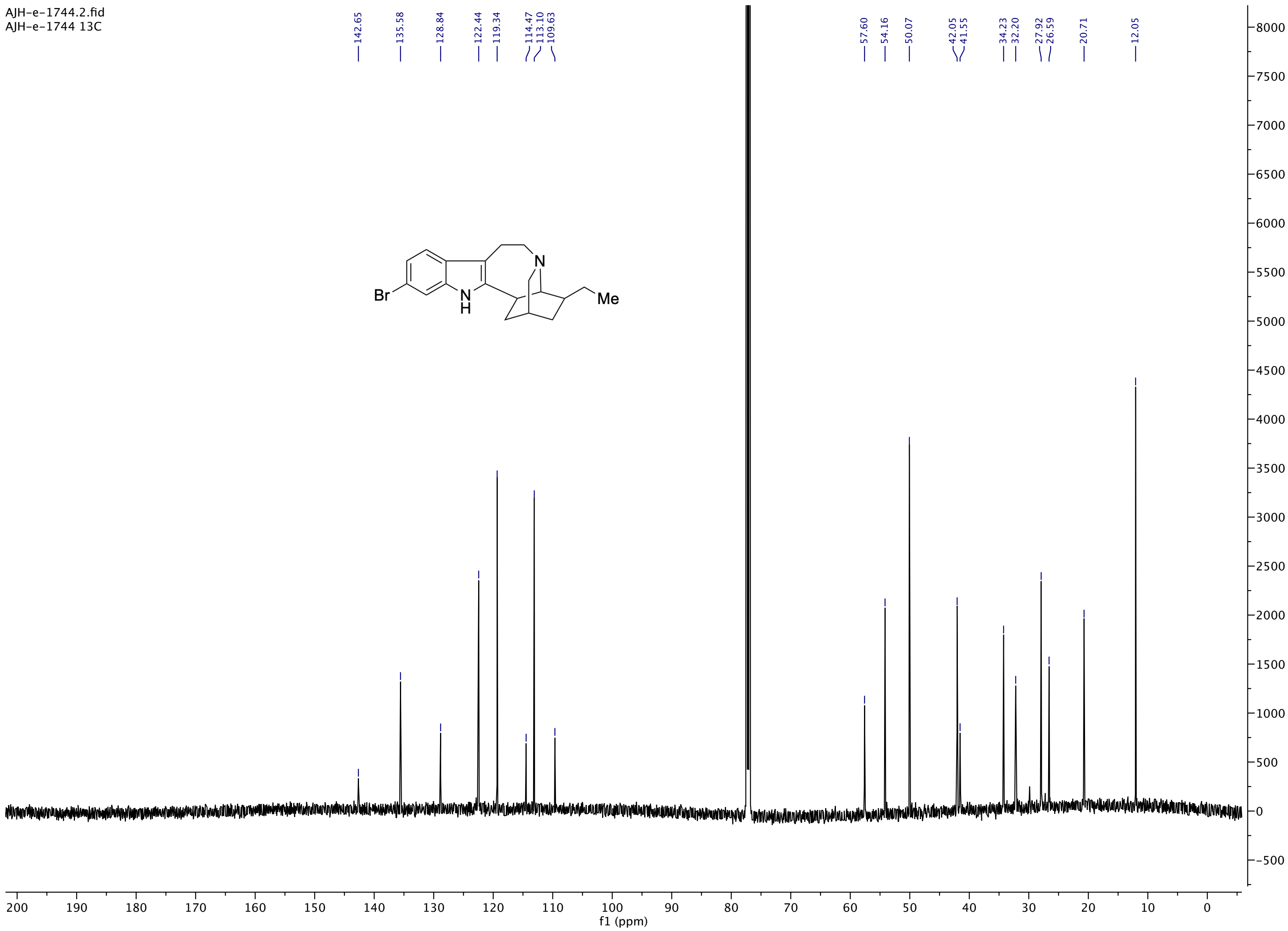

^{1}H NMR (600 MHz, CDCl₃) of tabernanthine, 3

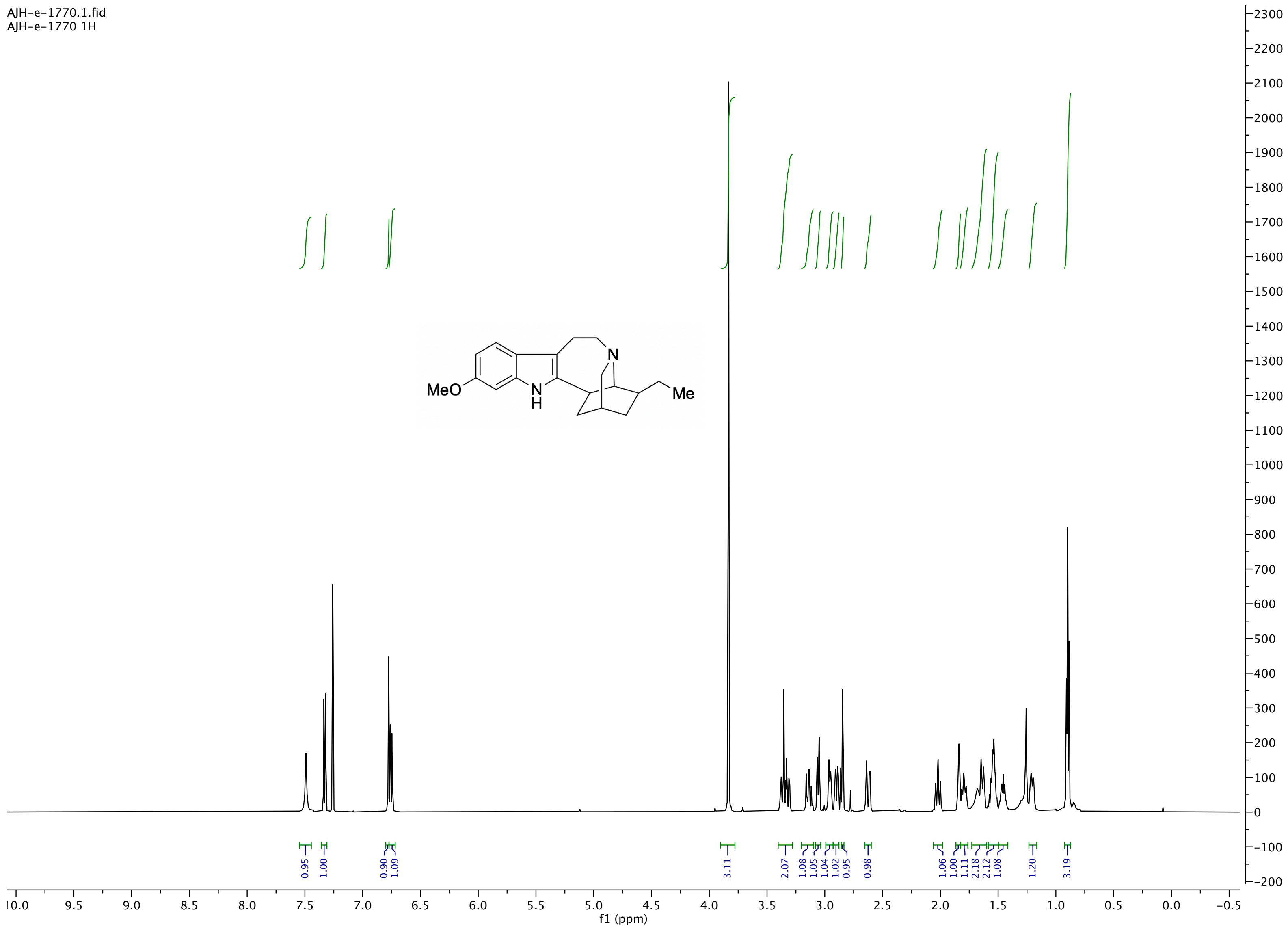

^{13}C NMR of (151 MHz, $CDCl_3$) of tabernanthine, 3

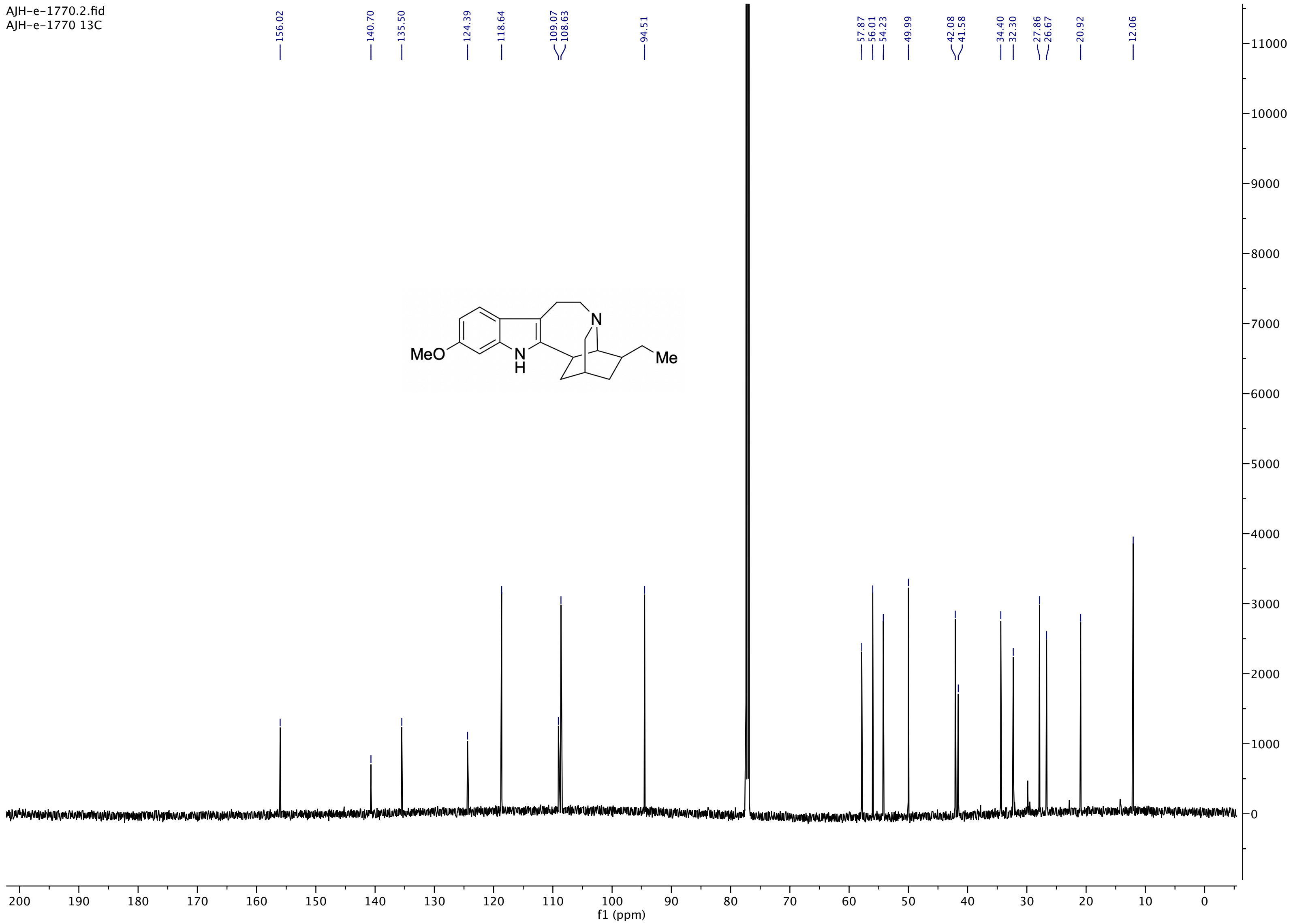

^{1}H NMR (600 MHz, $CDCl_3$) of Compound 7e

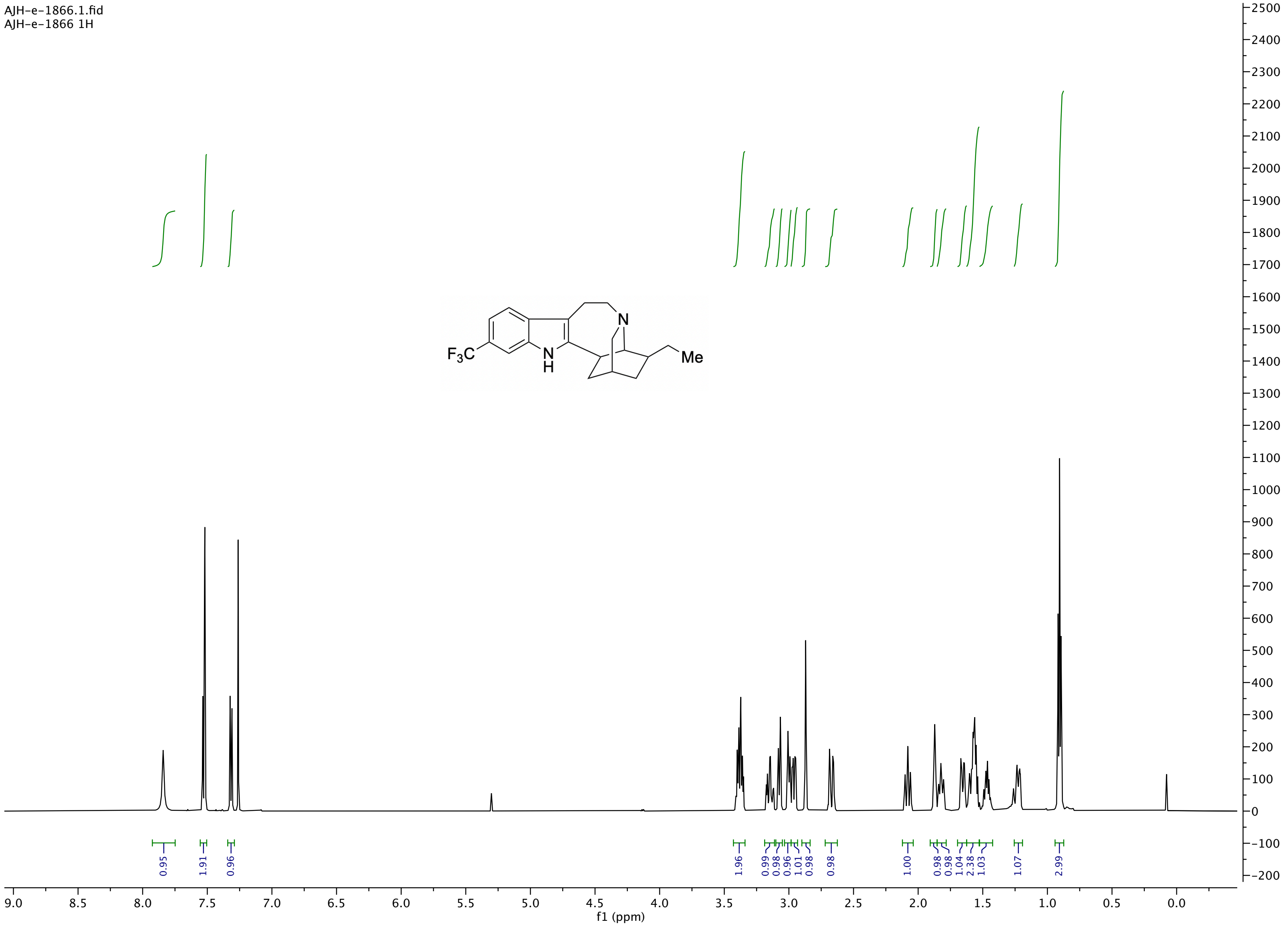

^{13}C NMR of (151 MHz, $CDCl_3$) of Compound 7e

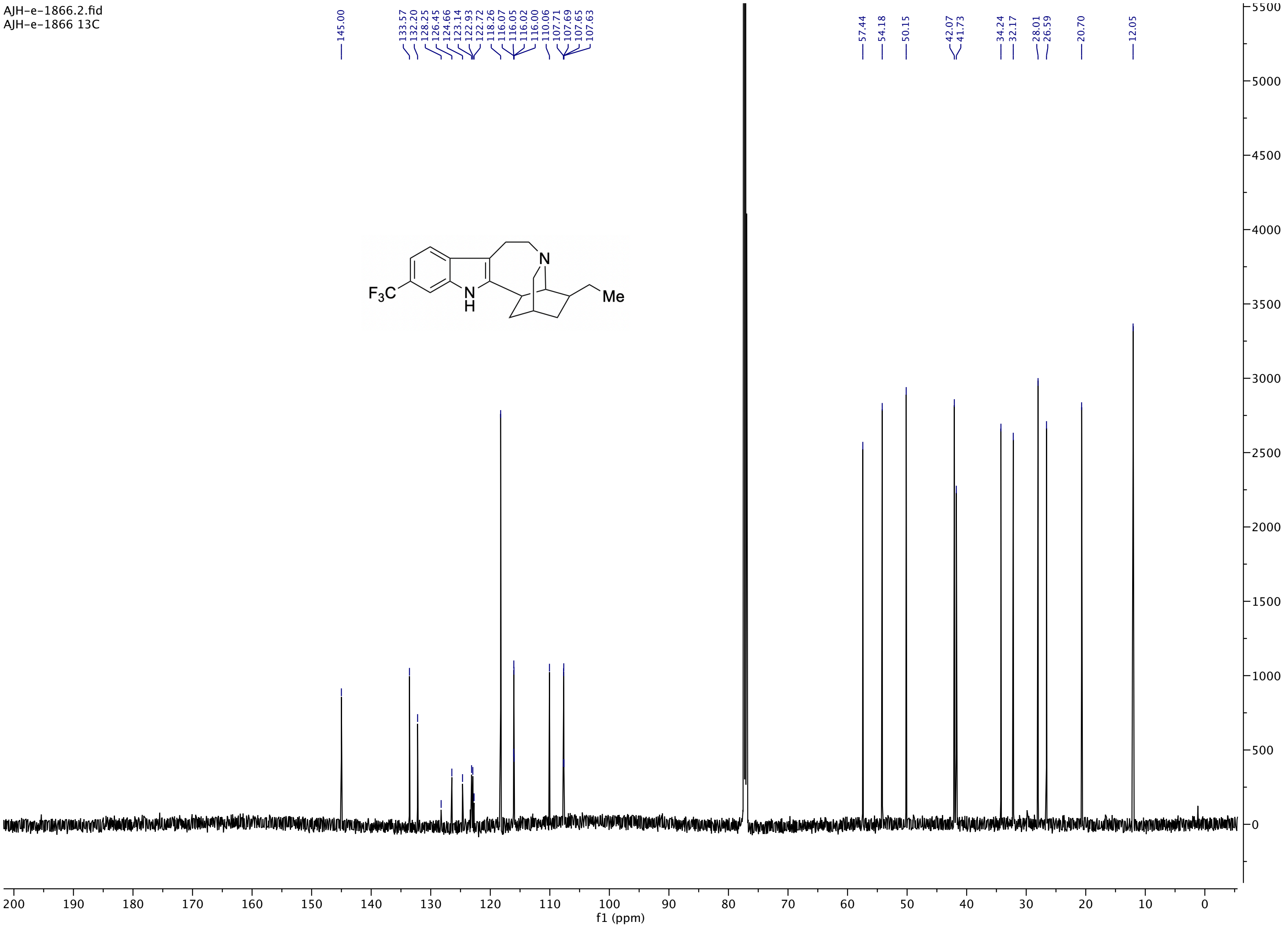

^{19}F NMR (471 MHz, $CDCl_3$) of 7e

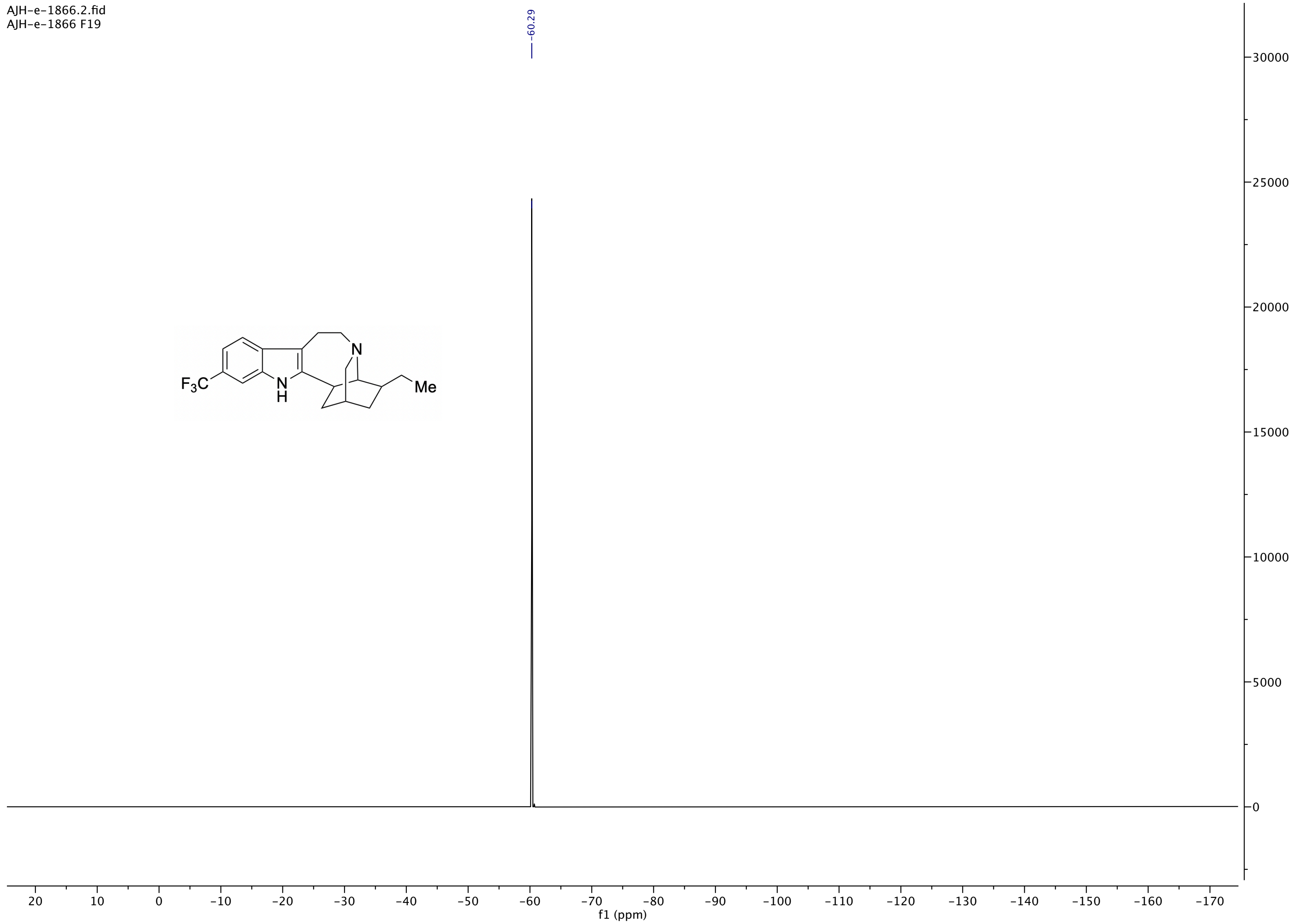

^{1}H NMR (600 MHz, $CDCl_3$) of Compound 7f

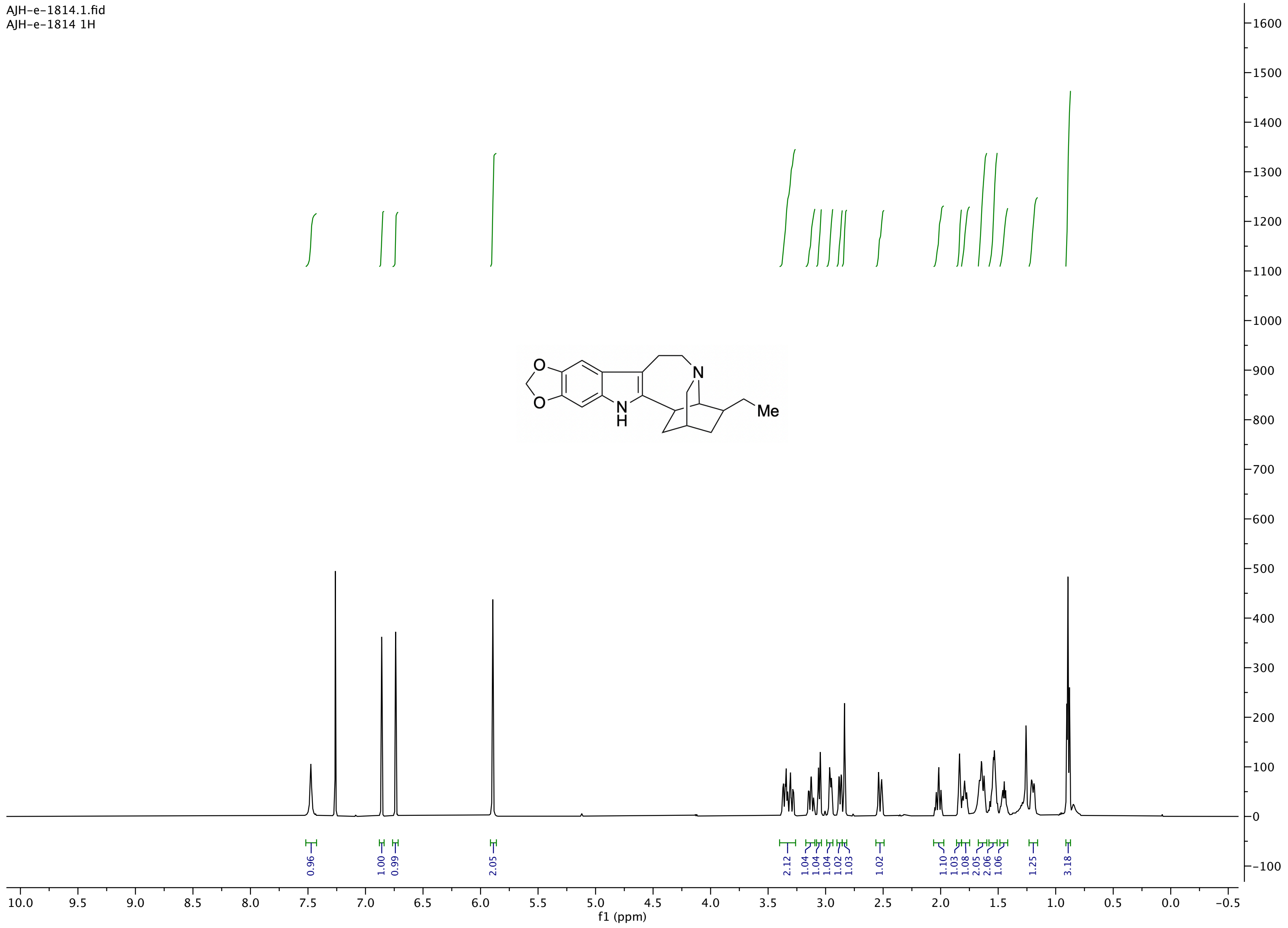

^{13}C NMR of (151 MHz, $CDCl_3$) of Compound 7f

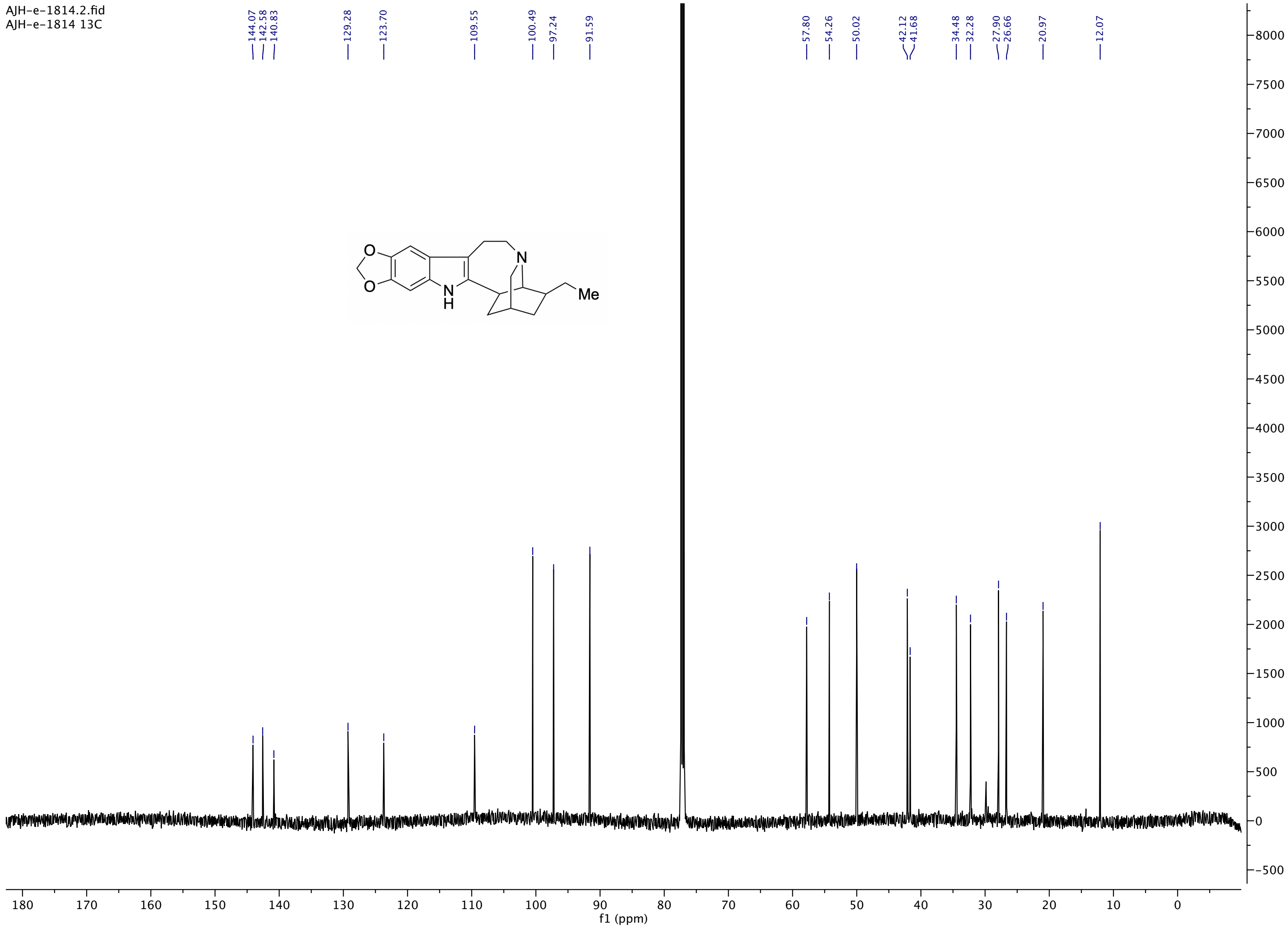

^{1}H NMR (600 MHz, $CDCl_3$) of Compound 7g

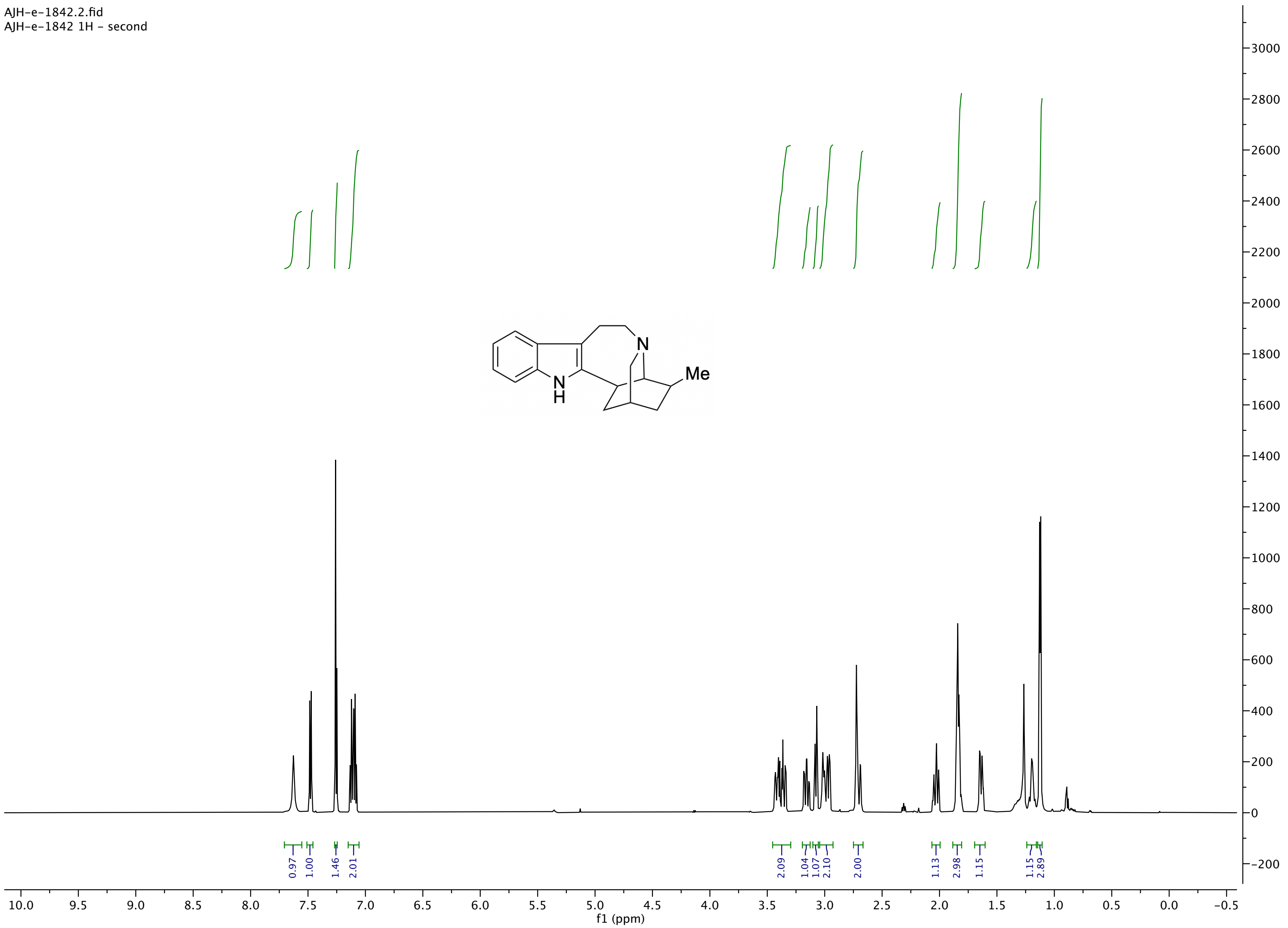

^{13}C NMR of (151 MHz, $CDCl_3$) of Compound 7g

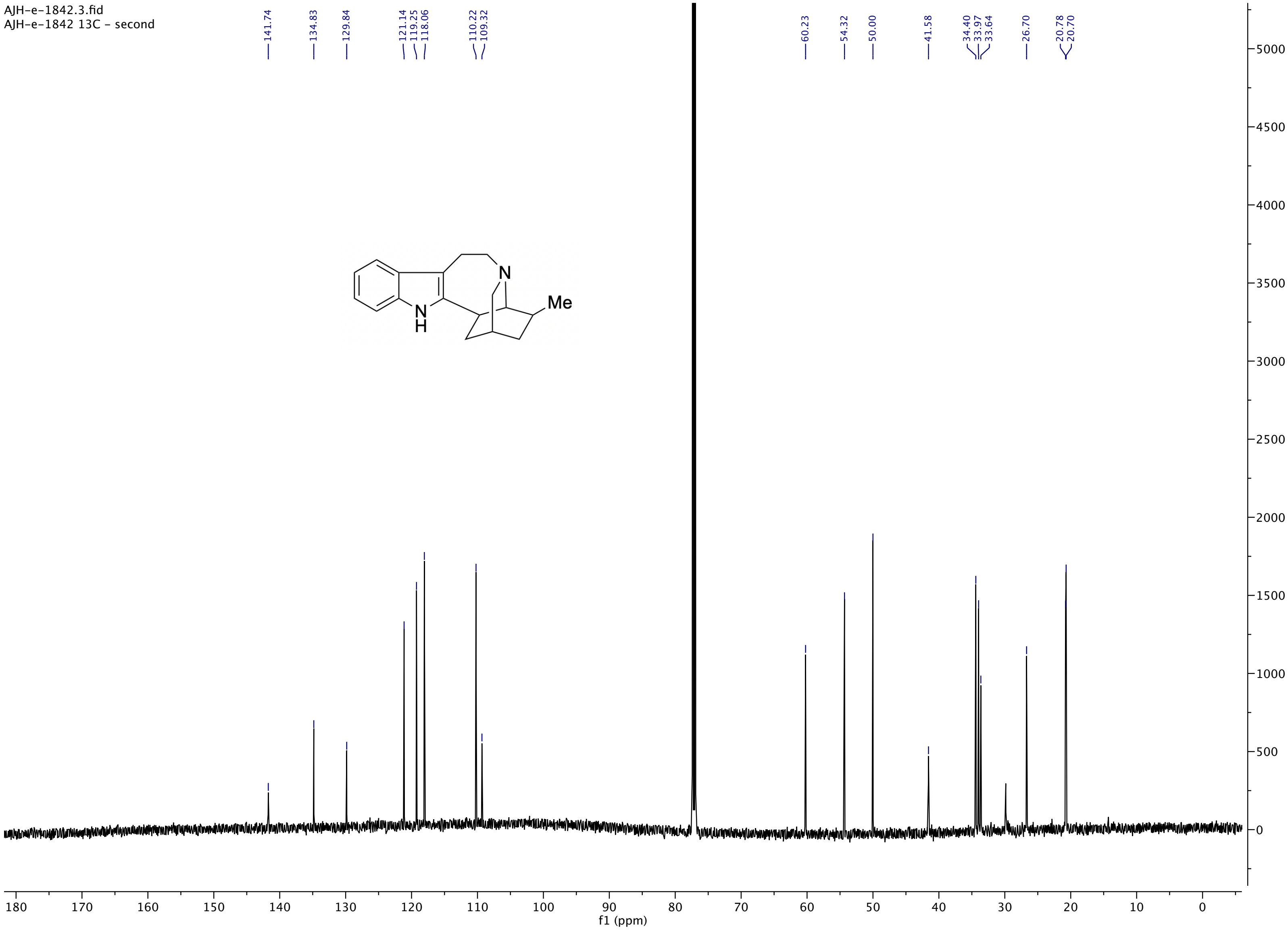

^{1}H NMR (600 MHz, $CDCl_3$) of Compound 7h

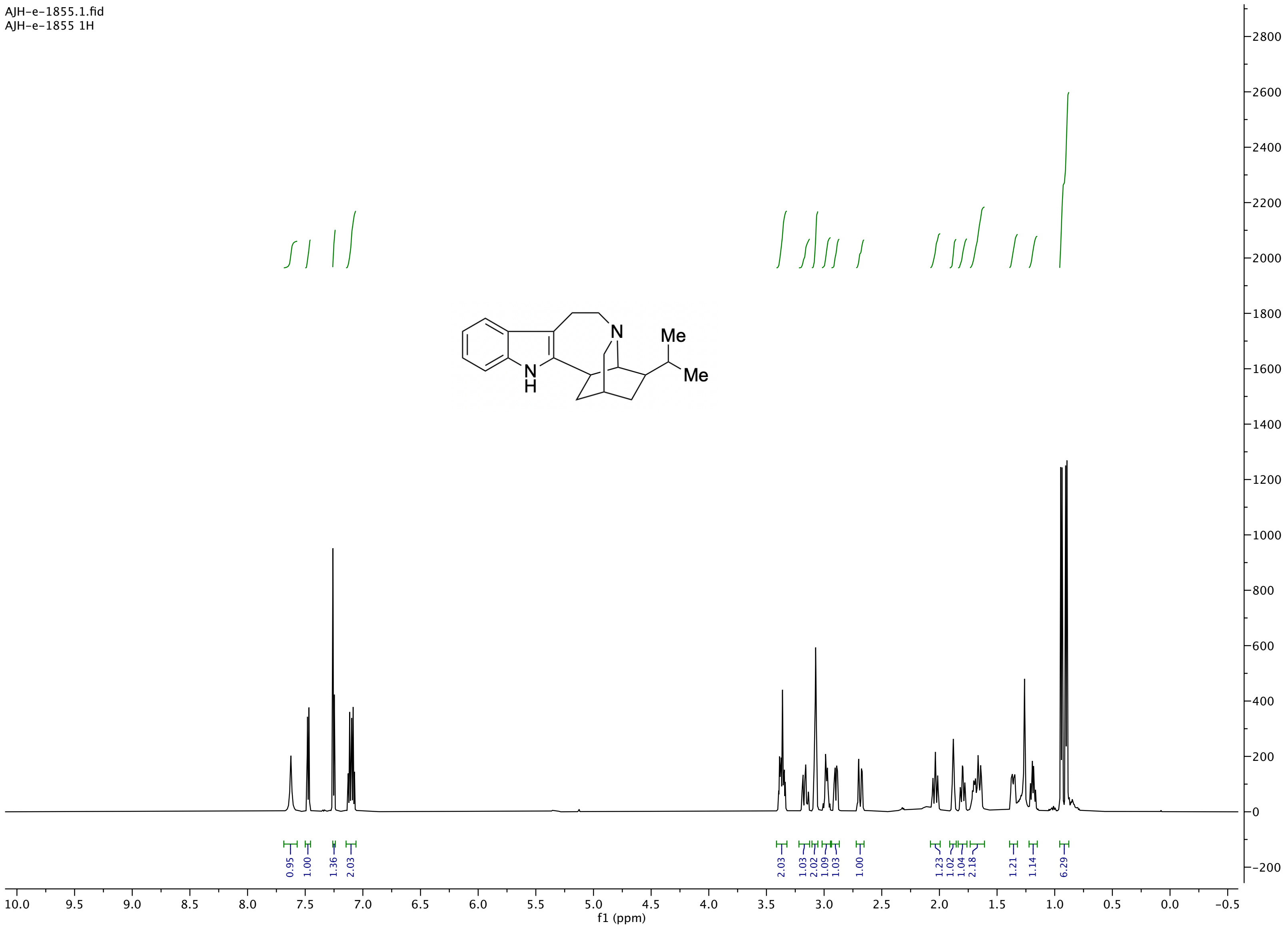

^{13}C NMR of (151 MHz, $CDCl_3$) of Compound 7h

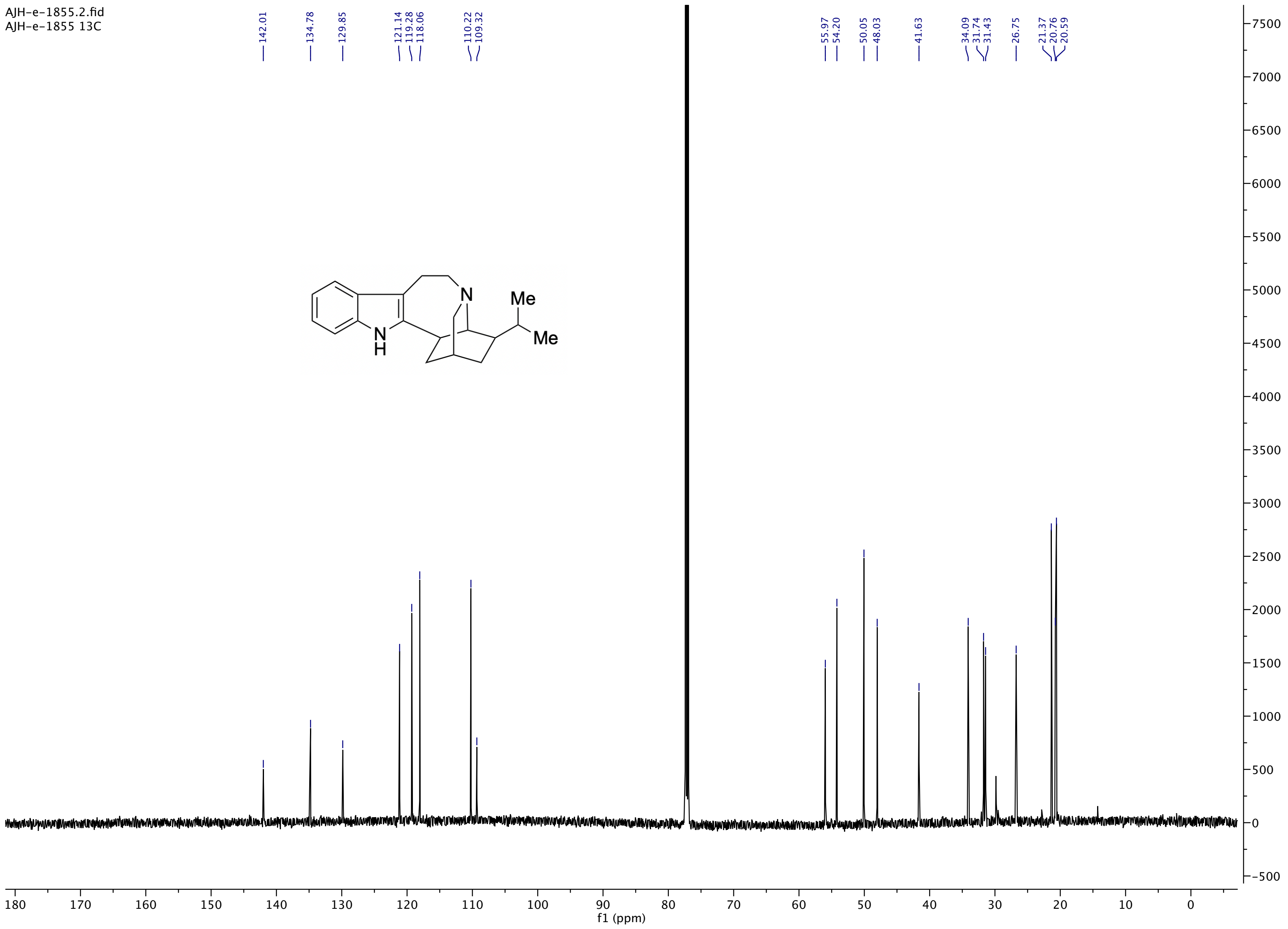

^{1}H NMR (600 MHz, $CDCl_3$) of Compound 7i

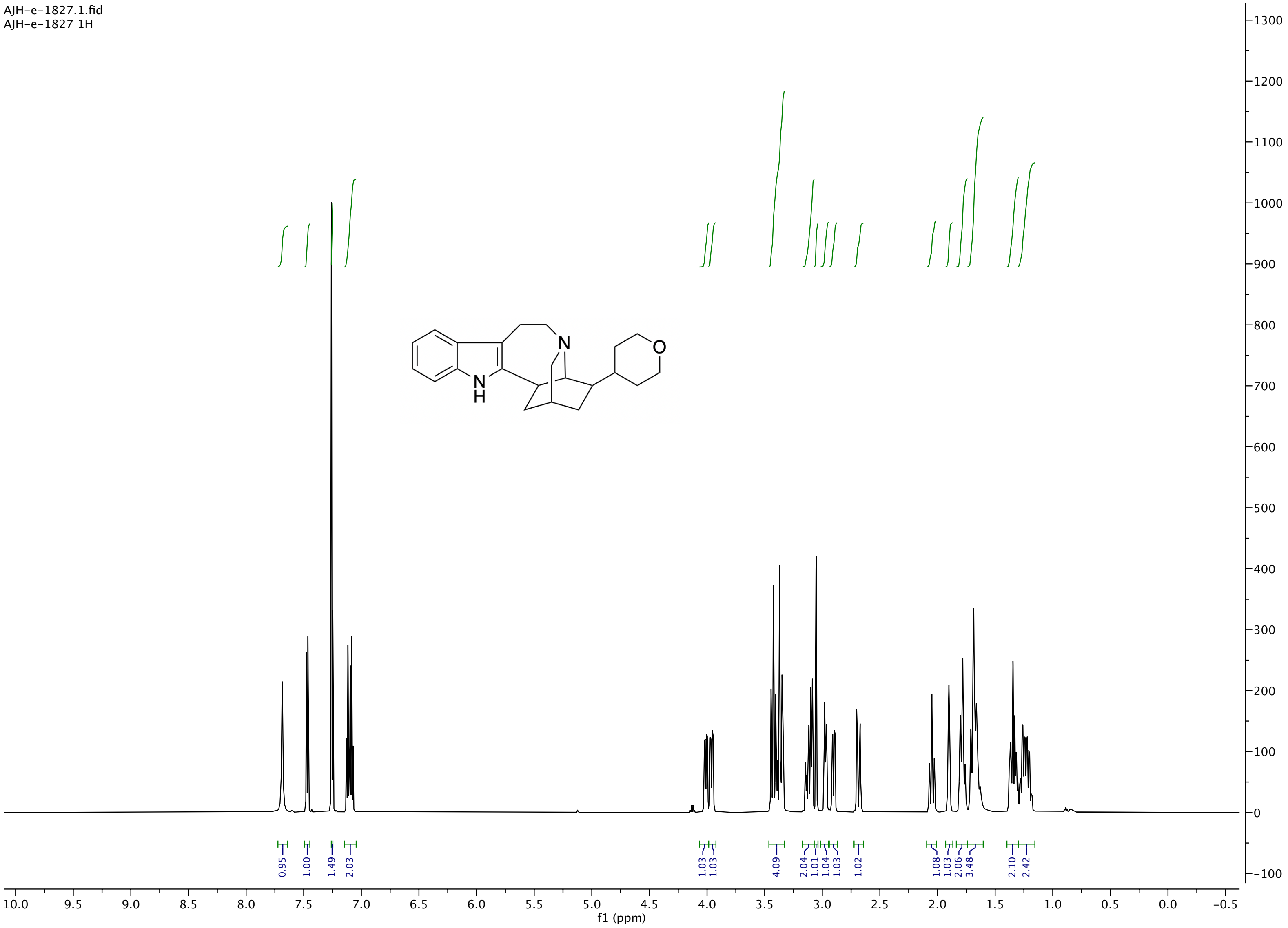

^{13}C NMR of (151 MHz, $CDCl_3$) of Compound 7i

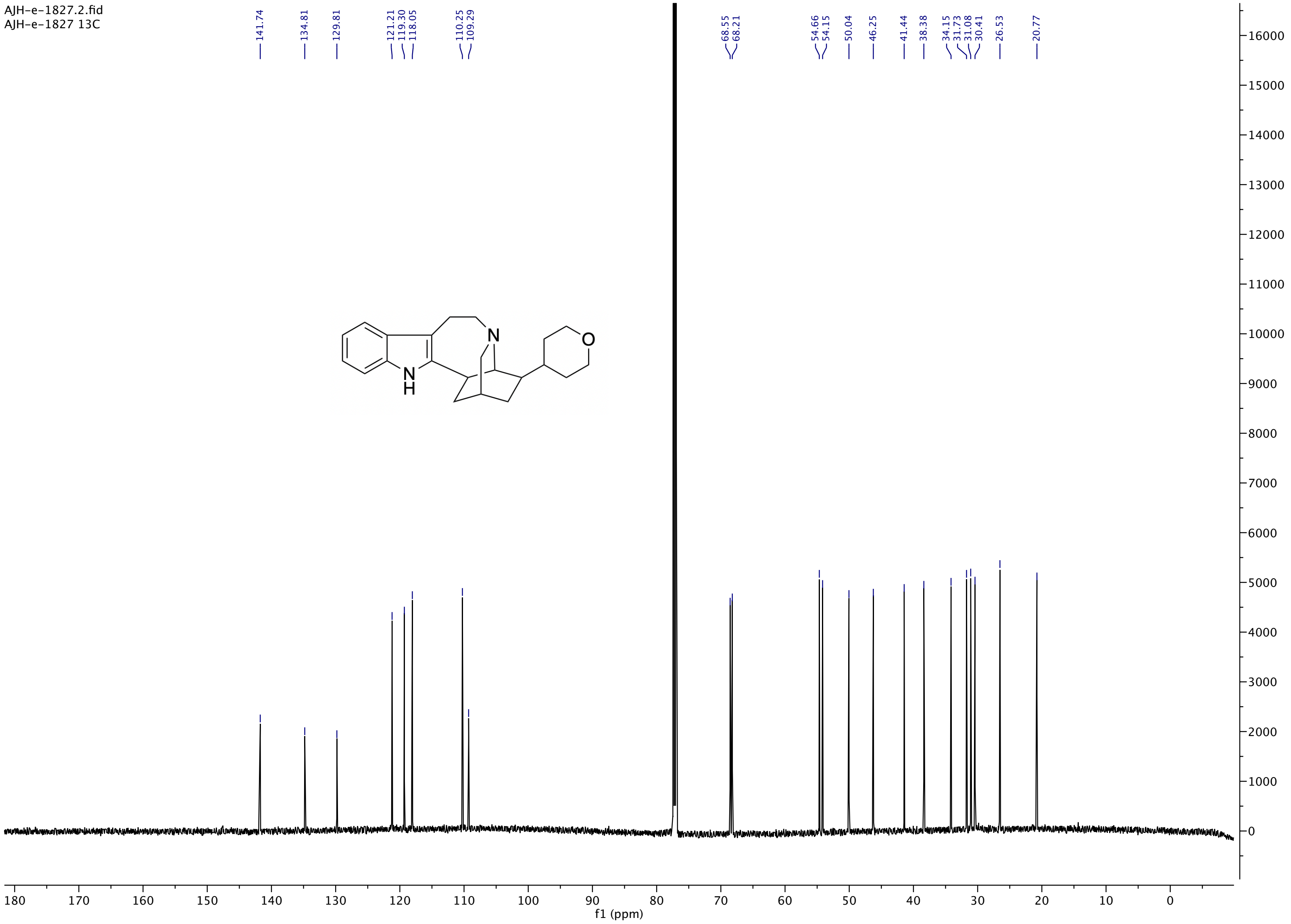

^{1}H-^{1}H COSY NMR ($CDCl_3$) of 7i

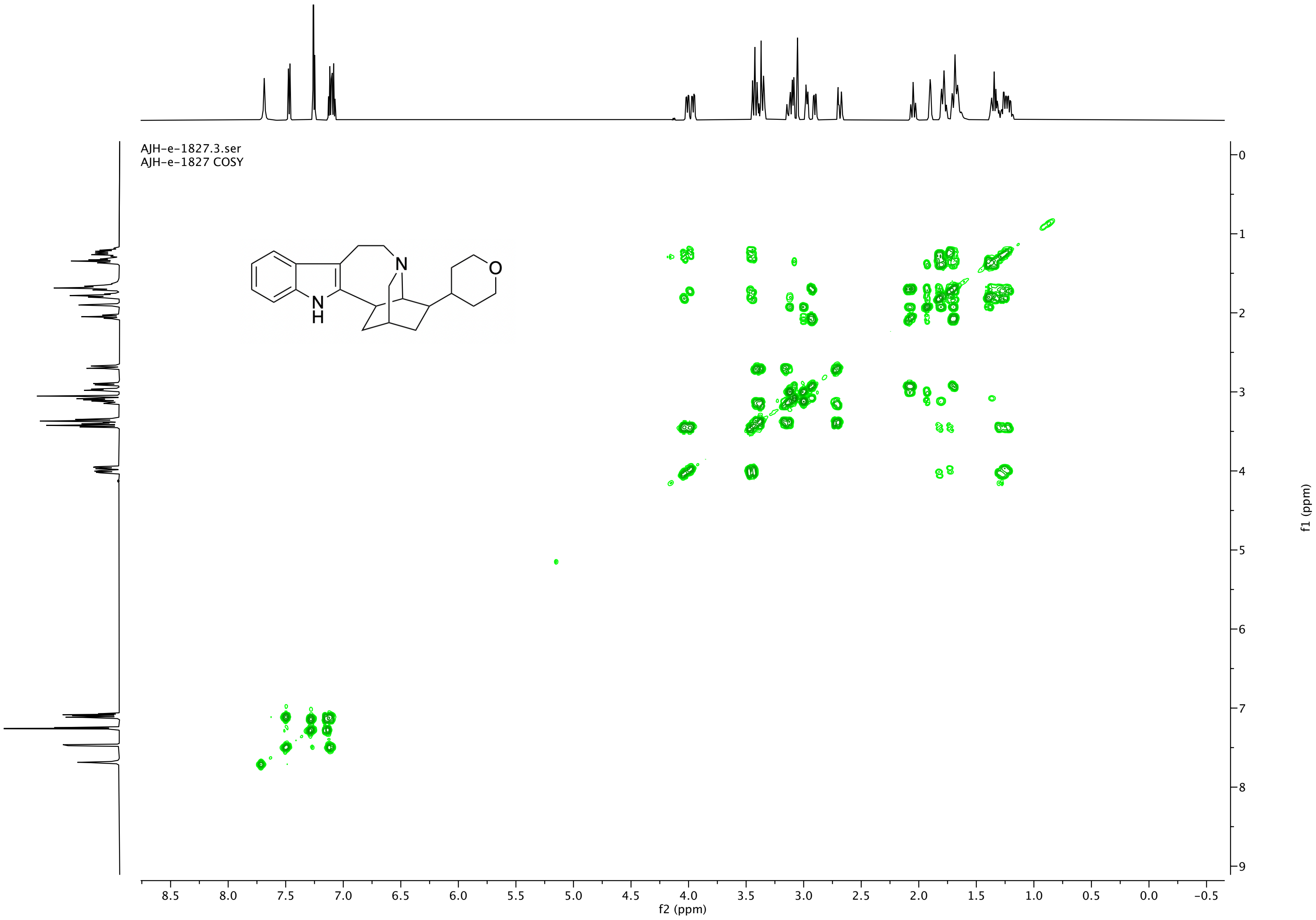

^{1}H-^{13}C HSQC NMR ($CDCl_3$) of 7i

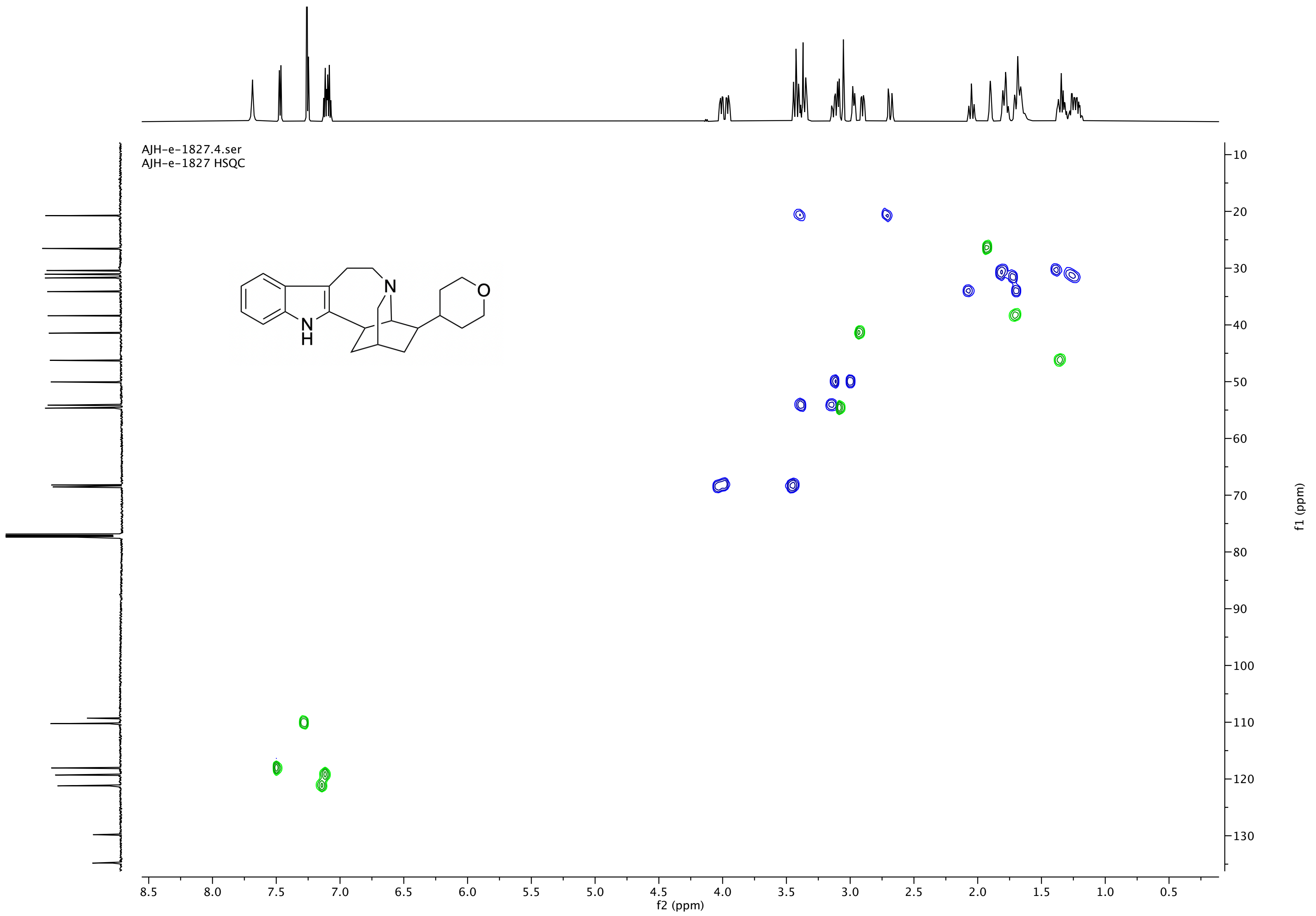

^{1}H NMR (600 MHz, DMSO) of Compound 20

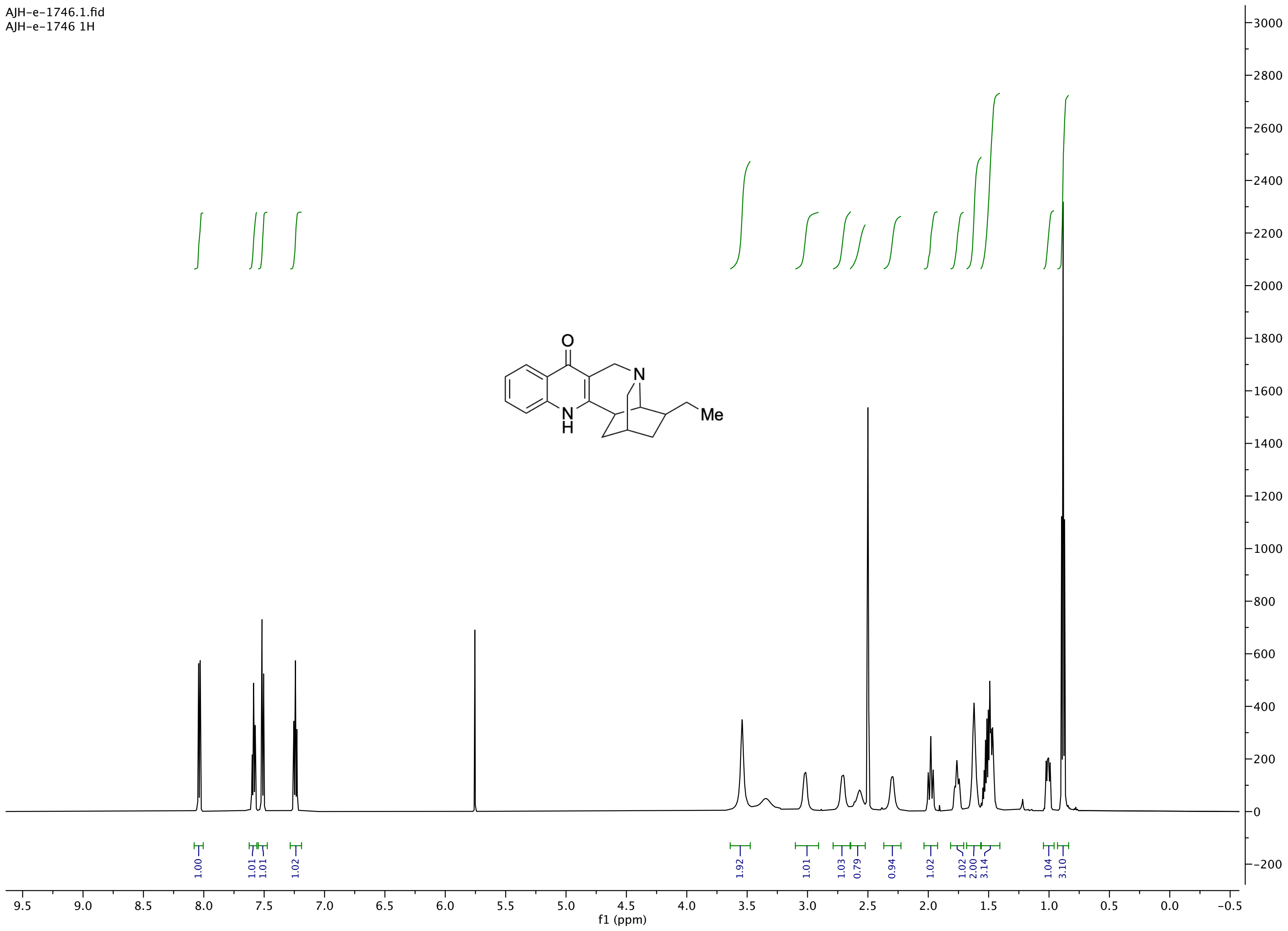

^{13}C NMR of (151 MHz, DMSO) of Compound 20

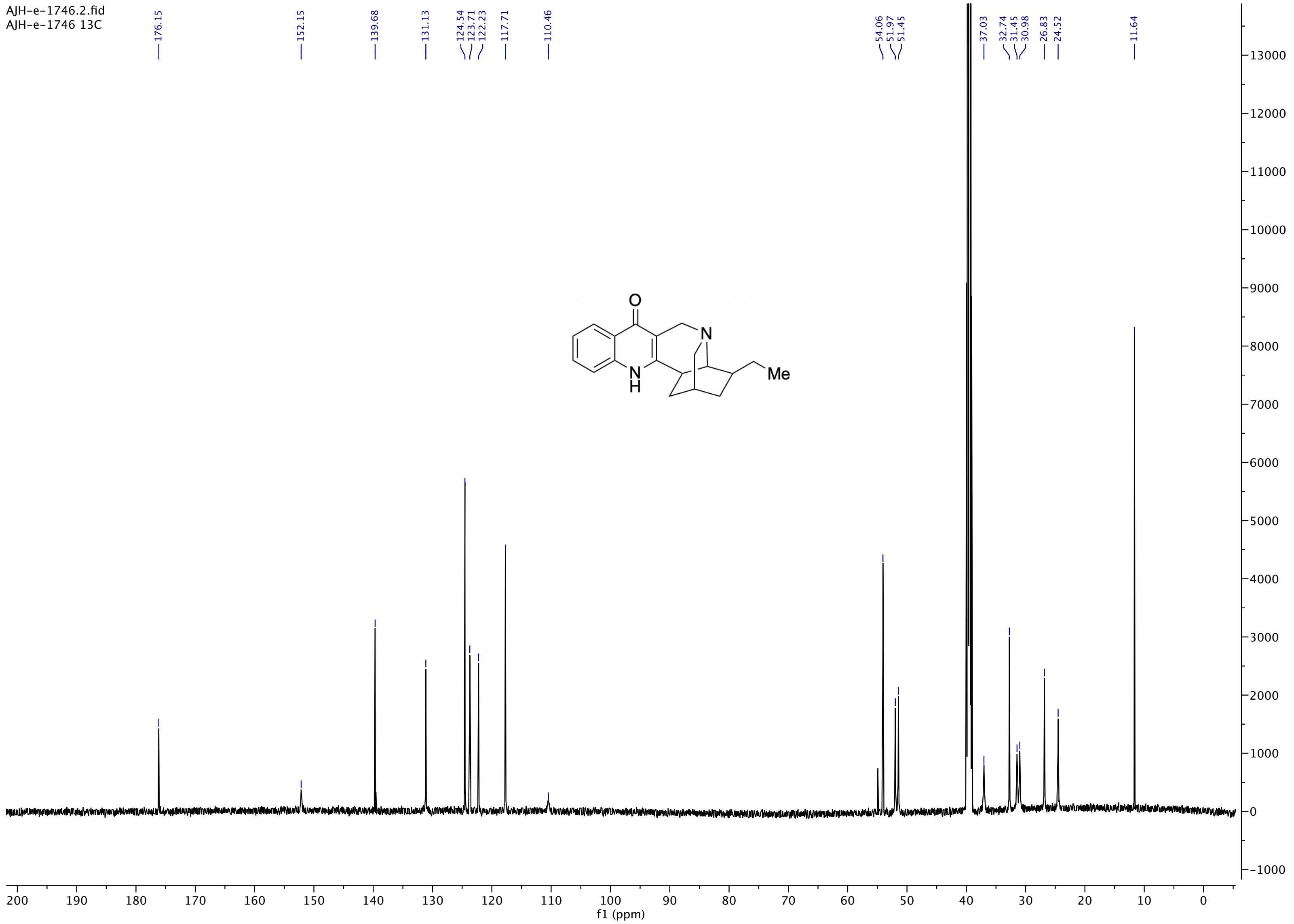

^{1}H-^{1}H COSY NMR (DMSO) of 20

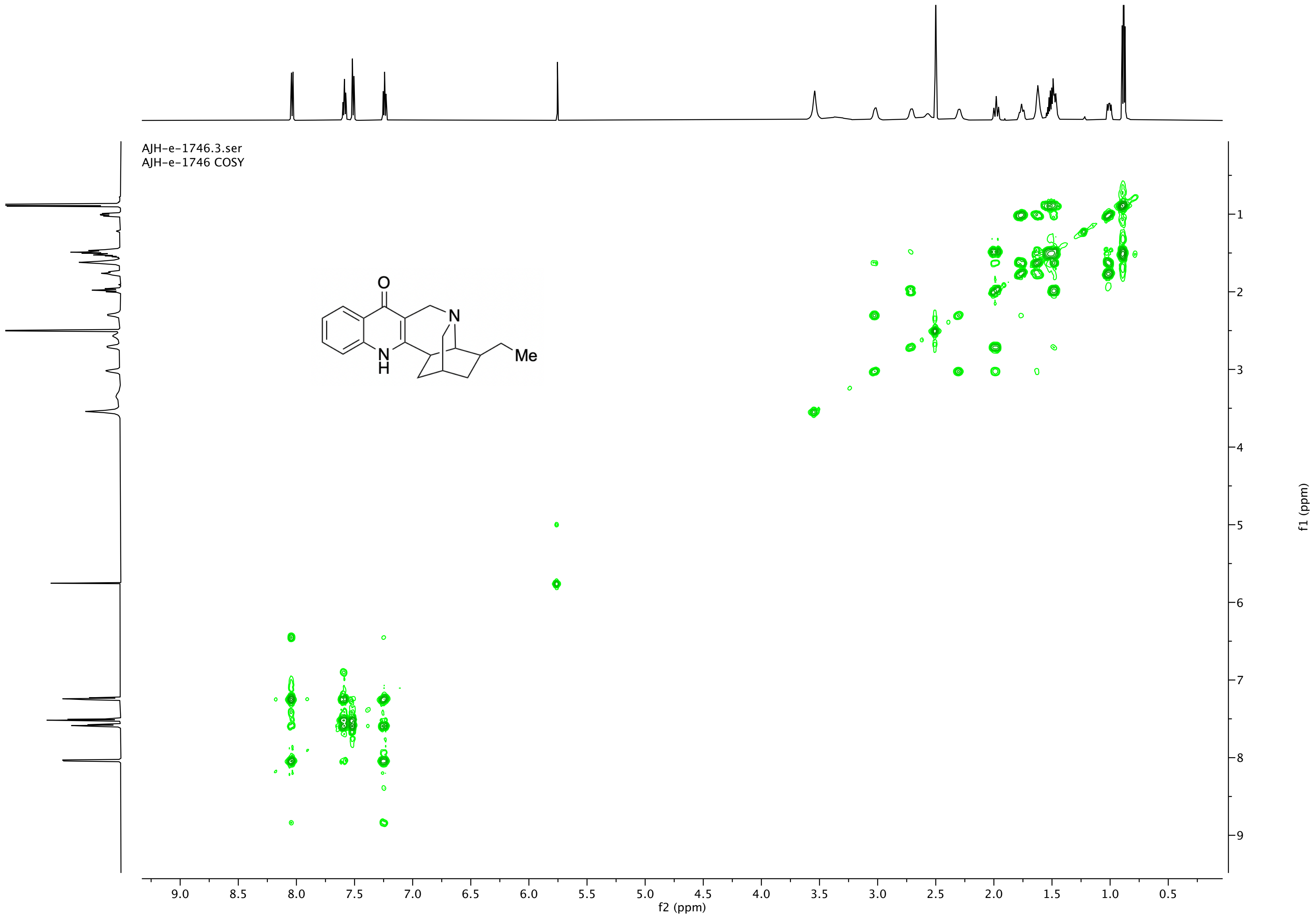

^{1}H-^{13}C HSQC NMR ($CDCl_3$) of 20

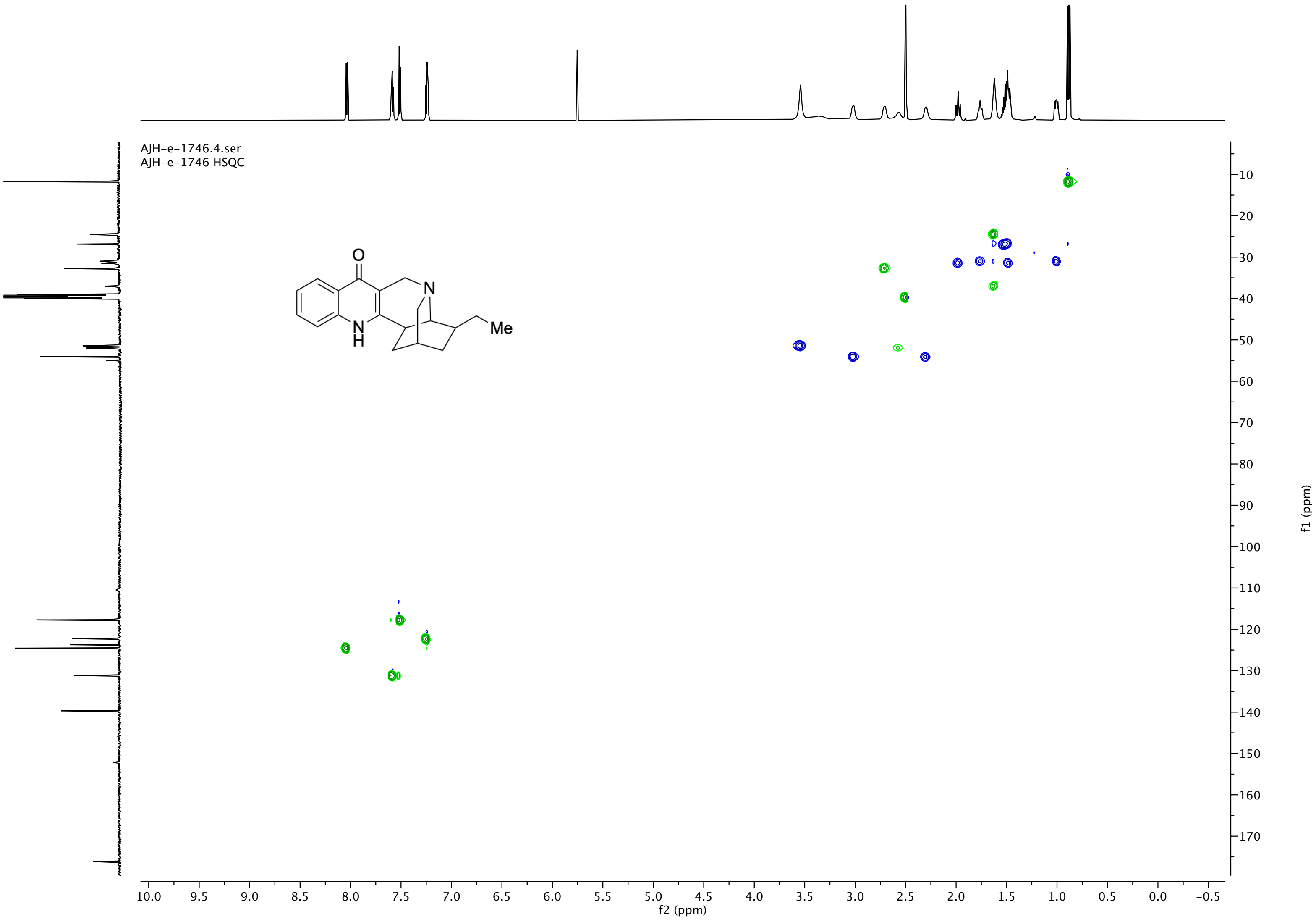

^{1}H-^{13}C HMBC NMR ($CDCl_3$) of 20

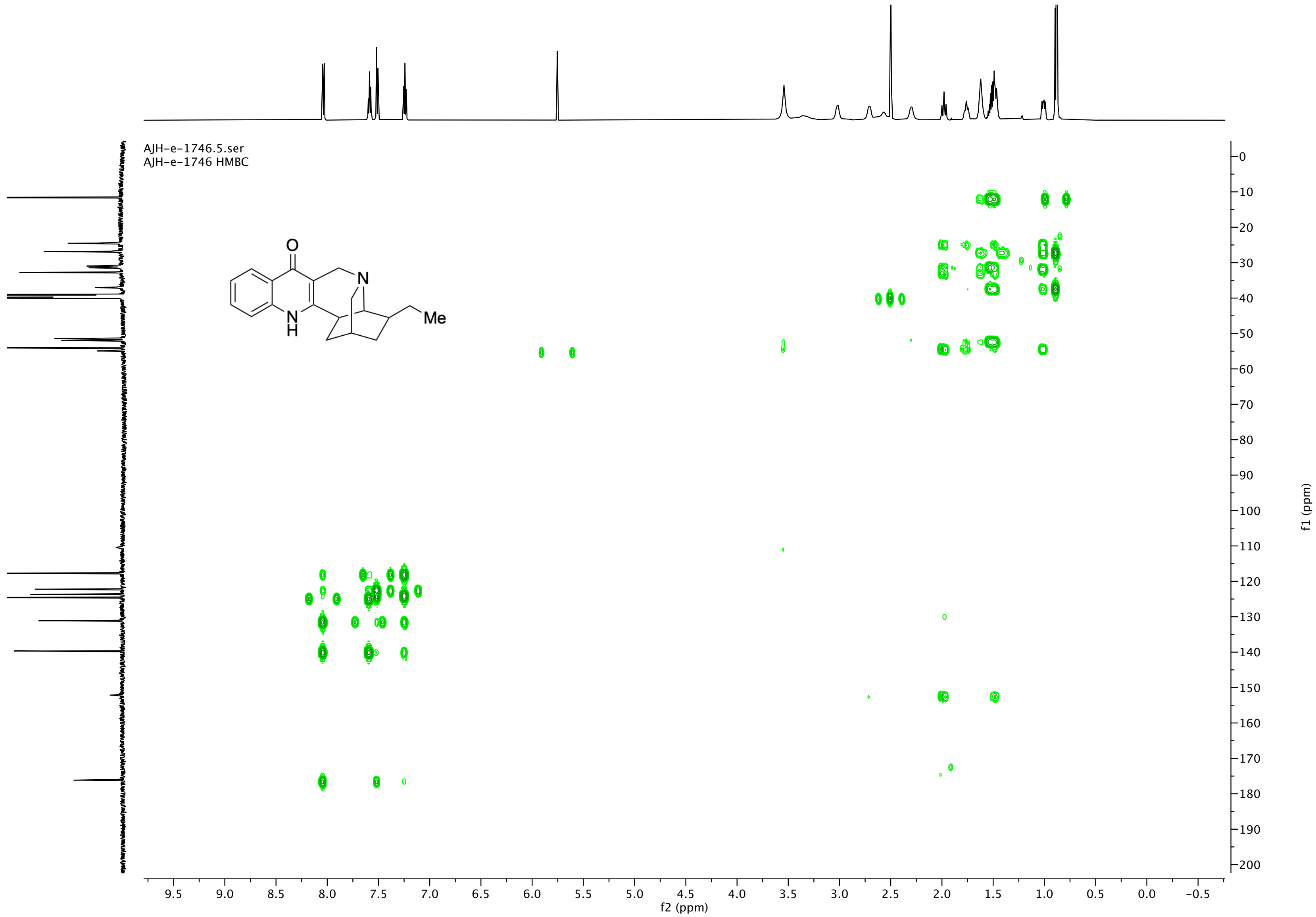

^{1}H NMR (600 MHz, DMSO) of Compound 21

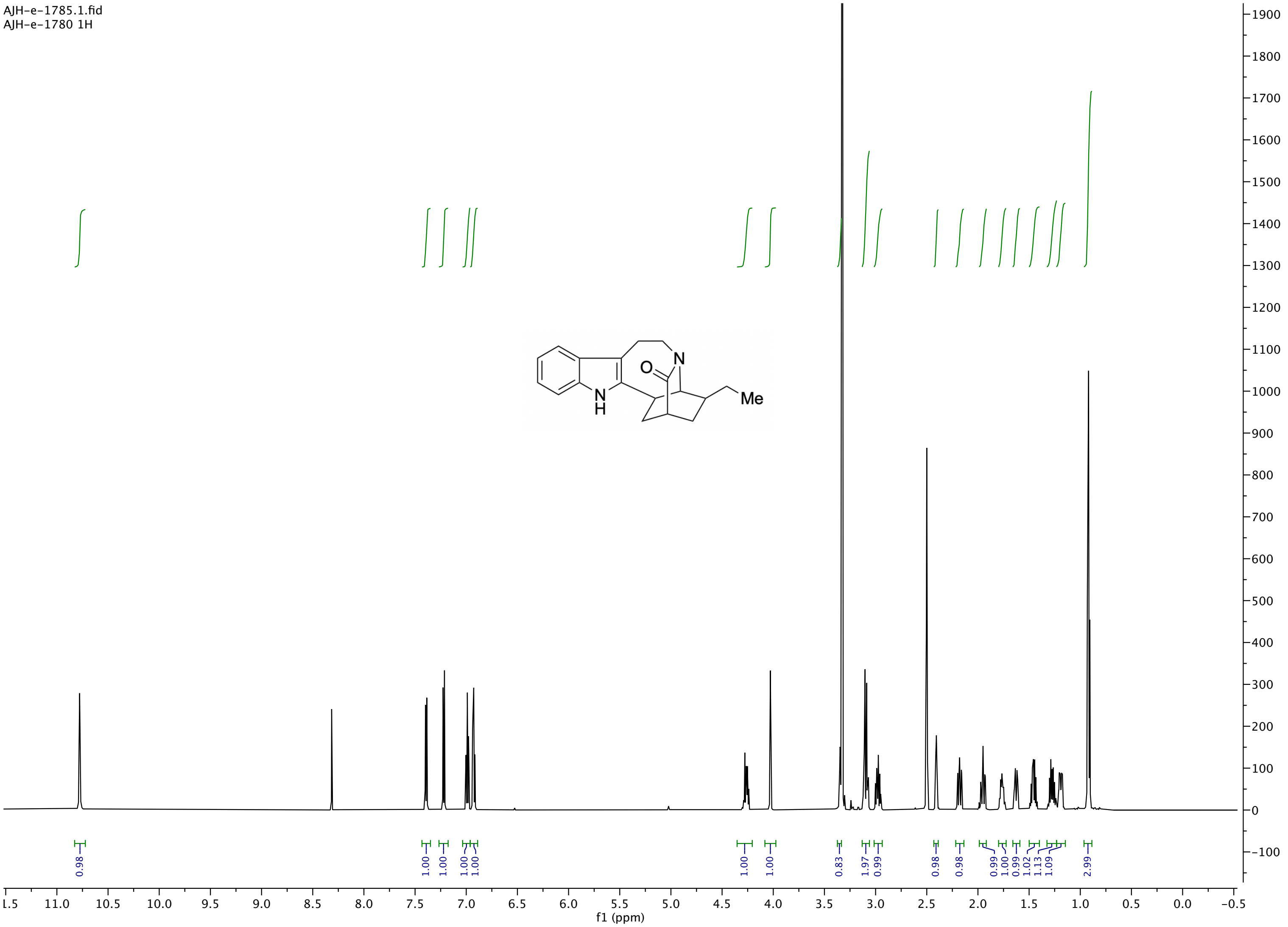

^{13}C NMR of (151 MHz, DMSO) of Compound 21

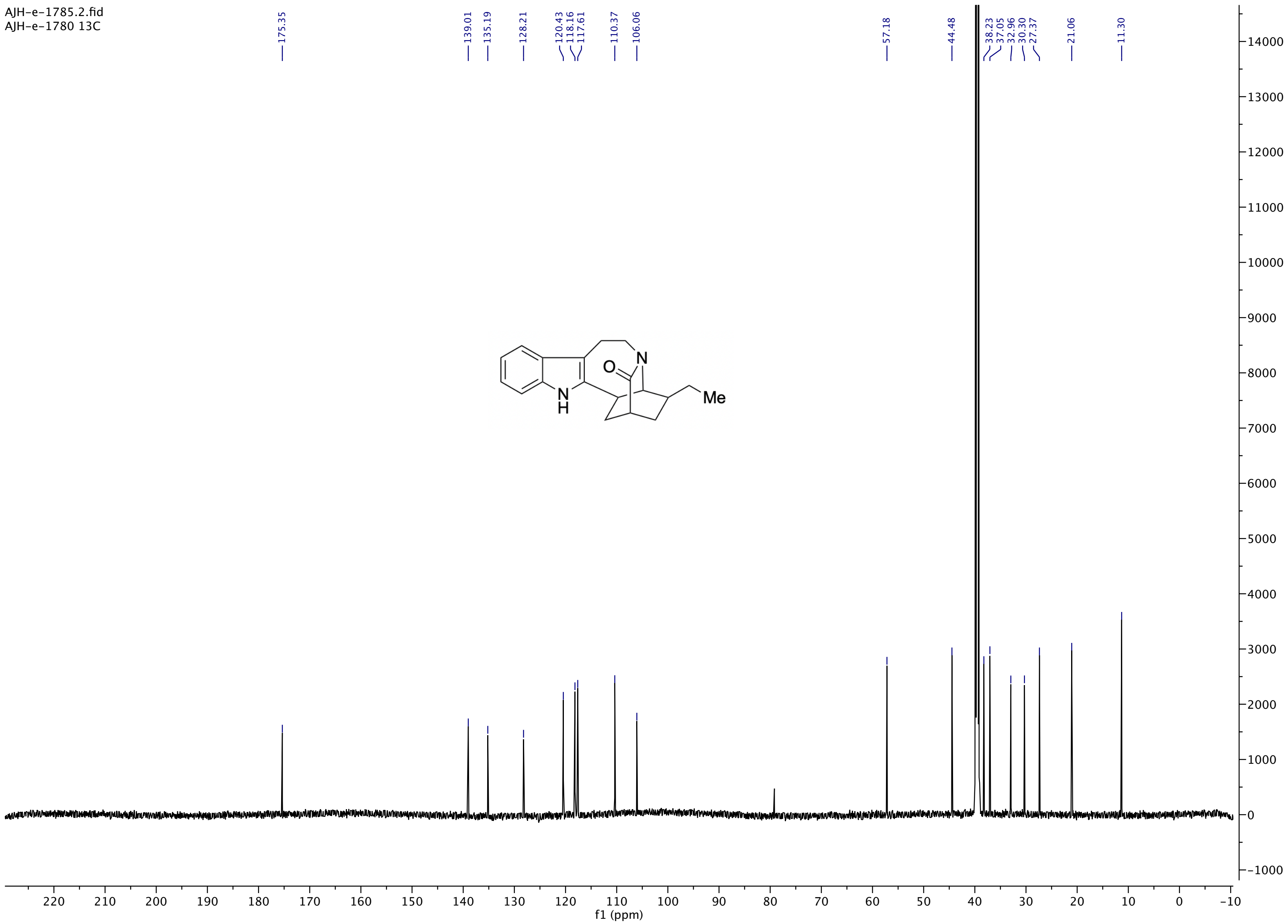

^{1}H-^{1}H COSY NMR (DMSO) of 21

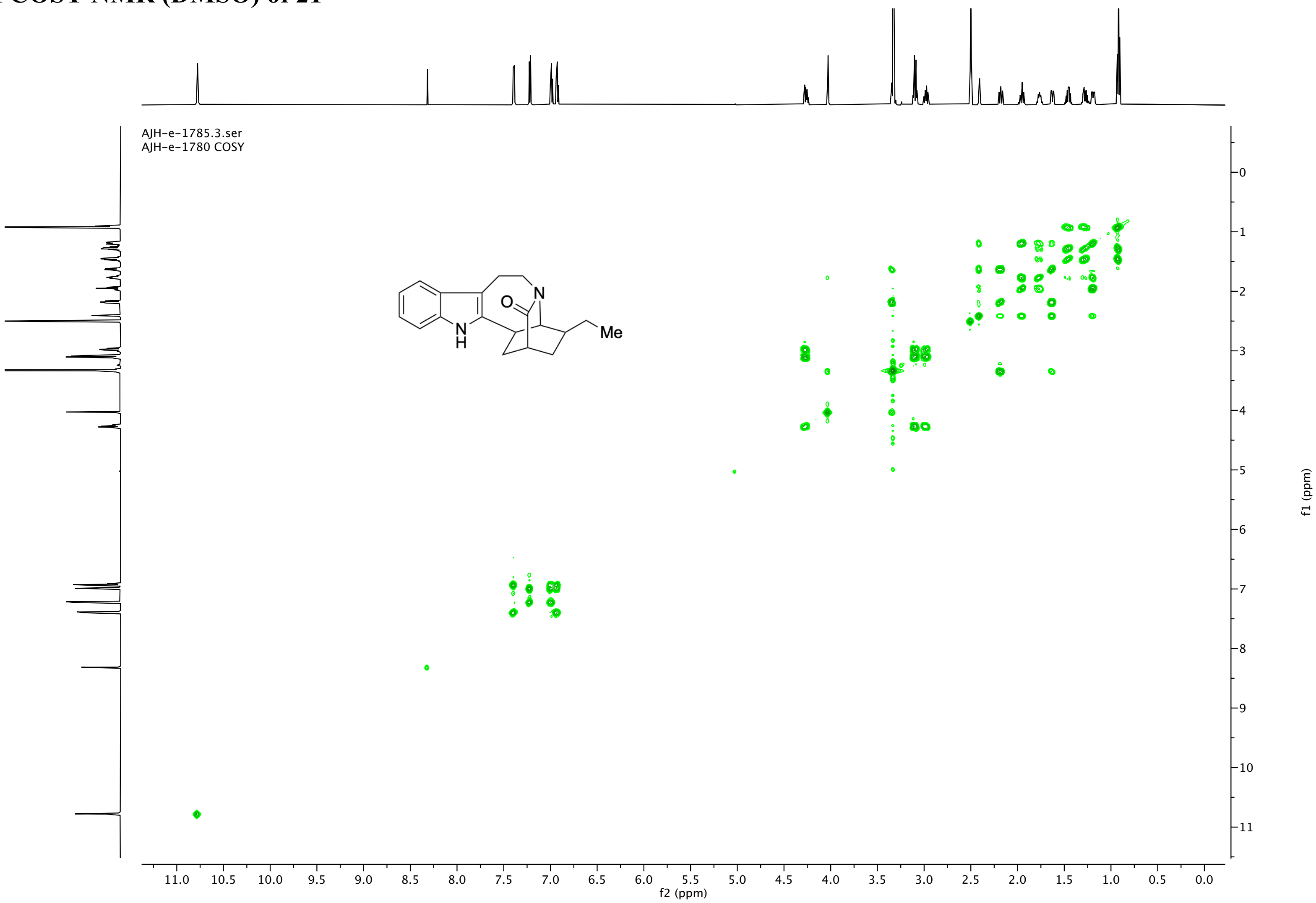

^{1}H-^{13}C HSQC NMR ($CDCl_3$) of 21

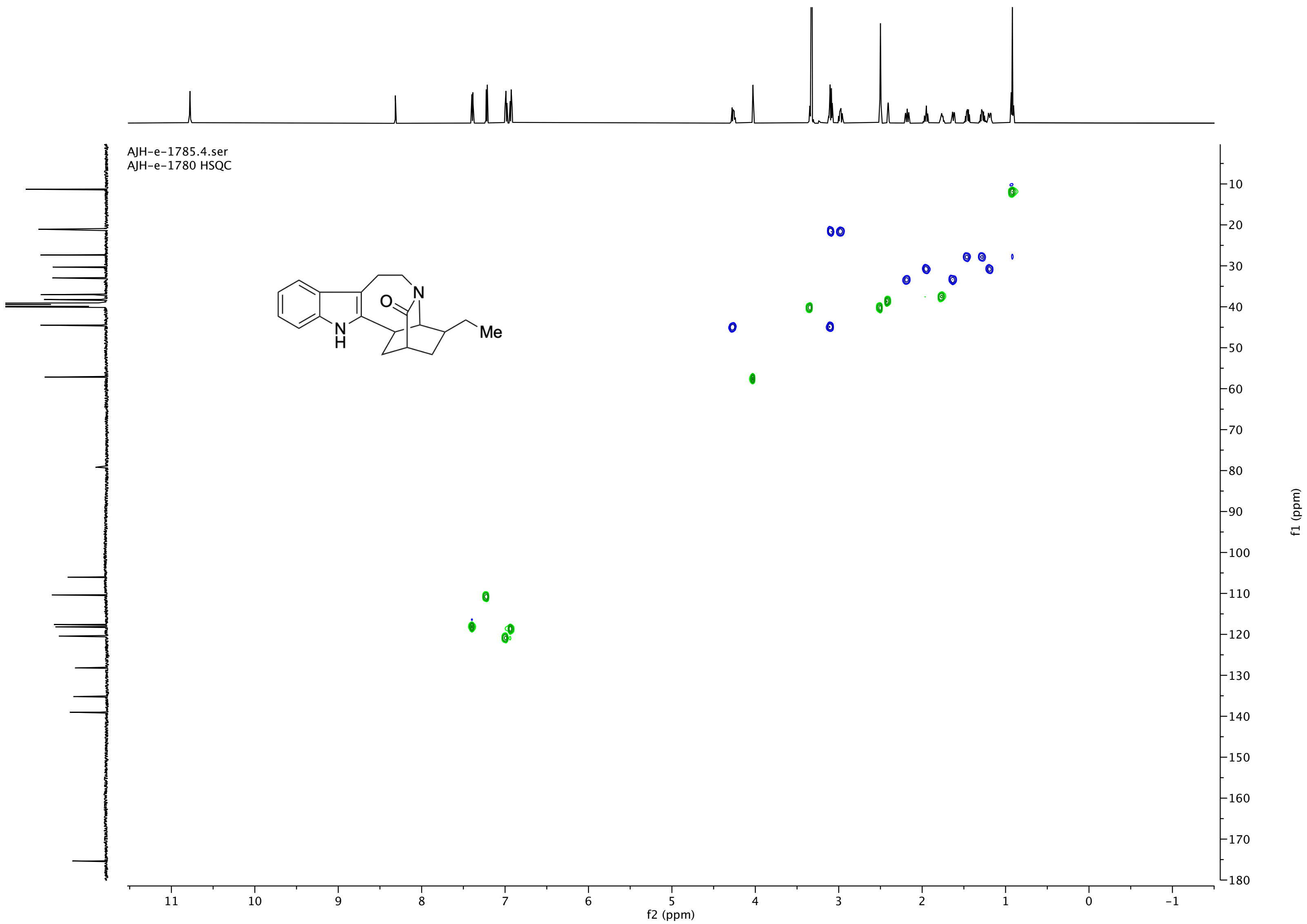

^{1}H-^{13}C HMBC NMR (DMSO) of 21

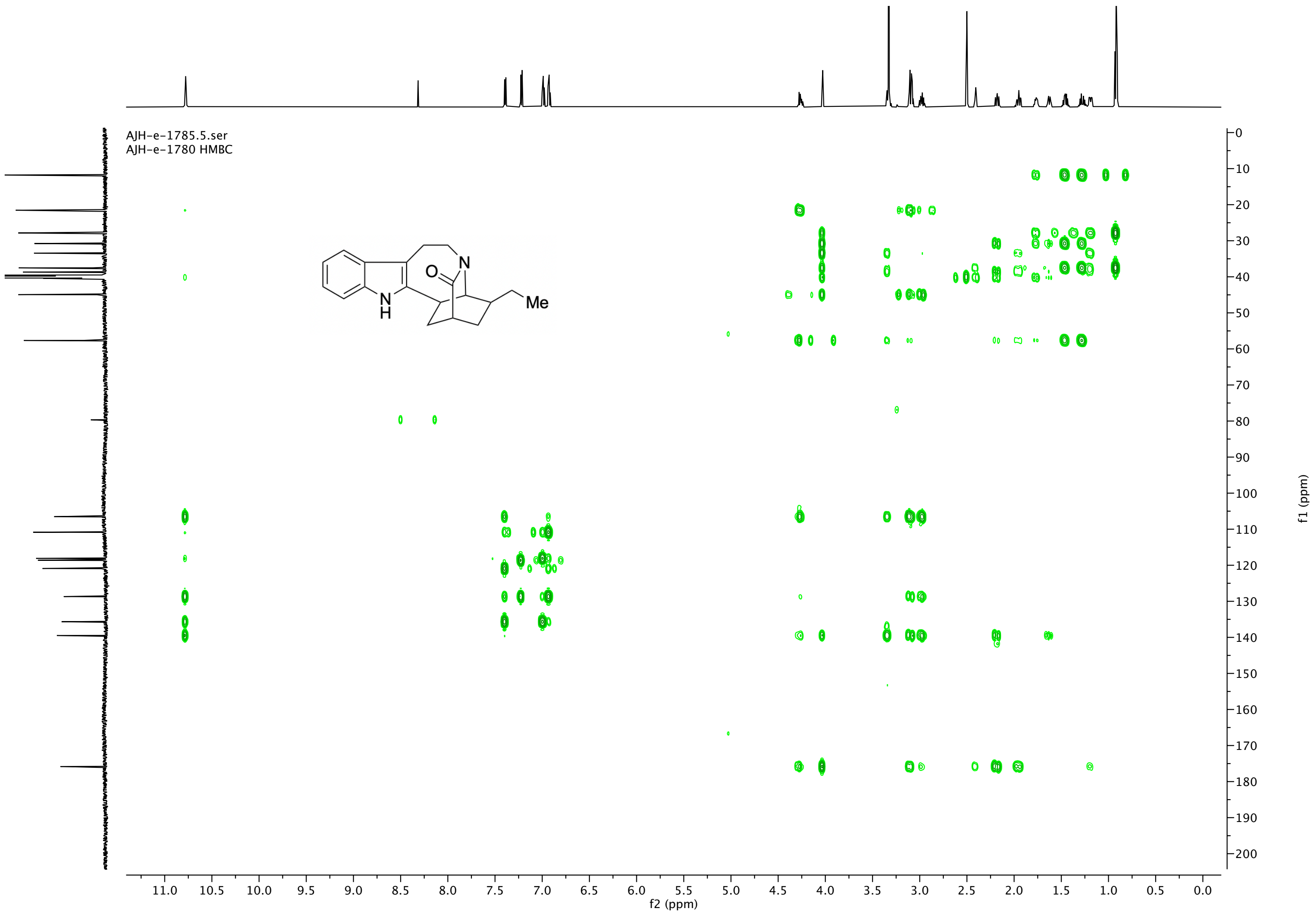

^{1}H NMR (600 MHz, $CDCl_3$) of Compound 22

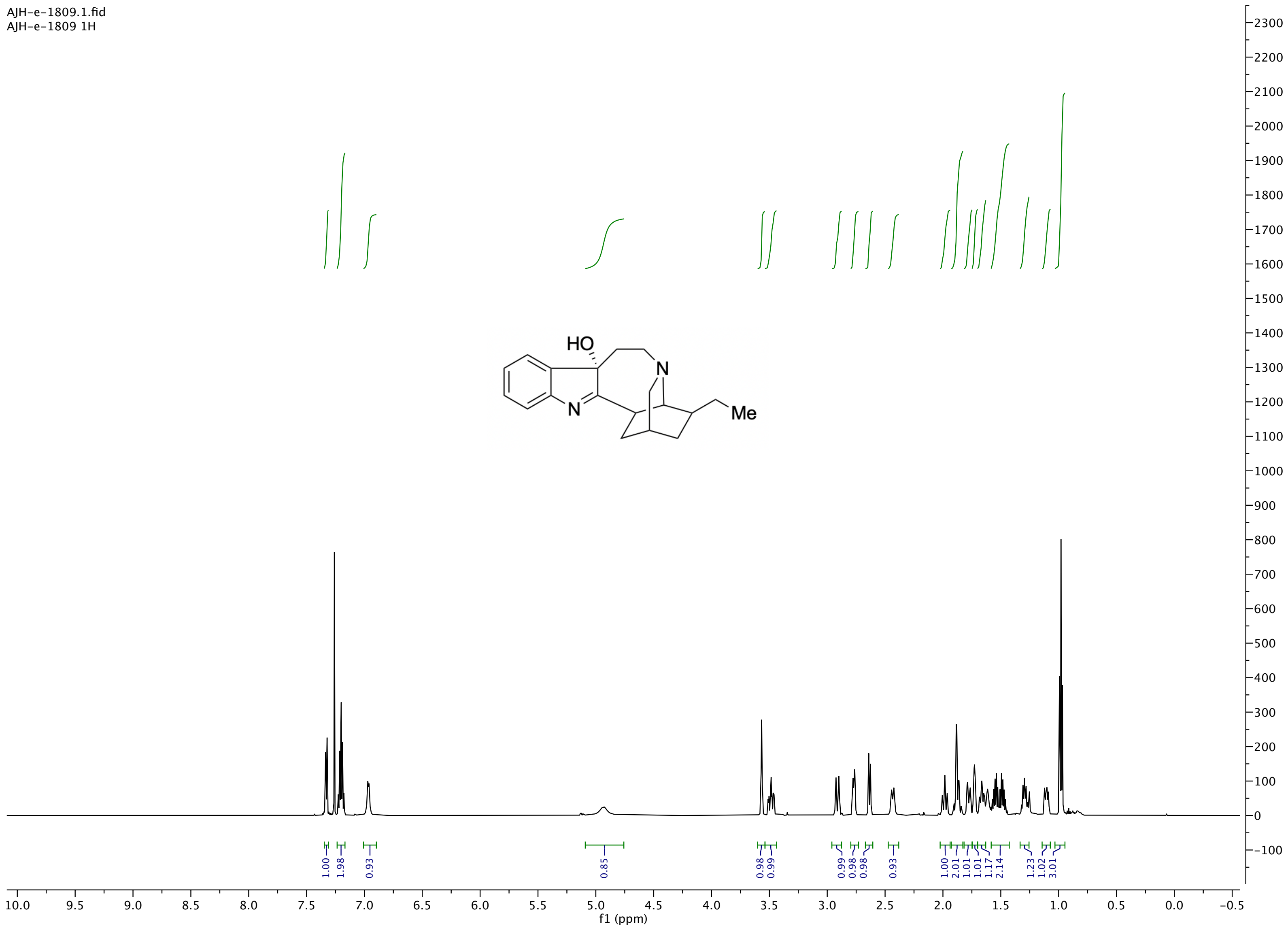

^{13}C NMR of (151 MHz, $CDCl_3$) of Compound 22

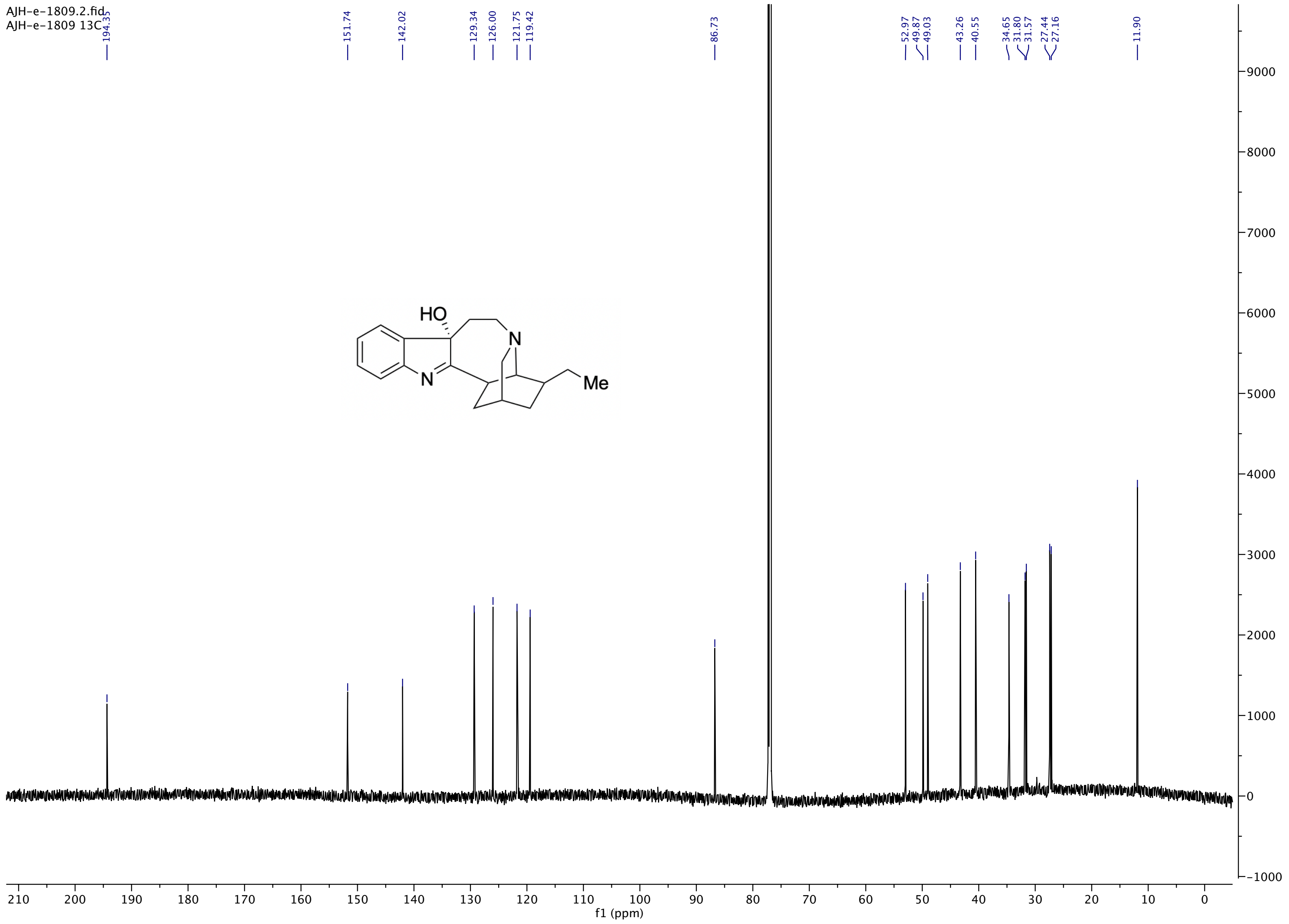

^{1}H-^{1}H COSY NMR ($CDCl_3$) of 22

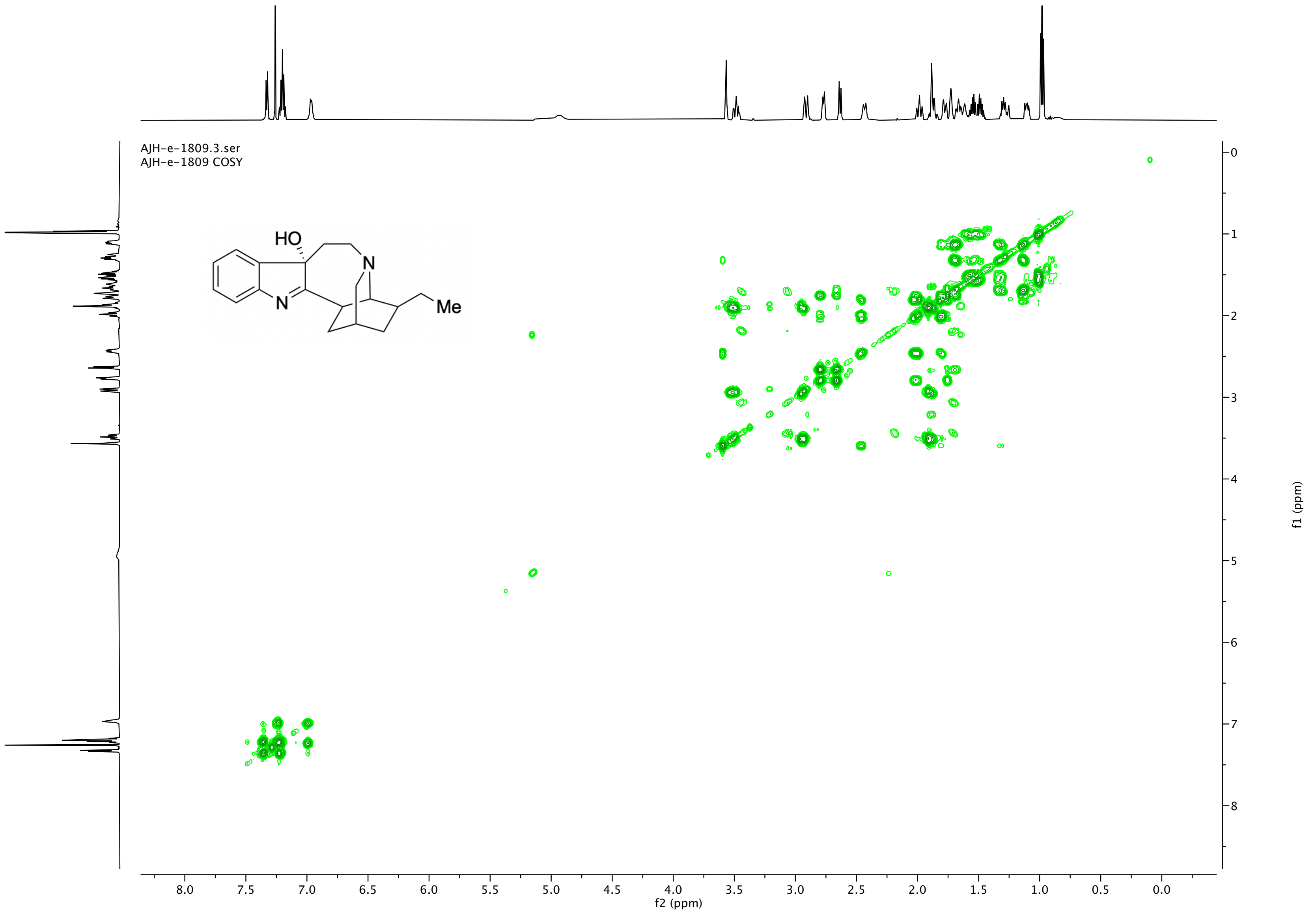

^{1}H-^{13}C HSQC NMR ($CDCl_3$) of 22

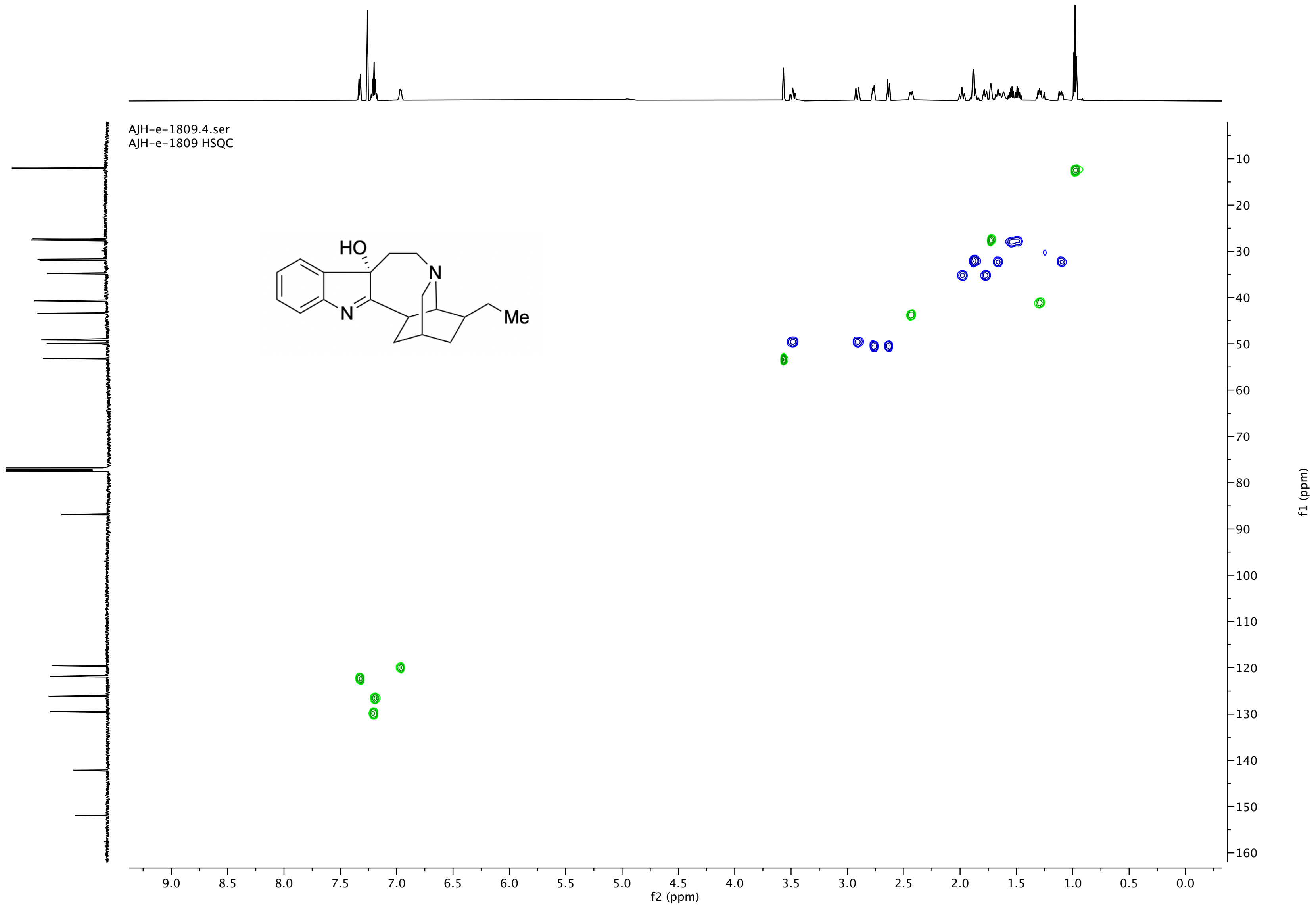

^{1}H-^{13}C HMBC NMR ($CDCl_3$) of 22

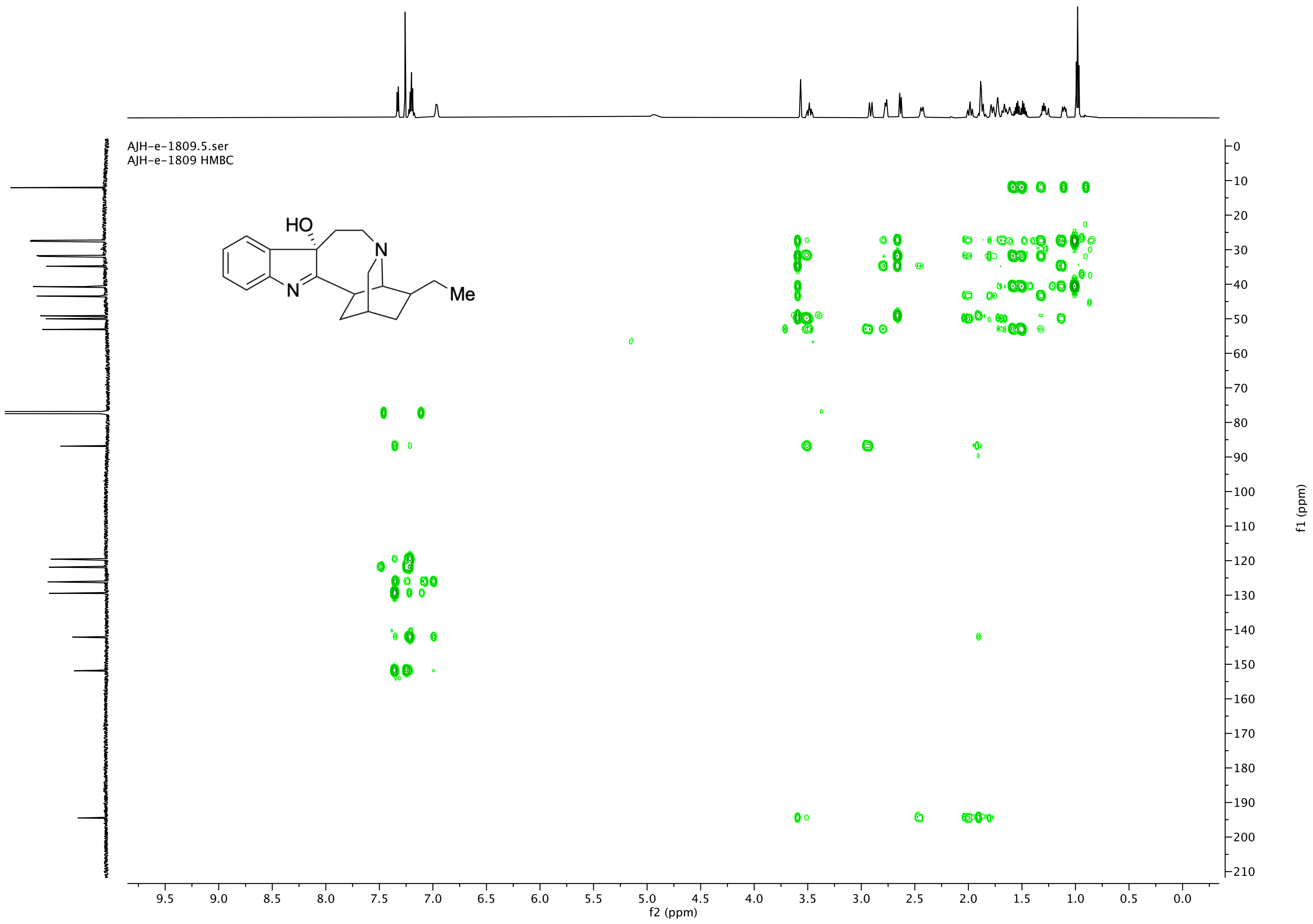

^{1}H NMR (600 MHz, $CDCl_3$) of Compound 23

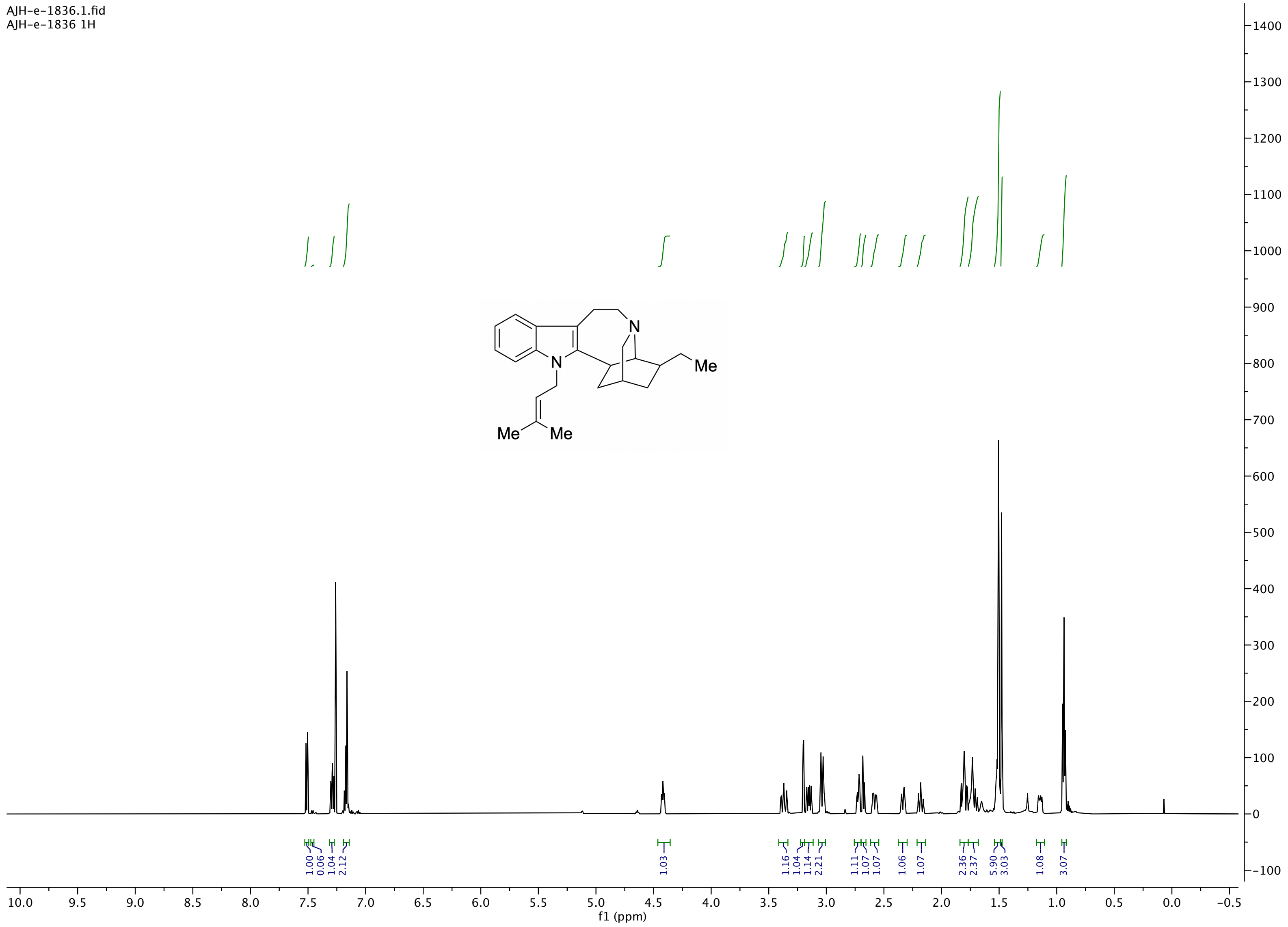

^{13}C NMR of (151 MHz, $CDCl_3$) of Compound 23

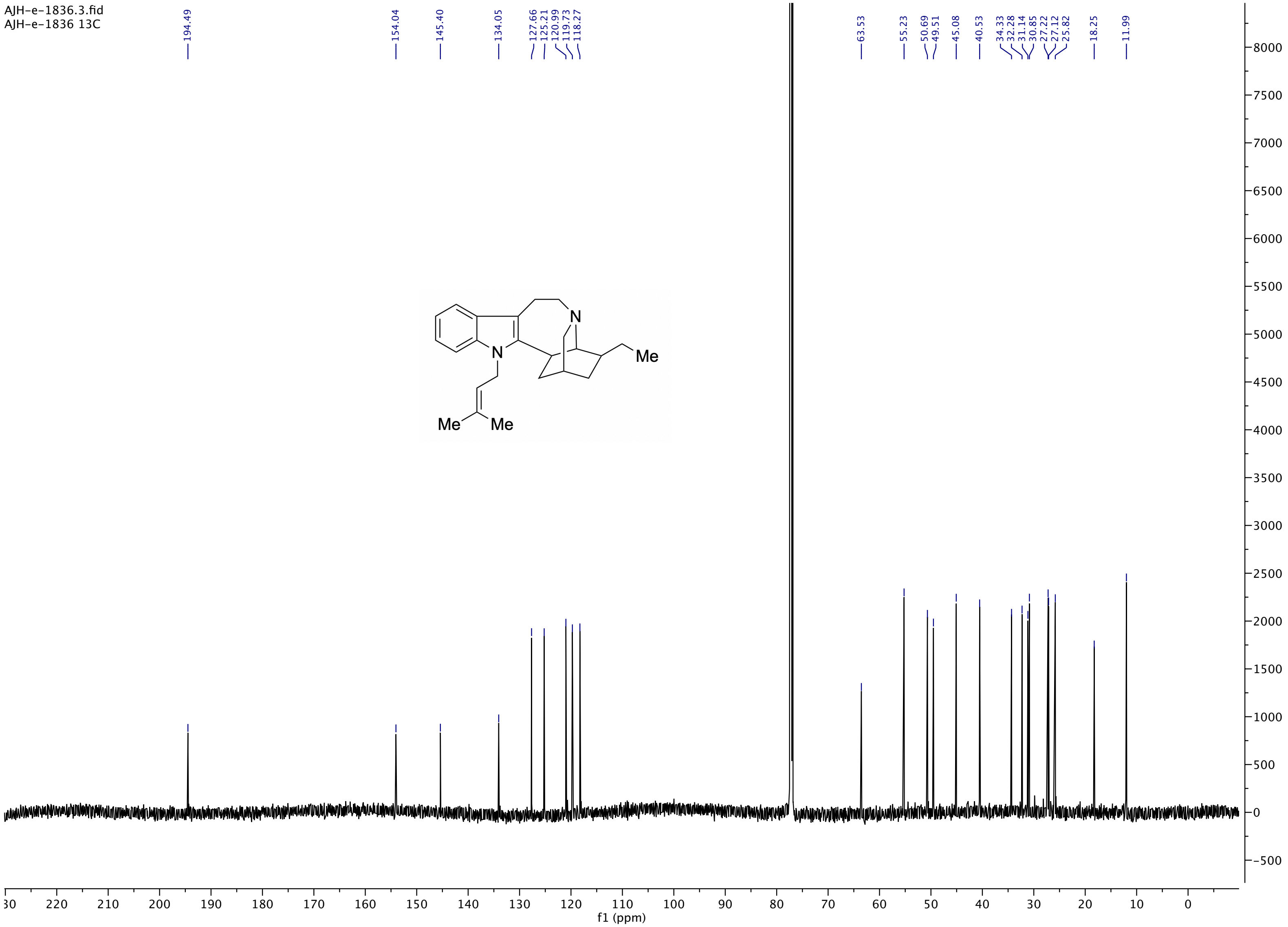

^{1}H-^{1}H COSY NMR (CDCl3) of 23

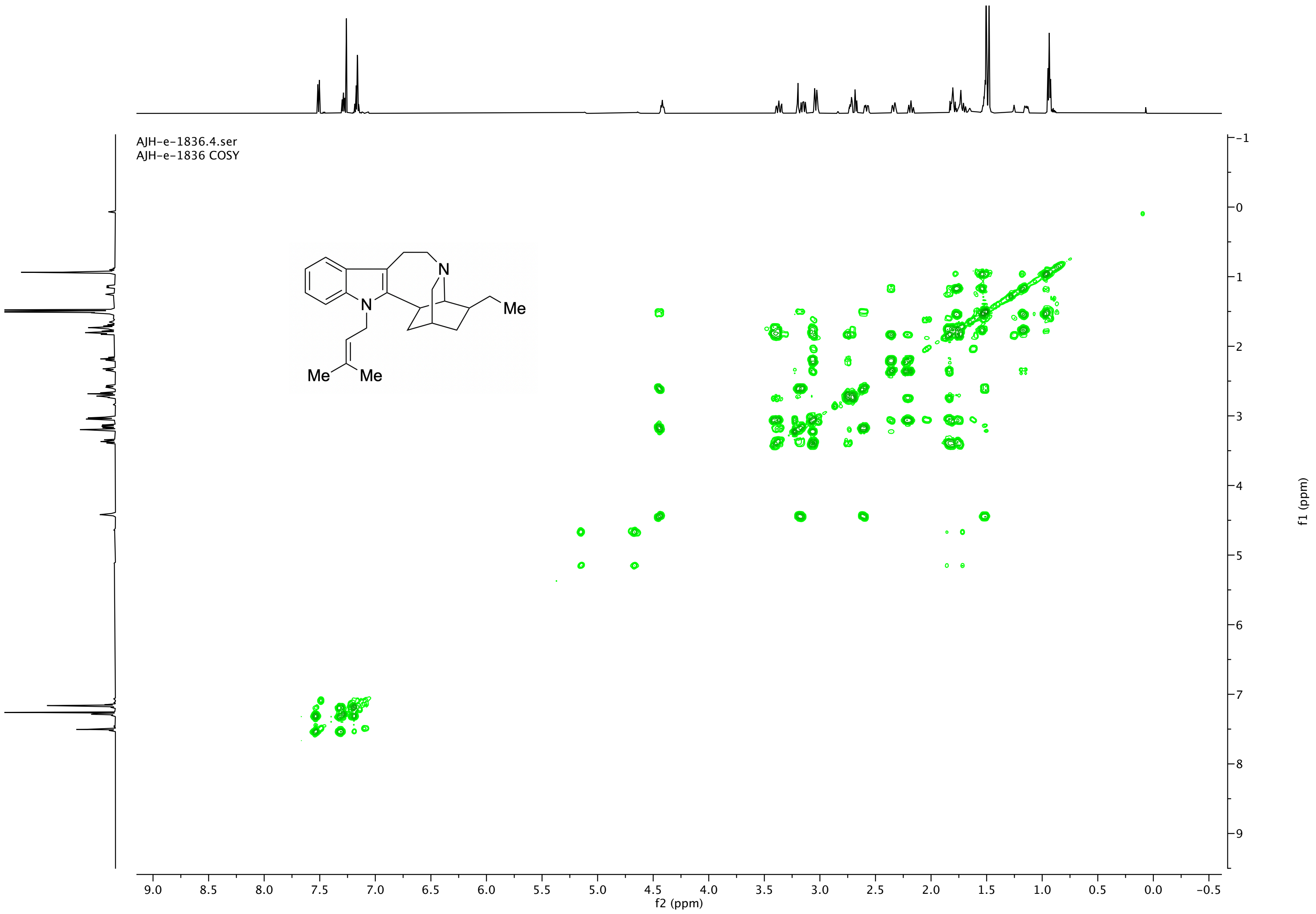

^{1}H-^{13}C HSQC NMR ($CDCl_3$) of 23

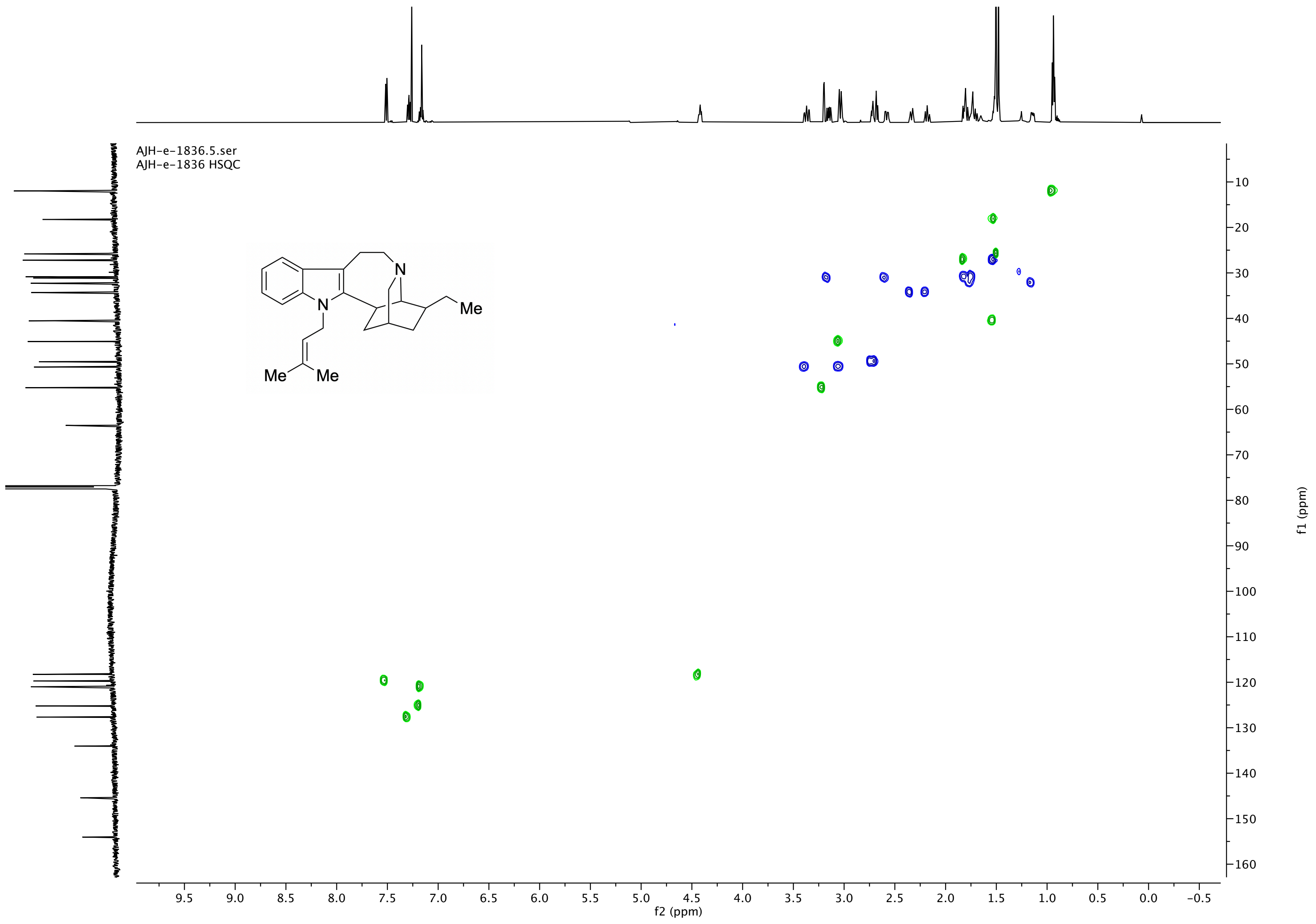

^{1}H-^{13}C HMBC NMR ($CDCl_3$) of 23

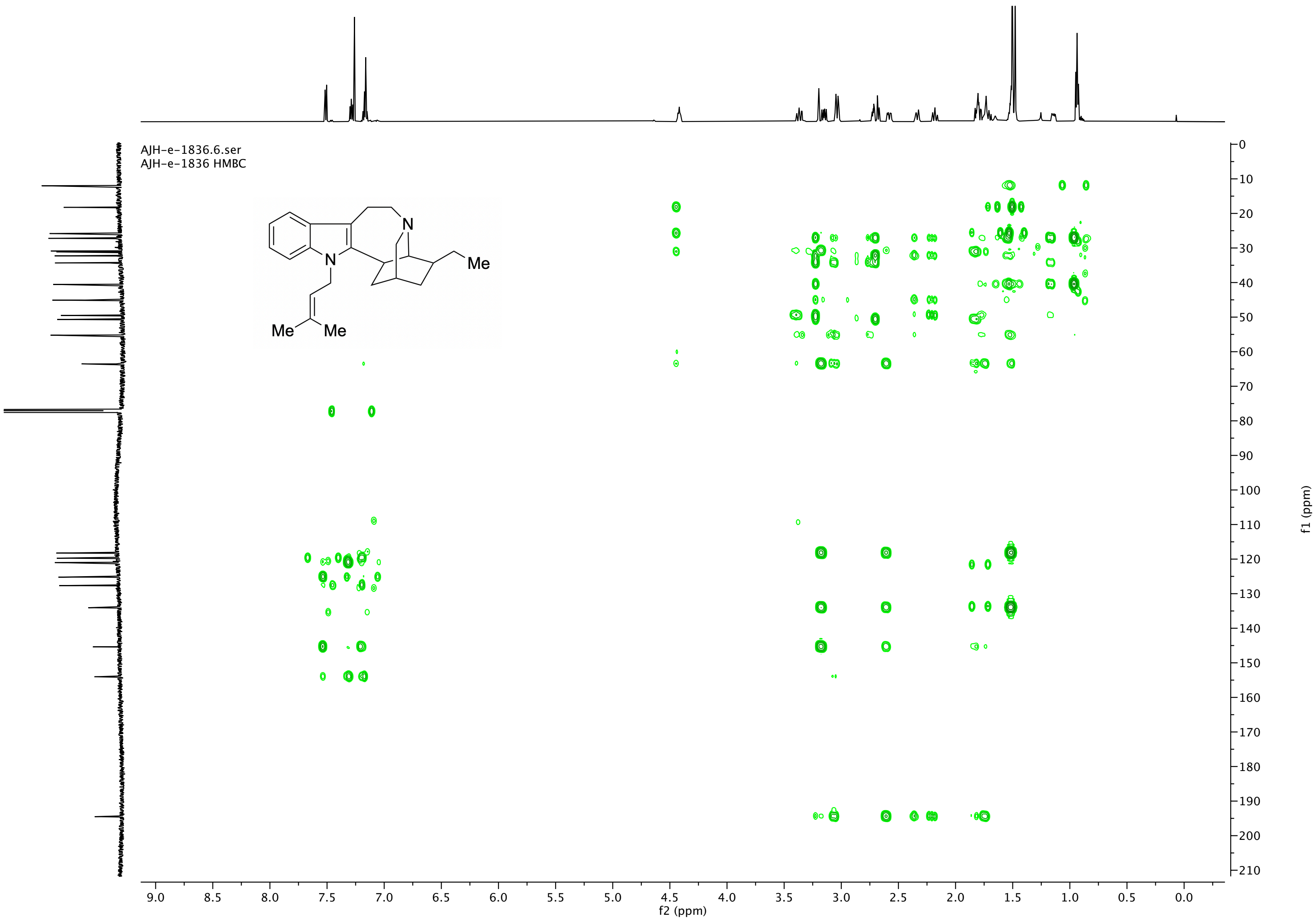

LCMS trace for compound 7e

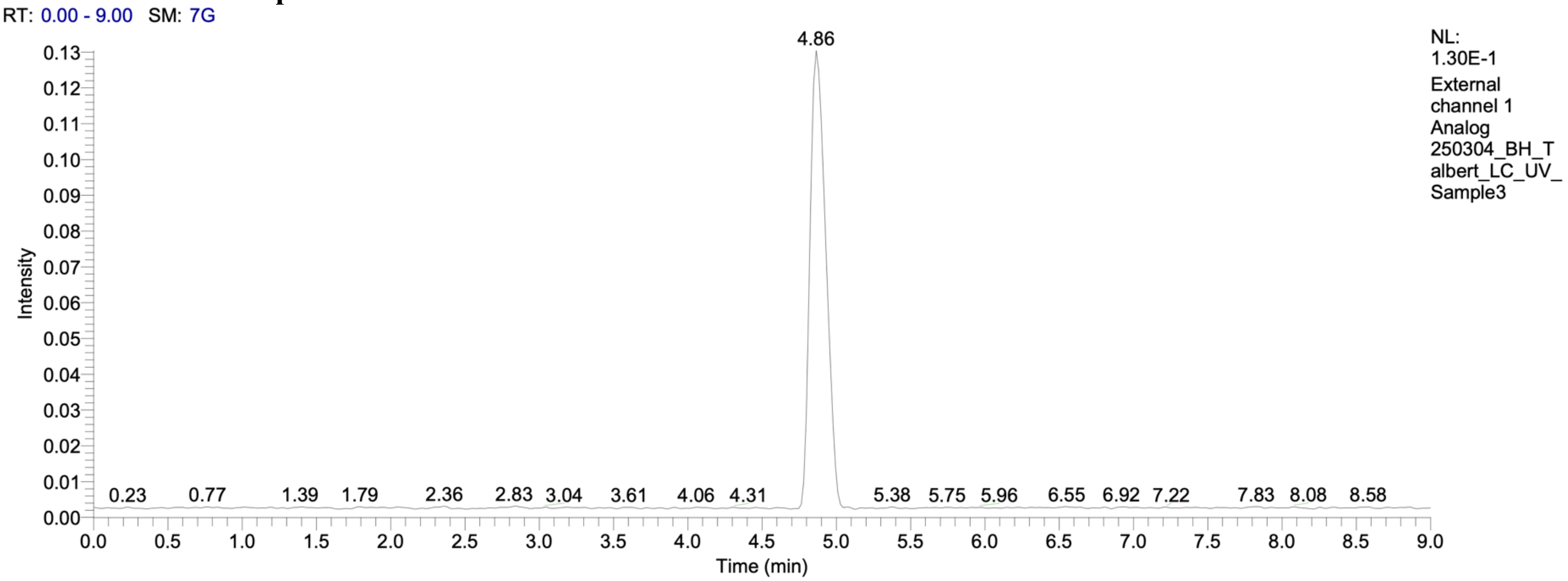

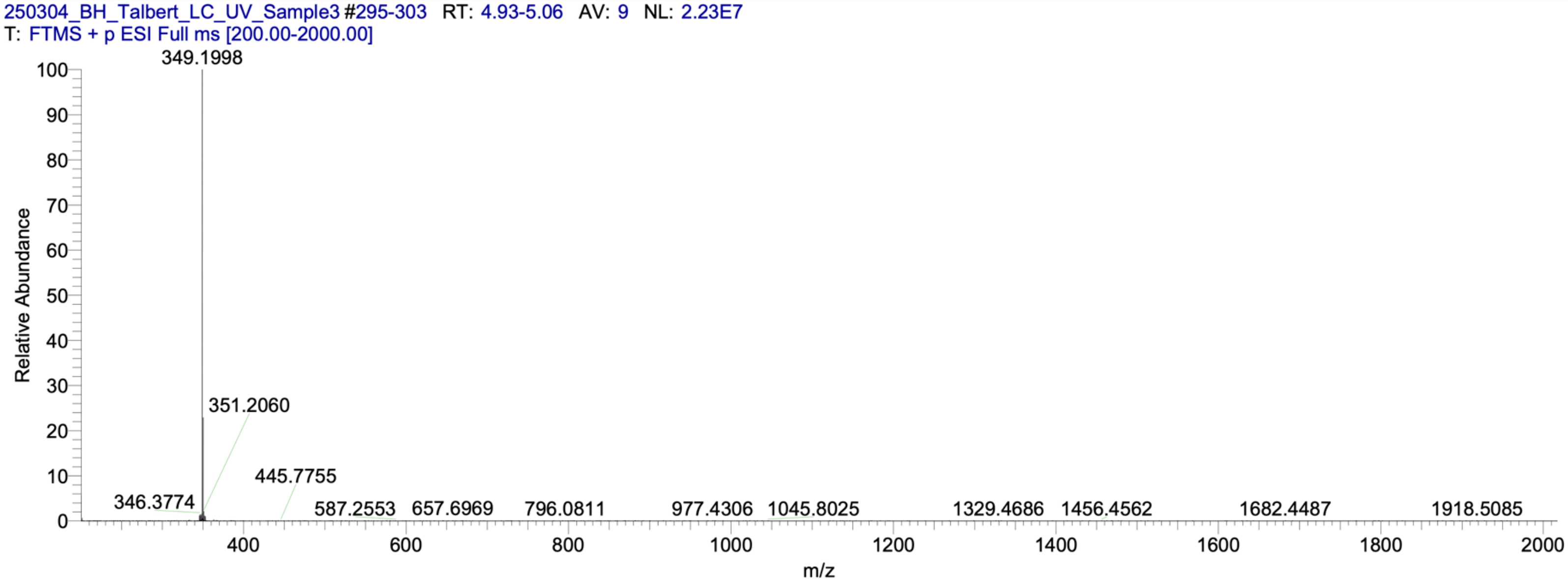

LCMS trace for compound 7i

RT: 0.00 - 9.00 SM: 7G

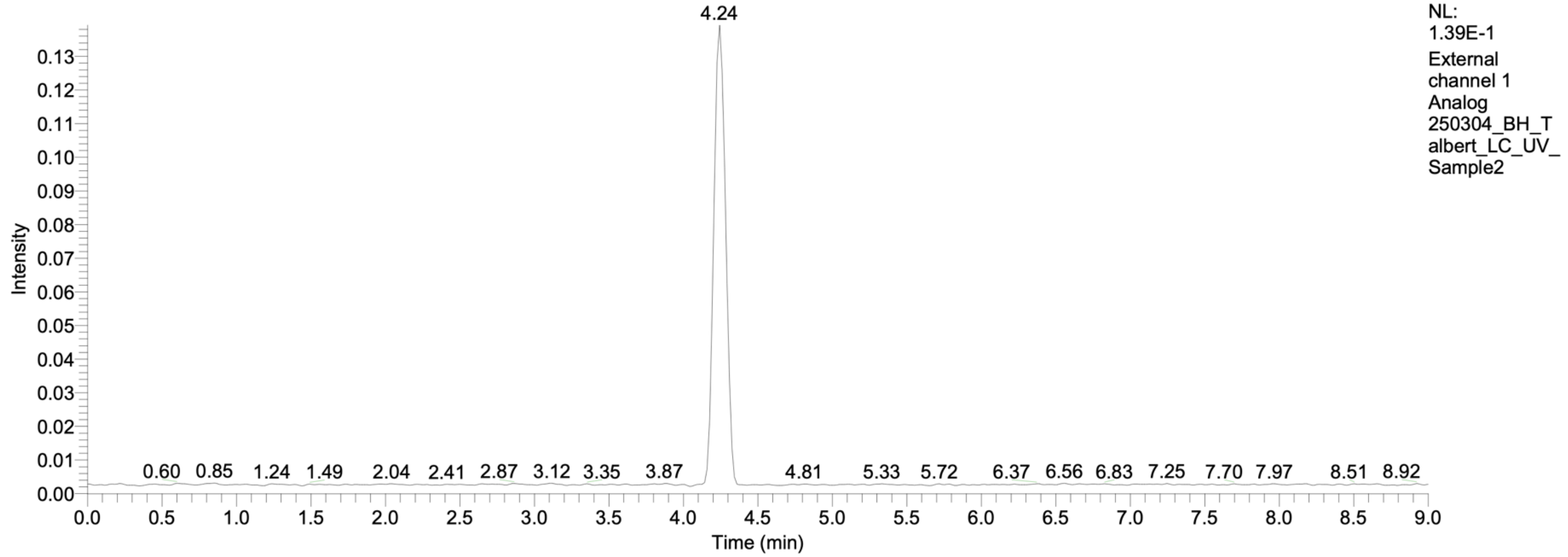

250304_BH_Talbert_LC_UV_Sample2 #255-267 RT: 4.26-4.46 AV: 13 NL: 9.02E6

T: FTMS + p ESI Full ms [200.00-2000.00]

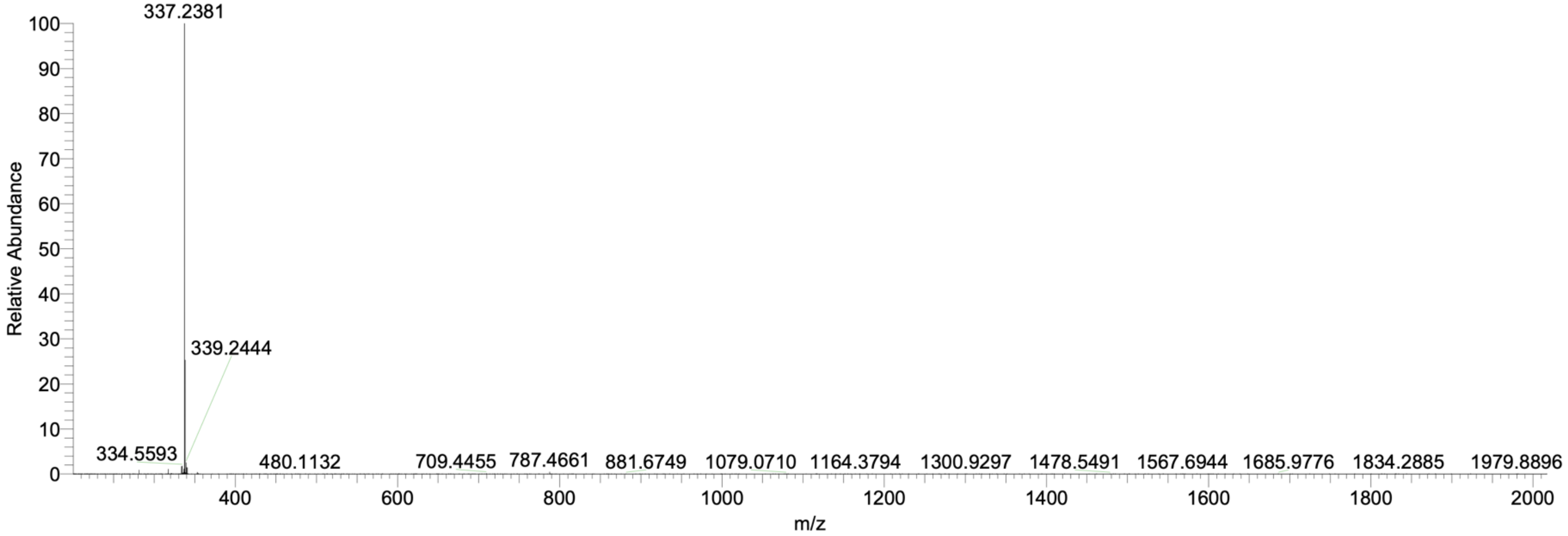

Method: Mobile phase A was 0.1% formic acid in water and mobile phase B was 0.1% formic acid in acetonitrile. Gradient was 5-95% B using an Agilent Poroshell 120 EC-C18 2.7 um 3x50mm column. UV was measured at a wavelength of 254 nm.

References

(1) Waterhouse, A.; Bertoni, M.; Bienert, S.; Studer, G.; Tauriello, G.; Gumienny, R.; Heer, F. T.; Tjaart, A.; Rempfer, C.; Bordoli, L.; et al. SWISS-MODEL: homology modelling of protein structures and complexes. *Nucleic Acids Research* **2018**, *46* (W1), W296-W303.
(2) Hanwell, M. D.; Curtis, D. E.; Lonie, D. C.; Vandermeersch, T.; Zurek, E.; Hutchison, G. R. Avogadro: an advanced semantic chemical editor, visualization, and analysis platform. *J Cheminf* **2012**, *4* (1), 17.
(3) Eberhardt, J.; Santos-Martins, D.; Tillack, A. F.; Forli, S. AutoDock Vina 1.2.0: New Docking Methods, Expanded Force Field, and Python Bindings. *JCIM* **2021**, *61* (8), 3891-3898.
(4) Trott, O.; Olson, A. J. AutoDock Vina: Improving the speed and accuracy of docking with a new scoring function, efficient optimization, and multithreading. *J Comput Chem* **2010**, *31* (2), 455-461.
(5) Cui, D.-M.; Ke, Y.-N.; Zhuang, D.-W.; Wang, Q.; Zhang, C. Gold-catalyzed hydrative cyclization of 1,6-diynes in ionic liquid media. *Tetrahedron Letters* **2010**, *51* (6), 980-982.
